# The metabolic and anatomical complexity of root microhabitats modulate their interaction with the microbiota

**DOI:** 10.1038/s41467-026-76440-4

**Published:** 2026-08-13

**Authors:** Juan P. Frene, Valéria Custódio, Helena Rouco, Sandra Martinez-Jarquin, Yi-Qun Gao, Vincenzo di Bari, Niokhor Bakhoum, Trent R. Northen, Benjamin P. Bowen, Katherine B. Louie, Katerina Velchova, Alexander Ware, Anthony Bishopp, Alvaro Mata, Gabriel Castrillo

**Affiliations:** 1 School of Biosciences, University of Nottingham, Sutton Bonington, UK; 2 School of Pharmacy, University of Nottingham, Nottingham, UK; 3 Biodiscovery Institute, University of Nottingham, Nottingham, UK; 4 DOE Joint Genome Institute, Lawrence Berkeley National Lab, Berkeley, CA USA; 5 Environmental Genomics and Systems Biology Division, Lawrence Berkeley National Lab, Berkeley, CA USA; 6 Department of Chemical and Environmental Engineering, University of Nottingham, Nottingham, UK; 7 Present Address: Agricultural Science Centre, New Mexico State University, Clovis, NM USA; 8 Present Address: UFR Social and Environmental Sciences, Department of Environment Biodiversity Sustainable Development, Kaolack/Joint Microbiology Laboratory, Sine-Saloum El Hadj Ibrahima University NIASS, BP 1386, Dakar, Senegal

**Keywords:** Plant morphogenesis, Secondary metabolism, Microbiome

## Abstract

Plant roots constantly communicate with their microbiota, adapting their anatomy to facilitate microbial colonisation under abiotic stresses. Microbes, in turn, can reshape root anatomy once they establish. However, the mechanisms that coordinate this interplay remain largely unknown. Working with the aquatic plant family *Lemnaceae*, we reveal that the inherent complexity of root anatomy determines root plasticity in response to microbial colonisation. This microbiota-driven anatomical plasticity enhances plant survival in nutrient-competitive environments. By combining synthetic root models with real roots, we also find that anatomical plasticity is associated with metabolic reprogramming during microbial establishment. Moreover, we identify a plant metabolite, N^6^,N^6^,N^6^-Trimethyl-L-lysine, that regulates anatomical plasticity in response to microbial colonisation. Our work generalizes the importance of microhabitat complexity for microbiome recruitment under challenging environmental conditions.

## Abstract (web summary)

In *Lemnaceae*, inherent root anatomy complexity determines microbiota-driven plasticity, enhancing survival in competitive environments. This plasticity links to metabolic reprogramming and is regulated by the metabolite N^6^,N^6^,N^6^-Trimethyl-L-lysine.

## Introduction

Plant roots, which are functionally similar to the animal gut^1^, are colonised by communities of diverse and metabolically active microbes. The processes of root microbiota colonisation and establishment are driven by intricate interactions among the host, the microbiota, its constituent members, and the environment^2–6^. These interactions enable microbes to simultaneously adapt to and remodel the physical and chemical properties of the microhabitats within the roots^2,7–9^, likely through mechanisms that have evolved over millions of years as root-associated microbes engaged with host-originated signals^10^. At the morphological level, host traits such as root surface area and diameter^11,12^, root type^13^, number of lateral roots^14^, and root hairs^15^ influence the establishment of root microbial communities. Reciprocally, members of the root microbiota are capable of modifying root architecture^7,8^ and root cell anatomy^16^ in a variety of plants. However, our knowledge on how these mechanisms of microbial recruitment and establishment operate at a lower anatomical level remains limited. Only recently the ability of the root microbiota to modify root cellular anatomy has been recognised as an important mechanism for preventing the establishment of parasitic plants^17^ and for regulating mineral nutrient homeostasis^16^. In animals, mechanisms have been described that explain how physical microhabitats control the selection of bacterial symbionts and how these bacteria perform beneficial functions for the host^18^. However, in plants, the anatomical traits that influence the colonisation of beneficial microbial communities colonising root microhabitats, as well as the developmental mechanisms governing these traits, remain elusive.

Emerging theoretical and empirical studies have emphasised the pivotal influence of mycorrhizal associations on the development of root anatomical allometry, a fundamental aspect of root anatomical organization^19^. Building on this, recent theories propose a direct link between root anatomy and microbial function, offering insights into how evolutionary changes in root anatomy influence the capacity of roots to associate with beneficial microbiota^20,21^. However, most studies analysing the influence of root anatomical traits on microbiota recruitment include only a limited number of plant species^13,22–24^. Consequently, their findings may result from nonspecific trait interactions or context-dependent observations, limiting their generalization across multiple species. These challenges, together with the vast diversity of anatomical properties and functions present in angiosperms^24^, the poorly understood trade-off between anatomical traits and microbiota functions, or among anatomical traits themselves, and the effects of location and soil conditions on these interactions^24^, have hindered investigations into how root microhabitat characteristics, both anatomical and metabolic, influence microbiota establishment and vice versa.

The establishment of aquatic duckweeds (family *Lemnaceae*) as model plants, whose roots are highly reduced in morphology and anatomy compared to other flowering plant species^25^, represents a unique opportunity to study how root microhabitat anatomy and metabolic profile influence microbiota recruitment. Roots of members of this family neither branch nor form root hairs^26^. The five genera within *Lemnaceae* represent an evolutionary gradient in root number, root cell number, and root anatomical complexity^27^. For example, in order of divergence, the genera Spirodela and Landoltia possess multiple roots per frond, Lemna typically develops a single root, while Wolffia and Wolffiela are rootless^27^. We hypothesised that using *Lemnaceae* as a model under controlled conditions would allow us to test the effects of progressively reduced root anatomical traits, potential covarying trait linkages, root metabolic complexity, and environmental influences, thereby enabling the emergence of relevant trade-offs between root microhabitat properties and microbiota colonisation.

To this end, we compared 12 species and 6 accessions of *Lemnaceae* with varying root anatomical complexity to uncover the mechanisms regulating root anatomical plasticity in response to microbial colonisation. We revealed that root anatomical plasticity can be influenced by the microbiota under nutrient-limited conditions, promoting plant survival. Using both real roots and “synthetic root” models, we identified trade-offs between anatomical and metabolic changes and the resident microbiota. Further, this study identifies a plant-derived metabolite, N^6^,N^6^,N^6^-Trimethyl-L-lysine, as a key component of the mechanisms controlling microbiota-driven root anatomical plasticity. The discovery of these mechanisms may have important implications for breeding programs or microbe-based strategies aimed at improving beneficial microbial colonisation of plant roots.

## Results

### Root complexity influences microbiota recruitment and plant survival

**Fig. 1:**
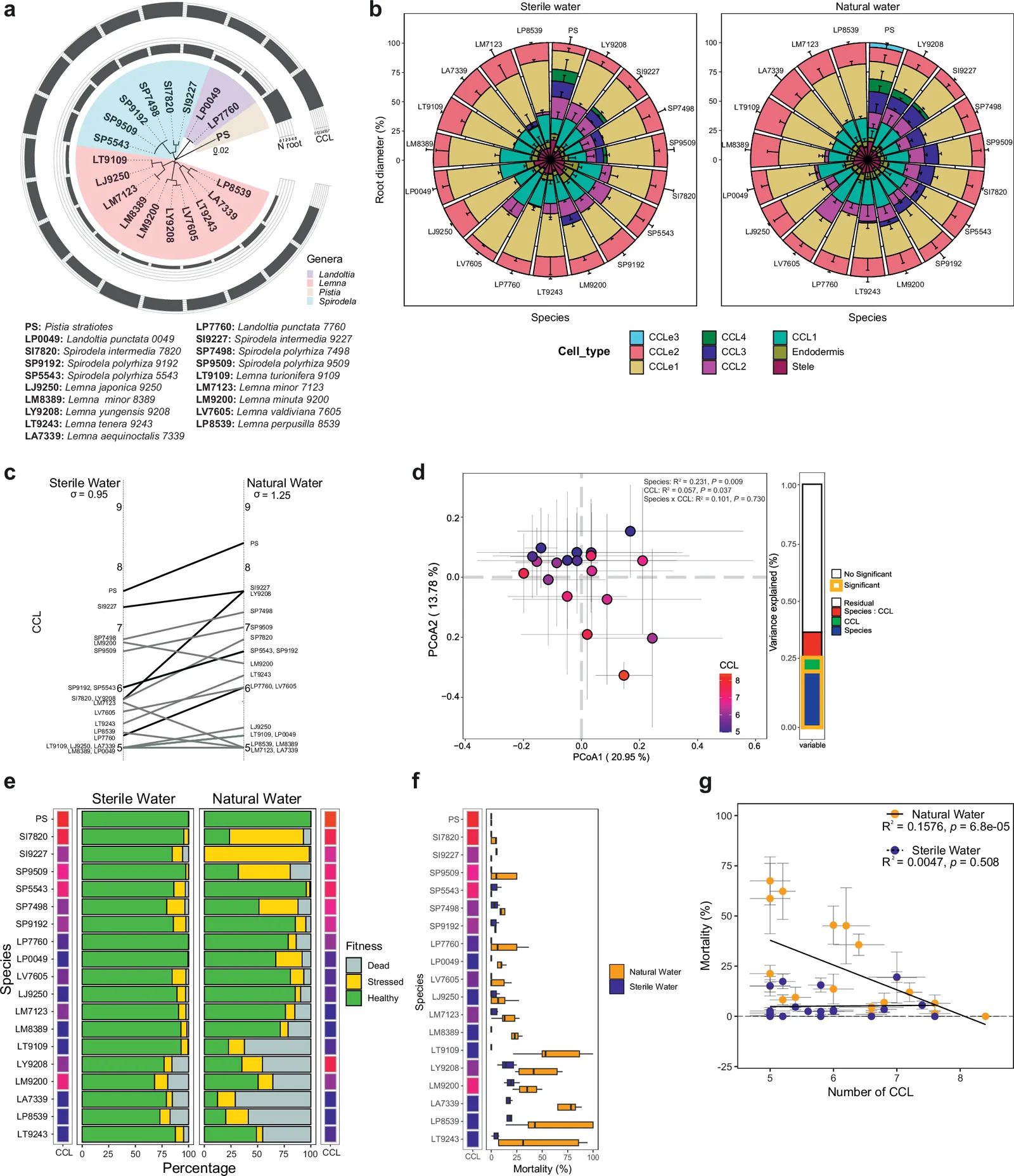
Anatomical plasticity of roots increases in response to the microbiota under nutrient-limited conditions. **a** Phylogenetic tree showing the distribution of *Lemnaceae species* and *Pistia stratiotes* used in this work. The tree was built using variations in the chloroplast *RPL16* gene sequences, encoding the L16 ribosomal protein, a component of the large (50S) ribosomal subunit in chloroplasts of all plants used. The inner and the outer rings represent the total number of roots and root cell layers (CCL), respectively, found in these plants grown in sterile natural water. Background colours represent plant genera. **b** Rose diagram displaying the average percentage diameter of each root cell layer (stele, endodermis, cortical cell layer 1 to 4 (CCL1 to CCL4) and external cortical cell layer 1 to 3 (CCLe1 to CCLe3) quantified from confocal images of root sections of *Lemnaceae* species and *Pistia stratiotes* grown in sterile (left) and natural (right) water with the microbiota present. Error bars in the diagram represent standard deviation, *n* = 5. **c** Comparative analysis of the number of root cell layers (CCLs) in the collection of *Lemnaceae* species and *Pistia stratiotes* grown in sterile and natural water. The line connecting both values is the difference between the number of CCLs in plants grown axenically or with the microbiota present. A black line indicates statistical significance (*p* < 0.1) determined via a linear model. The variance for each microbial treatment is indicated at the top of the panel, *n* = 5. **d** Principal coordinates analysis showing the projection of the root bacterial composition profile of *Lemnaceae* species and *Pistia stratiotes* plants grown in natural water. Each point represents a plant species, and they are coloured according to the number of root cell layers (CCLs) in each plant. The error bars represent standard deviations (SD). The bar on the right denotes the percentage of the variance explained by the experimental variables using a Permutational Multivariate Analysis of Variance (PERMANOVA). Significant variables are highlighted in orange (*p* < 0.05, *n* = 5 except for LM9200, LM8389, LP7760, LT9243 where *n* = 4). **e** Stacked bar plot showing the percentage of dead (grey), stressed (yellow), and healthy (green) plants quantified for each plant species used in this work grown in sterile and natural water, *n* = 5. **f** Box plots showing the percentage of dead plants quantified for each plant species grown in sterile and natural water. Box plots show the median (centre line), the first and third quartiles (box boundaries), and whiskers extending to the most extreme data points within 1.5× the interquartile range, *n* = 5. The heatmaps on the left and right of panels e and f represent the number of cell layers counted in each plant root grown in sterile (SW) and natural water (NW), respectively. **g** Linear regression analysis between the anatomical feature, number of root cell layers (CCLs), and the percentage of mortality for each plant species used grown in sterile (blue) and natural (orange) water. Error bars represent the standard deviation (SD), *n* = 5. The coefficient of determination R^2^ and its associated *p* value are also shown in each case.

We evaluated changes in root anatomical plasticity in a collection of plants from the *Lemnaceae* (duckweed) family (Fig. 1a) available in our laboratory, grown in a natural water collected from Attenborough Nature Reserve, UK, harbouring a natural microbiota (Supplementary Fig. 1a). This collection of 12 species and 6 accessions represents a gradient in the number of roots and in inherent root anatomical complexity, spanning from the earliest-diverging genera (Spirodela and Landoltia) with multiple roots and greater number of root cell layers to the later-diverging genus Lemna with a single root and a reduced number of root cell layers (Fig. 1a, b and Supplementary Fig. 1b). None of the genera in this family have the ability to branch or form root hairs^27^. To further increase root number and complexity in our analysis, we included *Pistia stratiotes*, another aquatic member of the *Araceae* family, which shares a degree of morphological similarity with the *Lemnaceae* but has a more complex inherent root anatomy, including features like branching. We grew plants in natural water and in the same water sterilized to eliminate microbiota present. We confirmed, in plants grown in sterile water, a positive correlation between number of roots and features related to inherent anatomical complexity, such as the number of cells in the external cortical cell layer 1, external cortical cell layer 2, and cortex^27^ (Fig. 1a, b and Supplementary Fig. 2). Thus, roots of plants with a small number of roots were less anatomically complex compared with plants with a higher number of roots (Fig. 1a, b and Supplementary Fig. 2). We observed that these differences in anatomical complexity across plant roots further increased in plants grown in the natural water, with the microbiota present (Fig. 1b, c, Supplementary Fig. 2, Supplementary Fig. 3 and Supplementary Fig. 4). This plasticity was less obvious in species with initially simpler inherent anatomy (Fig. 1b, c, Supplementary Fig. 1b, Supplementary Fig. 2, Supplementary Fig. 3, Supplementary Fig. 4 and Supplementary Fig. 5). These findings suggest a link between the inherent anatomical complexity of root microhabitats and the microbiota colonising them in plant roots grown in natural water.

To explore the possible relationship between root microbiota composition and root anatomical complexity, we determined root and frond bacterial community composition using *16S rRNA* gene amplicon sequencing. Significant differences in root and frond alpha diversity were detected in only a small number of the plant species analysed (Supplementary Fig. 6a). For example, in the case of root alpha diversity, we observed significant differences between the group of *Pistia stratiotes* and *Lemna tenera* 9243 and the rest of the plants analysed. In the fronds, only *Pistia stratiotes* and *Lemna yungensis* 9208 showed significantly different alpha diversities (Supplementary Fig. 6a). However, we observed a significant positive linear correlation (R^2^ = 0.2599, *p* = 0.0258) between the root alpha diversity index and the number of root cell layers, a proxy for root anatomical complexity, across all plant species used (Supplementary Fig. 6b). This correlation was not significant when we used the frond alpha diversity (R^2^ = 0.0591, *p* = 0.3465) (Supplementary Fig. 6b). This might suggest that root anatomical complexity could have an impact on the bacterial ability to colonise roots.

A principal component analysis using all *Lemnaceae* species showed significant differences in bacterial community composition across the samples. Most of the variance observed between samples was primarily explained by the variable “species” and, to a lesser extent, by the sample fractions (root, frond, and water) (Supplementary Fig. 6c). Supporting this observation, significant differences in microbial community membership across sample fractions were found in only 6 out of the 19 individual species analysed, suggesting that, under our conditions, the microbial filtering mechanisms present in the organs of these aquatic plants were less stringent compared to terrestrial plants^28,29^ (Supplementary Fig. 6c and Supplementary Fig. 7). This is consistent with the progressive anatomical and functional reduction found in the roots of some plants present in the *Lemnaceae* collection used^27^. However, we confirmed a reduced overlap in the presence of unique ASVs between water, root, and frond for all plants analysed, indicating the existence of active microbial filtering mechanisms in these plant organs (Supplementary Fig. 8).

Bacterial abundance profile analysis identified bacterial taxa such as *Proteobacteria*, *Cyanobacteria*, and *Acidobacteria* enriched in roots, *Proteobacteria* in fronds, and a depletion in *Bacteroidetes* in roots and fronds across all species compared to their abundance in water (Supplementary Fig. 9a). We confirmed these enrichments at different taxonomical levels (Supplementary Fig. 9b–d). A principal coordinate analysis (PCoA) showed significant differences in root bacterial community membership across the *Lemnaceae* species that was explained by the variables ‘species’ and ‘number of root cell layers’ (root complexity) (Fig. 1d). The ‘number of root cell layers’ did not explain the observed differences in bacterial community composition in fronds (Supplementary Fig. 10a). Thus, our findings suggest that root complexity impacts the composition of root microbial communities in *Lemnaceae* species. Supporting this hypothesis, we found a significant correlation between root anatomical feature dissimilarities and root microbiota dissimilarities (Mantel test; r = 0.118, *p* = 0.0162) in *Lemnaceae* plants colonised by microbes (Supplementary Fig. 10b). This correlation was not significant when we used frond microbiota dissimilarities (Mantel test; r = 0.06 *p* = 0.113) (Supplementary Fig. 10c). Thus, we found that microbiota-induced anatomical plasticity could influence the root’s ability to be colonised by specific microbial communities, potentially providing an advantage for plant survival in competitive environments.

We then evaluated plant growth kinetics, using total frond area as a proxy for plant growth, in natural water and in sterilised natural water (Supplementary Fig. 11a). In the timeframe analysed, all species tested showed a linear increase in plant growth in sterile water, indicating that the nutrient concentration present in the water was sufficient to support the growth of all plants during the entire experiment (Supplementary Fig. 11a and b). In contrast, in natural water, with microbiota present, frond growth followed a saturation curve, suggesting competition for resources between the plant and the microbiota, particularly at later time points (Supplementary Fig. 11a and b). We observed that ~74% of plants grown in natural water exhibited faster growth during early time points (the linear phase) compared to axenically grown plants (Supplementary Fig. 11a and b). In contrast, only 26% of the plants analysed showed similar (*Lemna yungensis* 9208, *Spirodela intermedia* 7820, *Spirodela polyrhiza* 9192, *Lemna tenera* 9243) or lower (*Lemna minuta* 9200) growth rates at early time points in natural water compared to sterile conditions (Supplementary Fig. 11a and b). The enhanced plant growth observed in plants colonised by a natural microbiota correlated significantly with 20 out of 21 anatomical traits measured in their roots (Fig. 1b, Supplementary Fig. 2, Supplementary Fig. 3b, and Supplementary Fig. 11c). We verified that, in the case of axenic plants, only 7 out of 21 anatomical traits correlated significantly with the plant growth in the linear phase (Supplementary Fig. 11c). These results suggest that microbiota-induced complex root anatomy could enhance plant growth, improving plant survival under these challenging growth conditions.

To evaluate the plant capacity to survive this competitive environment, we quantified the number of healthy, stressed, and dead plants at the end of the growth series. We found that, in general, plants with complex microbiota-induced root anatomy survived better than those plant species with less complex microbiota-induced roots in the natural water (Fig. 1e, f). In line with these observations, a significant linear correlation (R^2^ = 0.1576, *p* < 0.001) between plant survival and microbiota-induced root complexity was observed in plants grown with the microbiota (Fig. 1g). On the contrary, inherit root complexity did not impact the growth and survival rates of plant species grown in sterilized water (Fig. 1e–g). All these results strongly suggest that mechanisms controlling microbiota-driven root plasticity regulate root-microbiota interactions in *Lemnaceae* species in resource-competitive environments. The presence of these mechanisms in species with inherently relatively complex root anatomy, for example a higher number of root cell layers, could increase plant survival under nutrient-limiting conditions.

### Root complexity, and not nutrient availability, influences microbial community assembly

To further assess the coordination of root microhabitat complexity with microbial establishment in roots, we first generated a bacterial culture collection. We isolated bacterial strains from two *Lemnaceae* species, *Spirodela polyrhiza* 9509 and *Wolffia australiana* 7211, available in our laboratory, and grown in two different natural waters collected from the arboretum of the University of Nottingham, Sutton-Bonington Campus, and the Peak District National Park, UK (Supplementary Fig. 1a). Roots and frond pieces were washed, pulverised, and used to inoculate ten different agar-based selective media (Supplementary Data 1). We isolated 245 bacterial strains whose taxonomic attributes were assigned by Sanger sequencing of the bacterial *16S rRNA* gene. This taxonomic information, along with the morphology and colour of the bacterial strains were used to eliminate possible strain duplications. Our final bacterial collection comprises strains from the classes Gammaproteobacteria (63.67%), Actinobacteria (13.06%), Alphaproteobacteria (3.67%), Bacilli (15.51%), Betaproteobacteria (3.67%) and Cytophagia (0.41%) (Supplementary Fig. 12a and Supplementary Data 2).

To begin to unravel the contribution of root anatomical complexity to microbial colonisation of the roots, we selected six representative *Lemnaceae* species with high ( > 7 root cell layers in total), medium (total root cell layers between 5 and 7), and low (with <5 root cell layers in total) inherent anatomical complexity (Supplementary Fig. 12b). To emulate the wide range of nutrient availability that may be present in the natural water experiment, resulting from the different species used and their interaction with the microbiota, we grew these plant species at three nutrient concentrations using different dilutions of SH medium (1/2 SH, 1/100 SH, and 1/1000 SH) with or without inoculation with a 190-members bacterial synthetic community comprising 75.5% of the collection strains. This synthetic community approximates, at the family level, the bacterial community composition found in *Lemnaceae* species ( ~ 30% across sample compartments) grown in our experiments using a natural water (Supplementary Fig. 12c). Overall, we validated the findings of the natural water experiments (Fig. 1). Similarly to the natural water experiment (Supplementary Fig. 11), we observed that plants grown in low nutrient conditions, 1/1000 SH, inoculated with the bacterial synthetic community followed growth kinetics of saturation likely due to competition between plants and the microbes for resources (Supplementary Fig. 13). We confirmed the microbiota-driven root anatomical plasticity observed in plants with medium and high inherent root relative complexity grown with a natural microbiota (Fig. 1b, c and Supplementary Fig. 5) in plants grown in medium (1/100 SH) and low (1/1000 SH) nutrient concentrations inoculated with the bacterial synthetic community (Supplementary Fig. 14). We also replicated in our system that plant species with more relatively complex microbiota-driven root anatomy survive better than plants with simple microbiota-driven roots under limited resources (Supplementary Fig. 14 and Supplementary Fig. 15a, b). The values of all parameters analysed (plant growth, root anatomy, and plant survival) reduced with increasing amounts of nutrients available in the medium (Supplementary Fig. 14 and Supplementary Fig. 15a, b). We observed that the decrease in nutrient concentrations induced changes in root anatomy and mortality in plants grown under axenic conditions (Supplementary Fig. 14a, b and Supplementary Fig. 15a, b). However, the values of these parameters, at low nutrient concentrations, were lower compared to plants grown under the same conditions inoculated with the bacterial synthetic community (Supplementary Fig. 14 and Supplementary Fig. 15).

**Fig. 2:**
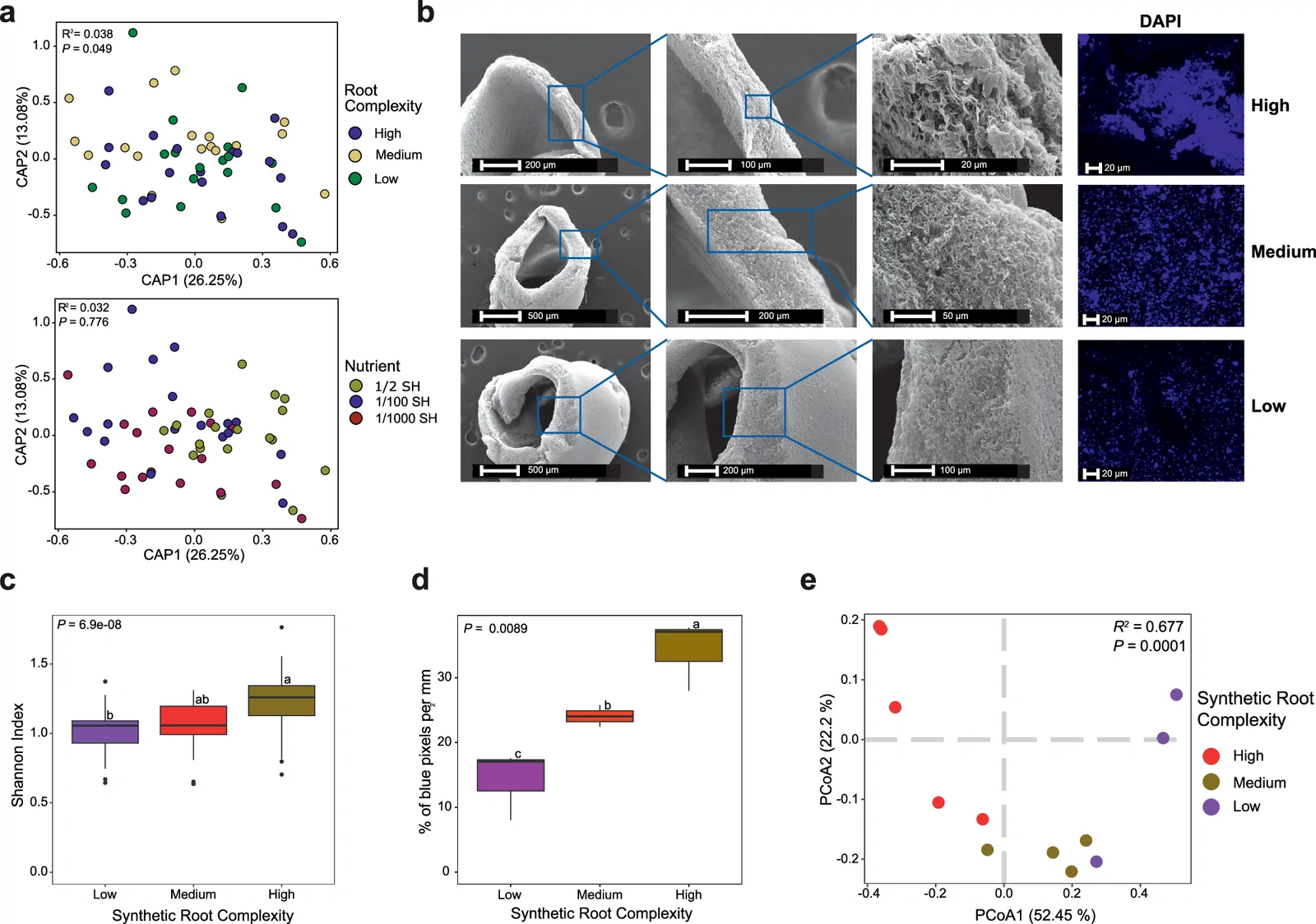
The anatomical complexity of the root influences the establishment of the microbiota. **a** Canonical analysis of principal coordinates (CAP) showing the influence of (top) root anatomical complexity (high, medium, and low) and (bottom) nutrient content in the growth medium (1/2 SH, 1/100 SH, and 1/1000 SH) on the bacterial community composition in the root. The R^2^ and *p*-values within each panel are from a PERMANOVA model and correspond to the root complexity and nutrient variables, respectively, *n* = 3. **b** From top to bottom, the panels show scanning electron microscope (SEM) images of protein-based, self-assembled “synthetic roots” with high, medium, and low complexity. From left to right, the panels show SEM images of the same “synthetic roots” taken at progressively higher magnification to highlight their detailed anatomical structure. The last column, from top to bottom, shows the bacterial communities colonising each synthetic root, visualized by DAPI staining. **c** The graph shows the bacterial alpha diversity across “synthetic roots” with different anatomical complexities (colours), estimated using the Shannon diversity index. “Synthetic roots” roots were inoculated with the bacterial synthetic community in 1/1000 SH medium. Box plots show the median (centre line), the first and third quartiles (box boundaries), and whiskers extending to the most extreme data points within 1.5× the interquartile range. Significance was determined via ANOVA followed by Tukey’s post-hoc tests (*p* < 0.05, *n* = 3), testing for two-sided differences. Different lowercase letters indicate significant changes among treatments. **d** Boxplots showing the quantification of blue pixels per mm^2^ of synthetic root surface, used as a proxy for estimating the abundance of bacteria colonising “synthetic roots” with different anatomical complexities. Bacterial colonisation was visualised using DAPI staining. Box plots show the median (centre line), the first and third quartiles (box boundaries), and whiskers extending to the most extreme data points within 1.5× the interquartile range. Statistical significance was determined via ANOVA followed by Tukey’s post-hoc test (*p* < 0.05, *n* = 3), testing for two-sided differences. Different lowercase letters indicate significant differences among treatments. **e** Principal coordinate analysis (PCoA) of the microbiota composition projection in “synthetic roots” with different complexity levels (colours), inoculated with the bacterial synthetic community and grown in 1/1000 SH medium. The PERMANOVA R² and *p* value are shown within the panel. Sample sizes for high, medium, and low synthetic root complexity are *n* = 5, 4, 3, respectively.

We evaluated the effect of nutrient availability and root anatomical complexity on the composition of the plant microbial community. A CAP analysis showed that a fraction of the variance in root bacterial community composition among plant species was explained by root anatomical complexity (R^2^ = 0.038, *p* = 0.049), but not by nutrient levels in the growth media (R^2^ = 0.032, *p* = 0.776) (Fig. 2a). In contrast, variation in bacterial community membership in fronds was primarily explained by nutrient availability (R^2^ = 0.044, *p* = 0.008) (Supplementary Fig. 16a). Supporting these observations, root microbiota composition dissimilarities significantly negatively correlated with the microbiota-driven root anatomy complexity dissimilarities only under low nutrient conditions (Mantel test; r = −0.3643, *p* = 0.0916) (Supplementary Fig. 16b, c). We did not find this significant correlation when we used the frond microbiota composition dissimilarities (Supplementary Fig. 16b, c). These findings highlight how root microhabitat anatomical traits and their plasticity help shape microbiota composition under nutrient-limited conditions.

### Anatomical and metabolic complexity controls root-microbe interactions

Given the above, we sought to further delineate how the variation in root microhabitats generated by different degrees of anatomical complexity influences root microbial colonisation. To achieve this, we first confirmed, using a different approach, that root physical and chemical properties change upon colonisation by the bacterial synthetic community. We determined the specific temperature ranges and heat flux associated with phase transitions of core root components using differential scanning calorimetry^30,31^. These parameters, which are intrinsically linked to the composition of the analysed materials and can also influence their morphology^32^, were quantified in the root of six species and four accessions of *Lemnaceae* exhibiting different levels of inherent root anatomical relative complexity. This analysis compared plants grown axenically with those inoculated with the bacterial synthetic community. By analysing thermal transitions, such as the shift of membrane lipids or starch from a solid to a fluid state, we quantified how bacterial colonisation affects the structural and metabolic stability of root tissues. Confirming our previous observations related to microbiota-driven root anatomical plasticity using confocal microscopy (Supplementary Fig. 2, Supplementary Fig. 3, and Supplementary Fig. 14a, b), the heat flux values obtained in roots colonised by the bacterial synthetic community were, in general, different for all plant species analysed compared to roots of axenically grown plants (Supplementary Fig. 17a). Supporting this, we found a non-significant correlation between heat flux peaks obtained in axenic roots with inherent root relative complexity measured as the number of root cell layers (R^2^ = 0.0438, *p* = 0.2755) (Supplementary Fig. 17b). On the contrary, this correlation was significant in roots colonised by microbes (R^2^ = 0.1343, *p* = 0.0505) (Supplementary Fig. 17c). All these results indicate that the microbiota increases anatomical complexity in roots that are inherently more relatively complex and/or alter the metabolic properties of root microhabitats.

To disentangle the effects of root anatomy and root metabolic composition on root microbial community composition, we created “synthetic roots” using a protein-based multi-component self-assembling material^33^ that forms cylindrical structures with tuneable anatomical traits such as stiffness and permeability^33–35^. With this system, we can vary the anatomical complexity of microbial microhabitats while keeping their metabolic composition constant, and vice versa. We prepared three types of “synthetic roots” with high, medium, and low anatomical complexity that were incubated in the same low-nutrient medium (1/1000 SH) and supplemented with the bacterial synthetic community (Fig. 2b and Supplementary Fig. 18a). We recapitulated with this artificial system microbial composition differences observed in real roots with different inherent anatomical complexities. Alpha diversity changed with the synthetic root complexity (Fig. 2c). As in actual roots, “synthetic roots” with different complexity also assembled different microbial communities with distinct microbial abundance (Fig. 2d, e). We concluded that in “synthetic roots”, lacking a plant immune system and with a constant metabolic composition, changes in root anatomy alone were sufficient to significantly alter microbial assembly. This supports our observations on the real roots of different *Lemnaceae* species.

Next, we evaluated the effect of different metabolic profiles on microbial assembly using the synthetic root system. To cover realistic variations in root metabolic complexity, we extracted exudates from ten *Lemnaceae* species, reproducing the diverse metabolic landscapes naturally found in these plant root exudates (Supplementary Fig. 18b, c and Supplementary Data 3). We observed that exudate metabolic composition dissimilarities significantly negatively correlated with root complexity in the plant species used (R^2^ = 0.3141, *p* = 0.001) (Supplementary Fig. 19a). This might suggest a link between the factors controlling root anatomy and those shaping root metabolism, which could, in turn, influence microbial assembly.

Thus, we compared variations in microbial composition in artificial roots exposed to nine metabolically different exudates across three levels of synthetic root anatomical complexity. We found a general trend of increasing alpha diversity with greater synthetic root complexity across most of the metabolic combinations tested (Supplementary Fig. 19b). We observed significant changes (R^2^ = 0.508, *p* = 0.001) in bacterial beta diversity only in “synthetic roots” with high anatomical complexity (Supplementary Fig. 19c). In this line, we found that the number of ASVs showing significant changes in their relative abundance compared to the control condition (without root exudates) was highest in “synthetic roots” with high anatomical complexity (Supplementary Fig. 19d). Furthermore, we confirmed that the abundance of the bacterial synthetic community assembled in these “synthetic roots” was significantly negatively correlated (R^2^ = 0.141, *p* = 2.44E-5) with the inherent complexity of the plant roots used to produce the metabolites (Supplementary Fig. 19e). Overall, these results suggest a feedback loop between root microhabitat traits, both anatomical and metabolic, and the composition of the microbiota they host. Specifically, the anatomy and the metabolic composition of different root microhabitats might influence microbial establishment, and, in turn, microbes might alter the anatomical and metabolic properties of the root.

**Fig. 3:**
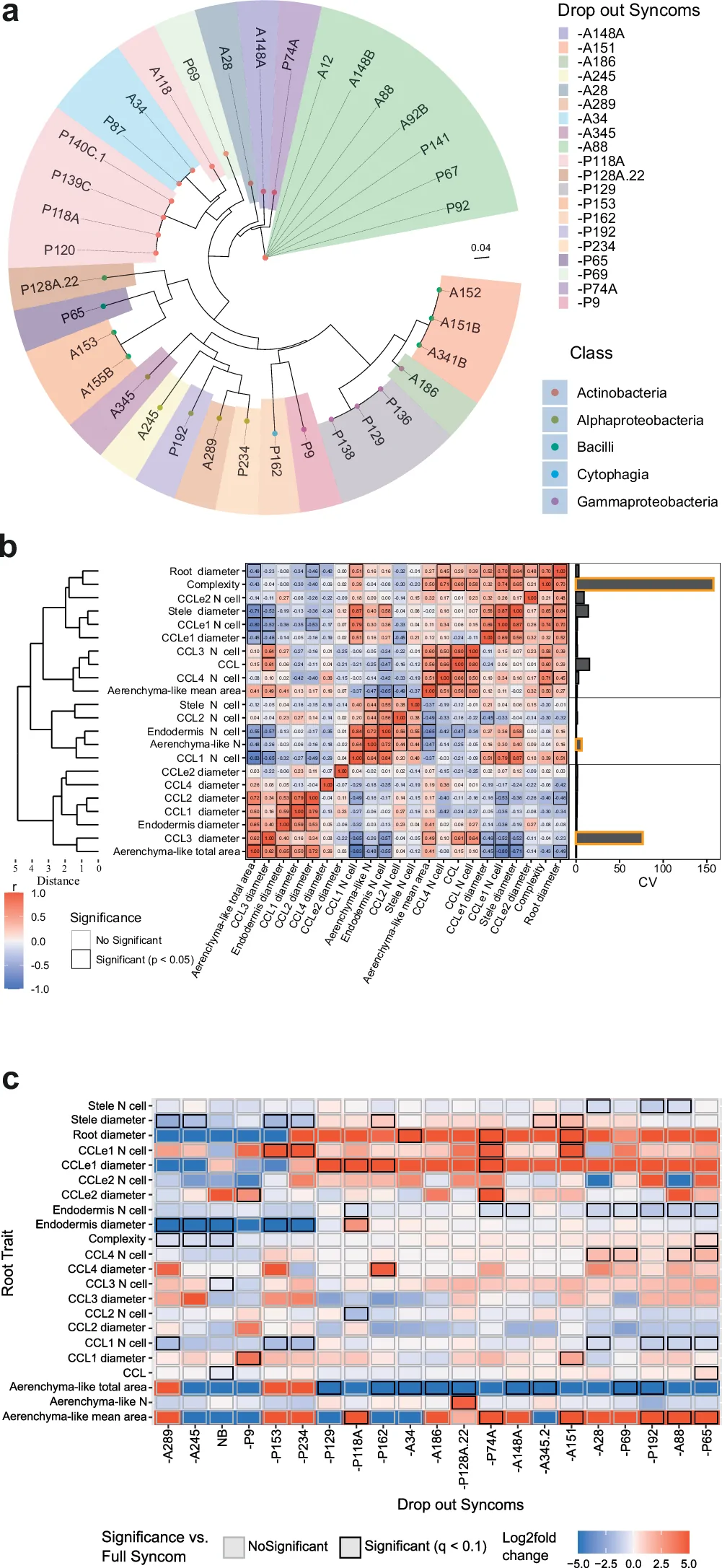
Root plasticity in plants with complex roots is mediated by the presence of specific ASVs. **a** A phylogenetic tree was built using the*16S rRNA* gene sequences of the 20 most abundant bacterial ASVs colonising the roots of the studied *Lemnaceae* species. Tree tips are coloured according to bacterial taxonomic class, with strains sharing the same colour shade belonging to the same ASV. Each of these ASVs was individually removed from the full bacterial synthetic community to generate 20 independent drop-out synthetic communities. **b** Heatmap showing the Pearson correlation coefficients for each pairwise comparison of average root anatomical features quantified in *Spirodela polyrhiza* 9509 (higher inherent root anatomical complexity) following inoculation with different bacterial synthetic communities tested (full and dropout communities). Values are clustered based on average root features. Correlation coefficient (r) values are displayed within each grid, and significant correlations are highlighted in black (*p* < 0.05, *n* = 6). The dendrogram represents hierarchical clustering of traits, and the scale indicates the distance between clusters. The bar graph on the right shows the coefficient of variation for each root trait in *Spirodela polyrhiza* 9509 in response to all bacterial synthetic community inoculations, with the highest coefficients of variation in each cluster highlighted in orange. **c** Heatmap showing the log_2_ fold-change of root trait values in *Spirodela polyrhiza* 9509 exposed to bacterial synthetic communities in which each of the 20 most abundant ASVs colonising the roots was individually dropped out, compared with root traits measured in plants exposed to the full bacterial synthetic community. Plants grown under axenic conditions (NB) were used as a control. Statistically significant enriched (red) and depleted (blue) root trait values, relative to those in plants exposed to the full bacterial synthetic community, are outlined in black (test *q* < 0.1, *n* = 6).

To validate the link between microbial composition and microbiota-driven root anatomical plasticity, we measured anatomical changes in *Spirodela polyrhiza* 9509, which exhibits high inherent root anatomical relative complexity compared to other duckweed species (Fig. 1), when exposed to different bacterial inocula. We inoculated this plant with the full 190-member bacterial synthetic community, as well as with twenty derivative bacterial synthetic communities generated by individually dropping out each of the top 20 most abundant ASVs colonising *Lemnaceae* roots (Fig. 3a and Supplementary Data 2). We quantified changes in 21 root anatomical features in response to the different microbial combinations under nutrient-limited conditions (1/1000 SH) (Fig. 3b). To avoid redundancy, we focused on anatomical features that showed high variability in response to the bacterial synthetic community treatments among groups of strongly correlated traits. We found that anatomical features such as the index of root complexity, number of aerenchyma-like structures, and number of cells in the third cortical cell layer were highly responsive to the synthetic communities (Fig. 3b). We observed that the three bacterial synthetic communities dropping out the highly abundant ASVs corresponding to the bacterial strains A289 (*Allorhizobium-Neorhizobium-Pararhizobium-Rhizobium*), A245 (*Brevundimonas*), and P65 (*Staphylococcus*) significantly modified the highly responsive parameter index of root complexity compared to plants inoculated with the full synthetic community (Fig. 3c and Supplementary Fig. 20a). Similarly, the number of aerenchyma-like structures changed significantly in response to the synthetic community that eliminated the ASV corresponding to the bacterial strain P128A.22 (*Paenibacillus*) (Fig. 3c). These results indicate that microbiota-driven root plasticity in plants with inherently complex roots depends on specific bacterial strains. We repeated this experiment using *Lemna aequinoctalis* 7339, which has a less anatomically complex root. We confirmed differences in root anatomical trait variations in response to the full and drop-out bacterial synthetic communities between *Lemna aequinoctalis* 7339 and *Spirodela polyrhiza* 9509. Indeed, we identified a different set of highly variable anatomical features (number of aerenchyma-like structures, aerenchyma-like mean area, number of cells in the endodermis, and number of cells in the external cortical cell layer 2) in *Lemna aequinoctalis* 7339 in response to the synthetic communities (Supplementary Fig. 20b). Furthermore, *Lemna aequinoctalis* 7339 showed fewer anatomical changes in response to the synthetic community treatments compared to the many variable traits observed in *Spirodela polyrhiza* 9509, which has inherently relatively complex roots (Supplementary Fig. 20c). These data confirm that baseline inherent anatomical complexity determines the magnitude of root anatomical plasticity in response to the microbiota.

Mono-association experiments with the 20 most abundant ASVs in *Spirodela polyrhiza* 9509 showed that microbial diversity is needed to trigger root plasticity in plants with inherently relatively complex roots. Mono-association experiments did not reproduce the high anatomical variability seen in drop-out experiments (Supplementary Fig. 20d), nor did they have opposite effects on root anatomy (Supplementary Fig. 20e). Similar results were observed in *Lemna aequinoctalis* 7339, which has fewer inherently complex roots (Supplementary Fig. 20f, g); however, the number of affected anatomical features was much lower than in *Spirodela polyrhiza* 9509 (Supplementary Fig. 20d–g).

Next, to confirm the proposed link between microbial composition and root anatomical and metabolic complexity, we analysed the metabolic profile of *Spirodela polyrhiza* 9509 roots colonised by the full synthetic community; by the three synthetic communities dropping out the isolates A289 (*Allorhizobium-Neorhizobium-Pararhizobium-Rhizobium*), A245 (*Brevundimonas*), and P65 (*Staphylococcus*), which significantly altered microbiota-driven root plasticity; and by the same ASVs in monoassociation experiments. As observed for root anatomy (Fig. 3c), all tested bacterial synthetic communities, and to a lesser extent, the monoassociations, caused significant changes in metabolite accumulation in *Spirodela polyrhiza* 9509 roots compared to axenic roots (Supplementary Fig. 21a, b, Supplementary Data 4 and Supplementary Data 5). In *Lemna aequinoctalis* 7339, which has roots with less inherent anatomical complexity, these microbial treatments induced smaller changes in both root anatomy and metabolite accumulation compared to axenic plants (Supplementary Fig. 20c, 21a, b, Supplementary Data 4 and Supplementary Data 5). Thus, we confirmed that microbiota-induced plasticity in complex roots is linked to metabolic changes. Based on evidence from both artificial and natural roots, we hypothesise that metabolites differing between roots of varying inherent anatomical complexities might control microbial assembly in root microhabitats.

**Fig. 4:**
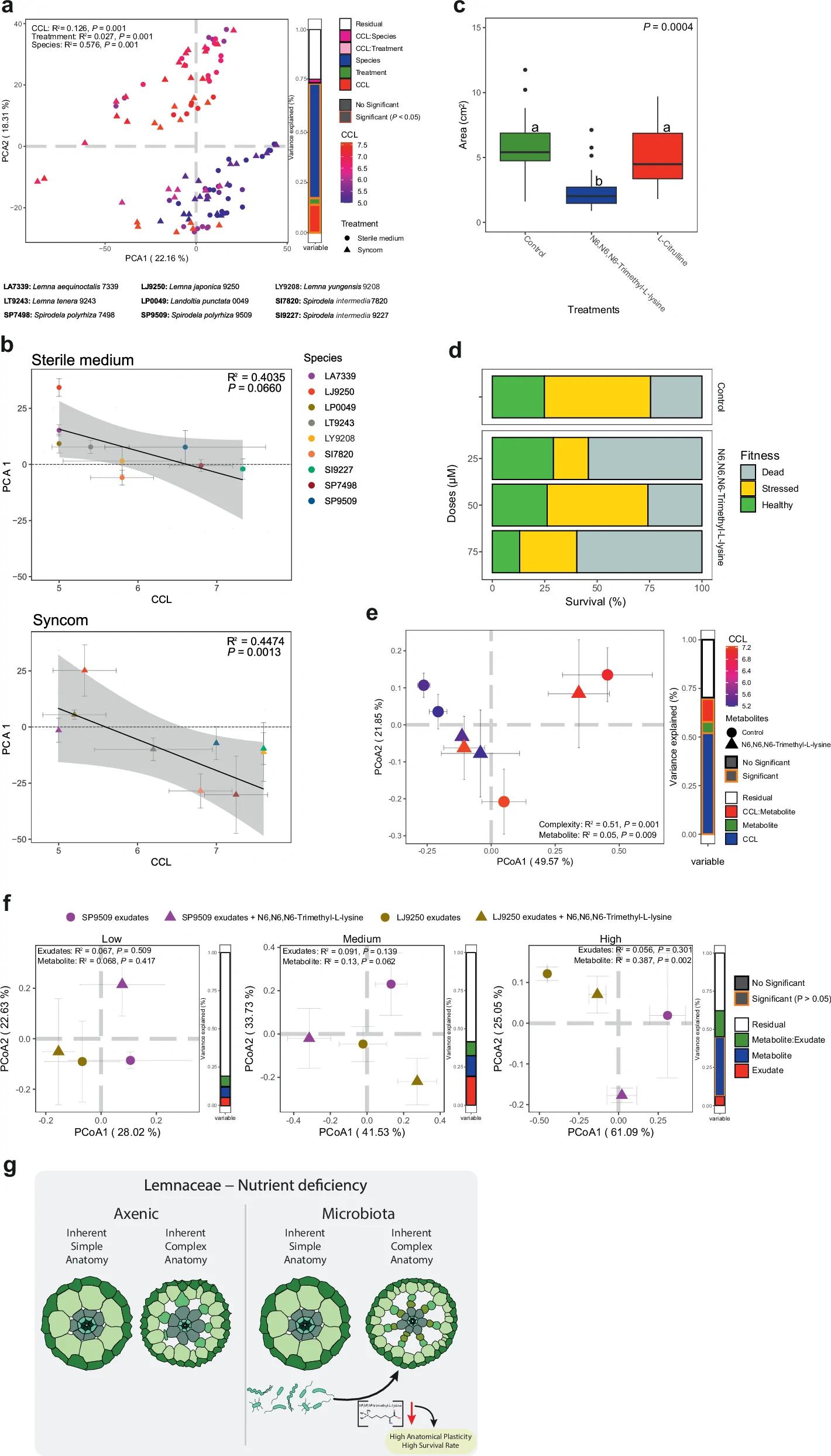
The modulatory metabolite N^6^,N^6^,N^6^-Trimethyl-L-lysine modifies the composition of the root microbiota community, impacting the anatomical plasticity of the root. **a** Principal Component Analysis (PCA) showing the projection of root metabolic composition profiles of nine *Lemnaceae* species grown in 1/1000 SH medium or in the same medium supplemented with the full bacterial synthetic community. Samples are coloured according to the number of root cell layers (CCLs) present in the plant species from which they were collected. The bar on the right denotes the percentage of variance explained by the experimental variables using a Permutational Multivariate Analysis of Variance (PERMANOVA, *n* = 6). **b** Linear regression analysis between root metabolite composition (shown in panel **a**), expressed as principal component 1 (PC1), in axenic plants (sterile medium) or in plants inoculated with the full bacterial synthetic community (Syncom), and the number of root cell layers (CCLs) across the *Lemnaceae* species used. The proportion of variation in metabolic complexity explained by the number of root cell layers (R²), together with the associated *p* values, is shown in the panel. Error bars represent standard deviations (SD; *n* = 6). **c** The graph shows the average total frond area of plants (LJ9250: *Lemna japonica* 9250; LP0049: *Landoltia punctata* 0049; LP8539: *Lemna perpusilla* 8539; LY9208: *Lemna yungensis* 9208; SP7498: *Spirodela polyrhiza* 7498; SP9509: *Spirodela polyrhiza* 9509) grown for 15 days in 1/1000 SH medium inoculated with the full bacterial synthetic community alone (control) or supplemented with N^6^,N^6^,N^6^-Trimethyl-L-lysine or L-Citrulline. Box plots show the median (centre line), the first and third quartiles (box boundaries), and whiskers extending to the most extreme data points within 1.5× the interquartile range. Significant differences among treatments were determined using a one-way ANOVA followed by Tukey’s post-hoc tests (*p* < 0.05, *n* = 6). Different lowercase letters indicate significant differences among treatments. **d** Stacked bar plot showing the percentage of dead (grey), stressed (yellow), and healthy (green) plants quantified for *Spirodela polyrhiza* 9509, a species with high inherent root anatomical complexity, *n* = 6. Plants were grown in sterile 1/1000 SH medium, with or without supplementation with N^6^,N^6^,N^6^-Trimethyl-L-lysine at different concentrations (25, 50, and 75 μM). **e** Principal coordinates analysis (PCoA) showing the projection of bacterial community composition profiles in the roots of four *Lemnaceae* species (SP9509: *Spirodela polyrhiza* 9509, SP7498: *Spirodela polyrhiza* 7498, LP0049: *Landoltia punctata* 0049, LJ9250: *Lemna japonica* 9250) with medium and high inherent root complexities. Plants were grown in 1/1000 SH medium inoculated with the full bacterial synthetic community and either exposed to 50 μM N^6^,N^6^,N^6^-Trimethyl-L-lysine or left untreated (control). Each point in the scatter plot represents a plant species and is coloured according to the number of root cell layers CCLs present in its roots. The error bars represent standard deviations SD. The bar on the right denotes the percentage of the variance explained by the experimental variables using a Permutational Multivariate Analysis of Variance (PERMANOVA, *n* = 6). **f** Principal coordinates analysis PCoA showing the projection of bacterial community composition profiles in “synthetic roots” of different complexities (low, medium, and high) supplemented with root exudates from two *Lemnaceae* species, *Spirodela polyrhiza* 9509 and *Lemna japonica* 9250, and inoculated with the full bacterial synthetic community (control) or additionally treated with 50 μM N^6^,N^6^,N^6^-Trimethyl-L-lysine. Each point in the scatter plots represents synthetic roots exposed to a given treatment and corresponds to the average distribution of values along both axes. Error bars represent the standard deviation (SD, *n* = 5). *P-values* within each panel (**e**, **f**) were obtained from a PERMANOVA model and correspond to the variables metabolites and root complexity (CCLs) for real roots and metabolites and exudates for “synthetic roots”. **g** Proposed model of microbiota-driven anatomical plasticity affecting plant survival in nutrient-depleted environments.

To uncover these modulatory metabolites, we analysed the roots of nine *Lemnaceae* species with different inherent root anatomical complexity, grown either axenically or with the full bacterial synthetic community under low nutrient conditions (1/1000 SH) (Supplementary Fig. 22a). We found significant differences in metabolic composition between anatomically relatively complex roots and simple roots across all species and microbial conditions analysed (Fig. 4a). Furthermore, we observed that the metabolic complexity of the roots significantly and negatively correlated (R^2^ = 0.4474, *p* = 0.0013) with the number of root cell layers, an indicator of root anatomical complexity, only in plants colonised by microbes (Fig. 4b). To identify metabolites that modulate microbiota-driven root anatomical plasticity, we first selected metabolite features that differed significantly between axenic plants and plants inoculated with the bacterial synthetic community (Supplementary Fig. 22b, c), identifying 341 putatively annotated metabolites (Supplementary Fig. 22c and Supplementary Data 6). From this set, we further narrowed this selection to 15 putative metabolites whose concentrations were significantly negatively correlated with root anatomical complexity across the *Lemnaceae* species studied (Supplementary Fig. 22d, e, and Supplementary Data 7). Among these 15 candidates, only two metabolites could be identified with confidence level ID2, whereas the others remain putatively annotated based on spectral features. This negative correlation suggests that these compounds might differentially influence root plasticity depending on the root’s inherent anatomical complexity. High levels of these compounds might limit microbiota-driven plasticity in roots that are inherently more complex and exhibit lower concentrations of these metabolites.

We evaluated the effects of the two putative metabolites (L-citrulline and N^6^,N^6^,N^6^-Trimethyl-L-lysine), whose identities were assigned with confidence level ID2 (Supplementary Data 7) and which were commercially available, on the growth kinetic of six *Lemnaceae* species, with different inherent root complexities, inoculated with the bacterial synthetic community under low-nutrient conditions (1/1000 SH) (Fig. 4c and Supplementary Fig. 23a). We observed that the exogenous addition of N^6^,N^6^,N^6^-Trimethyl-L-lysine significantly reduced the total area of plant fronds (Fig. 4c and Supplementary Fig. 23a). In contrast, we did not observe a significant effect of L-citrulline on the growth of the plant species analysed (Fig. 4c and Supplementary Fig. 23a) and therefore focused exclusively on the characterisation of N^6^,N^6^,N^6^-trimethyl-L-lysine.

First, we confirmed the N^6^,N^6^,N^6^-trimethyl-L-lysine identity with a confidence level ID1 using a reference standard^36^ (Supplementary Fig. 23b). Then, we exposed *Spirodela polyrhiza* 9509, with an inherent complex root, to increasing concentrations of N^6^,N^6^,N^6^-Trimethyl-L-lysine in the presence of the bacterial synthetic community under low nutrient conditions. As expected, increasing levels of N^6^,N^6^,N^6^-Trimethyl-L-lysine reduced plant survival under these conditions (Fig. 4d). Furthermore, a similar pattern of changes was observed in root anatomy in plants treated with N^6^,N^6^,N^6^-Trimethyl-L-lysine. At low concentrations of this compound (25 μM), quantification of root anatomical features yielded results comparable to those observed in untreated plants colonised by the synthetic bacterial community (Supplementary Fig. 23c, d). However, roots treated with higher doses of N^6^,N^6^,N^6^-Trimethyl-L-lysine (50 and 75 μM) exhibited reduced anatomical plasticity in response to the microbial colonisation, and their anatomical features were in general similar to those of untreated plants grown under axenic conditions (Supplementary Fig. 23c, d). These conclusions were more evident when we correlated individual root anatomical features measured in plants treated with different concentrations of N^6^,N^6^,N^6^-Trimethyl-L-lysine with those obtained from axenic roots or from roots inoculated with the bacterial synthetic community. We found that roots treated with a low concentration of N^6^,N^6^,N^6^-Trimethyl-L-lysine (25 μM) exhibited the highest number of anatomical features that correlated with those observed in roots inoculated with the bacterial synthetic community treatment (Supplementary Fig. 23e). In contrast, higher doses of this compound (50 and 75 μM) compromised root plasticity in response to the bacterial synthetic community colonisation, resulting in plants whose key anatomical traits showed reduced correlation with those of microbiota-inoculated roots and increased similarity to those measured in axenic plants (Supplementary Fig. 23e).

We also investigated the effect of supplementing with 50 μM N^6^,N^6^,N^6^-Trimethyl-L-lysine on plant microbial community structure in four *Lemnaceae* species with medium to high inherent anatomical root complexity (*Lemna japonica* 9250, *Landoltia punctata* 0049, *Spirodela polyrhiza* 7498, and *Spirodela polyrhiza* 9509), all inoculated with the full bacterial synthetic community. In line with its effect on anatomical plasticity (Supplementary Fig. 23c–e), we observed that beta-diversity was explained by the variables “number of root cell layers (CCL)”, a proxy for root complexity, “metabolite”, and their interaction (Fig. 4e and Supplementary Fig. 24a). These results indicate that external supplementation of this compound in plants with inherently relatively complex anatomical roots, where it is normally present at lower concentrations, could increase endogenous levels and alter root microbial composition, thereby impacting the anatomical plasticity of the root. Consistent with these findings, we observed that N^6^,N^6^,N^6^-Trimethyl-L-lysine modulates microbial community assembly only in “synthetic roots” with high anatomical complexity (R^2^ = 0.387, *p* = 0.002) that were inoculated with the bacterial synthetic community and exposed to two distinct root exudates extracted from *Spirodela polyrhiza* 9509 and *Lemna japonica* 9250 (Fig. 4f and Supplementary Fig. 24b). No significant metabolite effect on microbial profiles was detected in “synthetic roots” with low or medium anatomical complexities under the same conditions (Fig. 4f and Supplementary Fig. 24b). In this system, which lacks a plant immune component, changes in microbial assembly induced by N^6^,N^6^,N^6^-Trimethyl-L-lysine can be attributed either to its direct effects on bacterial growth or to emergent properties arising from interactions between root anatomical/metabolic complexity and metabolite concentration. Indeed, it promoted the growth of certain individual members of the full bacterial synthetic community in vitro (Supplementary Fig. 24c). Overall, these results indicate that N^6^,N^6^,N^6^-Trimethyl-L-lysine mediates root anatomical plasticity responses associated with microbial colonisation.

Although the mechanism of action of the modulatory compound N^6^,N^6^,N^6^-Trimethyl-L-lysine in roots is not fully characterised here, we speculate that, in addition to its possible direct effects on root anatomy and bacterial growth, its differential local accumulation across anatomically diverse root microhabitats influences the spatial distribution of microbiota members, consistent with previous publications^37^. This effect might modulate microbial growth by serving as a carbon source, thereby shaping microbial activity across root microhabitats and ultimately affecting root plasticity and plant stress tolerance.

## Discussion

Habitat characteristics largely determine the ability of microbial communities to colonise plant roots^9,38^. Our results, using a collection of plants with different inherent root anatomy, demonstrate that the anatomical complexity of the microhabitats present in the roots controls the establishment of specific microbiota. This differential microbial colonisation induces changes in anatomical plasticity, necessary for plant survival under nutrient-limited conditions, which under our experimental conditions only occurs in more anatomically complex roots. Our experiments with synthetic communities further confirmed these observations and led to the discovery that root anatomical plasticity can be controlled by the microbiota. This mechanism requires a certain level of inherent anatomical complexity and, eventually, a preserved root capacity to interact with the microbiota. The progressive reduction in root anatomical complexity, linked to the loss of nutrient uptake capabilities observed in some roots in our collection^27^, might impair the ability of roots to interact effectively with the microbiota.

We demonstrate, using synthetic and natural roots, that microbiota-driven root anatomical plasticity integrates the anatomical and metabolic features of root microhabitats. We identify putative metabolites differentially accumulated in roots with distinct inherent anatomical complexity. One metabolite, N^6^,N^6^,N^6^-Trimethyl-L-lysine, has a modulatory effect on microbial colonisation and root anatomical plasticity important for plant tolerance to low nutrient conditions (Fig. 4g).

N^6^,N^6^,N^6^-Trimethyl-L-lysine is a precursor of carnitine, a molecule that plays a central role in plant tolerance to stress. For example, carnitine functions as antioxidant, directly protecting plant cells from the damaging effects of reactive oxygen species generated in response to various abiotic stresses^39^. Furthermore, carnitine acts as an effective osmoprotectant, helping plants maintain DNA integrity under osmotic stress conditions^40^. Exogenous application of carnitine has been shown to promote plant growth and stimulate mitosis under salt stress^40^.

Indirectly, the synthesis of N^6^,N^6^,N^6^-Trimethyl-L-lysine can influence the cell’s methylation metabolism. By affecting methyl group availability, changes in the level of N^6^,N^6^,N^6^-Trimethyl-L-lysine could indirectly impact DNA and histone methylation, potentially altering the expression of genes related to development and stress responses^41^. It is also known that active DNA demethylation regulates myo-inositol secretion by roots, with consequences for beneficial interactions between the plant and a member of the root microbiota^42^. Similarly, dysfunction of the histone demethylase IBM1 alters root microbiota composition in *Arabidopsis thaliana*^43^.

Therefore, we cannot exclude the possibility that local changes in N^6^,N^6^,N^6^-Trimethyl-L-lysine levels, resulting from its interaction with the microbiota across different *Lemnaceae* species, may affect the pool of available carnitine in the plant. Nor can we exclude that the indirect effect of changes in N^6^,N^6^,N^6^-Trimethyl-L-lysine concentration could impact epigenetic mechanisms controlling plant-microbiota interactions. Both scenarios might have consequences for plant abiotic stress tolerance and root plasticity.

Our in vitro results on the effect of N^6^,N^6^,N^6^-Trimethyl-L-lysine on the growth of microbiota members, as well as its impact on the microbial composition of real and artificial roots with different anatomical and metabolic complexities, support the notion that emergent properties arising from the interaction between anatomical and metabolic features of microhabitats might regulate the establishment of beneficial microbes. These findings are consistent with a recent publication demonstrating that the differential local accumulation of glutamine along the root cylinder influences the spatial distribution of microbiota members capable of using it as a carbon source^37^. It is therefore plausible that the local accumulation of N^6^,N^6^,N^6^-Trimethyl-L-lysine across anatomically distinct root microhabitats, including the rhizosphere, might similarly influence the spatial distribution of beneficial microbiota members by serving as a carbon source or signalling molecule, as N^6^,N^6^,N^6^-Trimethyl-L-lysine has also been detected as a component of the root exudate core metabolome^44^.

Taken together, our work contributes to further explaining the functional feedback between anatomical and metabolic features of root microhabitats and root microbial colonisation previously suggested^9,12,45,46^ and generalise the previously found effect of microbiota on root anatomical plasticity^9^. This dynamic interplay between root microhabitat configurations and the microbial communities inhabiting them determines plant survival under restricted nutritional conditions.

Although a deeper understanding of the genetic components underlying the mechanism described here is required before its application, we envision that this knowledge could contribute to optimising root microbial function to enhance root plasticity and plant stress tolerance. Plants can be genetically engineered to optimise the production of modulatory metabolites that positively influence the recruitment of beneficial microbes^47,48^. Alternatively, using synthetic biology strategies, we can precisely control the exudation of key metabolites that microbes use as carbon sources, thereby affecting microbial recruitment and localization^49–51^. These strategies could be used to effectively regulate the concentration of N^6^,N^6^,N^6^-Trimethyl-L-lysine in roots according to their inherent anatomical complexity, maximising beneficial microbe-driven root plasticity. Beyond optimising modulatory metabolite concentrations, synthetic biology approaches hold potential to specifically enhance aspects of root anatomy without negatively impacting root function, including its interactions with the microbiota^52,53^. Combined with the direct application of N^6^,N^6^,N^6^-Trimethyl-L-lysine, these strategies could modulate root interactions with beneficial microbes. Together, these advances, along with our growing understanding, should stimulate the development of new microbiota-based strategies with a positive impact on agricultural practices.

## Methods

### Lemnaceae collection

All *Lemnaceae* species used in this work are from the Landolt collection (ETH Zurich; http://www.duckweed.ch), except *Spirodela polyrhiza* 9509, which was donated by Prof Klaus Appenroth from Friedrich Schiller University Jena, Germany. *Pistia stratiotes* was obtained from JAM Aquatics, Wrexham, United Kingdom (Supplementary Data 8).

### Natural water collection

For experiments analysing the effect of the microbiota on *Lemnaceae* root anatomy, natural water was collected from a lake located in the Attenborough Nature Reserve, Nottinghamshire, UK (52° 54′ 3.747′′ N, 1° 14′ 0.744′′ W). Water was collected from an area of the lake supporting a natural population of *Lemnaceae* using autoclaved 1-L glass bottles. During water collection, all personnel wore gloves. The water was filtered in a laminar flow using paper filters to remove plant debris and large particulate matter. Filtered water was stored at 4 °C until use.

### Plant growth conditions

The natural water was divided into two portions: one portion was autoclaved twice, with a one-day interval between sterilisation cycles, to eliminate the water microbiota, while the other portion was kept in the dark at 4 °C to preserve the microbiota. To assess sterility, 100 µL of the autoclaved water was placed onto LB and YPD agar plates and incubated at 28 °C for two weeks.

Prior to the experiment, all plants were surface sterilized with 10% bleach, washed with 2% sodium thiosulfate to neutralize residual bleach, and rinsed at least three times with sterile water. Plants were then cultured in flasks containing ½-strength Schenk and Hildebrandt (SH) medium (Supplementary Data 9).

For experimental setup, 100 mL of either intact natural water or autoclaved water (control) was added to sterile 250-mL flasks. Three plants of each *Lemnaceae* species were then introduced into the corresponding flasks. In the case of *Pistia stratiotes*, each flask was inoculated with a single juvenile plant exhibiting three visibly emerging leaves. Five biological replicates (five individual flasks) containing either intact natural water or autoclaved water were used for each treatment. Flasks were manually randomised and placed in a Conviron growth chamber. To minimize positional effects within the growth chamber, flasks were re-randomised every 48 h throughout the experiment. Plants were grown at 22 °C, 70% relative humidity, a light intensity of 120 μmol m^−2^ s^−1^, and a 16 h dark/8 h light photoperiod for 21 days.

### Root samples preparation for anatomical studies

To determine differences in root anatomical traits between plants grown with or without a natural microbiota, five plants per species and treatment were harvested. Plants were removed from the flasks and gently dried on filter paper before the roots were immersed in a cylindrical vessel containing molten 4.5% agarose. The vessels were then placed in cold water to accelerate agarose cooling and solidification. After solidification, agarose blocks were carefully trimmed with a razor blade to obtain a small block containing a vertically oriented root. Blocks were mounted in a vibratome, and 15–20 root cross-sections were obtained from each root sample. Cross-sections were generated using a blade speed of 1 mm/s, a section thickness of 100 μm, and a blade oscillation frequency of 65 Hz. Root sections were stained with a 0.3% (w/v) solution of fluorescent brightener 28 (calcofluor) and subsequently rinsed in distilled water for 1 min.

Each section was imaged using a Leica SP5 confocal microscope equipped with a 40x objective (NA = 0.8), with excitation at 405 nm and emission collected in the 620–630 nm band-path range. Confocal images were analysed using ImageJ (Polar Transform plugin)^54^, and root anatomical features were quantified.

Root tissue layers were identified, and 21 root traits were measured: the total number of root cell layers (CCL); endodermis diameter (endodermis diameter); stele diameter (stele diameter), diameter of cortical cell layer 1-4 (CCL1- CCL4 diameter); diameters of external cortical cell layer 1 and 2 (CCLe1 and CCLe2 diameter); total root diameter (Root diameter); the number of cells in each root tissue layers (endodermis N cell, stele N cell, CCL1-CCL4 N cell, and CCLe1-CCLe2 N cell), the total number of aerenchyma-like structures (Aerenchyma-like N); the mean area of aerenchyma-like structures (Aerenchyma-like mean area); and the number of roots per plant (Root N).

### Plant imaging and growth parameters quantification

Plants were photographed every 48 h for 21 days. Images were captured from the bottom of the flasks using a transparent elevated platform with a water bath to minimise optical distortion, with an EOS 4000D DSLR camera (Canon) equipped with an 18-55 mm lens operating in automatic mode. Image analysis was performed using ImageJ^54^ to quantify total frond area, number of roots, and the number of healthy, stressed, and dead plants in each sample. Plants were classified as healthy, stressed, or dead based on a predominance of green, yellow, or white pixels in their images, respectively.

### DNA extraction for 16S rRNA gene analysis

For DNA extraction, roots and fronds from all plants were separated using scissors previously sterilised with 70% ethanol. The roots and fronds were collected separately and placed in 1.5 mL Eppendorf tubes containing sterile water. Samples were rinsed at least three times with sterile water to remove weakly associated microbes and then stored at −80 °C until DNA extraction.

Additionally, 1 mL of water was collected from each flask. The water samples were centrifuged at maximum speed for 20 min in an Eppendorf 5810 R centrifuge at room temperature. The supernatant was discarded, and the pellets were resuspended in 1 mL of sterile water, followed by a second centrifugation under the same conditions. All water pellets were stored at −80 °C until further use.

Before DNA extraction, root and frond samples were lyophilised using an Alpha 2-4 LD freeze-dry system and then pulverised in a TissueLyser II (Qiagen) with two cycles of 30 s at a frequency of 30 s^−1^. DNA from roots, fronds, and water samples was extracted using a 96-well-format MoBio PowerSoil Kit (MOBIO Laboratories; Qiagen) according to the manufacturer’s instructions. Prior to extraction, all tubes containing the samples, including those containing water, were randomised by placing them in a plastic bag and shaking thoroughly. Tubes with the samples were then removed individually from the bag and placed into the DNA extraction plates. This randomization was maintained throughout library preparation and sequencing.

### 16S rRNA gene library preparation and sequencing

For natural water experiments, *16S rRNA* gene sequencing was performed by amplifying the V3-V4 region of the bacterial *16S rRNA* gene using the primers 338 F (5′-ACTCCTACGGGAGGCAGCA-3′) and 806 R (5′- GGACTACHVGGGTWTCTAAT-3′)^55^. Two barcodes and six frameshifts were added to the 5’ end of the 338 F primer, and six frameshifts were added to the 5’ end of the 806 R primers, following the protocol of Lundberg et al.^28^. PCR reactions were performed in triplicate using ~20 ng of DNA template and included a unique mixture of three frameshifted primer combinations for each plate. The PCR blockers mPNA and pPNA were used to reduce amplification of plant host plastid and mitochondrial 16S *rRNA* gene sequences. PCR conditions consisted of 5 μL 1x Kapa Enhancer, 5 μL 10x Kapa Buffer A, 1.25 μL 5 μM 338 F primer, 1.25 μL 5 μM 806 R primer, 0.375 μL mixed plant rRNA gene-blocking peptide nucleic acids (PNAs; 1:1 mix of 100 μM plastid PNA and 100 μM mitochondrial PNA), 0.5 μL 10 mM Kapa dNTPs, 0.2 μL 5 U/μL Kapa Robust Taq, 8 μL nuclease-free dH2O, and 5 μL DNA template. Thermal cycling conditions were as follows: initial denaturation at 95 °C for 60 s; 24 cycles of 95 °C for 15 s, 78 °C for 10 s (PNA annealing), 50 °C for 30 s, and 72 °C for 30 s; followed by a hold at 12 °C until further processing.

PCR reactions were cleaned using AMPure XP magnetic beads (Beckman Coulter) to remove primer dimers. The PCR products were indexed using a forward primer containing the Illumina adaptor (5′AATGATACGGCGACCACCGAGATCTACACGCCTCCCTCGC GCCATCAGAGATGTG-3′) and 96 indexed reverse primers containing the reverse Illumina adaptor(5′CAAGCAGAAGACGGCATACGAGATXXXXXXXXXGTGACTGGAGTTCAGACGTGTGCTC3′). PCR amplification was performed using Kapa HiFi Hotstart Readymix with the following thermal cycling conditions: 95 °C for 60 s; 9 cycles of 95 °C for 15 s, 78 °C for 10 s (PNA annealing); 60 °C for 30 s, and 72 °C for 35 s; followed by a hold at 12 °C until use. PCR products were purified using AMPure XP magnetic beads (Beckman Coulter) and quantified using a Qubit 2.0 fluorometer (Invitrogen). Amplicons were pooled in equimolar amounts and diluted to 10 pM for sequencing. Sequencing was performed on an Illumina MiSeq platform using a 600-cycle v3 chemistry kit at the DeepSeq facility, University of Nottingham.

### 16S rRNA gene amplicon sequence data processing

Paired-end sequences were merged, quality filtered and demultiplexed according to their barcodes. The resulting sequences were denoised and collapsed into amplicon sequence variants (ASVs) using DADA2 (v.1.10.1)^56^. Representative ASV sequences were taxonomically classified using the Mothur naïve Bayes classifier trained on the SILVA SSU 138 database. ASVs assigned to chloroplast, mitochondria, oomycetes, archaea, or lacking a kingdom-level assignment were removed. Finally, using the selected ASVs, we created raw count, rarefied (500 reads per sample), and relative abundance tables. These relative abundance tables were processed and analysed as a phyloseq object using the phyloseq package (v1.46.0)^57^ and visualised using the ggplot2 (v3.5.1)^58^ in RStudio (Build 446) with R^59^.

### Computational and statistical analysis of experiments using natural water

The phylogenetic tree of the *Lemnaceae* collection used in this study was constructed based on variations in chloroplast *rpl16* gene sequences, which encode the L16 ribosomal protein, a component of the large (50S) ribosomal subunit in the chloroplasts of all plant used^60^. The sequences were aligned using the AlignSeqs function from the DECIPHER (v2.30.0) package^61^. The tree was visualised using ggplot2 (v3.5.1)^58^.

To generate the map of the United Kingdom showing the sample collection locations, we used ggplot2 (v3.5.1)^58^ and geographr (v1.0.1)^62^ packages in RStudio. The GPS coordinates for the water sample collection sites shown on the map were the Arboretum, School of Biosciences, Sutton-Bonington Campus, University of Nottingham (52.834139, −1.253199); Peak District National Park (53.365604, −1.697046); and Attenborough Nature Reserve (52.904268, −1.233926), Nottingham, UK.

For the analysis of root anatomical traits, we constructed a matrix (sample x root traits) in which each cell contained the calculated value of a given trait for each sample. Each root trait was then standardised across samples using a *z*-score transformation. To compare root trait profiles between sterile and natural water samples, or between sterile SH medium and SH medium inoculated with the bacterial synthetic community samples, we performed a principal coordinate analysis (PCoA) based on Euclidean distances between samples, using the *z*-score-transformed matrix as input. The PCoA was plotted using the ggplot2 package (v3.5.1)^58^.

In addition, we generated a heatmap using the pheatmap function from the pheatmap v1.0.12 package^63^. In the heatmap, both columns (samples) and rows (root traits) were clustered using the hclust function from the R stats package v4.3.0^59^.

We compared the values of each root trait in *Lemnaceae* and Pistia species grown in natural water with those grown in sterile water using the *t* test function from the R stats package (v4.3.0)^59^. A similar statistical analysis was used to determine differences in root traits between plants grown in sterile media and plants grown in the presence of the bacterial synthetic community supplemented with different nutrient concentrations.

Treatment-specific enrichment profiles for natural water samples relative to sterile water samples were calculated using the DESeq2 package (v1.24.0)^64^. We visualized the enrichment patterns (log2 fold change) of the primary root traits using a heatmap generated with the ggplot2 (v3.5.1) package^58^ in R. In the heatmap, both columns (*Lemnaceae* species) and rows (root traits) were clustered using the hclust function from the R stats package (v4.3.0)^59^.

To visualise differences in anatomical features, such as the number of root cell layers (CCL) and the diameter of each layer, across plant species, we used rose diagrams for plant roots grown in sterile and natural water. The diagrams were generated using the coord_polar function from the ggplot2 package (v3.5.1)^58^ in RStudio. Similar rose diagrams were used to visualised differences in anatomical features across the six *Lemnaceae* species grown in SH medium with different nutrient levels, with or without inoculation with the bacterial synthetic community.

We determined pairwise correlations among all root anatomical traits measured in plants grown in natural and sterile water using Pearson’s correlation analysis implemented via the corr function from the ggcorrplot package (v0.1.4.1)^65^. To visualise the correlations, we constructed a root trait x root trait matrix, in which each cell contained the corresponding Pearson correlation coefficient for each pair of traits. The correlation plot was generated using the ggplot2 package (v3.5.1)^58^, and hierarchical clustering of the traits was performed using the hclust function from the R stats package (v4.3.0)^59^.

The correlation between the number of root cell layers (CCL) and plant mortality in plants grown in sterile and natural water was assessed using linear models implemented with the lm function from the R stats package (v4.3.0)^59^. The results were visualised using the ggplot2 package (v3.5.1)^58^. The same approach was used to assess correlations between CCL and DAPI-stained “synthetic roots” supplemented with *Lemnaceae* exudates, between CCL and the metabolites PCoA1 axis, and between CCL and the thermal peak value for each Lemnaceae species across the two treatments (axenic roots and roots colonised by the bacterial synthetic community)

To quantify plant growth, we measured the total frond area in each flask at multiple time points, and all values were normalised to day 0. Differences in total frond area across plant species and time points in plants grown in natural and sterile water were assessed using the *t* test function from the R stats package (v4.3.0)^59^. Individual growth curves were visualised using the ggplot2 package (v3.5.1) in R^58^. Growth rates during the linear phase of the curves were calculated as the slope of each curve using the slope function from the pkr package (v0.1.3)^66^, and the maximum value of each curve was determined using the max function from the R stats package (v4.3.0)^59^. To compare growth parameters among *Lemnaceae* species, heatmaps were generated using the ggplot2 package (v3.5.1) in R^58^. A similar approach was used to quantify growth differences among the six *Lemnaceae* species grown in SH medium at different nutrient levels, with or without inoculation with the bacterial synthetic community.

In all cases, for the *16S rRNA* gene sequencing analysis, the abundance tables obtained from the bioinformatic processing were imported and analysed as a phyloseq object using the phyloseq package (v1.46.0)^57^ and visualised with the ggplot2 (v3.5.1)^58^ in R^59^.

To assess alpha diversity across water treatments, species, and compartments, the Shannon index was calculated using the estimate_richness function from the phyloseq package^57^. Differences between treatments were evaluated using one-way ANOVA followed by a Tukey’s post-hoc test. Additionally, linear regression was performed to examine the relationship between alpha-diversity and plant mortality percentage using the lm function from the stats package (v4.3.0)^59^. A similar approach was used to assess alpha diversity in the six *Lemnaceae* species grown in SH medium at different nutrient levels, with or without inoculation with the bacterial synthetic community, and in the “synthetic roots” with different complexities and plant exudates. In this case, an ANOVA followed by a Tukey’s post-hoc test was used to evaluate significant differences among treatments.

For beta diversity, principal coordinate analysis (PCoA) was performed using Bray-Curtis dissimilarity matrices derived from the ASV abundance table. We used the following design to determine beta diversity across compartments:

$${{{\rm{Bray}}}}-{{{\rm{Curtis\; Dissimilarity}}}} \sim {{{\rm{Compartment}}}}$$

and this one to determine beta diversity across species in each compartment:

$${{{\rm{Bray}}}}-{{{\rm{Curtis\; Dissimilarity}}}} \sim {{{\rm{Species}}}}$$

We performed a permutational multivariate analysis of variance (PERMANOVA) using the adonis2 function from the vegan package (v.2.6-4) in R^67^ to assess the variance explained by species and the number of root cell layers (CCL).

Pairwise correlations between microbiota composition (PCoA1 axis) and anatomical root traits (PCoA1 axis) were assessed using a Mantel test implemented via the mantel function from the vegan package (v.2.6-4)^67^. The resulting correlations were visualised using the ggplot2 package (v3.5.1) in R^58^.

We used the DESeq2 package (v1.24.0)^64^ to calculate compartment-specific bacterial taxa enrichment profiles for frond, water, and root samples separately. The abundance differences of each taxonomic unit were assessed across six taxonomic levels, Phylum, Class, Order, Family, Genus, and ASV, comparing root and frond compartments to water for the 19 plant species studied. These differences were modelled using a generalized linear model (GLM) with the following formula:

$${{{\rm{Abundance}}}} \sim {{{\rm{Compartment}}}}+{{{\rm{Rep}}}}$$

Enrichment patterns (log2 fold change) of the main taxonomic units at each taxonomic level were visualised in heatmaps, separated by compartment (frond and root), using the ggplot2 package (v3.5.1)^58^.

Unique ASVs specific to a single compartment (water, frond, or root) were identified using the ps_venn function from the MicroEco package (v1.13.0)^68^. A bar plot illustrating the number of unique ASVs in each compartment was generated using the ggplot2 R package (v3.5.1)^58^.

### Bacterial culture collection

To isolate representative bacterial strains from the *Lemnaceae* microbiota, we grew two *Lemnaceae* species, *Spirodela polyrhiza* and *Wolffia australiana*, in two natural water samples collected from the arboretum at the Sutton-Bonington campus, University of Nottingham (52.834139, −1.253199) and from the Peak District National Park, UK (53.365604, −1.697046). *Lemnaceae* plants were collected, and roots and fronds were separated using tweezers and washed twice with 1x PBS (roots) or 10 mM MgCl_2_ (fronds)^69,70^. Water samples were also collected. The roots and fronds were pulverised using sterile mortars and pestles with 5 mL of 10 mM MgCl_2_ under laminar flow conditions. The resulting homogenates were diluted 1:200,000 in 10 mM MgCl_2_, and 100 μL of each dilution was plated onto ten different growth media with distinct nutritional characteristics (Supplementary Data 1). Plates were incubated at 28 °C for two to three weeks. Bacterial colonies displaying distinct morphologies and colours were sequentially re-streaked onto LB agar plates until pure cultures were obtained. In total, 245 bacterial isolates were recovered. For each isolate, a glycerol stock (1:1 v/v 80% glycerol) was prepared and stored at −80 °C.

### Characterisation of the bacterial strains using Sanger sequencing

To assign taxonomy to the bacterial cultures, we PCR-amplified and Sanger sequenced the *16S rRNA* gene from each individual bacterial strain. Total bacterial genomic DNA was isolated following the protocol described in Bai et al.^70,71^. Briefly, 6 µL of each bacterial culture was transferred to a 96-well plate containing 10 µL of buffer I (25 mM NaOH, 0.2 mM EDTA, pH 12). The cultures were lysed at 95 °C for 30 min, after which 10 µL of buffer II (40 mM Tris-HCl, pH 7.5) was added to neutralise the reaction. The V1-V9 region of the bacterial *16S rRNA* gene was amplified using primers 27 F (5′- AGRGTTTGATCMTGGCTCAG-3′) and 1492 R (5′- ACCTTGTTACGACTT-3′), with genomic DNA as the template. PCR reactions contained 12.5 µL of 1x Kapa HiFi Hotstart Readymix, 0.5 µL of 5 μM primer 27 F, and 0.5 µL of 5 μM primer 1492 R, resulting in a final volume of 50 µL. The PCR cycling conditions were as follows: initial denaturation at 95 °C for 5 min, 34 cycles of denaturation at 95 °C for 30 s, annealing at 55 °C for 30 s, and extension at 72 °C for 1 min, followed by a final extension at 72 °C for 5 min. PCR products were purified using the QIAquick Gel Extraction Kit according to the manufacturer’s instructions and were Sanger sequenced using the 27 F primer. The bacterial collection isolates are listed in Supplementary Data 2.

### Bacterial synthetic community design and preparation

The bacterial synthetic community used in this study was composed of 190 individual bacterial strains selected from the bacterial culture collection (Supplementary Data 2). Each strain was inoculated into an individual well in a 3-mL 96-well plate containing 1 mL of LB medium and incubated at 28 °C with agitation at 120 r.p.m. for 5 days. To include slow-growing strains, 100 μL of each culture was transferred to a new 96-well plate containing 1 mL of fresh LB medium and incubated under the same conditions for an additional 5 days. Cultures from both plates, containing fast- and slow-growing strains, were pooled and centrifuged at 3220 g for 8 min using a bench centrifuge (Eppendorf 5810 R). Bacterial pellets were rinsed three times with 10 mM MgCl_2_ to remove spent medium and cellular debris. The cells were then centrifuged under the same conditions, resuspended in 1 mL of 10 mM MgCl_2,_ and the optical density of each culture was determined at 600 nm. To equalise bacterial concentrations, we assumed that 1 OD_600nm_ unit corresponds to 1 × 10^9 ^c.f.u./mL, and a volume equivalent to 1 × 10^5^ c.f.u./mL from each individual strain was combined in a 15-mL Falcon tube to assemble the bacterial synthetic community. The final OD_600nm_ was adjusted to 0.08 before adding the bacterial synthetic communities to the medium.

### Experiment to demonstrate the influence of root complexity and nutrient availability on microbiota assembly

For this experiment, we selected six *Lemnaceae* species differing in the number of root cell layers (CCL): *Spirodela polyrhiza* 9509 (high number of CCL, 7 layers), *Spirodela Intermedia* 7820 (high number of CCL, 7 layers), *Landoltia punctata* 0049 (intermediate number of CCL, 6 layers), *Lemna japonica* 9250 (intermediate number of CCL, 6 layers), *Lemna aequinoctalis* 7339 (low number of CCL, 5 layers), and *Lemna perpusilla* 8539 (low number of CCL, 5 layers). Plants were grown in flasks containing 100 mL of Schenk-Hildebrandt (SH) medium. The experiment was conducted under three nutrient conditions, high (1/2 SH), medium (1/100 SH), and low (1/1000 SH), with or without inoculation with 100 µL of the bacterial synthetic community prepared as described above. Four biological replicates were used per treatment. At the start of the experiment, three plants of each species were added to each flask. All flasks were manually randomised and placed in a Conviron growth chamber, and their positions were re-randomised every two days to minimise positional effects. Plants were grown for 21 days at 22 °C, 70% relative humidity, under a light intensity of 120 μmol m^−2^ s^−1^ with a 16 h darkness/8 h light photoperiod.

### Preparation of “synthetic roots”

“Synthetic roots” were developed through multi-component co-assembly using an elastin-like recombinamer (ELR) and graphene oxide (GO) as building blocks. This strategy enables the creation of soft and permeable interfacial materials that integrate the electronic and mechanical properties of GO with the enhanced biocompatibility provided by proteins^35^. Graphene oxide (GO) was purchased from Sigma-Aldrich, USA (product n° 777676; 4 mg/mL aqueous dispersion). According to the supplier´s certificate of analysis (batch no. MKCH5557), the GO oxygen content was 45%, the solution pH was 2.0, and morphological analysis indicated a predominance of monolayer sheets. The elastin-like recombinamer (ELR-IK24, sequence: MESLLP-(VPGIG VPGIG VPGKG VPGIG VPGIG)_24_; molecular weight (MW) = 51.9 KDa) was obtained from TP Nanobiotechnology (Spain). The ELR was produced and purified using an *Escherichia coli* recombinant expression system. Both the sequence and MW were confirmed by amino acid analysis, as previously described^72^.

To prepare the “synthetic roots”, an aqueous suspension of GO (2 mg/mL) was first obtained by appropriate dilution with distilled water. Then, 200 µL of this suspension was added to each well of a 96-well plate. In parallel, an aqueous solution of ELR (20 mg/mL) was prepared by dissolving the solid protein in distilled water. The ELR solution was then heated to its transition temperature (30 °C), at which the molecules adopt a conformation characterised by a collapsed aggregated core and an expanded external structure, promoting their interaction with the GO lamella^35^. While hot, 20 µL of the ELR solution was gently dispensed into each well containing the GO suspension, ensuring that the pipette tip touched the bottom of the well before slowly releasing the protein solution vertically. Multilayered “synthetic roots” formed immediately at the interface between the two components, and their complexity was adjusted by maintaining them in the GO suspension for different incubation periods (1 h, 5 h or 24 h). More anatomically complex “synthetic roots” were obtained after 1 h of incubation, whereas less anatomically complex “synthetic roots” were obtained after 24 h. Finally, the structures were washed three times with distilled water to remove excess GO and then incubated with the different media solutions, which were added to the wells containing the “synthetic root”. All materials were UV-sterilised prior to use, dissolved or dispersed in sterile water, and assembled under sterile conditions in a biosafety cabinet.

### Scanning electron microscopy analysis of “synthetic roots”

Scanning electron microscopy (SEM) was used to assess the thickness and microstructure of the “synthetic roots” and to visualise bacterial adhesion. “Synthetic roots” were fixed with 4% paraformaldehyde (Thermofisher Scientific, USA) for 30 min and subsequently dehydrated through a graded ethanol series. Ethanol was removed using a critical point dryer (K850, Quorum Technologies, UK). The dried samples were then sputter-coated with platinum and imaged using a FEI XL30 scanning electron microscope (Thermofisher Scientific, USA).

### Bacterial colonisation experiments using “synthetic roots”

“Synthetic roots” with different morphological complexities were synthesised in individual wells of 96-well plates. Eight biological replicates of each “synthetic root” type were supplemented with 180 μL of 1/1000 SH medium (low nutrient concentration) and inoculated with 20 μL of the bacterial synthetic community prepared as above. Plates were incubated at 28 °C with agitation at 120 r.p.m. for 5 days. Subsequently, five replicates of each “synthetic root” type were harvested into 1.5 mL Eppendorf tubes and stored at −80 °C for microbial colonisation analysis by *16S rRNA* gene sequencing. In parallel, three replicates of each “synthetic root” type were used to visualise and quantify bacterial abundance using 4′,6-diamidino-2′-phenylindole dihydrochloride (DAPI) staining and confocal microscopy.

### Confocal microscopy analysis

For visualisation of bacterial colonisation on “synthetic roots”, samples were fixed with 4% paraformaldehyde for at least 30 min. Samples were then washed with fresh saline solution (0.9% NaCl) to remove excess fixative and incubated for 1 h with a 1/500 DAPI solution in PBS to stain microbial cells. After washing with PBS, z-stack images were acquired using a TCS SP2 confocal microscope (Leica Microsystems, Germany). Bacterial colonisation of the different “synthetic roots” was subsequently quantified by counting the number of blue pixels in the *z*-stack images using Fiji software (ImageJ)^54^.

### DNA extraction from “synthetic roots”

For the DNA extraction, “synthetic root” samples were lyophilised using an Alpha 2-4 LD freeze-dry system and subsequently pulverised in a TissueLyser II (Qiagen) using two cycles of 30 s at a frequency of 30 s^−1^. DNA was then extracted from the “synthetic root” samples using a 96-well-format MoBio PowerSoil Kit (MOBIO Laboratories; Qiagen), following the manufacturer’s instructions. To minimise batch effects, all tubes containing the samples were randomised by placing them in a plastic bag and shaking thoroughly. Tubes with the samples were then randomly retrieved from the bag, and samples were loaded into the DNA extraction plates. This randomisation was maintained throughout library preparation and sequencing. Library preparation and sequencing were performed as described above.

### Computational and statistical analysis of experiments using bacterial synthetic communities

Phylogenetic trees of the bacterial collection and the 20 most abundant ASVs colonising *Lemnaceae* roots were built based on alignments of *16S rRNA* gene sequences (V1-V9 region) generated using the AlignSeqs function from the DECIPHER package (v2.30.0)^61^. Finally, using the chosen ASVs, we created raw count, rarefied (500 reads per sample), and relative abundance tables. The trees were visualised using the ggplot2 package (v3.5.1)^58^.

To calculate ASV enrichment profiles at each nutrient concentration relative to ½ SH nutrient level, we used the DESeq2 package (v1.24.0)^64^. For each ASV in the bacterial abundance tables, differences in abundance across nutrient levels (1/2 SH, 1/100 SH, and 1/1000 SH) were estimated within each plant compartment (root and frond) by fitting a generalized linear model (GLM) with the following design:

$${{{\rm{Abundance}}}}\,\sim\,{{{\rm{nutrient\; level}}}}$$

From the fitted model, we extracted the following contrasts within each plants compartment: ASV abundance at 1/100 SH vs 1/2 SH, and ASV abundance at 1/1000 SH vs 1/2 SH. To visualize the results, we generated a heatmap of log2 fold changes for abundant ASV using the ggplot2 R package (v3.5.1) in R^58^. Both columns (*Lemnaceae* species) and rows (ASVs) were clustered using the hclust function from the R stats package (v4.3.0) in R^59^.

A canonical analysis of principal coordinates (CAP), using the ASV abundance table, was performed to assess beta diversity in plants grown under different nutrient concentrations, with the following design:

$${{{\rm{Bray}}}}-{{{\rm{Curtis}}}}\,{{{\rm{Dissimilarity}}}}\,\sim{{{\rm{nutrient}}}}\,{{{\rm{level}}}}$$

For plants with varying root complexity (measured as the number of root cell layers, CCL), the design used was:

$${{{\rm{Bray}}}}-{{{\rm{Curtis}}}}\,{{{\rm{Dissimilarity}}}}\,\sim {{{\rm{number}}}}\,{{{\rm{of}}}}\, {{{\rm{CCL}}}}$$

We then performed a PERMANOVA using the adonis2 function from the vegan package (v.2.6-4)^67^ to compute the variance explained by nutrient concentration and root complexity (CCL).

To assess correlations between root traits and the microbiota composition in fronds and roots of plants grown under different nutrient concentrations, we compared dissimilarity matrices for each dataset pair individually (root bacterial composition vs. root traits, and frond bacterial composition vs. root traits at each nutrient concentration) using the Mantel function from the vegan package (v.2.6-4)^67^. Briefly, we first calculated the microbiota dissimilarity matrices for roots and fronds using Bray-Curtis distances. Root trait dissimilarity matrices were also calculated using Bray-Curtis distances. Finally, Mantel tests were performed to correlate the matrices by permuting them 1,000 times. Mantel correlations were visualised using the ggplot2 R package (v3.5.1) in R^58^.

We used the DESeq2 package (v1.24.0)^64^ to calculate nutrient level-specific bacterial taxa enrichment profiles. We evaluated differences in ASV abundance at 1/100 SH and 1/1000 SH nutrient levels compared to the 1/2 SH nutrient level across six *Lemnaceae* species, using a generalised linear model (GLM) with the following design:

$${{{\rm{Abundance}}}}\,\sim\,{{{\rm{nutrient}}}}\,{{{\rm{level}}}}$$

To visualise enrichment patterns, we generated a heatmap of log2 fold change for the main taxonomic units, separated by compartment (frond and root) and nutrient level (low and medium), using the ggplot2 package (v3.5.1) in R^58^.

Principal Coordinate Analysis (PCoA) based on Bray-Curtis dissimilarity matrices derived from the ASV abundance table was used to assess beta diversity among “synthetic roots” with different complexity, using the following design:

$${{{\rm{Bray}}}}-{{{\rm{Curtis}}}}\,{{{\rm{Dissimilarity}}}}\,\sim\,{{{\rm{synthetic}}}}\,{{{\rm{root}}}}\,{{{\rm{complexity}}}}$$

We performed a PERMANOVA using the adonis2 function from the vegan package (v.2.6-4)^67^ to quantify the variance explained by the different levels of “synthetic root” complexity.

### Collection of plant root exudates

For the collection of root exudates, 4 mL of sterile 1/1000 SH medium (low nutrient) was added to individual wells in 12-well plates. Three plants from each selected *Lemnaceae* species (*Spirodela polyrhiza* 9509, *Spirodela polyrhiza* 7498, *Spirodela Intermedia* 7820, *Spirodela Intermedia* 9227, *Landoltia punctata* 0049, *Lemna japonica* 9250, *Lemna Tenera* 9243, *Lemna yungensis* 9208, *Lemna perpusilla* 8539, and *Lemna aequinoctalis* 7339), representing a gradient of root complexity, were placed to the corresponding wells. All plates were manually randomised and incubated in a Conviron growth chamber under the following conditions: 70% humidity, 22 °C, 120 μmol m^−2^ s^−1^ light intensity, with 16 h darkness/8 h light photoperiod for 21 days. Plates were re-randomised every 48 h to account for positional effects within the growth chamber. Three biological replicates (three individual wells) were used for each species. At the end of the experiment, all media containing root exudates were collected, centrifuged at high speed to remove plants debris, and subsequently filtered to eliminate small particles.

### Exudates preparation for metabolic analyses

All collected root exudates were prepared for metabolic analysis following a modified version of the protocol described by McLaughlin et al. protocol^44^. Briefly, samples were centrifuged at 15,000 g for 5 min, then 250 µL of supernatant was transferred to new Eppendorf tubes with 750 µL of cold LC-MS grade methanol precooled at −20 °C, then the tubes were shaken for 1 min and incubated at 4 °C for 16 h. After incubation, samples were centrifuged at 15,000 g for 5 min, and the supernatants were carefully transferred to new Eppendorf tubes. Samples were dried again, and a second cycle of resuspension in solvent, vortexing, incubation, centrifugation, and drying was performed. The dried samples were stored at −80 °C, and they were resuspended in 100 µL of methanol for analysis. An extraction blank using a low nutrient medium (1/1000 SH) was subjected to the same extraction procedure.

### LC-MS analysis of the root exudates

Root exudate composition was analysed using the Ultimate 3000 UHPLC system (Thermo Fisher Scientific, Hemel Hempstead, UK) equipped with a ZIC-pHILIC column (4.6 × 150 mm, 5 μm particle size, Merck Sequant, Watford, U.K). The column temperature was maintained at 45 °C, with a flow rate of 300 µL/min. The mobile phases consisted of (A) 20 mM ammonium carbonate and (B) acetonitrile. The gradient elution started at 20% (A) and increased to 95% (A) over 15 min, followed by a return to initial conditions over 2 min and a re-equilibration period of 7 min (total run time: 24 min). The injection volume was 10 µL, and samples were maintained at 4 °C during analysis. High-resolution mass spectrometry was performed using a Q Exactive Plus Orbitrap mass spectrometer (Thermo Fisher Scientific, Hemel Hempstead, UK) operated in both positive and negative electrospray ionisation modes (ESI+ and ESI-). The spray voltages were set to 4.5 kV (ESI + ) and 3.5 kV (ESI-). Sheath, auxiliary, and sweep gas flow rates were set to 40, 5, and 1 (arbitrary units), respectively, for both ionisation modes. Capillary and heater temperatures were maintained at 275 °C and 150 °C, respectively. Data were acquired in full-scan mode at a resolution of 140,000 over an m/z range of 70-1050.

Raw files were processed using Compound Discoverer v3.3 (Thermo Fisher Scientific, Bremen, Germany) for data pre-processing and molecular formula determination. Retention time alignment was performed using pooled quality control (QC) samples. Extraction blanks were used to identify and mark background features. Peak detection was performed using a minimum peak intensity threshold of 1 × 10⁴, and features were retained only if detected in at least 2 files per class. A peak rating threshold of 2 was applied. Only features present in at least 50% of the QC samples were included in the final dataset. Raw files were deposited in the Metabolomics Workbench repository under the Data Track Number ST004637

Background features were flagged using the Mark Background node based on sample-to-blank intensity ratios and excluded from downstream analysis. Missing values were addressed using the Fill Gaps algorithm with a mass tolerance of 5 ppm. and a signal-to-noise (S/N) threshold of 1.5. Molecular formula prediction was performed with a mass tolerance of 5 ppm and a minimum isotopic pattern match of 30%.

### Analysis of microbial community composition in “synthetic roots” supplemented with Lemnaceae root exudates

To assess the effects of metabolic and morphological complexity on microbiota community composition, “synthetic roots” with different levels of morphological complexity (low, medium, and high) were exposed to 180 μL of root exudates extracted from each of the ten *Lemnaceae* species in 96-well plates. Subsequently, all “synthetic roots” were inoculated with 20 μL of a bacterial synthetic community prepared as above. “Synthetic roots” exposed to SH medium served as controls. All plates were incubated in a Conviron growth chamber under the following conditions: 70% humidity, 22 °C, 120 μmol m^−2^ s^−1^ light intensity, and a 16 h darkness/8 h light photoperiod for 5 days.

For microbial community composition analysis, five biologicals replicate of “synthetic roots” from each morphological complexity and treatment were collected in 1.5 mL Eppendorf tubes and stored at −80 °C for DNA extraction and *16S rRNA* gene sequencing. Library preparation and sequencing were performed as described above. Finally, using the chosen ASVs we created raw count, rarefied (500 reads per sample), and relative abundance tables. In parallel, three additional “synthetic roots” replicates from each treatment were stained with DAPI to visualise and quantify microbial colonisation on “synthetic roots” exposed to exudates with differing metabolic complexities. Microbe-colonised “synthetic roots” were imaged using confocal microscopy.

### Lemnaceae species grown for root thermodynamic analysis

For this experiment, we select 10 *Lemnaceae* species covering the full range of root cell layers (CCL) variability observed in this study: *Spirodela polyrhiza* 9509, *Spirodela polyrhiza* 7498, *Spirodela Intermedia* 7820, *Spirodela Intermedia* 9227, *Landoltia punctata* 0049, *Lemna japonica* 9250, *Lemna Tenera* 9243, *Lemna yungensis* 9208, *Lemna aequinoctalis* 7339, and *Lemna perpusilla* 8539. The plants were grown under the same conditions as those used for the collection of plant exudates. After 21 days, the *Lemnaceae* plants were harvested, and the roots were used for further analysis.

### Analysis of the thermal properties of sterile and bacterial colonised roots

In this study, the thermal properties of plant roots were investigated by differential scanning calorimetry (DSC) using a Setaram Micro DSC III (France) controlled by Calisto Acquisition software (v1.062)^73^. The instrument operates on the Tian-Calvet principle and uses temperature- and corrosion-resistant metal cylindrical batch cells (sample and reference cell, 1 mL capacity). Both cells were filled to 75% of their volume: the sample cell contained water and plant roots, while the reference cell contained an equal volume of water only. This filling level was used to minimise free volume above the sample, ensure uniform heat flow throughout the sample, and prevent water evaporation. Samples were heated from 20 °C to 85 °C at a rate of 1 °C/min. Data were analysed using Calisto data processing software to calculate the phase transition peak and enthalpy^32^.

### Computational and statistical analysis of experiments related to “synthetic roots” and plant exudate metabolic analyses

For beta-diversity analysis of the “synthetic root” communities, principal coordinate analysis (PCoA) based on Bray-Curtis dissimilarity matrices calculated from the ASV abundance table was performed. The following model was used to assess the effect of synthetic complexity level:

$${{{\rm{Bray}}}}-{{{\rm{Curtis}}}}\,{{{\rm{Dissimilarity}}}}\,\sim\,{{{\rm{Synthetic}}}}\,{{{\rm{root}}}}\,{{{\rm{complexity}}}}\,{{{\rm{level}}}}$$

To evaluate the effect of *Lemnaceae* species exudates added to the “synthetic roots” at each complexity level (low, medium, and high), the following model was applied:

$${{{\rm{Bray}}}}-{{{\rm{Curtis\; Dissimilarity}}}} \sim {{{\rm{Species}}}}$$

Permutational multivariate analysis of variance (PERMANOVA) was conducted using the adonis2 function from the vegan package (v.2.6-4) in R^67^ to quantify the variance explained by “synthetic root” complexity level and exudate addition effects.

To compute ASV-specific enrichment profiles in “synthetic roots” amended with *Lemnaceae* species exudates relative to the SH medium control, we used the DESeq2 package (v1.24.0)^64^. For each ASV in the bacterial abundance tables, differences in abundance among the different exudates were estimated within each “synthetic root” complexity level (low, medium, and high) by fitting a generalized linear model (GLM) with the following design:

$${{{\rm{Abundance}}}} \sim {{{\rm{Lemnaceae\; exudate}}}}$$

Enrichment patterns (log2 fold change) of the main ASVs were visualised using a heatmap, separately for each “synthetic root” complexity level (high, medium, and low), generated with the ggplot2 package (v3.5.1) in R^58^.

For exudate composition analysis, we constructed a sample x metabolite matrix in which each cell contained the calculated value for a given metabolite in a given sample. Each metabolite was then *z*-score transformed across all samples. To compare metabolite profiles among *Lemnaceae* species, principal coordinate analysis (PCoA) was performed using Euclidean distances calculated from the *z*-score-transformed matrix. PCoA plots were generated using the ggplot2 package (v3.5.1)^58^.

Additionally, a heatmap of the top 100 most abundant metabolites was generated using the pheatmap function from the pheatmap package (v1.0.12)^63^. In this heatmap, both columns (*Lemnaceae* species) and rows (metabolites) were clustered using the hclust function from the stats package (v4.3.0) in R^59^.

To assess differences in the thermodynamic properties of plant roots grown under axenic conditions and those colonised by bacteria, we performed a multivariable analysis of variance (MANOVA) using the stats package (v4.3.0) in R^59^. Thermodynamic curves for each *Lemnaceae* species were visualised using the ggplot2 package (v3.5.1) in R^58^.

Thermal transition responses were quantified by calculating the peak of each thermodynamic curve using the max function from the stats package (v4.3.0) in R^59^. Normalised thermodynamic values for each *Lemnaceae* species were visualised as heatmaps using the ggplot2 package (v3.5.1) in R^58^.

### Synthetic communities’ preparation and inoculation procedure for the Lemnaceae experiments

For this experiment, bacterial synthetic communities were prepared as above. For the drop-out synthetic communities, each of the 20 most abundant ASVs was individually dropped out from the full synthetic community, resulting in 20 drop-out synthetic communities (Supplementary Data 2). Prior to inoculation, the OD_600_ of each synthetic community was adjusted to 0.08, and 10 µL of each synthetic community was added to 4 mL of 1/1000 SH medium before transferring the *Lemnaceae* species. In addition, mono-association experiments were performed in which *Lemnaceae* species were individually inoculated with each of the 20 most abundant ASVs at a concentration of 10^5 ^c.f.u./mL of medium (Supplementary Data 2).

### In vitro plants growth conditions for the drop-out and mono-association experiments

Two *Lemnaceae* species (*Spirodela polyrhiza* 9509 and *Lemna aequinoctalis* 7339), representing contrasting root anatomical complexity, were grown either axenically or inoculated with the full synthetic community, dropout synthetic communities, or the 20 individual ASVs in 4 mL of 1/1000 SH medium in 12-well plates. Growth conditions were as described above. After 21 days, roots were harvested and stored in 70% ethanol at 4 °C for anatomical trait quantification using confocal microscopy imaging. Six biological replicates were used for of each treatment.

### Metabolic analysis of roots inoculated with dropout bacterial synthetic communities and individual ASVs in mono-association experiments

For metabolic analysis of roots colonised by the dropout synthetic communities and individual ASVs, we used two *Lemnaceae* species, *Spirodela polyrhiza* 9509 and *Lemna aequinoctalis* 7339, which represent different levels of root anatomical complexity. Plants were grown in 4 mL of 1/1000 SH medium in 12-well plates, either axenically or inoculated with the full bacterial synthetic community or with dropout bacterial synthetic communities lacking P65, A245, and A289 (Supplementary Data 2). These bacterial strains were previously shown to be important for microbiota-induced root anatomical plasticity. In parallel, plants were also inoculated individually with P65, A245, and A289. Growth conditions were as above. After 21 days, roots were harvested and stored at −80 °C for further analysis.

To extract metabolites from root tissue (9-10 mg), frozen samples were lyophilized and stored at −80 °C until extraction. Dried samples were extracted by adding 500 µL of LC-MS grade 100% methanol, briefly vortexing, and sonicating in an ice-water bath for 10 min. Samples were then centrifuged at 10,000 g for 5 min at 10 °C to pellet cell debris. The supernatant was collected, transferred to new microcentrifuge tubes, and dried using a SpeedVac vacuum concentrator (Thermo Scientific Savant SpeedVac SPD130DLX) at room temperature. The same procedure was performed on four empty tubes to serve as extraction controls. Dried extracts were stored at −80 °C until final resuspension. Samples were resuspended in 100% methanol containing isotopically labelled internal standards (Supplementary Data 4), with the resuspension volume adjusted to normalize by root biomass (60 mg mL^−1^). After resuspension, samples were vortexed, sonicated for 10 min in an ice-water bath, and centrifuged under the same conditions as described above. The supernatant was then transferred to Ultrafree MC-GV centrifugal filters with a 0.22 µm Durapore PVDF membrane (Millipore) and filtered by centrifugation at 10,000 g for 2.5 min. The filtrate was transferred to amber glass vials with inserts for LC-MS/MS analysis.

LC-MS/MS analysis of metabolites was performed using a Thermo Orbitrap Exploris 120 mass spectrometer (Thermo, San Jose, CA) coupled to an Agilent 1290 UHPLC system. Polar metabolites were analysed using normal-phase chromatography following standard methods described previously^74^, while nonpolar metabolites were analysed using reverse-phase chromatography, also as previously described^75^. Briefly, MS1 spectra were collected using a 3 µL injection volume, and MS2 fragmentation spectra were acquired for the most intense ions unless fragmentation for the same m/z had been collected within the previous 4-7 s. Samples consisted of four biological replicates and four extraction controls. The sample injection order was randomized, with a blank injection run between each sample. Internal standards and QC compound mixtures were included for quality control.

For targeted analysis, metabolites were identified by comparing experimental spectra with those of authentic isotopically labelled internal reference standards analysed in-house using the same LC-MS/MS methods. Data were processed using a combination of custom Python code, manual curation, and a workflow implemented in MetAtlas (https://github.com/biorack/metatlas)^76^. Confidence scores ranging from 0 to 3 were assigned to each feature (defined by a unique mass-to-charge (m/z) ratio and retention time (RT)) based on established criteria from the Metabolomics Standards Initiative^77^. A compound was considered positively identified when the detected *m/z* was within ≤ 5 p.p.m. or 0.001 Da of the theoretical value and the RT was within ≤ 0.5 min of the corresponding isotopically labelled internal standard. The highest confidence level (score of 3, exceeding Level 1) was assigned when the feature also exhibited matching fragmentation spectra to the standard. Identifications were invalidated when the acquired MS/MS spectra did not match those of the reference standard. A summary of compound identifications and confidence levels is provided in Supplementary Data 5, along with mirror plots comparing experimental and standard MS/MS spectra (Supplementary Data 10). This dataset has been deposited in the MassIVE (Mass spectrometry Interactive Virtual Environment) repository under the accession number MSV000098287.

### Computational and statistical analysis for experiments using drop-out synthetic communities and individual ASVs in mono-association

For the analysis of root anatomical traits in plant roots colonised with different bacterial synthetic communities and individual ASVs in mono-associations, we constructed a matrix (sample x root trait) in which each cell contained the calculated value for a given trait in a given sample. Each root trait was then standardised across samples using a *z*-score transformation. Pearson correlation coefficients were calculated between all root traits across all treatments. To visualise these correlations, a square matrix (root trait x root trait) was generated, with each cell containing the correlation coefficient resulting for each pair of traits. The correlation heatmap was plotted using the ggplot2 package (v3.5.1)^58^, and both axes were hierarchically clustered using the hclust function from the stats package (v4.3.0) in R^59^. The number of clusters was determined using the ‘Elbow method for k-means clustering’ based on the function fviz_nbclust function in the factorextra package (v1.0.7) in R^78^.

We additionally calculated the coefficient of variation (CV) for each root trait across treatments using the following formula:

$${{{\rm{CV}}}}=({{{\rm{Standard\; Deviation}}}}/{{{\rm{Mean}}}})*100$$

For the analysis of root anatomical traits in plants colonised with different bacterial synthetic communities and individual ASV in mono-associations, we created a matrix (samples x root traits) in which each cell contained the calculated value for a given trait in a given sample. Each root trait was then standardised across samples using a *z*-score transformation. Root traits measured in plants inoculated with the modified synthetic communities or individual ASVs in mono-association were compared with those of plants inoculated with the full synthetic community using a linear model with the following design:

$${{{\rm{Root\; traits}}}} \sim {{{\rm{Synthetic\; communities}}}}$$

After fitting the model, statistically significant differences between conditions were identified using pairwise comparisons. A heatmap was generated to visualize the main root traits using the ggplot2 package (v3.5.1) in R^58^. To better represent the data structure, both columns (drop-out synthetic communities and individual ASVs in mono-association) and rows (root traits) were hierarchically clustered using the hclust function from the stats package (v4.3.0) in R^59^.

For the analysis of root metabolic profiles in plants colonised with the full synthetic community, dropout synthetic communities, and individual ASVs, we created a matrix (sample x metabolite) in which each cell contained the calculated value for a given metabolite in a given sample. Each metabolite was then standardised across samples using a *z*-score transformation. To compare metabolite profiles across treatments for each *Lemnaceae* species, we performed a principal coordinate analysis (PCoA) using Euclidean distances calculated from the *z*-score-transformed matrix for each species. The PCoA results were visualised using the ggplot2 package (v3.5.1)^58^.

### Lemnaceae selection and growth conditions for plant root metabolic profile

To test how root complexity impacts the metabolic profile, we selected nine *Lemnaceae* species representing the variability observed in the number of root cell layers (CCL) within the plant collection used in this study: *Spirodela polyrhiza* 9509, *Spirodela polyrhiza* 7498, *Spirodela Intermedia* 7820, *Spirodela Intermedia* 9227, *Landoltia punctata* 0049, *Lemna japonica* 9250, *Lemna Tenera* 9243, *Lemna yungensis* 9208, and *Lemna aequinoctalis* 7339.

For the experiment preparation, 4 mL of 1/1000 SH medium was added to 5-mL 12-well plates. Twelve biological replicates (twelve individual wells) were used in each case, and half of the biological replicates were inoculated with 10 μL of the full synthetic community prepared as above. Then, three plants of each *Lemnaceae* species were added to the corresponding wells. All plates were manually randomised and positioned in a Conviron growth chamber. Growth conditions were as described above. After 21 days, the *Lemnaceae* plants were harvested, the roots were separated from the fronds, and stored at −80 °C.

### Sample extraction for metabolic analyses

All collected roots were prepared for metabolic analysis according to a modified version of the Moreno-Pedraza et al. protocol^79^. Briefly, the *Lemnaceae* root sample was lyophilised using an Alpha 2-4 LD freeze-dry system. Then, metabolites were extracted from the samples using 1 mL of an extraction solution consisting of methanol: water in a proportion 70:30 (v/v). We used methanol LC-MS grade (Fischer Scientific) and 18.2 MΩ·cm ultrapure water. Afterwards, the samples were sonicated for 15 min in a sonication bath and centrifuged at 15,000 g for 15 min. All supernatants were collected in a new Eppendorf tube. The resulting pellets were re-extracted again using the methanol: water solution and sonicated for 15 min. The samples were centrifuged at 15,000 g for 15 min, and all supernatants were combined with the previously collected supernatants. The resulting solutions, containing metabolites present in the root exudates, were vortexed for 1 min and then filtered using a 0.45 µM syringe filter. The filtered solutions were individually transferred to HPLC vials for LC-MS analysis. A representative sample (QC sample), used to assess the quality of the analytical process, was created by combining 10 µL of each sample.

### LC-MS analysis of the plant root metabolic profile

Root metabolic profile composition analysis was performed as described as above using an Ultimate 3000 UHPLC system (Thermo Fisher Scientific, Hemel Hempstead, UK) equipped with a ZIC-pHILIC column (4.6 × 150 mm, 5 μm particle size; Merck Sequant, Watford, UK). Sample pre-processing was done with Compound Discoverer v3.3 (Thermo Fisher Scientific, Bremen, Germany) as described above. Modules for compound annotation were included in the workflow. An in-house mass list containing 240 standards was included with matches based on MS1 and RT. ChemSpider was used with the databases Biocyc (Chemical Biology Department, Max Planck Institute of Molecular Physiology), Human Metabolome database, KEGG, and PlantCyc, all for accurate mass versus calculated mass with a 5 ppm error. The MZCloud (Thermo Scientific) database was used for MS^2^ data analysis, with thresholds of 80% for MZCloud Best Match and MZCloud Best Match Confidence. Manual curation of the final table was performed to assess the quality of the chromatographic peaks and the reported annotation level^80^. For the significative features, MS2 data that did not pass the MZCloud threshold or did not have match Sirius (6.3.4) was used to achieve a better annotation, a threshold of 0.64 confidence score was used to report features as level 2 for those that did not pass this threshold, chemical class was reported according to ClassyFire classes (https://bio.tools/ClassyFire)^81^. Raw files derived from these analyses were deposited in the Metabolomics Workbench repository under the Data Track Number ST004637

### Selection of Lemnaceae and growth conditions to test the effect of the selected metabolites

To test the effects of the selected metabolites on root plasticity and plant survival, we selected six *Lemnaceae* species representing the range of variability observed in the number of root cell layers (CCL) in our collection: *Spirodela polyrhiza* 9509, *Spirodela polyrhiza* 7498, *Landoltia punctata* 0049, *Lemna japonica* 9250, *Lemna yungensis* 9208, and *Lemna perpusilla* 8539.

For this experiment, we selected the following metabolites: N^6^,N^6^,N^6^-Trimethyl-L-lysine and L-(+)-citrulline. All metabolites were purchased from Thermo Fisher Scientific Inc. (USA). Stock solutions (1 mM) were prepared for each metabolite. The N^6^,N^6^,N^6^-trimethyl-lysine and L-(+)-citrulline were dissolved in water.

For the experiment preparation, 4.75 mL of 1/1000 SH medium was added to 5-mL wells of 12-well plates. The medium was supplemented with 250 μL of a 1 mM solution of each metabolite (final concentration: 50 μM) and 10 μL of the full bacterial synthetic community prepared as above. Three plants of each *Lemnaceae* species were then added to the corresponding wells. Control treatments included plants inoculated with the full bacterial synthetic community alone and plants grown axenically in 1/1000 SH. Six biological replicates were used for each treatment. Growth conditions were as described above. After 21 days, the *Lemnaceae* plants were harvested, and the roots were separated from the fronds and stored at −80 °C.

### Evaluation of the effect of metabolites on the growth of individual members of the bacterial synthetic community

To evaluate the effect of the selected metabolites on individual bacterial growth under in vitro conditions, individual members of the synthetic community were grown in 0.15 mL of 1/1000 SH medium, supplemented or not with each compound at a final concentration of 50 μM, in 96-well plates. Cultures were incubated for 24 h at 28 °C with agitation at 250 r.p.m. Bacterial growth curves were determined by measuring OD_600 nm_ every 4 h over a 24 h period using a FLUOstar® Omega microplate reader (BMG LABTECH).

### Dose effect analysis of the N^6^,N^6^,N^6^-Trimethyl-Lysine

To test the effects of different doses of N^6^,N^6^,N^6^-trimethyl-lysine on plant survival and root anatomical plasticity, we selected two *Lemnaceae* species, *Spirodela polyrhiza* 9509 and *Lemna japonica* 9250, with contrasting inherent root complexity.

We first confirmed the identification of N^6^,N^6^,N^6^-trimethyl-lysine using a Fusion Lumos Tribrid Orbitrap Mass Spectrometer (Thermo Scientific) coupled to a Dionex Ultimate 3000 UHPLC system. An authentic standard of N^6^,N^6^,N^6^-trimethyl-lysine hydrochloride (Sigma-Aldrich, ≥97%, TLC) was prepared at 10 mM in acetonitrile and further diluted to 1 µM for LC-MS/MS injection and comparison. Chromatographic separation was performed using the same column and conditions as in the primary analysis described above (ZIC-pHILIC column; 24-min gradient method).

Ten randomly selected biological samples, including quality control (QC) samples, were thawed from −80 °C and reanalysed alongside the authentic standard. Full-scan MS and data-dependent MS^2^ were acquired in positive ion mode, targeting the precursor ion m/z 189.1597 [M + H]^+^ corresponding to N^6^,N^6^,N^6^-trimethyl-L-lysine. A resolution of 120,000 (FWHM at m/z 200) was used for MS^1^, and 30,000 for MS^2^. Fragmentation was performed using stepped higher-energy collisional dissociation at collision energies of 20%, 35%, and 60%. Raw files were analysed with Compound Discoverer 3.3 (Thermo Scientific), and preprocessing and analysis were performed using an expected-compounds workflow targeting the N^6^,N^6^,N^6^-trimethyl-L-lysine molecule.

For the experiment preparation, 5 mL of 1/1000 SH medium was added to individual wells of 12-well plates. The medium was supplemented with 125, 250, and 375 μL of 1 mM N^6^,N^6^,N^6^-trimethyl-lysine, resulting in final concentrations of 0.025, 0.05, and 0.075 mM, respectively. Each well was then inoculated with 10 μL of the full bacterial synthetic community prepared as described above, followed by the addition of three plants of each *Lemnaceae* species. Control treatments included plants inoculated with the bacterial synthetic community alone and plants grown axenically without metabolites. Four replicates were used for each treatment. After 21 days, plant growth parameters, survival rate, and root anatomy were analysed as described above.

### Preparation of “synthetic roots” with root exudates

“Synthetic roots” containing plant root-exudate metabolites were developed through multi-component co-assembly using an elastin-like recombinamer (ELR) and graphene oxide (GO) as building blocks as described above. The resulting structures were washed three times with distilled water to remove excess GO and then incubated with the different plant metabolite solutions, which were added to the wells containing the “synthetic roots”. Metabolite incorporation is expected to occur primarily through adsorption, mediated by electrostatic interactions, hydrogen bonding, and other noncovalent mechanisms. All materials were UV-sterilised prior to use, dissolved or dispersed in sterile water, and assembled under sterile conditions in a biosafety cabinet.

### Computational and statistical analyses related to the identification and validation of putative metabolites controlling microbiota-driven root plasticity

To generate a heatmap of all identified putative metabolites that were differentially accumulated in plants inoculated with the bacterial synthetic community compared with axenic plants, we used the DESeq2 package (v1.24.0)^64^ to calculate putative metabolite differential abundance profiles. The heatmap was generated using the pheatmap function from the pheatmap package (v1.0.12)^63^. In the heatmap, both columns (Lemnaceae species) and rows (metabolites) were clustered using the hclust function from the stats package (v4.3.0) in R^59^.

To determine differences in metabolite abundance across *Lemnaceae* species and bacterial synthetic community treatments, we fitted a linear model with the following design:

$${{{\rm{Metabolite}}}} \sim {{{\rm{Synthetic\; communities}}}}$$

To generate the heatmap of the 341 putative metabolites that were differentially accumulated in plants inoculated with the bacterial synthetic community compared with axenic plants, we used the DESeq2 package (v1.24.0)^64^ to calculate the metabolite enrichment profiles in the presence of a bacterial synthetic community compared to axenic plants. We used the pheatmap function from the pheatmap package (v1.0.12)^63^. In the heatmap, both columns (*Lemnaceae* species) and rows (metabolites) were clustered using the hclust function from the stats package (v4.3.0) in R^59^.

Metabolites that showed a significant difference in any *Lemnaceae* species were then analysed using linear regression between the enriched profile of the selected metabolite and the number of root cell layers (CCL) in each *Lemnaceae* species used in this experiment. We fitted a linear model with the following design:

$${{{\rm{CCL}}}}\,\sim\,{{{\rm{Enriched}}}}\,{{{\rm{profile}}}}\,{{{\rm{of}}}}\,{{{\rm{selected}}}}\,{{{\rm{metabolites}}}}$$

After this, we selected all metabolites with a *P* value < 0.05. We visualised the standardised relative abundance (*z*-score) of all identified metabolites using a heatmap generated with the ggplot2 package (v3.5.1)^58^.

To assess the effect of metabolite addition on the plant-microbiota interactions, we performed a beta-diversity analysis with Principal Coordinate Analysis (PCoA) based on Bray-Curtis dissimilarity matrices. The analysis was conducted independently for each metabolite using the ASV abundance table. We applied the following model design for the synthetic complexity level:

$${{{\rm{Bray}}}}-{{{\rm{Curtis\; Dissimilarity}}}} \sim {{{\rm{Lemnaceae\; species}}}}*{{{\rm{CCL}}}}$$

We performed a permutational multivariate analysis of variance (PERMANOVA) using the adonis2 function from the vegan package (v.2.6-4) in R^67^ to compute the variance explained by the synthetic CCL. To visualise the results, we plotted the PCoA using the ggplot2 package (v3.5.1)^58^.

To identify the effect of the selected metabolite on bacterial growth after 24 h, we measured the growth rate during the linear phase of the growth curve in response to the metabolite addition and compared it to a control without the metabolite. The slope of the growth curve was calculated using the slope function from the pkr package (v0.1.3)^66^. For each strain, the slope value was log_2_-transformed and normalised to the corresponding control without the metabolite addition. The results were visualised by plotting the mean log_2_-normalised values with confidence intervals using the ggplot2 package (v3.5.1)^58^.

To assess the effect of metabolite addition on microbiota communities associated with “synthetic roots”, we performed a beta-diversity analysis using Principal Coordinate Analysis (PCoA) based on Bray-Curtis dissimilarity matrices. The analysis was conducted using the ASV abundance table. We applied the following design to estimate differences across “synthetic roots” with different morphological complexities:

$${{{\rm{Bray}}}}-{{{\rm{Curtis}}}}\,{{{\rm{Dissimilarity}}}}\,\sim\,{{{\rm{Lemnaceae}}}}\,{{{\rm{species}}}}+{{{\rm{Synthetic}}}}\,{{{\rm{root}}}}\,{{{\rm{complexity}}}}$$

We performed a permutational multivariate analysis of variance (PERMANOVA) using the adonis2 function from the vegan package (v.2.6-4) in R^67^ to compute the variance explained by “synthetic root” morphological complexity. To visualise the results, we plotted the PCoA using the ggplot2 package (v3.5.1)^58^.

### Reporting summary

Further information on research design is available in the Nature Portfolio Reporting Summary linked to this article.

## Supplementary information

Supplementary Information

Description of Additional Supplementary Files

Supplementary Data 1

Supplementary Data 2

Supplementary Data 3

Supplementary Data 4

Supplementary Data 5

Supplementary Data 6

Supplementary Data 7

Supplementary Data 8

Supplementary Data 9

Supplementary Data 10

Reporting Summary

Transparent Peer Review file

## Data Availability

All data generated from this project are publicly available. Raw sequences from the natural water census and the synthetic community colonisation experiments in “synthetic” and real roots are available at the EBI Sequence Read Archive under accession PRJNA1280680. Data from the metabolic analyses, performed at the Joint Genome Institute (JGI), involving plant roots colonised with the drop-out synthetic communities and single ASVs in mono-association experiments, have been deposited in the MassIVE (Mass spectrometry Interactive Virtual Environment) repository under the accession number MSV000098287. The rest of the metabolic data derived from the experiments using root exudates and roots are available at the Metabolomics Workbench repository under the Data Track number ST004637. The processed data are available at GitHub/Data (https://github.com/JuanFrene/Duckweed/tree/main/Data).

## Code availability

Custom scripts used for statistical analysis and plotting are available at (https://github.com/JuanFrene/Duckweed).
