# Supplementary material for: The metabolic and anatomical complexity of root microhabitats modulate their interaction with the microbiota
Source: Nat Commun. 2026 Aug 13;17:9738. doi: 10.1038/s41467-026-76440-4 (PMC13562559; doi:10.1038/s41467-026-76440-4)
Supplement: Supplementary file 1 [file 41467_2026_76440_MOESM13_ESM.pdf]

nature portfolio

Corresponding author(s): Castrillo, Gabriel

Last updated by author(s): Jul 18, 2026

Reporting Summary

Nature Portfolio wishes to improve the reproducibility of the work that we publish. This form provides structure for consistency and transparency in reporting. For further information on Nature Portfolio policies, see our Editorial Policies and the Editorial Policy Checklist.

Statistics

For all statistical analyses, confirm that the following items are present in the figure legend, table legend, main text, or Methods section.

n/a	Confirmed	
☐	☒	The exact sample size (*n*) for each experimental group/condition, given as a discrete number and unit of measurement
☐	☒	A statement on whether measurements were taken from distinct samples or whether the same sample was measured repeatedly
☐	☒	The statistical test(s) used AND whether they are one- or two-sided<br>*Only common tests should be described solely by name; describe more complex techniques in the Methods section.*
☒	☐	A description of all covariates tested
☐	☒	A description of any assumptions or corrections, such as tests of normality and adjustment for multiple comparisons
☐	☒	A full description of the statistical parameters including central tendency (e.g. means) or other basic estimates (e.g. regression coefficient) AND variation (e.g. standard deviation) or associated estimates of uncertainty (e.g. confidence intervals)
☐	☒	For null hypothesis testing, the test statistic (e.g. *F*, *t*, *r*) with confidence intervals, effect sizes, degrees of freedom and *P* value noted<br>*Give P values as exact values whenever suitable.*
☒	☐	For Bayesian analysis, information on the choice of priors and Markov chain Monte Carlo settings
☒	☐	For hierarchical and complex designs, identification of the appropriate level for tests and full reporting of outcomes
☐	☒	Estimates of effect sizes (e.g. Cohen's *d*, Pearson's *r*), indicating how they were calculated

Our web collection on statistics for biologists contains articles on many of the points above.

Software and code

Policy information about availability of computer code

Data collection: The root sections taken with Confocal microscope and Lemanaceae pictures for growth analysis were analysed using ImageJ (polar transformation plugging) (version ImageJ 1.54f).

Data analysis: Summary of data analysis software:

R v4.3.0 was used for statistical analyses, including functions from the base stats package. All figures were plotted with ggplot2 v3.4.2 unless otherwise stated.

Lemnaceae collection analysis:
The Lemnaceae collection tree was constructed using the AlignSeqs function from the DECIPHER v2.30.0 package.
The map of the United Kingdom was created using ggplot2 v3.5.1 and geographr v1.0.1 packages in RStudio.

Root anatomical traits analysis:
For the analysis of the root anatomical traits, we created a matrix (sample x root trait) in which each cell was filled with the calculated parameter in a given sample. Afterwards, we applied a Z-score transformation of each root trait across the samples in the matrix. Next, to compare the root trait profiles of plants grown in sterile media and in the presence of the bacterial natural or synthetic community, we applied a Principal Component Analysis (PCA) using the Euclidean distance between samples and the z-score matrix. To visualize each Lemnaceae species' root trait, a heatmap plot was created using the ggplot2 package v3.5.1 in R. A windrose figure was created using the coord_polar function from ggplot2 R package v3.5.1 to show the number of cortical cell layers (CCL) and the diameter of each layer across the Lemnaceae species grown sterile or natural water and in media with different nutrient levels and inoculated or not with the bacterial

synthetic community.

Plant growth measurement:
To quantify the plant growth, we measured the total frond area in each flask at different time points. All values were normalised against day 0. To compare the total frond area across each plant species and time points in plants grown in natural and sterile water, we used the t.test function from the stats package v4.3.0. To visualize the individual growth curves, we used the ggplot2 package version 3.5.1 in R. To measure the growth rate at the linear phase of the growth curves, the slope of each curve was calculated using the 'slope' function from the pkr package v0.1.3 and the max of each curve was calculated using the 'max' function from the stats package v4.3.0. To visualize each Lemnaceae species' growth parameters, a heatmap plot was created using the ggplot2 package v3.5.1 in R.

16S rRNA sequencing analysis:
Amplicon data were demultiplexed and trimmed with Cutadapt v4.6, and subsequently processed using DADA2 v1.24.0. The SILVA SSU 138 database was used for microbial taxonomic classification. Finally, using the selected ASVs, we created raw count, rarefied (500 reads per sample), and relative abundance tables.

Natural water bacterial communities analysis:
To determine the general characteristics of the microbiota of Lemnaceae roots inoculated with the natural water, the resulting abundance tables were processed and analysed using the phyloseq package v1.46.0. To analyse the alpha diversity across species and plant compartments, we calculated the Shannon index using the phyloseq package v1.46.0. ANOVA test followed by a Tukey's post-hoc test was used to evaluate significant differences among species. For beta diversity, principal coordinate analysis (PCoA) was performed using Bray-Curtis dissimilarity matrices derived from the ASV abundance table. In addition, we performed a permutational multivariate analysis of variance (PERMANOVA) using the adonis2 function from the vegan package (v.2.6-4) in R to assess the variance explained by species and the number of root cell layers (CCL). Pairwise correlations between microbiota composition (PCoA1 axis) and anatomical root traits (PCoA1 axis) were assessed using a Mantel test implemented via the mantel function from the vegan package (v.2.6-4). The resulting correlations were visualised using the ggplot2 package (v3.5.1) in R.
We used the DESeq2 package (v1.24.0) to calculate compartment-specific bacterial taxa enrichment profiles for frond, water, and root samples separately. The abundance differences of each taxonomic unit were assessed across six taxonomic levels, Phylum, Class, Order, Family, Genus, and ASV, comparing root and frond compartments to water for the 19 plant species studied. Enrichment patterns (log2 fold change) of the main taxonomic units at each taxonomic level were visualised in heatmaps, separated by compartment (frond and root), using the ggplot2 package (v3.5.1). Unique ASVs specific to a single compartment (water, frond, or root) were identified using the ps_venn function from the MicroEco package (v1.13.0). A bar plot illustrating the number of unique ASVs in each compartment was generated using the ggplot2 R package (v3.5.1)

Bacterial synthetic communities analysis:
To determine the general characteristics of the microbiota of Lemnaceae roots inoculated with the bacterial synthetic community, the resulting abundance tables were processed and analysed using the phyloseq package v1.46.0.
To analyse the alpha diversity across treatments, species, and nutrient conditions, we calculated the Shannon index using the phyloseq package v1.46.0. ANOVA test followed by a Tukey's post-hoc test was used to evaluate significant differences among treatments. A canonical analysis of principal coordinates (CAP), using the ASV abundance table, was utilised to calculate the beta diversity in plants grown across the different nutrient concentrations for plants with different root complexity (number of cortical cell layers (CCL)). We performed a permutational multivariate analysis of variance (PERMANOVA), using the adonis2 function from the vegan R package v.2.6-4 to compute the variance explained by the nutrient concentrations and the root complexity, measured as the number of cortical cell layers (CCL).
To calculate correlations between root traits and the microbiota composition in fronds and roots of plants grown in different nutrient concentrations, we compared dissimilarity matrices for each pair of datasets individually (root bacterial composition vs. root traits and frond bacterial composition vs. root traits in each nutrient concentration level) using the Mantel function from the vegan R package v.2.6-4. Briefly, we calculated the frond and root bacterial microbiota dissimilarity matrix using the Bray-Curtis distance. Then, we calculated the root trait dissimilarity matrix using Bray-Curtis distance. Finally, we used a Mantel test to correlate the matrices by permuting the matrices 1,000 times, and Mantel correlations were plotted using the ggplot2 R package v3.5.1 in R.

Exudate composition analysis:
Z-score transformation was applied for each metabolite across the samples in the matrix. Next, to compare the metabolic profile of each Lemnaceae species, we applied a principal component analysis (PCA) using the Euclidean distance between samples, and the z-score matrix as input for each Lemnaceae species. Additionally, we created a heatmap with the top 100 most abundant metabolites using the pheatmap v1.0.12 package. In the heatmap, both columns (Lemnaceae species) and the rows (metabolites) were clustered using the stats package v4.3.0 in R. The linear regression analysis between the number of root cortical cell layers and the metabolites PCA1 axis was determined using the lm function from the stats package v4.3.0 in R.

Thermodynamic properties analysis:
To determine the differences in plant roots grown under axenic conditions and those colonised by bacterial synthetic community, we used the Multivariable analysis of variance (MANOVA) analysis from the stats package v4.3.0 in R. To measure the thermal transition response, the peak of each thermodynamic curve was calculated using the 'max' function from the stats package v4.3.0 in R. The correlation between the number of root cortical cell layers and the value of the thermal pick for each Lemnaceae species across the two treatments (axenic roots and roots colonised by the bacterial synthetic community) was determined using the stats package v4.3.0 in R.

Metabolic profile analysis:
Principal component analysis (PCA) using Euclidean distance between samples and the z-score matrix as input for each Lemnaceae species was used to ascertain the metabolic profile. We used the DESeq2 package (v1.24.0) to calculate the metabolite enrichment profiles in the presence of a bacterial synthetic community compared to axenic plants. To generate a heatmap of all identified putative metabolites (2373) and the 341 putative metabolites that were differentially accumulated in plants inoculated with the bacterial synthetic community compared with axenic plants, we used the DESeq2 package (v1.24.0) to calculate putative metabolite differential abundance profiles. The heatmap was generated using the pheatmap function from the pheatmap package (v1.0.12). In the heatmap, both columns (Lemnaceae species) and rows (metabolites) were clustered using the hclust function from the stats package (v4.3.0) in R. To identify the compounds involved in root complexity, a comparative investigation was conducted between Lemanaceae species across the two treatments (axenic roots and roots

colonised by the bacterial synthetic community) using fold change assessment. Subsequently, the differences in fold changes were compared with the root cortical cell layers (CCL) values of each species by linear regression analysis, selecting those with a p-value < 0.05. The linear regression analysis between the number of root CCLs and the metabolites fold change was determined using the lm function from the stats package v4.3.0 in R.

Bacteria growth inhibition analysis:
The growth rate at the linear phase of the growth curves in response to the metabolite additions and the control was compared. The slope of each curve was calculated using the pkr package v0.1.3, and normalized.

For manuscripts utilizing custom algorithms or software that are central to the research but not yet described in published literature, software must be made available to editors and reviewers. We strongly encourage code deposition in a community repository (e.g. GitHub). See the Nature Portfolio guidelines for submitting code & software for further information.

Data

Policy information about availability of data

All manuscripts must include a data availability statement. This statement should provide the following information, where applicable:

- Accession codes, unique identifiers, or web links for publicly available datasets
- A description of any restrictions on data availability
- For clinical datasets or third party data, please ensure that the statement adheres to our policy

All data generated from this project is publicly available. Raw sequences from the natural water census and the synthetic community colonisation experiments in synthetic and real roots are available at the EBI Sequence Read Archive under accession PRJNA1280680. Data from the metabolic analyses performed at JGI is available under accession MSV000098287. All metabolic data are available at the Metabolomics workbench under the Data Track ID ST004637 . The processed data areis available at GithHub/Data

Research involving human participants, their data, or biological material

Policy information about studies with human participants or human data. See also policy information about sex, gender (identity/presentation), and sexual orientation and race, ethnicity and racism.

Reporting on sex and gender	Not applicable
Reporting on race, ethnicity, or other socially relevant groupings	Not applicable
Population characteristics	Not applicable
Recruitment	Not applicable
Ethics oversight	Not applicable

Note that full information on the approval of the study protocol must also be provided in the manuscript.

Field-specific reporting

Please select the one below that is the best fit for your research. If you are not sure, read the appropriate sections before making your selection.

☒ Life sciences ☐ Behavioural & social sciences ☐ Ecological, evolutionary & environmental sciences

For a reference copy of the document with all sections, see nature.com/documents/nr-reporting-summary-flat.pdf

Life sciences study design

All studies must disclose on these points even when the disclosure is negative.

Sample size	No statistical methods were used to pre-determine sample sizes. In the growth, dropout, metabolic, and synthetic root experiments, the total number of samples used for each condition or species was always equal or greater than four. The Lemnaceae exudates profile, DAPI-measured synthetic root bacterial abundance, and thermodynamics property analysis used three (3) replicates.
Data exclusions	No samples were excluded from the analyses.
Replication	Figures 1a-c. A total of n = 5 independent biological replicates. Figures 1d. A total of n = 5 independent biological replicates, except n=4 for LM9200, LM8389, LP7760, LT9243. Figures 1f-g. A total of n = 5 independent biological replicates. Figures 2a, 2c-e. A total of n = 3 independent biological replicates. Figures 3. A total of n = 6 independent biological replicates. Figures 3a-c. Figures 4a-e. A total of n = 6 independent biological replicates. Figures 4f. A total of n = 5 independent biological replicates. Supplementary Figures 1b. A total of n = 5 independent biological replicates, with a minimum of five plants per replicate, were used. Supplementary Figures 2, 3a-b, 4, and 5. A total of n = 5 independent biological replicates. Supplementary Figures S6a and S6b. A total of 5 independent biological replicates, except n=4 for Root: LM9200, LM8389, LP7760, LT9243; Frond: SI9227 and LA7339. Supplementary Figures 6c, 7, 8, and 9. We used n = 5 independent biological replicates, except n=4 for Root: LM9200, LM8389, LP7760, LT9243; Frond: SI9227 and LA7339; and Water: LY9208, SI9227, and SP9192. Supplementary Figure 10. Each point represents the mean of n = 5 independent biological replicates (except n=4 for SI9227 and LA7339).

	Supplementary Figure 11. n = 5 independent biological replicates. Supplementary Figures 13, 14, and 15. We used n = 4 independent biological replicates. Supplementary Figure 16a. n = 4 independent biological replicates. Supplementary Figures 16b-c. n = 4 independent biological replicates for frond samples and n = 3 independent biological replicates for root samples. Supplementary Figures 17, 18, and 19a. n = 3 independent biological replicates. Supplementary Figures 19b-e. n = 6 independent biological replicates. Supplementary Figure 20. n = 4 independent biological replicates. Supplementary Figure 21. n = 5 independent biological replicates. Supplementary Figures 22, 23, and 24a-b. n = 6 independent biological replicates. Supplementary Figures 24c. n = 3 independent biological replicates.
Randomization	For the Lemanecea growth, all the flask and plates were randomized during the incubation time, and randomly shuffled every two days to minimize location-based effects. For the analysis of bacterial microbiota, the order of samples was randomized during the DNA extraction, library preparation, and sequencing steps.
Blinding	Numbers were used to avoiding condition identification during sampling.

Reporting for specific materials, systems and methods

We require information from authors about some types of materials, experimental systems and methods used in many studies. Here, indicate whether each material, system or method listed is relevant to your study. If you are not sure if a list item applies to your research, read the appropriate section before selecting a response.

Materials & experimental systems

n/a	Involved in the study	
☒	☐	Antibodies
☒	☐	Eukaryotic cell lines
☒	☐	Palaeontology and archaeology
☒	☐	Animals and other organisms
☒	☐	Clinical data
☒	☐	Dual use research of concern
☐	☒	Plants

Methods

n/a	Involved in the study	
☒	☐	ChIP-seq
☒	☐	Flow cytometry
☒	☐	MRI-based neuroimaging

Dual use research of concern

Policy information about dual use research of concern

Hazards

Could the accidental, deliberate or reckless misuse of agents or technologies generated in the work, or the application of information presented in the manuscript, pose a threat to:

No	Yes	
☒	☐	Public health
☒	☐	National security
☒	☐	Crops and/or livestock
☒	☐	Ecosystems
☒	☐	Any other significant area

Experiments of concern

Does the work involve any of these experiments of concern:

No	Yes	
☒	☐	Demonstrate how to render a vaccine ineffective
☒	☐	Confer resistance to therapeutically useful antibiotics or antiviral agents
☒	☐	Enhance the virulence of a pathogen or render a nonpathogen virulent
☒	☐	Increase transmissibility of a pathogen
☒	☐	Alter the host range of a pathogen
☒	☐	Enable evasion of diagnostic/detection modalities
☒	☐	Enable the weaponization of a biological agent or toxin
☒	☐	Any other potentially harmful combination of experiments and agents

Plants

Seed stocks	All Lemnaceae species used in this work are from the Landolt collection (ETH Zurich; http://www.duckweed.ch), except Spirodela polyrhiza 9509, which was donated by Prof Klaus Appenroth from Friedrich Schiller University Jena, Germany. Pistia stratiotes was obtained from JAM Aquatics, Wrexham, United Kingdom.
Novel plant genotypes	None
Authentication	Not applicable
