# Supplementary material for: The metabolic and anatomical complexity of root microhabitats modulate their interaction with the microbiota
Source: Nat Commun. 2026 Aug 13;17:9738. doi: 10.1038/s41467-026-76440-4 (PMC13562559; doi:10.1038/s41467-026-76440-4)
Supplement: Supplementary file 2 [file 41467_2026_76440_MOESM14_ESM.pdf]

nature portfolio

Peer Review File

The metabolic and anatomical complexity of root microhabitats modulate their interaction with the microbiota

Corresponding Author: Dr Gabriel Castrillo

This file contains all reviewer reports in order by version, followed by all author rebuttals in order by version.

Version 0:

Reviewer comments:

Reviewer #1

(Remarks to the Author)
The manuscript of Frene et al reports about the effects of root anatomy on root-associated bacterial communities and the role of a specific metabolite in modulating this community with feedbacks on root anatomy and plant performance. The authors report the results of a broad range of different experiments, centred around Lemnaceae species as model. They selected species with different complexity in root anatomy and studied the implications this has on root bacterial communities, how this in return can modulate plant root traits and provide benefits to the plant. The work includes experiments using artificial roots with different traits, use of synthetic bacterial communities, variation in plant nutrient supply and experiments focussing on the role of root metabolites to resolve plant-bacteria interactions and feedbacks. The authors present very interesting results that advance our knowledge and deserve to be published.
Having said that, I see different shortcomings that prevent the publication of this work as it is. Most importantly, some of the conclusions are not well supported by the presented data. In addition, the manuscript has many limitations in the presentation of results, which made it very difficult to follow the main story, i.e. statements are not always supported by the figures referred to. This requires systematic improvement.
In addition, the manuscript includes an unusually high number of analyses and data, which are described in a very condensed way. I had to spend a lot of time to understand the work, as it requires reading of results, methods and figures / figure legends in parallel to get all relevant details. It would help if some more details would be given in the results section and if the figure legends would be more informative. This is also outlined more specifically below, but the authors should consider further improvement beyond these points.
The introduction is very short a bit more background information would be helpful, especially why the work was done with Lemnaceae. Reading statements about the value of the findings of this study for agriculture and plant breeding programs in the abstract, it is surprising to read in l. 70 for the first time that the model plant here was duckweed. This calls into question the transferability of findings.
Regarding the comments below, please note that I do not necessarily point out recurring issues repeatedly, check systematically.
Specific comments:
Abstract:
It should be mentioned in the abstract that the study uses Lemanceae as model system
l. 40: is this a plant or microbial derived metabolite?
Results:
l. 69: maybe better “differences” than “changes”?
l. 70: I had to look into the supplementary methods to understand that only the water was collected from a nature reserve and not the plants.
l. 74: does “various” mean different root types? Or what is varying? Not clear at this point.
l. 80: “intact natural water”, why “intact”?
l. 87: I do not see this in Fig. S1c, this type of presentation does not allow to see this easily. Remove reference to Fig. S1c.
l. 94 and elsewhere: please add “gene” behind 16S rRNA, you did not analyse RNA, but the gene encoded by DNA
l. 95: The post-hoc tests do show some differences, though it is stated here that no significant differences were seen. Please provide results that support the statement and/or adjust the statement. Perhaps, the overall test statistic was not significant? This is not reported anywere.
l. 95-96: I found in the supplementary methods the information that the weakest sample was represented by 500 reads. This is not much, does it represent one of the outliers in Fig. S3a? Usually, samples are rarefied and samples with insufficient

coverage are removed to have robust comparisons of alpha diversity. How many reads did the other samples have and can you do a robust comparison here without rarefying the data; i.e. do the obtained read numbers cover the diversity in all samples adequately? Please provide more evidence that you have robust datasets.
l. 97: please provide information on the type of correlation (linear?!) and the R2-value in this text.
l. 101: why on specific bacterial taxa, it might define the total carrying capacity for the microbiota
l. 101-104: I disagree with this statement; the R2-values are very low and even lower for compartment than for species, so sample fractions (=compartments) does not explain most of the variation.
l. 114: if you emphasize that Proteobacteria are enriched in roots, is that compared to water or why to you mention them again to be enriched in fronds?
l. 118: I am confused here, because the text says CAP, which is a constrained analysis, whereas figure 1d shows a PCoA plot, which is the outcome of an unconstrained analysis.
l. 120: what made you select “number of cortical cell layers” as independent variable here and not any of the other quantified root traits?
l. 125: it would be useful to provide the correlation coefficients along with the p-values.
l. 139: it should become clear that this faster growth was not observed for all plant species
l. 154: the authors use in Fig. 1f CCL as measure for root complexity; it would be helpful to know why the authors selected this particular trait among all those that were analysed as measures for root complexity and to demonstrate the dependency between plant fitness and root traits
l. 155: I don’t see the statement visualized in Fig. 1e, 1f and Fig S5 a and b; if so it needs information what to look at to conclude about root complexity
l. 163: again, it should become very clear whether plants were collected from natural habitats and directly used for isolation or only water, which was then used to culture plants in the lab for isolation
l. 164: why was this isolation procedure done based on different plant species? At least W. australiana has not been used in the preceding experiment, for S. polyrhiza it appears to be a different species here, should be clarified.
l. 164: Australiana should not be written with capital letter
l. 164: methods state the use of three natural waters
l. 167: methods say 10 different media
l. 168: methods report 255 strains and Table S3 lists 245 and legend of Fig. S6a 195 strains, what is correct or why these differences?
l. 181: I do not understand this statement and how Fig. 1 and Fig. S1 inform about nutrient availability in the water, nutrient data are not presented and how can there be a range of nutrient availability if only one type of water is used?
l. 211: would be good to report p- and r-values in the text here; besides, the correlation should not be too much emphasized, because the p-value is not very convincing.
l. 220: change instead of changes
l. 220-235: here, a bit more background information and guidance would be helpful to understand this approach; what does e.g. phase transition mean and what are root constituent components? What are the analysed materials?
l. 222: bacterial instead of bacteria
l. 230: please provide more information about the statistics here in the text (type of correlation, R-value, p-value); this will enable a better understanding especially about the “non-significant correlation”.
l. 245-249: Did the authors perform DAPI staining with synthetic roots that were not incubated in the presence of the SynCom, with the aim to demonstrate that DAPI does not bind to these roots and show false-positive signals?
l. 257: data for 10 plant species are presented, but here it says 9
l. 266: it is said that the experiment was repeated; do the authors mean the experiment described in the preceding sentence (which the wording suggests, but appears unlikely) or the experiment described in the preceding sections (which the wording does not say, but appears more likely).
l. 267-268: I do not see hat Fig. S8h supports this statement and recommend a regression analysis to support the statement.
l. 270: in Fig. S8j, I see responsive ASVs for roots of high and medium complexity, not only high; I wonder if the statement here, saying that beta diversity only changed in high complexity roots, would be better confirmed if a more sensitive q-value of <0.05 would be chosen to highlight responsive ASVs? Or can you argue with stronger fold-changes under high complexity to support your claim? As now, the Fig. does not convincingly support the claim and I would I recommend focussing on the statistical analyses shown for Fig. S8i to support it.
l. 272: looking at the statistics given in figure S8k, there is no significant correlation, the statement here appears incorrect (as elsewhere, it would be convenient to have information about the type of correlation as well as of p and R-values given in the text).
l. 264-275: taken together the results presented in this section, the data are not very convincing to support the conclusion. Alpha diversity does not show a clear consistent trend depending on CCL (Fig. S8h), it would require further proof to confirm this. Beta diversity appears to be only affected in high complexity roots, but in the PCoA plot there is no convincing evidence that CCL plays a role (Fig. S8i). The correlation visualized in Fig. S8k seems not to be significant.
l. 279: move comma behind reference to Fig. 1
l. 283: reference to Table S4 appears incorrect here
l. 284: what does “nutrient limited” mean here? 1/100 or 1/1000 HS?
l. 289: table S4 is not a meaningful here
l. 284-289 and Fig. 3b: does the clustering seen in Fig. 3b support the grouping of correlated variables into three groups?
l. 288, 293: “number of aerenchyma” appears to lack information, aerenchyma does not appear to be a countable unit.
l. 284-307: I am lost here; the authors state that they focus in their analyses on three specific root traits (l.288-289), but the subsequent reporting of results does not focus on these three traits, nor do the figures in Fig. S9.
l. 291: I would find it valuable to get the genus and species names for these three taxa; they play a major role in the following.
l. 344-346: is this correlation equally good in axenic plants and plants with Syncom?

l. 358-360: Why is trimethyl-lysine highlighted here and subsequently in the focus of this study? Figure 4c lacks important information to understand this (see comments below) and Fig. S12a suggests that other components showed even stronger inhibitory effects than this one across the tested plant species. Further, Fig. S12b does not indicate a survival problem related to this compound, compounds such as glutaric acid appear to be much more detrimental. Where does growth reduction end and toxicity begin? Why not going for pyrone, which reduces growth as well? The reason for a focus on trimethyl-lysine as key component requires more convincing justification or better explanation of the results.
l. 364: The reference to Fig. S12c does not appear meaningful and I do not understand the relevance of Fig. S12c.
l. 368-371: maybe I get tired, but I do not understand how this statement is supported by Fig S12d and e.
l. 373-374: similar problem here with Fig. 4e: it is said that the trimethyl lysine treated plants assembled an atypical root microbiota, but this cannot be seen in Fig. 4e, because the treated and non-treated plants are shown in different plots, so I cannot assess differences in the communities between these plants. Further, Fig. S13a and b do not show any results for trimethyl lysine as far as I can see.
l. 375-377: it would be helpful to report the R2-values of the permanova to evaluate the disappearance of differences, because the p-values with trimethyl-lysine are only hardly non-significant in Fig. 4e. Fig. 13a and b are again not useful to support this statement.
l. 378: what does “high concentration” mean in this context? How much was applied, also to compare with Fig. 4d? Info could be added to figure legend (Fig. 4e).
l. 381: what does between-sample variation mean here? And how does Fig. S13c support the statement in l. 380-384? (applies also to Fig 4f). It is very hard to follow the argumentation here and see it supported by the figures.
Figures:
The text in nearly all figures is too small and not readable. In several cases, even enlargement of the figures does not allow to read text due to poor resolution. This applies also to figures in the supplementary material.
Figure 1a: what does PS stand for? Please add the similarity measure and tree reconstruction algorithm. What does the RPL16 gene encode? Why only 18 plant species/accessions shown and not 19?
l. 482: gene, not gen
l. 486: It should probably be “water” here, not “waters”
l. 489: difference, not different
Fig. 1d: it is very hard to see if species:CCL is significant or not; what do the error bars show, SD or SE? What method was used to assess the explained variance?
Fig. 1f: specify type of correlation in figure legend
Fig. 2b: a scale bar would be helpful in the SEM images to allow an estimation of cavity sizes and better compare these structures with those of real roots, e.g. the images in Fig. S1g, which actually also lack scale bars (or the size of the black bars, which may be scales).
l. 522, 528: If I got it correctly, these incubations were done with diluted SH medium; please specify this in the text here.
l. 524: “con”?
Fig. 2d: how many images were analysed here?
Fig. 3a: not readable at all. Don’t understand the color codes; the taxonomic information that can be extracted appears of little value for the understanding of the manuscript. Genus- and species-names would be more informative in such a figure, but even with this information, this figure does not appear relevant for the further understanding; the authors do not discuss taxonomy effects.
Fig. 4b and c: x-and y-axis labelling have text formatting problems
Fig. 4c: is a control treatment available without added metabolite that could be added here for comparison? Were these data obtained after 7 days of growth? And which plant species was used to generate these data here?
Fig. 4d: x-axis labelling should read percentage, not porcentage
Fig. 4e: the color code is not explained (red/blue) and it remains unclear why the plot on the right includes only four of the six plant species. Difficult to draw conclusions without understanding these details.
Supplementary material:
Page numbers would be very helpful for such an extensive document and the file size should be reduced. It slowed down Word substantially, especially when looking at the plots on page 50.
Supplementary methods:
The methods section should be better structured, following the structuring in the results section.
Some methods appear to be described redundantly such as DNA extraction in section 2.9 or data analyses procedures, which are repeatedly described for different datasets. Also, plant growth conditions are described repeatedly. I did not check but would guess that it is always the same.
I miss information about sequencing depth and minimum read numbers or defined for rarefaction. This is not systematically given for all data sets, but important information to evaluate the quality of the presented results. The 500 reads mentioned raise questions about the robustness.
1.3: were all plants cultivated in the same way before transferring them to the flasks for the 21-d incubation experiment? Can we exclude that some plants came in with a different microbiome than others due to cultivation conditions used before?
1.3: Conviron is a company, what is the instrument? Can you add information about the light intensity?
1.6: I assume the 1.5 ml Eppendorf tubes with the samples were put in the plastic bag? Please specify accordingly, I got confused here.
1.7: the Lundberg reference lacks information about the year; please give nucleotide concentrations and polymerase in U, not only volumes; in the second PCR, what was the assay composition and was a PNA-specific step indeed required here?
2.2: please note, 16S rRNA gene sequencing does not allow to identify redundant strains reliably, because different strains of the same species can have identical 16S rRNA gene sequences; phrasing is not precise here
2.2: as before, volumes of primer solutions are not informative
2.3: considering that 10 different media were used for enrichment and isolation, I am surprised to see that all isolates were cultured here on LB medium; did the authors validate adequate growth of the individual strains?

Supplementary figures:
The text in the figure legends has language mistakes. As there are no line numbers given, but a lot of text, I do not point them out, but ask for systematic proof-reading.
I miss detailed information that explains all elements in the figures, e.g. about statistical tests that were applied or number of replicates. Only later, I found this information in the supplementary methods section. The authors should consider adding this info to the figures directly.
Similarly, several figures show dendrograms, but information is missing regarding their reconstruction, i.e. what kind of dissimilarity matrix was calculated and based on which algorithm was a dendrogram calculated.
Fig. 1c: I do not understand what the vertical data represent. I do get that this is the water treatment and the 19 different plant species, but the plot includes many more samples. What are these?
Fig 1e: It is said here that 21 root traits were identified in panel c that were used to run the PCoA. However, Fig. 1c shows only 21 traits displayed, so there was no selection of traits beyond what is shown in Fig. 1 c and d?! (though Fig. 1d shows only 20 traits as far as I can see).
Figure 1e: it appears that the CCL numbers are color-coded by a continuous color gradient, this would not be meaningful if CCL numbers are integer numbers. Actually, it is unclear if the CCL numbers of individual plants are already mean values of multiple counts. If not, the color code should be adapted accordingly. Please ensure that also other plots have appropriate color gradients, CCL is quite often color-coded.
Fig. 1f: It is said that the data were clustered according to plant species. I assume the authors mean the sorting of species, illustrated by the dendrogram. This dendrogram is not explained in the legend, does it reflect phylogeny and based on which algorithms was it constructed? However, as the authors emphasize the decreasing number of significantly differential features for plants with less complex roots, I recommend a plant species sorting that reflects root complexity instead of phylogeny.
Fig. 1g: this figure is not mentioned in the text, as far as I can see.
Fig 2b: I do not understand what “number of aerenchyma” means? And what has caused the authors to present these root traits from the 21 that were determined and are presented in Fig. S1? Are these the ones with significant findings?
Fig. 3a: what kind of statistical test was applied here and what is the overall result of it?
Fig. 3b: the aspect of mortality remains unexplained; what does it mean, how was it determined, and is it actually relevant here?
Fig. 3c: what is the compartment “inoculum”?
Fig. 3 e-f: x-axis labeling should be “sequences”, not “sequence”
Fig. 4 b-d: There are several grammatical mistakes (singular/plural) in the figure legend text related to these plots.
Fig. 4e: to keep it as intuitive as possible, I recommend adding the error bars to this figure, likewise as in Fig. 1d.
Fig. 4g: as far as I can see, PCoA2 was selected here for frond microbiome, whereas PCoA1 was selected for the root microbiome. Why not PCoA1 for frond microbiome, wouldn’t that more likely result in significant mantel correlation?
Fig 5c: what type of correlation; also here, it is very difficult to differentiate significant from non-significant correlations
Fig. 6a: not readable at all; be more precise in wording it is not simply “16S rRNA”, but 16S rRNA gene sequences; further, 16S rRNA should not be written in italic; what is the algorithm used for tree reconstruction here?
Fig. 6b: this figure is not informative and should either be modified or removed
Fig. 6c could be removed, the information given here appears to be redundant to Fig. 1a, in which the relevant taxa might eventually be marked; further, for the subsequent experiment (thermodynamics) another selection of plant species was made, which is not specifically highlighted either, so why this selection here?
Fig. 6d and e: how many replicates? Would it be possible to label the plant species with low, medium and high root complexity? Are the panels possibly sorted by complexity? That will help to quickly see possible changes in dependence on root complexity.
Fig. 6e: what is the unit of the rate?
Fig. 6h: appears to present the same data as 6f; I don’t see that both are needed. The authors never refer explicitly to only one of these.
Fig. 8a: legend says “after microbial colonization”, but I assume “with microbial colonization” would be more meaningful here? Do the figures show data of individual plants or replicates?
Fig. 8f: “six species” is confusing, because data on 10 plant species are presented
Fig. 8g: why was PCoA axis 2 chosen for the correlation analysis and not PCoA axis 1, which covers more variation? Besides, the reference to Fig. 8f appears incorrect, the PCoA plot is shown in Fig 8e.
Fig. 8i: it seems that the dots in the PCoA plot represent results from replicates, i.e. are mean values; explain this more clearly in the figure legend. Likewise, the statistical test applied here is not clear.
Fig. 8k: I was already wondering when looking at Fig. 8h and 8i whether the CCL-numbers were specifically determined in this experiment for each plant sample used to collect root exudates or whether the CCL-number are taken from the first experiment, in which root traits were determined for all plant species; I initially assumed that the existing data were used, but what I find confusing here is the variation in CCL numbers for each of the individual symbols in the plot, with CCL numbers being not limited to integer numbers. This suggests that all replicates presented here were treated with root exudates from specific plant replicates, each consisting of multiple plants that were analysed individually regarding CCL to obtain mean values?
Fig. S10: is a selection of metabolites shown here or all? Legend for fold-change does not display “-“ correctly; dendrograms unclear.
Fig. S11a: this figure appears redundant to the one in Fig. 1a and without novel information. Though, following the description in the figure legend, I do not understand how the data about plants grown axenically or inoculated with Syncoms are implemented in this figure.
Fig. S11b-d: why are these heatmaps so blurry? Each metabolite should have a specific abundance in each of the plant species, color gradients between adjacent entries do not appear meaningful.
Fig. S11b and c: it is said in the legend that the data are organized according to the number of CCL, indicated by the boxes

at the bottom of the plot, but these do not indicate a systematic sorting along a color gradient. Futher, I do not get what the difference between these two figures is and why both are needed.
Fig. S11d seems not to be relevant to support the statement in l. 351-353.
Fig. S11e, legend: the reference to panel d does not appear meaningful. And what kind of correlation analysis was performed here?
Fig S12a: figure legend below the six panels has text formatting problems; similarly, Fig. S12e has problems.
Fig S12d: I do not understand this plot, the color coding is problematic, there are different colors in the plot and in the legend and the syncom, represented by squares, is only depicted once despite the fact that it was applied with different concentrations of trimethyl-lysine. I cannot conclude anything here that supports the statements in the text.
Fig S12e: I do not understand the control termed "NB". The legend says that fold-changes between plants with Syncoms vs. plant with Syncoms plus trimethyl-lysine are displayed. The Control is supposed to show plants without syncom, so how to calculate a fold-change here? Was a +/- trimethyl-lysine treatment applied and if so, which concentration?
Fig. S13b and c: show again these diffusive color gradients that do not appear meaningful
Fig. S13c: legend says that synthetic roots and conditions are the same as in panel c, this is panel c!
Fig. S13d: only one of the six figures is required.
Supplementary tables:
I recommend adding all smaller tables to the supplementary data file and keep only the large table as extra file, especially if each table has to be downloaded separately later.
Table S2: I find it very useful to use R2A medium for aquatic samples, but it is unclear why there was methanol in that medium? That is not a usual component of R2A. Besides, correct the word "methanol". Check also mannitol. Lastly, I recommend harmonizing the writing of the chemicals.
Your table S4 is labelled as table S5 in the excel file.

(Remarks on code availability)

Reviewer #2

(Remarks to the Author)
Review Frene et al
The metabolic and anatomical complexity of root microhabitats modulate their
interaction with the microbiota
Title stays very general but suggests a causal relationship
Abstract – only clear hard core message ".. specific metabolite N6,N6,N6-Trimethyl-L-Lysine has been identified as controlling "root anatomical plasticity"
The Introduction is very short, stays very general, remains vague about the scale to be addressed and meanders between morphology and anatomy of roots and single cells. No actual state of the art is presented. No discussion of system complexity or reductionist approach is made. Very disappointing introduction. The introduction provides no information on the plant system to be investigated.
Only from the results section one learns that the article is obviously about aquatic plants; i.e. plants naturally not growing in soil – this is a very poor management of readers expectations.
I continued reading until the middle of the results section without getting across any exciting novelty – there I stopped reading.
My conclusion:
May be the authors describe something exciting, but it is very disappointing that they failed to inform the reader, from the title, abstract and introduction, about the system they are dealing with – aquatic plants. They completely ignore terrestrial systems, which is fine if they stated this, but they do not. I even think that they could learn a lot from reading the literature on terrestrial plants/ land plants. In my opinion, this is completely unsuitable for the journal.

(Remarks on code availability)

Reviewer #3

(Remarks to the Author)
This manuscript explores the relationship between root anatomical complexity, metabolic profiles, and microbiota assembly in Lemnaceae. The combination of in vivo experimentation, synthetic root models, and metabolomic analysis is original and yields a convincing framework linking structural and metabolic root traits to microbial colonisation. The identification of N6,N6,N6-trimethyl-L-lysine as a regulatory metabolite represents a significant and novel finding.

Nevertheless, several areas require clarification or improvement before publication.

Major Comments

1. Mechanistic Clarity

The role of N6,N6,N6-trimethyl-L-lysine in controlling microbiota-driven root plasticity should be elaborated.

Does this compound act directly on microbial physiology, on plant signalling, or both?

Include discussion of possible biochemical or redox mechanisms and whether this metabolite could influence methylation or stress-response pathways.

L258-260: explain/elaborate this concept.

2. Root Complexity vs. Plasticity

Distinguish clearly between inherent anatomical complexity and microbiota-induced anatomical plasticity.

The manuscript occasionally conflates these concepts, which could confuse readers regarding causality.

3. Reproducibility and Methodological Transparency

Provide detailed descriptions of synthetic-root material composition and preparation.

Include LC–MS/MS parameters used for metabolite identification and confirm annotation quality.

State the number of biological replicates and normalisation (e.g., 16S data).

4. Data Presentation and Structure

The abstract and results are dense; reduce descriptive language and emphasise key findings.

Improve figure captions: define abbreviations (e.g., CCL), consolidate statistical details, and ensure clarity of legends.

Consider adding a schematic summarising the anatomical–metabolic–microbiota feedback model to aid reader understanding.

Minor Comments

Ensure consistent taxonomic and metabolite nomenclature throughout.

Revise the conclusion to focus on implications for microbiome optimisation and crop improvement.

(Remarks on code availability)

Reviewer #4

(Remarks to the Author)
Authors in this manuscript entitled “The metabolic and anatomical complexity of root microhabitats modulate their interaction with the microbiota” have gathered a large and thorough amount of experiments and data to study the interaction and correlation between root anatomy and its plasticity with the microbiota composition/recruitment and nutrient availability in duckweed. The experiments include using synthetic root models and synthetic microbiota with 16S rRNA sequencing and metabolomics approaches to study different variables independently.

My comments are largely on the metabolomics results and their significance. Unfortunately, the methodology included in the Extended Material is very scarce and does not allow me to evaluate correctly the results from the metabolomics approaches. The metabolomics processed data is also not included as supplemental material. I would also strongly recommend submitting the raw metabolomics data to a data repository such as MetaWorkbench.

Major comments:

Section 3.2/3.3 in the Extended Material; for the analysis of the root exudate using LC-MS with Zic-pHILIC column on QE-plus, there is no details on how the data was further processed: normalization, QC correction, blk subtraction, comparison; and statistically analyzed?? What data was used to create Extended Figure 8f (terrible resolution of the figure) and 8g. How many features are detected? How were they evaluated? How were the most abundant selected? Authors must add a Supplemental Table with this information.
Section 4.4/4.5 in the Extended Material; for the analysis of the Lemnaceae roots after dropouts of individual ASVs using LC-MS with Poroshell 120 HILIC-Z column on QE, again there are almost no details on how the data was processed. There is mention of MetAtlas. What is this? Are Authors referring to Metabolic Atlas. This is not a tool to analyze mass spec data. Also, the method seems to suggest that the annotation of the peaks was done using an in-house library of authentic reference standards (by the way how many compounds in the library). However, there are clearly mis annotations based on

the Extended Fig 10a-b which shows heatmaps from the metabolite identified in pos and neg ion modes. These are meant to be complementary scans to allow the detection of greater number of compounds. However, looking at the heatmaps, there is one compound, pantothenic acid, identified by both showing contradictory changes in the same conditions (dA245 in SP9509). This seems to suggest that either the peak annotated in the pos or in the neg is wrongly annotated. Authors again need to provide the data in a supplemental table with the criteria for confidence levels of annotation.
Section 5.2/5.3; for the analysis of the Lemnaceae roots with different CCL using LC-MS with Zic-pHILIC column on QE-plus, again no details on how the data was further processed and annotated. Authors must add the data in a supplemental table and indicate level of confidence for annotations. The few selected compounds annotated were tested for their effect on root plasticity before their annotation was confirmed. In this case, the samples had to be rerun on a Fusion Lumos, as described in Section 5.6, alongside the standard for trimethyl Lysine to confirm the peak was correctly annotated. What about the other compounds selected? Were they also run and confirmed to significantly show the data in Fig 4a, since the authors purchased all the standards?
In addition, how did the authors select the concentration for the dose effect of the trimethylLysine? Did authors measure the concentration of the compound in the root exudate or what could be found in natural water?

Minor comments:

There is a lot of redundancy in the methodology. The method section could be shortened if the authors would refer to sections already described once. This document just looks like a copy and paste from a PhD dissertation. As an obvious example: Sections 3.2/3.3 and 5.2/5.3 are exactly the same.

Figure 3 is of poor resolution.

Extended Section 5.3 – change root exudate to root.

Line 349. “This analysis identified 341 metabolites”. This was confusing to me. It suggested that 341 metabolites were annotated but this is for the correlation of CCL and their abundance profile. Please rephrase it.

Extended Fig 11. The heatmap in panel d) shows features # that do not match with the ones in panel e), which are supposedly the same 15 selected metabolites?

(Remarks on code availability)

Version 1:

Reviewer comments:

Reviewer #1

(Remarks to the Author)
The authors addressed most of my previous comments convincingly. At this stage, I first read the revised manuscript and evaluated the responses to my comments afterwards. Accordingly, you will find a list of new issues first, followed by some comments from last time that require further clarification.
l. 132-133: statement unclear
l. 148: bacterial, not microbiota; here and elsewhere: don’t use the terms microbiota or microbial when referring to your own data, which are limited to the bacterial community
l. 178: sterile water means tap water, distilled water or sterilized natural water? This could become clearer here.
l. 198: better: “microbiota-induced complex root anatomy”
l. 264-266: sentence unclear
l. 301: specify type of change (increase with increasing complexity)
l. 332: microhabitats
l. 542: microbial, not microbiota
l. 602: plants
l. 669: reported p-value for post-hoc tests correct?
l. 671: delete “total”
l. 677: it is stated that a minimum of six samples was available, but the plot (2e) allows to see max. 5 per complexity level, do some replicates have identical communities (especially with decreasing complexity) or is there perhaps still a problem with this plot? Please check.
l. 682: if the author list changes, the author contributions should also be updated; further, not all authors of this manuscript appear in the contribution list, which raises questions
l. 848: axis labelling of Fig 4a says PCA, not PCoA as stated in fig. legend; is this indeed a principal coordinate analysis or a principle component analysis? Further, and in contrast to Fig 4a, the y-axis in Fig 4b is labelled as PCoA, though it was taken from Fig. 4a; what is correct?
l. 850 and 851: should probably be “Samples” instead of “Plant species”, as individual dots represent samples, not plant species.
l. 851-852: I don’t see any error bars in figure 4a; however, error bars are shown in Fig 4b, which remain unexplained; also, add number or replicates

l. 865: p-value cut-off for post-hoc tests?
l. 881-883: statement unclear; according to this description, there would be three treatments (I) exudates of S. polyrhiza, (II) exudates of L. japonica and (III) Trimethyl-lysine, but this is not the case, according to the figure, it is a 2-factorial design; clarify the description in this sentence
l. 884: I assume it is “roots” here, if n=6
l. 887: delete “of”
The supplementary material still includes a very comprehensive description of materials and methods. Redundancies have to some extent been eliminated. However, the description of synthetic roots should be described earlier, in the context of the first experiments using these, not at the end.
Suppl. l. 1297: what type of correlation coefficient?
Suppl. l. 1340: “A minimum of five roots was analysed for each condition (n=4).” This statement appears contradictory.
Suppl. l. 1494: delete “absolute”; this remains a relative measure, as it refers to a specific microscopic area, where cells were counted on an artificial root surface; the differences in root complexity may contribute to an underestimation of counts, especially in the most complex roots, as I assume you cannot quantify cells deeper within the structure; absolute estimates from such images appear thus unlikely. (please correct this wording systematically in the manuscript)
Suppl. fig. 11: some fields in the heatmap appear to be completely black; what does that mean?
Suppl. l. 1705: I don’t see in the figure that plant species are organized according to root complexity; the information about complexity is given by a color coding bar, but there is no obvious sorting according to complexity in fig 12c. Likewise in l. 1714 and fig. 12d. Such sorting would be helpful here.
The formatting of the last two supplementary tables could be improved, not all text is visible and in part not well readable.

Related to former reviewer comments:
Reviewer #1 comment 9 9- l. 87: I do not see this in Fig. S1c, this type of presentation does not allow to see this easily. Remove reference to Fig. S1c.
The problem remains despite the modified figure. It would require a sorting of plant species according to root number (complexity) to clearly see the described differences. The hierarchical clustering, used at present to sort species/treatments, does not provide this information.
Reviewer #1 comment 12 12- l. 95-96: I found in the supplementary methods the information that the weakest sample was represented by 500 reads. This is not much, does it represent one of the outliers in Fig. S3a? Usually, samples are rarefied and samples with insufficient coverage are removed to have robust comparisons of alpha diversity. How many reads did the other samples have and can you do a robust comparison here without rarefying the data; i.e. do the obtained read numbers cover the diversity in all samples adequately? Please provide more evidence that you have robust datasets.
The presented figure in the response letter is not convincing. It clearly shows that many samples reach saturation only with 10.000 reads. Thus, 500 reads are likely not sufficient to obtain robust information on Shannon diversity. What appears more convincing is the statement that alpha diversity was unaffected by read number. It would also be important to evaluate whether samples with low read numbers represent a specific set of samples, as this may lead to biased conclusions.
I do not agree to the solution that the information about data rarefaction has now been removed from the supplementary material in the revised version. I actually requested this information for all amplicon datasets in one of my other comments. This is important information for readers to allow the assessment of data quality. You should demonstrate convincingly that your data are robust despite weak samples or select a higher read number for rarefaction, even if some more samples are lost.
Reviewer #1 comment 46
46- l. 284-289 and Fig. 3b: does the clustering seen in Fig. 3b support the grouping of correlated variables into three groups? Can you specify what criteria or metrics were used to define the three groups in the clustering?
Please add this information about the definition of 3 clusters in the supplementary methods section.
Reviewer #1 comment 51
51- l. 358-360: Why is trimethyl-lysine highlighted here and subsequently in the focus of this study? Figure 4c lacks important information to understand this (see comments below) and Fig. S12a suggests that other components showed even stronger inhibitory effects than this one across the tested plant species. Further, Fig. S12b does not indicate a survival problem related to this compound, compounds such as glutaric acid appear to be much more detrimental. Where does growth reduction end and toxicity begin? Why not going for pyrone, which reduces growth as well? The reason for a focus on trimethyl-lysine as key component requires more convincing justification or better explanation of the results.
The manuscript is now very clear regarding the focus on trimethyl-lysine. However, I wonder if some of the other compounds may likewise play a role, but are now excluded from any further discussion, simply based on the fact that their identification is not as robust? This aspect could be worth being discussed. Is there more to discover in the future, or does the trimethyl-lysine explain it all?
Reviewer #1 comment 113
113-Fig. S10: is a selection of metabolites shown here or all?
Refers to Fig. S11 now; the question remains whether the authors focus on a selection of metabolites here or if the figure includes all compounds that were identified. If a selection was made, based on which criteria? This info could be added to the legend.
Reviewer #1 comment 115 115- Fig. S11b-d: why are these heatmaps so blurry? Each metabolite should have a specific abundance in each of the plant species, color gradients between adjacent entries do not appear meaningful.
Reviewer #1 comment 122
122- Fig. S13b and c: show again these diffusive color gradients that do not appear meaningful.
Just to clarify, some of the heatmaps in the preceding word document (which I opened with Microsoft Word) had smooth colour transitions from one field/tile to the surrounding ones, rather than fields/tiles with clearly defined borders; the new version is not affected by this problem. I recommend paying attention that this does not happen again.

(Remarks on code availability)
Sorry, this is not my main expertise.

Reviewer #3

(Remarks to the Author)
I congratulate the authors for addressing the comments and remarks made during the previous review with seriousness. The manuscript is now much improved.

(Remarks on code availability)

Reviewer #4

(Remarks to the Author)
I appreciate for having addressed most of the comments, including the detailed description of the metabolomics methodology, the submission of raw data to a public repository, and the careful manual curation to ensure inclusion of only confidently annotated metabolites. I have a few minor comments:
• Table S4: The table is unclear. It appears to list isotopically labeled internal standards, but the manuscript does not describe how these standards were used during data processing. Their role in normalization or in validating annotated metabolites is not mentioned. If the standards were not incorporated into the analysis, it may be best to remove this table.
• Table Numbering: In the Table S5 file, the table is referred to as Table S4 for POS mode and Table S6 for NEG mode. Please correct these inconsistencies.
• Extended Data Fig. 11:
o The legend is incorrect—Panel a corresponds to metabolites detected in negative mode, and Panel b corresponds to positive mode.
o Some metabolites removed from Table S5 due to low-confidence annotation are still shown in the figure (e.g., tryptophan in NEG, panel a). Please remove these entries from the figure.
• Result:
The paragraph beginning “To identify metabolites that modulate microbiota driven root anatomical plasticity…” remains unclear. As written, it implies that all 341 metabolites were confidently annotated, although only one of the final 15 is confidently identified. Please revise for clarity. In addition, please provide a supplemental table listing all metabolites that were confidently annotated for this experiment. This would help evaluate whether additional metabolites related to carnitine metabolism (especially lysine and methionine, precursors of TML) are present and further support the authors’ conclusions.

(Remarks on code availability)

Version 2:

Reviewer comments:

Reviewer #1

(Remarks to the Author)
The authors have adequately addressed my concerns and I congratulate to this comprehensive study with very valuable new insights.
I only spotted a last minor grammatical issue in the manuscript text: in l. 55, the word "was" should be deleted.
However, regarding the data provided on Github, it appears that the rearrangement of plots in figures and of complete figures in manuscript and supplement during the review process has not been considered in this data repository. Thus, data and scripts for several of the presented figures cannot be found anymore. It appears that it has not yet been (fully) updated.

(Remarks on code availability)
I only checked for availability and identified the problem mentioned in my response to the authors.

Reviewer #4

(Remarks to the Author)
All of my comments have been addressed, and the revisions are satisfactory. Thank you for your careful attention to the feedback.

(Remarks on code availability)

Open Access This Peer Review File is licensed under a Creative Commons Attribution 4.0 International License, which permits use, sharing, adaptation, distribution and reproduction in any medium or format, as long as you give appropriate credit to the original author(s) and the source, provide a link to the Creative Commons license, and indicate if changes were made.
In cases where reviewers are anonymous, credit should be given to 'Anonymous Referee' and the source.
The images or other third party material in this Peer Review File are included in the article's Creative Commons license, unless indicated otherwise in a credit line to the material. If material is not included in the article's Creative Commons license and your intended use is not permitted by statutory regulation or exceeds the permitted use, you will need to obtain permission directly from the copyright holder.
To view a copy of this license, visit https://creativecommons.org/licenses/by/4.0/

Point-by-point responses to the reviewers' comments

Reviewer #1 (Remarks to the Author)

Reviewer #1 comment 1
1- The manuscript of Frene et al reports about the effects of root anatomy on root-associated bacterial communities and the role of a specific metabolite in modulating this community with feedbacks on root anatomy and plant performance. The authors report the results of a broad range of different experiments, centred around Lemnaceae species as model. They selected species with different complexity in root anatomy and studied the implications this has on root bacterial communities, how this in return can modulate plant root traits and provide benefits to the plant. The work includes experiments using artificial roots with different traits, use of synthetic bacterial communities, variation in plant nutrient supply and experiments focussing on the role of root metabolites to resolve plant-bacteria interactions and feedbacks. The authors present very interesting results that advance our knowledge and deserve to be published.

Authors response: We would like to sincerely thank the reviewer for their positive, valuable and insightful comments and suggestions, which have greatly contributed to improving our manuscript. In response, we have revised the relevant sections of the manuscript to address all the reviewers' concerns.

Reviewer #1 comment 2
2- In addition, the manuscript includes an unusually high number of analyses and data, which are described in a very condensed way. I had to spend a lot of time to understand the work, as it requires reading of results, methods and figures / figure legends in parallel to get all relevant details. It would help if some more details would be given in the results section and if the figure legends would be more informative.

Authors response: We thank the reviewer for this valuable comment and agree that clarification is needed regarding some sections. Following the reviewer's recommendations, we have significantly improved the clarity and quality of the manuscript. Specifically, we have expanded the introduction, clarified the abstract, and revised multiple sections with particular emphasis on the results and figure legends, which now include more detailed explanations of the key findings.

Reviewer #1 comment 3
3-The introduction is very short a bit more background information would be helpful, especially why the work was done with Lemnaceae. Reading statements about the value of the findings of this study for agriculture and plant breeding programs in the abstract, it is surprising to read in l. 70 for the first time that the model plant here was duckweed. This calls into question the transferability of findings.

Authors response: Following the reviewers' recommendations, the introduction has been substantially expanded to include information from recent publications and theoretical perspectives relevant to the topic addressed in this research. We also clarified our rationale for using Lemnaceae species and accessions in this study, as well as the importance of Lemnaceae as an emerging model for investigating plant–microbiota interactions. Following the reviewer's comment, we have removed the sentence referring to knowledge translation from the abstract and have included in this section the model plant used.

Reviewer #1 comment 4
Specific comments:
4- Abstract:
It should be mentioned in the abstract that the study uses Lemanceae as model system
l. 40: is this a plant or microbial derived metabolite?

Authors response: The abstract now includes information about the Lemnaceae model used. We have clarified that the metabolite is plant derived. We have removed from the abstract the sentence related to the translation of findings to relevant crops. Now it reads:
"Plant roots constantly communicate with their microbiota, adapting their anatomy to facilitate microbial colonisation under abiotic stresses. Microbes, in turn, can reshape root anatomy once they establish. However, the mechanisms that coordinate this interplay remain largely unknown. Working with the

aquatic plant family Lemnaceae, we reveal that the inherent complexity of root anatomy determines root plasticity in response to microbial colonisation. This microbiota-driven anatomical plasticity enhances plant survival in nutrient-competitive environments. By combining synthetic root models with real roots, we also find that anatomical plasticity is associated with metabolic reprogramming during microbial establishment. Moreover, we identify **a plant metabolite**, N6,N6,N6-Trimethyl-L-lysine, that regulates anatomical plasticity in response to microbial colonisation. Our work generalizes the importance of the microhabitat complexity for microbiome recruitment under challenging environmental conditions".

Reviewer #1 comment 5
5- Results:l. 69: maybe better "differences" than "changes"?

Authors response: Thanks for the suggestion. Instead of differences in root anatomy, in this experiment we evaluated changes in root anatomical plasticity. This essentially involves plant altering root structure (cortical cell size, aerenchyma-like formation, number of cell layers, etc), to adapt to environmental conditions. Following the reviewer's recommendation, we have improved the sentence, which now reads:

"We evaluated changes in root anatomical plasticity in a collection of plants from the Lemnaceae (duckweed) family (Fig. 1a) available in our laboratory, grown in a natural water collected from Attenborough Nature Reserve, UK, harbouring a natural microbiota (Extended Data Fig. 1a)".

Reviewer #1 comment 6
6- l. 70: I had to look into the supplementary methods to understand that only the water was collected from a nature reserve and not the plants.

Authors response: We have modified this sentence to make it clear that only water was collected from the nature reserve. It now reads: We evaluated changes in root anatomical plasticity in a collection of plants from the *Lemnaceae* (duckweed) family (Fig. 1a and Table S1) **available in our laboratory**, grown in a natural water collected from Attenborough Nature Reserve, UK, harbouring a natural microbiota (Extended Data Fig. 1a).

Reviewer #1 comment 7
7- l. 74: does "various" mean different root types? Or what is varying? Not clear at this point.

Authors response: The word "various" was related to the number of roots present in the plant. To avoid confusion, we replaced the word "various" with the word "multiple." The sentence reads as follows: This collection of 12 species and 6 accessions represents a gradient in the number of roots and in inherent root anatomical complexity, spanning from the earliest-diverging genera (Spirodela and Landoltia) with **multiple** roots and high number of root cell layers to the later diverging genus Lemma with a single root and a reduced number of root cell layers (Fig. 1a-b and Extended Data Fig. 1b).

Reviewer #1 comment 8
8- l. 80: "intact natural water", why "intact"?

Authors response: The term "intact" was originally used to emphasize that, unlike the water we sterilize, this natural water was preserved and used without modification. To avoid confusion, we have removed the term "intact".

Reviewer #1 comment 9
9- l. 87: I do not see this in Fig. S1c, this type of presentation does not allow to see this easily. Remove reference to Fig. S1c.

Authors response: We have reorganized Fig. S1C to improve data visualization and clarify our conclusions. Instead of displaying each replicate individually, we combined all replicates from the same treatment and genotype and used the resulting average values to generate a new Fig. S1C panel. In this updated panel, it is easier to see that the values for anatomical traits, such as CCL2 diameter, CCL2 cell number, CCLe2 cell number, CCLe1 cell number, aerenchyma-like mean area, CCL, number of roots, CCL3 diameter, and CCL3 cell number, are higher in plants with a greater number of roots.

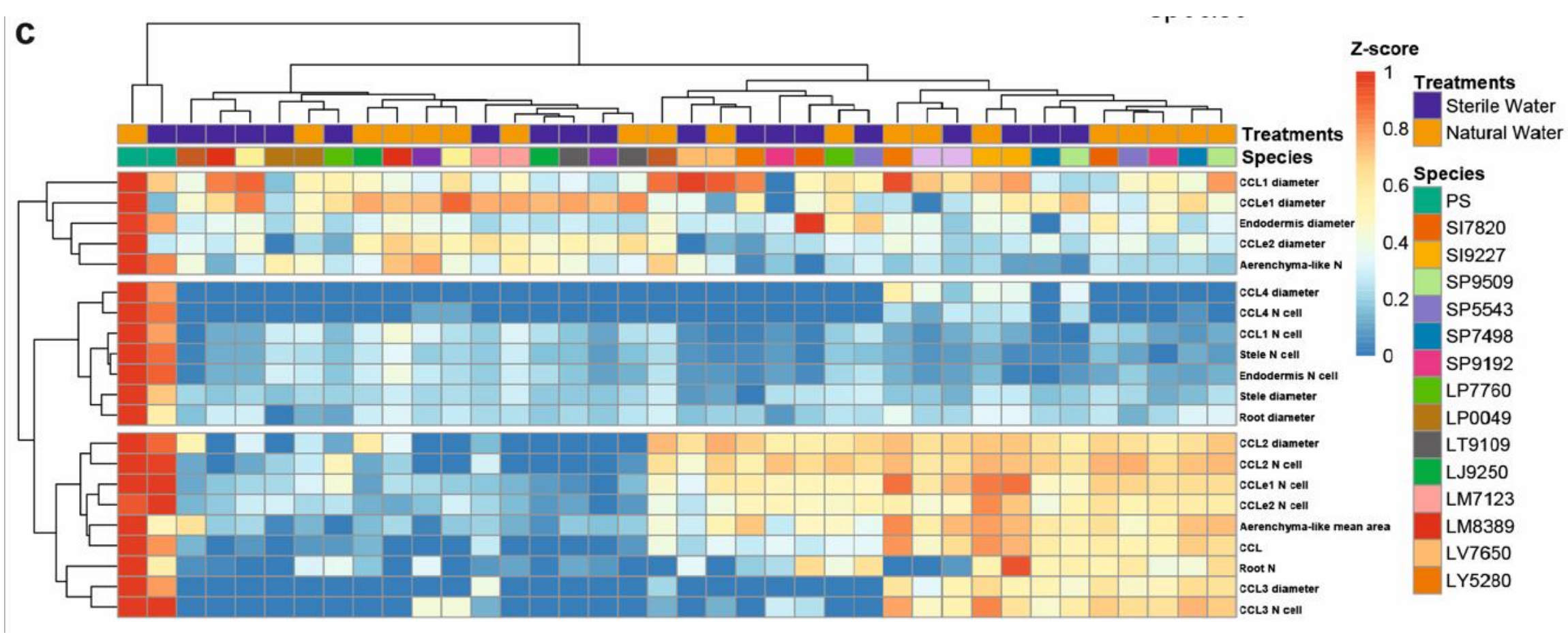

Reviewer #1 comment 10

10- l. 94 and elsewhere: please add “gene” behind 16S rRNA, you did not analyse RNA, but the gene encoded by DNA

Authors response: It has been fixed.

Reviewer #1 comment 11

11- l. 95: The post-hoc tests do show some differences, though it is stated here that no significant differences were seen. Please provide results that support the statement and/or adjust the statement. Perhaps, the overall test statistic was not significant? This is not reported anywhere.

Authors response: We thank the reviewer for this comment. We agree that the original statement was inaccurate. Although our intention was to speak in general terms, only a limited number of significant differences in alpha diversity were observed in roots and fronds across the plant species analysed, and the specific differences were not mentioned. This oversight has now been corrected.

The text reads now: We found significant differences in root and frond alpha diversity in a limited number of the plant species analysed (Extended Data Fig. 4a). For example, in the case of root alpha diversity, we observed significant differences between the group of Pistia stratiotes and Lemna tenera 9243 and the rest of plants analysed. In the fronds, only Pistia stratiotes and Lemna yungensis LY9208 showed significantly different alpha diversities (Extended Data Fig. 4a).

Reviewer #1 comment 12

12- l. 95-96: I found in the supplementary methods the information that the weakest sample was represented by 500 reads. This is not much, does it represent one of the outliers in Fig. S3a? Usually, samples are rarefied and samples with insufficient coverage are removed to have robust comparisons of alpha diversity. How many reads did the other samples have and can you do a robust comparison here without rarefying the data; i.e. do the obtained read numbers cover the diversity in all samples adequately? Please provide more evidence that you have robust datasets.

Authors response: Thank you for pointing this out. We agree that insufficient read depth can bias diversity estimates and should be carefully evaluated. Samples were rarefied to 500 reads to standardize sequencing depth across samples and maximize sample retention. This depth was sufficient to capture overall community patterns based on rarefaction analysis. As a result of this rarefaction threshold, samples with approximately 500 reads represent the lowest sequencing depth in the dataset; however, they do not constitute statistical outliers relative to the overall distribution. All other samples had substantially higher read numbers, ranging from 567 to 139838 reads (mean: 34,310.23 ± SE: 1,590.63), indicating that the majority of samples were sequenced at sufficient depth. Using this threshold, we retained 278 out of 288 samples for downstream analyses. In addition, we found no association between read number and alpha-diversity. To further evaluate whether sequencing depth adequately captured microbial alpha-diversity, we examined rarefaction curves, which approached asymptotes for all samples, including those with lower read counts, suggesting that alpha-diversity estimates are not driven by sequencing depth in this dataset.

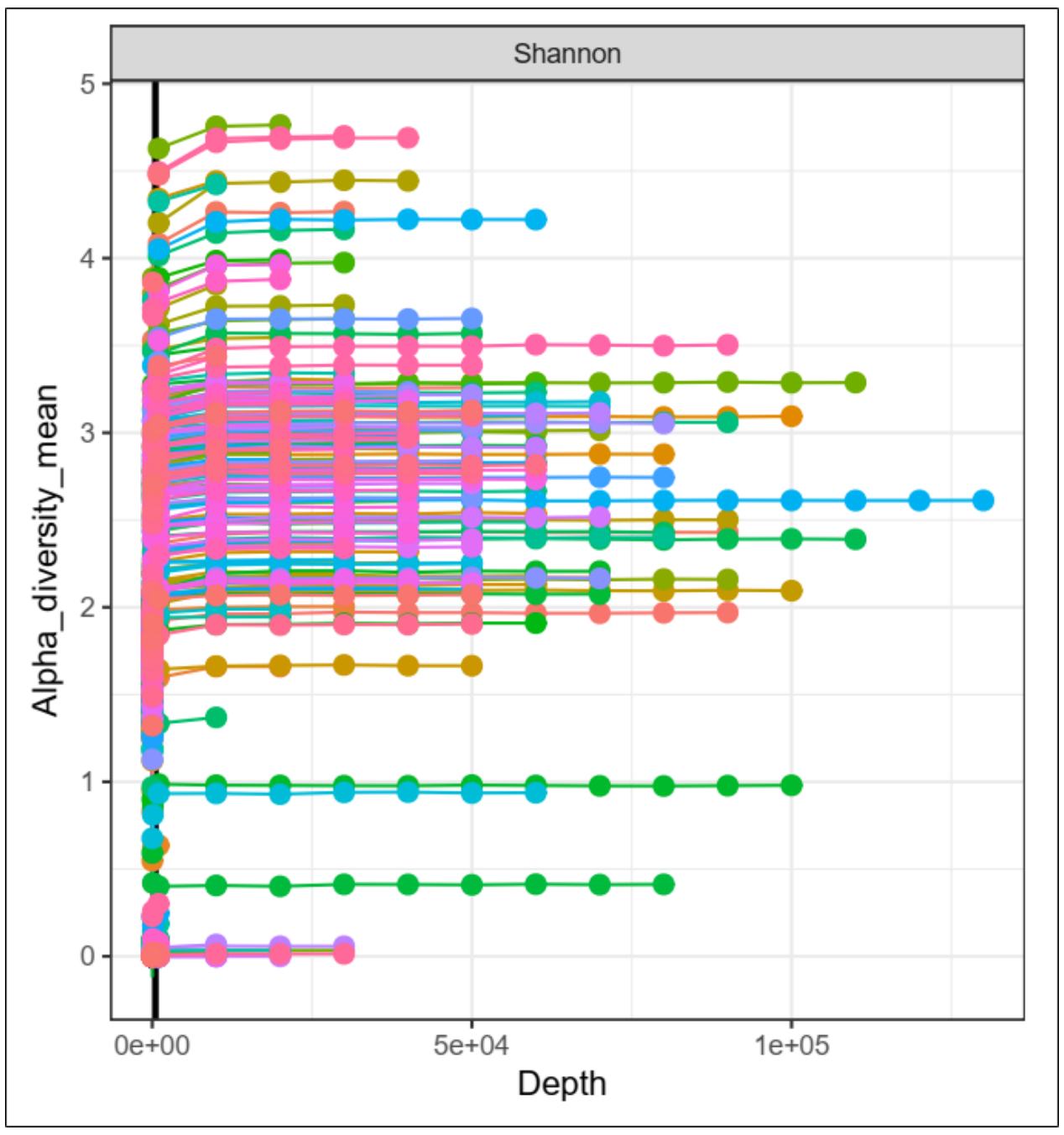

Reviewer #1 comment 13
13- l. 97: please provide information on the type of correlation (linear?!) and the R2-value in this text.

Authors response: This information has been added to the text.

Reviewer #1 comment 14
14- l. 101: why on specific bacterial taxa, it might define the total carrying capacity for the microbiota

Authors response: We thank the reviewer for this insightful comment. While we agree that root anatomical complexity may influence the overall carrying capacity of roots for colonising microbiota, our observations suggest that differences in anatomical complexity have a limited impact on microbial alpha diversity. This might suggest that although root anatomy plays a role in microbial colonization, its effect may be masked by other variables or may primarily affect a few bacterial taxa. To avoid confusion, we have revised this conclusion, which now reads: "This might suggest that root anatomical complexity could have an impact on the bacterial ability to colonize roots"

Reviewer #1 comment 15
15- l. 101-104: I disagree with this statement; the R2-values are very low and even lower for compartment than for species, so sample fractions (=compartments) does not explain most of the variation.

Authors response: We thank the reviewer for this comment. The reviewer is correct; we overlooked the contribution of the variable "species" to the between-sample variation in this case. Duckweed represents a unique system, with roots on a vestigial trajectory and demonstrated impairments in certain root functions. Therefore, in contrast to most terrestrial plants, where the variable "compartment" explains the majority of the differences observed in microbiome structure, species identity appears to play a more prominent role in this system. We misinterpreted the contribution of the variables due to this particularity. This has now been corrected in the text, which reads: "A principal component analysis using all Lemnaceae species showed significant differences in bacterial community composition across the samples. Most of the variance observed between samples was primarily explained by the variable "species" and, to a lesser extent, by the sample fractions (root, frond, and water) (Extended Data Fig.4c). Supporting this observation, significant differences in microbiota community membership

across sample fractions were found in only 6 out of the 19 individual species analysed, suggesting that, under our conditions, the microbiota filtering mechanisms present in the organs of these aquatic plants were laxer compared to terrestrial plants (Extended Data Fig. 4c, d). This is consistent with the progressive anatomical and functional reduction found in the roots of some plants present in the Lemnaceae collection used.".

Reviewer #1 comment 16
16- l. 114: if you emphasize that Proteobacteria are enriched in roots, is that compared to water or why to you mention them again to be enriched in fronds?

Authors response: We clarify in the text that these comparisons are in relation to water.

Reviewer #1 comment 17
17- l. 118: I am confused here, because the text says CAP, which is a constrained analysis, whereas figure 1d shows a PCoA plot, which is the outcome of an unconstrained analysis.

Authors response: We thank the reviewer for this comment. The reviewer was correct; there was an error in the text, which has now been corrected.

Reviewer #1 comment 18
18- l. 120: what made you select "number of cortical cell layers" as independent variable here and not any of the other quantified root traits?

Authors response: We thank the reviewer for this comment. The *Lemnaceae* represent a unique family of plants, characterized by roots following a vestigial trajectory and by plants undergoing miniaturization. These processes result in the loss of some root functions and anatomical traits present in other plants, as well as the compression of anatomical structures, making it difficult to confidently assign identities to the anatomical traits quantified in this study. Accordingly, we have replaced the term aerenchyma with aerenchyma-like structure, as a second analysis revealed insufficient evidence to classify these air spaces as true aerenchyma, despite their morphological similarity. Following the same reasoning, we also removed the terms exodermis and epidermis and renamed them as external cortical cell layers. Among the traits quantified, the total number of root cortical cell layers (CCLs) is the only one we consider reliable. In fact, the mean number of CCLs decreases progressively across the genera studied and effectively reflects root complexity. Furthermore, this trait is present in all species analysed and can be readily identified, even in roots showing clear vestigial features. Other traits vary substantially between species and are sometimes absent. Therefore, we consider that the total number of root cell layers the most reliable proxy for root complexity.

Reviewer #1 comment 19
19- l. 125: it would be useful to provide the correlation coefficients along with the p-values.

Authors response: This information has been added to the text.

Reviewer #1 comment 20
20- l. 139: it should become clear that this faster growth was not observed for all plant species.

Authors response: We thank the reviewer for this comment. For the sake of transparency, we clarify that not all plants analysed showed faster growth in natural water compared to axenic conditions. The revised text now reads:
"We observed that approximately 74% of plants grown in natural water exhibited faster growth during early time points (the linear phase) compared to axenically grown plants (Extended Data Figures 6a and b). In contrast, only 26% of the plants analysed showed similar (*Lemna yungensis* 9208, *Spirodela intermedia* 7820, *Spirodela polyrhiza* 9192, *Lemna tenera* 9243) or lower (*Lemna minuta* 9200) growth rate at early time points in natural water compared to sterile conditions (Extended Data Figures 6a and b)".

Reviewer #1 comment 21
21- l. 154: the authors use in Fig. 1f CCL as measure for root complexity; it would be helpful to know why the authors selected this particular trait among all those that were analysed as measures for root complexity and to demonstrate the dependency between plant fitness and root traits.

Authors response: We thank the reviewer for this comment. As we explained in the comment18, the *Lemnaceae* represent a unique family of plants, characterized by roots following a vestigial trajectory and by plants undergoing miniaturization. These processes result in the loss of some root functions and anatomical traits present in other plants, as well as the compression of anatomical structures, making it difficult to confidently assign identities to the anatomical traits quantified in this study. Accordingly, we have replaced the term aerenchyma with aerenchyma-like structure, as a second analysis revealed insufficient evidence to classify these air spaces as true aerenchyma, despite their morphological similarity. Following the same reasoning, we also removed the terms exodermis and epidermis and renamed them as external cortical cell layers. Among the traits quantified, the total number of root cortical cell layers (CCLs) is the only one we consider reliable. In fact, the mean number of CCLs decreases progressively across the genera studied and effectively reflects root complexity. Furthermore, this trait is present in all species analysed and can be readily identified, even in roots showing clear vestigial features. Other traits vary substantially between species and are sometimes absent. Therefore, we consider that the total number of root cell layers the most reliable proxy for root complexity.

Reviewer #1 comment 22

22- l. 155: I don't see the statement visualized in Fig. 1e, 1f and Fig S5 a and b; if so it needs information what to look at to conclude about root complexity.

Authors response: In the paper, we stated that "plants with complex microbiota-induced root anatomy survived better than those plant species with less complex microbiota-induced roots in the natural water". Supporting this statement, our quantification of healthy (green), stressed (yellow), and dead (white) plants at the end of the growth period showed that, in general, plants with complex root anatomy had higher survival rates than those with simpler root structures when grown in natural waters containing microbiota (Fig. 1e). To improve the interpretation of this figure, we added a lateral panel showing the number of root cell layers per plants species as a proxy for root complexity across microbial treatments. Additionally, we created a new figure showing the average number of dead plants in relation to root cell layers species (new Fig 1f), which clearly demonstrates that plant mortality is generally higher in roots with fewer cell layers.

To further support this conclusion, we analysed the correlation between the number of dead plants and the number of root cell layers. This analysis revealed a significant linear correlation between plant survival and root complexity in plants grown in natural water containing microbiota (new Fig. 1g). In contrast, root complexity did not influence survival in plants grown in sterilized water (new Figs. 1e-g). To enhance interpretability, we increased the visibility of the regression line under sterile conditions (new Fig. 1g). Finally, we removed references to Extended Data Figs. S5a and S5b, as they are not relevant to this experiment. We believe these adjustments strengthen both the clarity and the visual impact of the conclusions from these experiments.

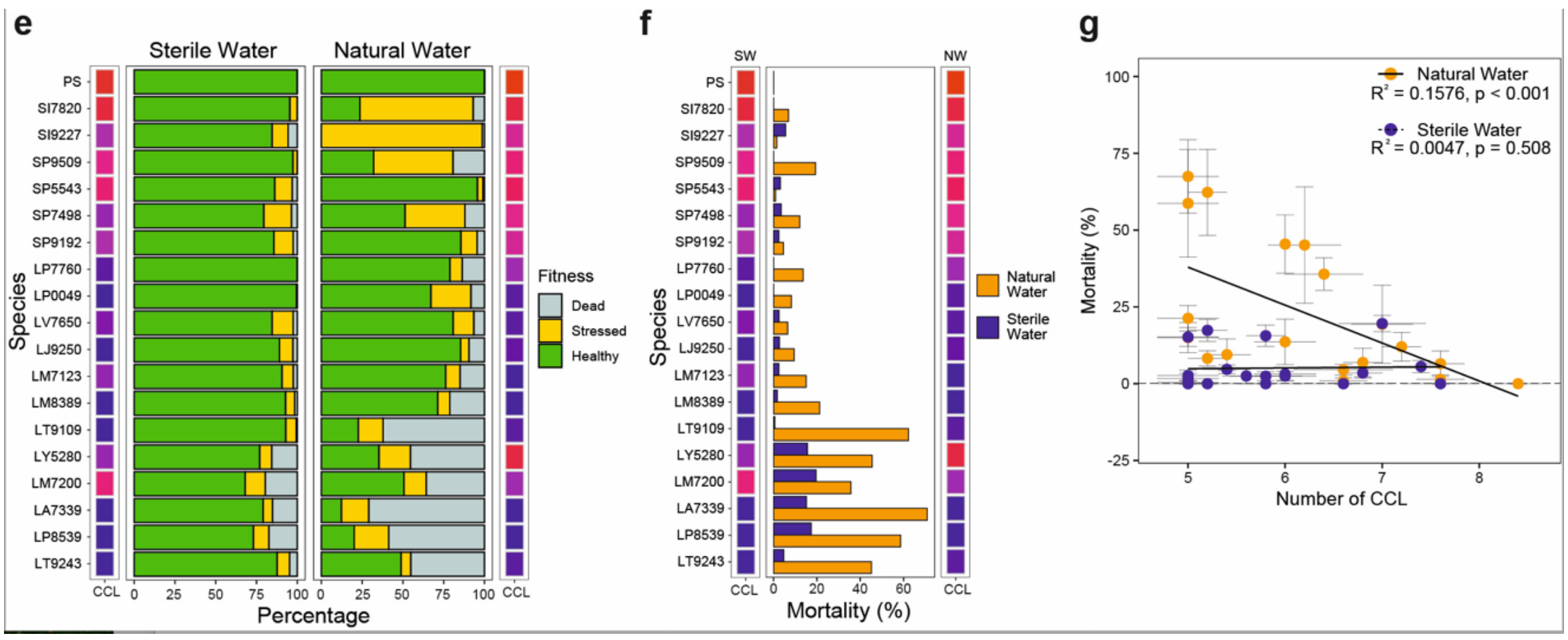

Reviewer #1 comment 23

23- l. 163: again, it should become very clear whether plants were collected from natural habitats and directly used for isolation or only water, which was then used to culture plants in the lab for isolation

Authors response: We have clarified this in the text, which now reads: "We isolated bacterial strains from two Lemnaceae species, *Spirodela polyrhiza* and *Wolffia australiana*, **available in our laboratory**, and grown in two natural waters collected from the arboretum of the University of Nottingham, Sutton-Bonington Campus, and the Peak district Nature Reserve, UK (Extended Data Figure 1a).

Reviewer #1 comment 24
24- l. 164: why was this isolation procedure done based on different plant species? At least W. australiana has not been used in the preceding experiment, for S. polyrhiza it appears to be a different species here, should be clarified.

Authors response: The bacterial collection was designed to capture the full range of root diversity within the Lemnaceae family, from species with no roots (*Wolffia australiana*) to species with multiple roots (*Spirodela polyrhiza*). Because we did not create a separate bacterial collection for each species, and to prevent potential host-specific biases arising from isolations tied to the species used in our experiments, all bacteria in the collection were isolated from species not included in the experiments. This strategy minimizes differences in colonization across the plant collection due to host adaptations, thereby reducing bias that could affect our conclusions.

Reviewer #1 comment 25
25- l. 164: Australiana should not be written with capital letter

Authors response: Fixed

Reviewer #1 comment 26
26- l. 164: methods state the use of three natural waters

Authors response: We apologize for the confusion. The main manuscript is correct; we only used two natural waters. This has now been corrected in the Methods section.

Reviewer #1 comment 27
27- l. 167: methods say 10 different media

Authors response: This typo has been fixed in the main text of the manuscript.

Reviewer #1 comment 28
28- l. 168: methods report 255 strains and Table S3 lists 245 and legend of Fig. S6a 195 strains, what is correct or why these differences?

Authors response: We thank the reviewer for pointing this out. In this study, we identified a total of 245 individual strains, which are now described in Table S2. We have corrected the methods section accordingly to report 245 strains. For the bacterial synthetic community designed in this work, we used 190 strains of the 245 identified strains. The incorrect value has been removed from the Fig. S6a legend.

Reviewer #1 comment 29
29- l. 181: I do not understand this statement and how Fig. 1 and Fig. S1 inform about nutrient availability in the water, nutrient data are not presented and how can there be a range of nutrient availability if only one type of water is used?

Authors response: The reviewer is correct in noting that only one type of water was used. However, this does not take into account that we worked with a collection of different plant species, each with distinct nutrient requirements. In addition, species vary in how they interact with and compete against the microbiota. For some species, the presence of microbiota can enhance nutrient uptake, whereas for others these interactions may be neutral or even detrimental. Given this variability, it is reasonable to assume that flasks containing different plant species grown in the same water with microbiota would not experience identical nutrient availability. Consequently, our experimental setup may reflect a natural gradient of nutrient conditions. To account for these potential differences, we used SH medium at three concentrations (low: 1/1000 SH, medium: 1/100 SH, and high: ½ SH nutrients). We believe that this range effectively captures the variability likely to occur in natural water.
To avoid confusion, we have revised the relevant sentence and removed references to figures. The updated text now reads: "To emulate the wide range of nutrient availability that may be present in the

natural water experiment, resulting from the different species used and their interaction with the microbiota, we grew these plant species at three nutrient concentrations using different dilutions of SH medium (1/2 SH, 1/100 SH, and 1/1000 SH) with or without inoculation with a 190-members bacterial synthetic community comprising 75.5% of the collection strains".

Reviewer #1 comment 30
30- l. 211: would be good to report p- and r-values in the text here; besides, the correlation should not be too much emphasized, because the p-value is not very convincing.

Authors response: This has been fixed in the text.

Reviewer #1 comment 31
31- l. 220: change instead of changes

Authors response: Fixed

Reviewer #1 comment 32
32- l. 220-235: here, a bit more background information and guidance would be helpful to understand this approach; what does e.g. phase transition mean and what are root constituent components? What are the analysed materials?

Authors response: The materials analysed are roots grown either in sterile medium or in medium containing the bacterial synthetic community. A phase transition refers to a change in the physical state of a material (i.e., from solid to liquid, or melting in this study), and thermal methods allow the characterisation of the melting profile of a given material, thereby quantifying structural differences across materials as reflected by distinct melting profiles. In this experiment, we applied a heating ramp at a constant rate of 1°C/min from 20 to 85°C to characterise the structural melting behaviour of the roots. Because there is limited information in the literature regarding the compositional and thermal properties of roots constituents, we employed thermal characterisation to enable a direct comparison of structural melting profiles across growth conditions and to identify key differences associated with those conditions.
We included a better explanation of this experiment in the manuscript: "We determined the specific temperature ranges and heat flux associated with phase transitions of core root components using differential scanning calorimetry. These parameters, which are intrinsically linked to the composition of the analysed materials and can also influence their morphology, were quantified in the root of six species and four accessions of Lemnaceae exhibiting different levels of inherent root anatomical complexity. This analysis compared plants grown axenically with those inoculated with the bacterial synthetic community. By analysing thermal transitions, such as the shift of membrane lipids or starch from a solid to a fluid state, we quantified how bacterial colonisation affects the structural and metabolic stability of root tissues".

Reviewer #1 comment 33
33- l. 222: bacterial instead of bacteria

Authors response: Fixed

Reviewer #1 comment 34
34- l. 230: please provide more information about the statistics here in the text (type of correlation, R-value, p-value); this will enable a better understanding especially about the "non-significant correlation".

Authors response: R^2 and p values have been added to the text in each case.

Reviewer #1 comment 35
35- l. 245-249: Did the authors perform DAPI staining with synthetic roots that were not incubated in the presence of the SynCom, with the aim to demonstrate that DAPI does not bind to these roots and show false-positive signals?

Authors response: We thank the reviewer for the comment. DAPI is generally highly specific for double-stranded DNA, binding strongly to A–T rich regions in the minor groove of dsDNA. Although DAPI can bind to other molecules such as RNA, proteins, membranes, polystyrene, glass, negatively

charged surfaces, and hydrogels, these interactions are weaker and typically result in less intense signals.
In our case, the synthetic roots contain the same concentration of graphene oxide, which can be negatively charged at neutral to basic pH. To address the possibility of non-specific DAPI binding, samples were thoroughly washed with PBS to remove excess DAPI. In addition, we included a negative control consisting of synthetic roots without microbes, which was used to adjust the microscope gain. This gain setting was then applied consistently to all subsequent samples to eliminate background signal arising from the material itself.
Furthermore, Figure 2 clearly demonstrate the absence of background DAPI fluorescence attributable to the synthetic root material and shows that different levels of bacterial colonization can be reliably detected. If the surface had interfered with the staining, it would not have been possible to distinguish differences in colonization across the experimental conditions.

Reviewer #1 comment 36
36- l. 257: data for 10 plant species are presented, but here it says 9.

Authors response: This typo has been corrected.

Reviewer #1 comment 37
37- l. 266: it is said that the experiment was repeated; do the authors mean the experiment described in the preceding sentence (which the wording suggests but appears unlikely) or the experiment described in the preceding sections (which the wording does not say but appears more likely).

Authors response: We repeated all the experiments twice. We agree with the reviewer that the way this paragraph was originally written was confusing. Therefore, we have edited it to improve clarity. It now reads: “Thus, we compared variations in microbiota composition in artificial roots exposed to nine metabolically different exudates, across three levels of synthetic root anatomical complexity. We found a general trend of increasing alpha diversity with greater synthetic root complexity across most of the metabolic combinations tested (Extended Data Fig. 9h)”.

Reviewer #1 comment 38
38- l. 267-268: I do not see hat Fig. S8h supports this statement and recommend a regression analysis to support the statement.
Authors response: Following the reviewer’ recommendations, we have prepared new figure panels showing the individual effect of the nine exudates and controls conditions on alpha diversity across the three levels of synthetic roots complexity. Within each panel, we included the result of corresponding comparison among treatments. Based on these results, we have toned down the text that now reads: “We found a general trend of increasing alpha diversity with greater synthetic root complexity across most of the metabolic combinations tested (Extended Data Fig. 9h)”.

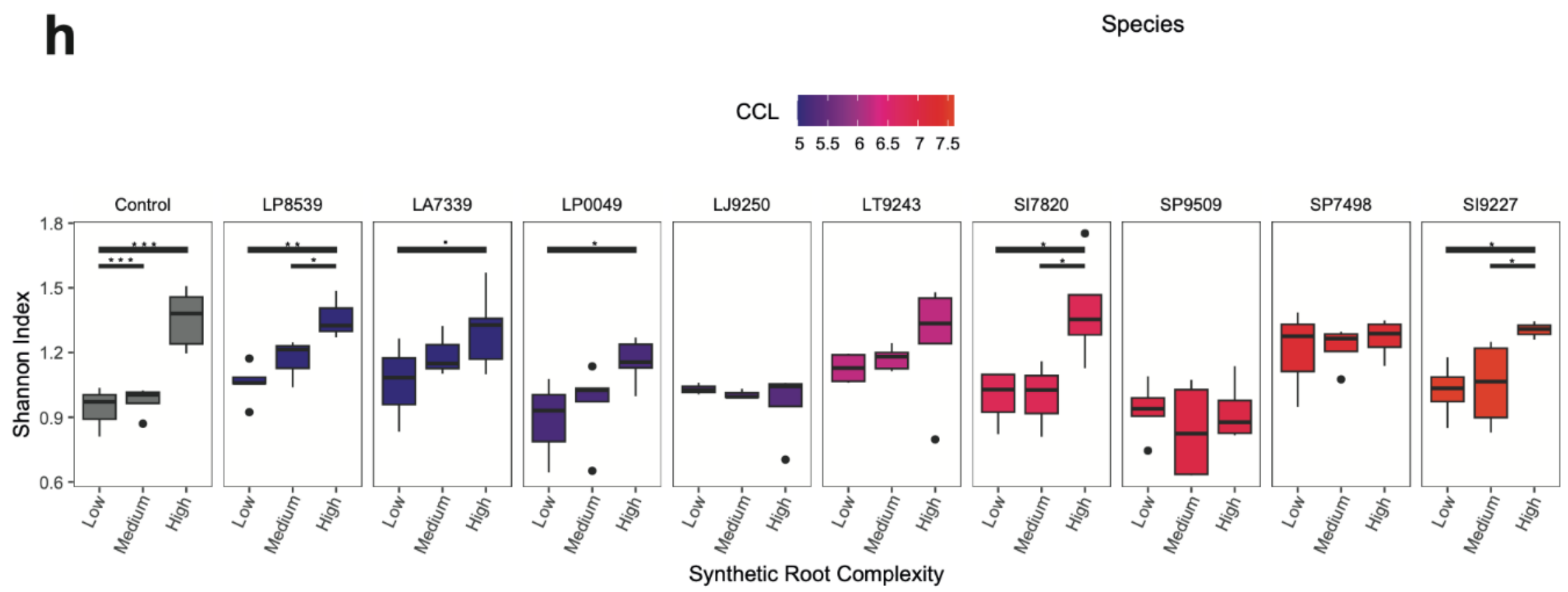

Reviewer #1 comment 39
39- l. 270: in Fig. S8j, I see responsive ASVs for roots of high and medium complexity, not only high; I wonder if the statement here, saying that beta diversity only changed in high complexity roots, would be better confirmed if a more sensitive q-value of <0.05 would be chosen to highlight responsive ASVs? Or can you argue with stronger fold-changes under high complexity to support your claim? As

now, the Fig. does not convincingly support the claim and I would I recommend focussing on the statistical analyses shown for Fig. S8i to support it.

Authors response: We thank the reviewer for this comment. To improve the readability of the manuscript and facilitate understanding of our results, we have now explained Figures S9i and S9j separately. In Figure S9i, we show that significant differences in microbiota structure among samples treated with different plant exudates are observed only when the complexity of the synthetic roots is high. This finding suggests a potential interaction between complex root anatomical traits and metabolism, with important consequences for microbiota recruitment. Supporting this observation, Figure S9j shows that the number of ASVs exhibiting significant changes in relative abundance compared with the control condition (synthetic roots without exudates) is highest in synthetic roots with the greatest anatomical complexity. The analyses address distinct questions: the first compares microbiota composition across different plant species, whereas the second assesses changes in relative abundance across all conditions relative to the control. Together, these results strengthen our conclusions regarding the role of anatomy-metabolism interactions in microbiota establishment. The revised text now reads:
" We observed significant changes (R^2 = 0.508, p = 0.001) in bacterial beta diversity only in synthetic roots with high anatomical complexity (Extended Data Fig. 9i). In this line, we found that the number of ASVs showing significant changes in their relative abundance compared to the control condition (without root exudates) was highest in synthetic roots with high anatomical complexity (Extended Data Fig. 9j)".

Reviewer #1 comment 40
40- l. 272: looking at the statistics given in figure S8k, there is no significant correlation, the statement here appears incorrect (as elsewhere, it would be convenient to have information about the type of correlation as well as of p and R-values given in the text).

Authors response: Thank you for the comment. Following the reviewer's recommendation, we checked the statistics in Figure S8k (now Fig. S9k). We found that there was a typographical error in the figure: the values for R^2 and P were inadvertently inverted. The P-value was actually the R^2 value, and vice versa. This has now been corrected, and the correct values are reflected in the updated text.

Reviewer #1 comment 41
41- l. 264-275: taken together the results presented in this section, the data are not very convincing to support the conclusion. Alpha diversity does not show a clear consistent trend depending on CCL (Fig. S8h), it would require further proof to confirm this. Beta diversity appears to be only affected in high complexity roots, but in the PCoA plot there is no convincing evidence that CCL plays a role (Fig. S8i). The correlation visualized in Fig. S8k seems not to be significant.

Authors response: After clarifying and improving our analysis based on the reviewer's recommendations and comments, we believe that our data and figures now better support our conclusions. We hope the reviewer agrees with this assessment.
Please see our response to comments 38–40.

Reviewer #1 comment 42
42- l. 279: move comma behind reference to Fig. 1

Authors response: Fixed

Reviewer #1 comment 43
43- l. 283: reference to Table S4 appears incorrect here

Authors response: All tables have been reconfigured and the references updated.

Reviewer #1 comment 44
44- l. 284: what does "nutrient limited" mean here? 1/100 or 1/1000 HS?

Authors response: Nutrient limited condition means 1/1000 SH. This information has been added to the text.

Reviewer #1 comment 45
45- l. 289: table S4 is not a meaningful here

Authors response: Fixed. The reference to Table S4 has been removed from the text.

Reviewer #1 comment 46
46- l. 284-289 and Fig. 3b: does the clustering seen in Fig. 3b support the grouping of correlated variables into three groups? Can you specify what criteria or metrics were used to define the three groups in the clustering?
Are there any statistical measures (e.g., silhouette score, dendrogram height) that confirm the robustness of these three clusters?
Did you consider alternative numbers of clusters, and how did the three-group solution compare?
Would you suggest any additional analyses to validate or refine the grouping?

Authors response: Thank you for pointing this out. To determine the number of clusters, we used the 'Elbow method for k-means clustering'. This analysis indicated that 3 clusters represent the most appropriate number for this dataset. The results of this analysis are shown below:

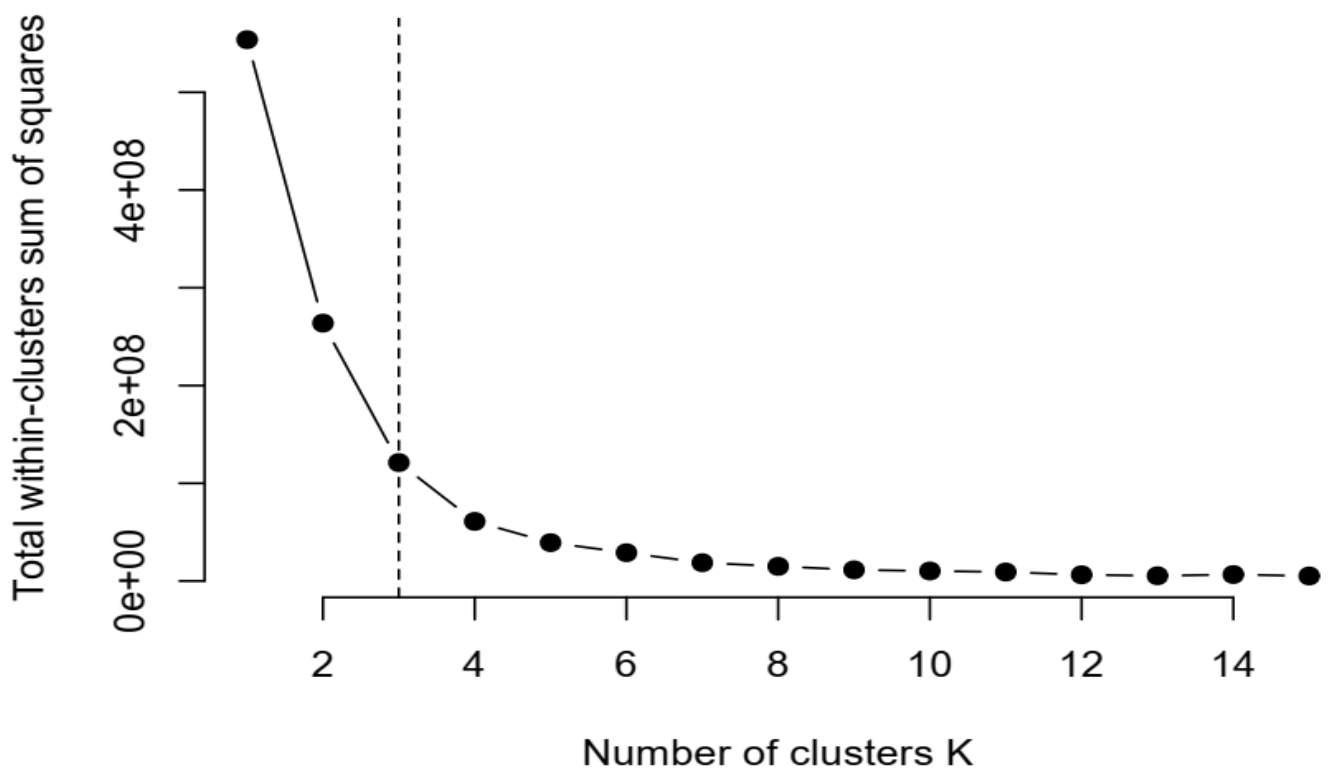

Reviewer #1 comment 47
47- l. 288, 293: "number of aerenchyma" appears to lack information, aerenchyma does not appear to be a countable unit.

Authors response: The term "number of aerenchyma" has been replaced with "amount of aerenchyma-like structures," which more accurately reflects the quantity of aerenchyma-like structures present in a given root. This terminology is commonly used in the literature (https://doi.org/10.1016/j.envexpbot.2025.106207
, https://doi.org/10.1104/pp.17.01157) to describe an anatomical trait characterised by a large air-filled spaces or cavities (aerenchyma) typically found in aquatic and wetland plants.

Reviewer #1 comment 48
48- l. 284-307: I am lost here; the authors state that they focus in their analyses on three specific root traits (l.288-289), but the subsequent reporting of results does not focus on these three traits, nor do the figures in Fig. S9.

Authors response: I kindly disagree with the reviewer on this comment. We focused on these three highly responsive traits for the interpretation and analyses of the drop-out and mono-association experiments. In the text, we specifically mentioned this: "We found that anatomical features such as the index of root complexity, amount of aerenchyma-like structures, and number of cells in the third cortical cell layer were highly responsive to the synthetic communities (Fig. 3b). We observed that the three bacterial synthetic communities dropping out the highly abundant ASVs corresponding to the bacterial strains A289 (*Allorhizobium-Neorhizobium-Pararhizobium-Rhizobium*), A245 (*Brevundimonas*), and P65 (*Staphylococcus*) significantly modified the highly responsive parameter index of root complexity compared to plants inoculated with the full synthetic community (Fig. 3c and Extended Data Fig. 10a). Similarly, the amount of aerenchyma-like structures changed significantly in response to the synthetic community that eliminated the ASV corresponding to the bacterial strain P128A.22 (*Paenibacillus*) (Fig. 3c). These results indicate that microbiota-driven root plasticity in plants with inherent complex roots

depends on specific bacterial strains. We repeated this experiment using *Lemna aequinoctalis* 7339, which has a less anatomically inherent complex root. We confirmed differences in root anatomical trait variations in response to the full and drop-out bacterial synthetic communities between *Lemmna aequinoctalis* 7339 and *Spirodela polyrhiza* 9309. Indeed, we identified a different set of highly variable anatomical features (amount of aerenchyma-like structures, aerenchyma-like mean area, number of cells in the endodermis, and number of cells in the external cortical cell layer 2) in *Lemmna aequinoctalis* 7339 in response to the synthetic communities (Extended Data Fig. 10b)"
For the rest of the analyses, we have also focused on these traits whenever possible. To make this more evident, we have highlighted these parameters in orange in Figure 3 and Extended Data Fig. 10.

Reviewer #1 comment 49
49- l. 291: I would find it valuable to get the genus and species names for these three taxa; they play a major role in the following.

Authors response: Following the reviewer's recommendation, we have added the genus names for the three taxa that significantly modified the highly responsive parameter index of root complexity: A289 (*Allorhizobium–Neorhizobium–Pararhizobium–Rhizobium*), A245 (*Brevundimonas*), and P65 (*Staphylococcus*). We also mapped the *16S rRNA* gene sequences of the isolates against the NCBI database and were able to identify the species for some of them: A289 (*Rhizobium daejeonense*), A245 (*Brevundimonas spp*.), and P65 (*Staphylococcus epidermidis*). However, since *16S rRNA* gene sequences are generally not sufficient to confidently assign bacterial strains to the species level, we decided not to include this information in the manuscript and to retain only the genus-level identification.

Reviewer #1 comment 50
50- l. 344-346: is this correlation equally good in axenic plants and plants with Syncom?

Authors response: We thank the reviewer for this comment. We have separated the samples into microbiota-colonized and axenic plants to perform the correlation analysis. We found that the metabolic complexity of the roots was significantly negatively correlated ($R^2 = 0.4474$, $p = 0.0013$) with the number of root cell layers only in plants colonized by microbes. This information has been added to the text (Fig3 b).

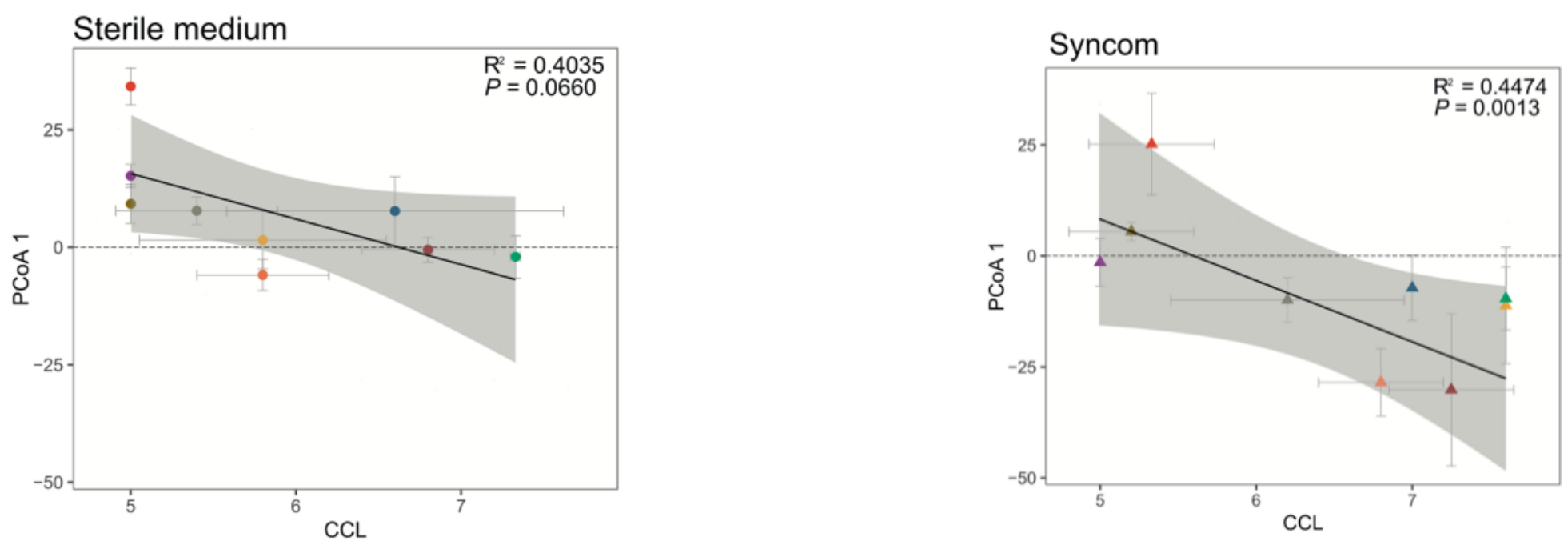

Reviewer #1 comment 51
51- l. 358-360: Why is trimethyl-lysine highlighted here and subsequently in the focus of this study? Figure 4c lacks important information to understand this (see comments below) and Fig. S12a suggests that other components showed even stronger inhibitory effects than this one across the tested plant species. Further, Fig. S12b does not indicate a survival problem related to this compound, compounds such as glutaric acid appear to be much more detrimental. Where does growth reduction end and toxicity begin? Why not going for pyrone, which reduces growth as well? The reason for a focus on trimethyl-lysine as key component requires more convincing justification or better explanation of the results.

Authors response: We thank the reviewer for this comment. For the selection of compounds screened in this work, we originally applied criteria based on a combination of the confidence level used to assign identities to the putative metabolites and their effects on the analysed plant phenotypes. Although some putative metabolites showed clear effects on plants, as noted by the reviewer, they were not selected

for further analyses because the confidence level of their identification was not sufficiently high. We agree with the reviewer that this rationale was not clearly explained in the original manuscript.
In the revised version of the manuscript, we applied stricter selection criteria and screened only metabolites identified with confidence level ID2 (putative annotation via spectral library match, no standard) and ID1 (confirmed identification with a reference standard, matching retention time and MS/MS spectra). This reduced the list to two compounds: L-citrulline (ID2) and N^6,N^6,N^6-Trimethyl-L-lysine (ID1). After analysing their effect on the phenotypes of interest, only N^6,N^6,N^6-Trimethyl-L-lysine showed a clear effect on plant growth and reproduction. Therefore, this compound was selected for further analysis.
We have better explained this selection in the revised version of the manuscript and removed irrelevant information that made the interpretation of our results difficult. The text now reads as follows:
"We evaluated the effects of the two putative metabolites (L-citrulline and N^6,N^6,N^6-Trimethyl-L-lysine), whose identities were assigned with confidence level ID2 (Table S6) and which were commercially available, on the growth kinetic of six Lemnaceae species, with different inherent root complexities, inoculated with the bacterial synthetic community under low-nutrient conditions (1/1000 SH) (Fig. 4c and Extended Data Fig. 13a). We observed that the exogenous addition of N^6,N^6,N^6-Trimethyl-L-lysine significantly reduced the total area of plant fronds, that we used as a proxy for plant growth (Fig. 4c and Extended Data Fig. 13a). In contrast, we did not observe a significant effect of L-citrulline on the growth of the different plant analysed (Fig. 4c and Extended Data Fig. 13a) and therefore focused exclusively on the characterisation of N^6,N^6,N^6-trimethyl-L-lysine. First, we confirmed the N^6,N^6,N^6-trimethyl-L-lysine identity with a confidence level ID1 using a reference standard (Extended Data Fig. 13b). Then, we exposed *Spirodela polyrhiza* 9309, with an inherent complex root, to increasing concentrations of N^6,N^6,N^6-Trimethyl-L-lysine in the presence of the microbiota under low nutrient conditions"

We have included the confidence index in a new Table S6. We have also removed irrelevant information from Fig 4 and Extended Data Fig. 13. We believe that this section of the manuscript has been significantly improved, and we thank the reviewer for pointing this out.

Reviewer #1 comment 52
52- l. 364: The reference to Fig. S12c does not appear meaningful and I do not understand the relevance of Fig. S12c.

Authors response: Figure S12C (New Fig S13b) provides confirmation, using a certified standard, that the compound identified in our root metabolomic analyses is indeed N^6,N^6,N^6-trimethyl-L-lysine. This result is critical for eliminating any doubt regarding the compound's identity. Therefore, we can state that this compound has been identified with confidence level ID1 (confirmed identification with a reference standard, matching retention time and MS/MS spectra). This confirmation is essential for the validation of our findings.

Reviewer #1 comment 53
53- l. 368-371: maybe I get tired, but I do not understand how this statement is supported by Fig S12d and e.

Authors response: We thank the reviewer for this comment. The aim of this experiment was to demonstrate that the microbiota-driven root anatomical plasticity observed in *Spirodela polyrhiza* 9509, which has a complex root anatomy, is compromised when roots are treated with high concentrations of N^6,N^6,N^6-Trimethyl-L-lysine. As controls, we used plants treated with the synthetic community, representing maximal root plasticity, and axenic plants (no-bacterial control), representing minimal root anatomical plasticity, as this process is microbiota-driven.
Based on this rationale, we included three additional treatments consisting of plants treated with the synthetic community in combination with three concentrations of N^6,N^6,N^6-Trimethyl-L-lysine (25, 50, and 75 uM). Anatomical traits were quantified for all treatments.
Principal coordinates analysis showed that the anatomical traits of plants treated with the synthetic community alone and those treated with a low concentration of the compound were highly similar (New Fig. S13c). Consistent with this result, the heatmap shown in Figure S13d, where all samples were compared against the synthetic community alone treatment, revealed that only 1 out of 21 traits differed significantly between these two treatments (Fig. S13d). This indicates that low concentrations of N^6,N^6,N^6-Trimethyl-L-lysine do not interfere with the microbiota-driven root anatomical plasticity.
In contrast, at higher concentrations of N^6,N^6,N^6-Trimethyl-L-lysine, anatomical traits diverged from those observed in the synthetic community treatment and became more similar to the no-bacteria

control (Fig. S13c-e). To clarify this conclusion, we added an additional panel (New Fig. 13e) showing correlations between the no-bacteria control or the full bacterial synthetic community treatment and the N^6,N^6,N^6-Trimethyl-L-lysine treatments. We have added this information to the new version of the manuscript: "These conclusions were more evident when we correlated individual root anatomical features measured in plants treated with different concentrations of N^6,N^6,N^6-Trimethyl-L-lysine with those obtained from axenic roots or from roots inoculated with the bacterial synthetic community. We found that roots treated with a low concentration of N^6,N^6,N^6-Trimethyl-L-lysine (25 μM) exhibited the highest number of anatomical features that significantly correlated with those observed in roots inoculated with the bacterial synthetic community treatment (Extended Data Fig. 13e). In contrast, higher doses of this compound (50 and 75 μM) compromised root plasticity in response to the microbiota, resulting in plants whose key anatomical traits showed reduced correlation with those of microbiota-inoculated roots and increased similarity to those measured in axenic plants (Extended Data Fig. 13e)".

Reviewer #1 comment 54

54- l. 373-374: similar problem here with Fig. 4e: it is said that the trimethyl lysine treated plants assembled an atypical root microbiota, but this cannot be seen in Fig. 4e, because the treated and non-treated plants are shown in different plots, so I cannot assess differences in the communities between these plants. Further, Fig. S13a and b do not show any results for trimethyl lysine as far as I can see.

Authors response: We thank the reviewer for pointing this out. Following the reviewer's comment, we prepared a new panel plotting treated and untreated plant data together (New Fig.4e). Differences in microbiota membership between samples are explained by the variables "root complexity measured as the number of root cell layer (CCL)", "metabolite" treatment, and their interaction. We can therefore conclude that TML treatment affects the assembly of the root microbiota in plants with inherently high root complexity. We have revised the text accordingly:

"We also investigated the effect of supplementing with 50 μM N^6,N^6,N^6-Trimethyl-L-lysine on plant microbiota structure in four *Lemnaceae* species with medium to high inherent anatomical root complexity (*Lemna japonica* 9250, *Landoltia punctata* 0049, *Spirodela polyrhiza* 7498, and *Spirodela polyrhiza* 9509), all inoculated with the full bacterial synthetic community. In line with its effect on anatomical plasticity (Extended Data Fig. 13c-e), we observed that beta-diversity was explained by the variables "number of root cell layers (CCL)", a proxy for root complexity, "metabolite", and their interaction (Fig. 4e and Extended Data Fig. 14a)".

We have improved the legend of the new Supp Fig. 14a (before Fig. S13a) to make the data about N^6,N^6,N^6-Trimethyl-L-lysine more obvious, now it reads:

"Heatmap showing the fold change in relative abundance of the most abundant ASVs in the roots of four *Lemnaceae* species with medium to high root complexities (SP9509: *Spirodela polyrhiza* 9509; SP7498: *Spirodela polyrhiza* 7498; LP0049: *Landoltia punctate* 0049; LJ9250: *Lemna japonica* 9250), grown in 1/1000 SH medium inoculated with the full bacterial synthetic community, and in plants additionally supplemented with the modulatory metabolite N^6,N^6,N^6-Trimethyl-L-lysine. Data were grouped by ASVs. The dendrogram illustrates the hierarchical clustering of ASVs, with the scale bar indicating the distance between identified clusters. Statistically significant enriched (red) and depleted (blue) root trait values relative to plants exposed to the bacterial synthetic community are outlined in black (q < 0.1) (n = 6)".

Reviewer #1 comment 55

55- l. 375-377: it would be helpful to report the R2-values of the permanova to evaluate the disappearance of differences, because the p-values with trimethyl-lysine are only hardly non-significant in Fig. 4e. Fig. 13a and b are again not useful to support this statement.

Authors response: In the new panel Fig. 4e we have included the R2-values of the PERMANOVA and its corresponding p-values (complexity R2=0.51, p=0.001; metabolite R2=0.05, p=0.009). Supporting the effect of TML on root microbiota composition, we obtained similar results in experiments using synthetic roots (Fig. 4f). Consistent with the findings in real roots, we observed that N^6,N^6,N^6-Trimethyl-L-lysine modulates microbiota assembly only in synthetic roots with high anatomical complexity (R^2 = 0.387, p = 0.002) that were inoculated with the bacterial synthetic community and exposed to two distinct root exudates extracted from *Spirodela polyrhiza* 9509 and *Lemna japonica* 9250 (Fig. 4f and Extended Data Fig. 14b). No significant metabolite effect on microbiota profiles was detected in

synthetic roots with low or medium anatomical complexities under the same conditions (Fig. 4f and Extended Data Fig. 14b).
Figure S14a-b (previously S13a-b), prepared using the same dataset, also help to visualise the effect of N^6,N^6,N^6-Trimethyl-L-lysine on the assembly of the root microbiota.

Reviewer #1 comment 56
56- l. 378: what does "high concentration" mean in this context? How much was applied, also to compare with Fig. 4d? Info could be added to figure legend (Fig. 4e).

Authors response: We thank the reviewer for this comment. In this context, "high concentration" refers to the external supplementation of N^6-N^6-N^6-Tri-Methyl Lysine (50 uM), which could increase the level of this compound within the roots of plant species with complex root systems, where it normally occurs at low concentrations. This externally induced high concentration could negatively affect root anatomical plasticity in these species. To avoid confusion, we have replaced the term "high concentration" with "external supplementation," and the text now reads: "This indicates that external supplementation of this compound in roots with inherently complex anatomical roots, where it is normally found at low concentrations, could increase its endogenous levels and modify the composition of the root microbiota, thereby impacting the anatomical plasticity of the root". In figure 4d we used three different concentrations of this metabolite 25, 50, 75 uM. For the experiment discussed here we used 50 uM, a concentration sufficient to induce significant changes in root anatomy, plant reproduction, and survival, and root microbiota profiles in plant with complex roots. Information regarding N^6-N^6-N^6-Tri-Methyl Lysine concentrations has been added to the Fig. 4e legend.

Reviewer #1 comment 57
57- l. 381: what does between-sample variation mean here? And how does Fig. S13c support the statement in l. 380-384? (applies also to Fig 4f). It is very hard to follow the argumentation here and see it supported by the figures.

Authors response: We have revised this part of the manuscript in response to this and previous reviewer's comments to better support our statements regarding the modulatory effect of N^6-N^6-N^6-Tri-Methyl Lysine on plant root anatomy and its consequences for microbiota assembly. To avoid confusion, we have removed the term "between-sample variation", which we had previously used as a substitute for beta-diversity. Our argument supporting the hypothesis related to N^6-N^6-N^6-Tri-Methyl Lysine now includes an analysis of its effect on microbiota structure in both real and synthetic roots. "We also investigated the effect of supplementing with 50 μM N^6,N^6,N^6-Trimethyl-L-lysine on plant microbiota structure in four *Lemnaceae* species with medium to high inherent anatomical root complexity (*Lemna japonica* 9250, *Landoltia punctata* 0049, *Spirodela polyrhiza* 7498, and *Spirodela polyrhiza* 9509), all inoculated with the full bacterial synthetic community. In line with its effect on anatomical plasticity (Extended Data Fig. 13c-e), we observed that beta-diversity was explained by the variables "number of root cell layers (CCL)", a proxy for root complexity, "metabolite", and their interaction (Fig. 4e and Extended Data Fig. 14a). These results indicate that external supplementation of this compound in plants with inherently complex anatomical roots, where it is normally present at lower concentrations, could increase endogenous levels and alter root microbiota composition, thereby impacting the anatomical plasticity of the root. Consistent with these findings, we observed that N^6,N^6,N^6-Trimethyl-L-lysine modulates microbiota assembly only in "synthetic roots" with high anatomical complexity (R^2 = 0.387, p = 0.002) that were inoculated with the bacterial synthetic community and exposed to two distinct root exudates extracted from *Spirodela polyrhiza* 9509 and *Lemna japonica* 9250 (Fig. 4f and Extended Data Fig. 14b). No significant metabolite effect on microbiota profiles was detected in "synthetic roots" with low or medium anatomical complexities under the same conditions (Fig. 4f and Extended Data Fig. 14b). In this system, which lacks a plant immune component, changes in microbiota assembly induced by N^6,N^6,N^6-Trimethyl-L-lysine can be attributed either to its direct effects on bacterial growth or to emergent properties arising from interactions between root anatomical/metabolic complexity and metabolite concentration. Indeed, it promoted the growth of certain individual members of the full bacterial synthetic community in vitro (Extended Data Fig. 14c). Overall, these results indicate that N^6,N^6,N^6-Trimethyl-L-lysine mediates root anatomical plasticity responses associated with microbial colonisation".

We hope the reviewer can now better appreciate how our different experiments converge on the same conclusion, supporting the role of N^6,N^6,N^6-Trimethyl-L-lysine in controlling microbiota-driven root plasticity.

Reviewer #1 comment 58
58- Figures:
The text in nearly all figures is too small and not readable. In several cases, even enlargement of the figures does not allow to read text due to poor resolution. This applies also to figures in the supplementary material.

Authors response: Thank you for the comments. We have increased the text size in all figures and improved their overall quality.

Reviewer #1 comment 59
59- Figure 1a: what does PS stand for? Please add the similarity measure and tree reconstruction algorithm. What does the RPL16 gene encode? Why only 18 plant species/accessions shown and not 19?

Authors response: PS stands for *Pistia stratiotes*, another member of the *Araceae* family which shares a degree of morphological similarity with the *Lemnaceae* but has a more complex root anatomy including features like branching. RPL16 refers to a gene encoding chloroplast *RPL16* gene sequences, encoding the L16 Ribosomal Protein, a component of the large (50S) ribosomal subunit in chloroplasts. Because RPL16 is present in almost all organisms and changes little over time, it serves as a reliable molecular marker to build phylogenetic trees and study evolutionary divergence among species or groups. We have prepared a new tree (new Fig. 1a) where all the 19 species used are now included. Information about the *RPL16* gene has been included in the figure legends.

Reviewer #1 comment 60
60- l. 482: gene, not gen

Authors response: Fixed

Reviewer #1 comment 61
61- l. 486: It should probably be “water” here, not “waters”

Authors response: Fixed

Reviewer #1 comment 62
62- l. 489: difference, not different

Authors response: Fixed

Reviewer #1 comment 63
63- Fig. 1d: it is very hard to see if species:CCL is significant or not; what do the error bars show, SD or SE? What method was used to assess the explained variance?

Authors response: We apologise for the use of red in the figure. To improve its visualisation, we have highlighted the significant variables in orange to provide a clearer contrast with the other colours. The error bars represent standard deviations (SD), and the method used to assess the explained variance was a Permutational Multivariate Analysis of Variance (PERMANOVA). This information has been added to the figure legend.

Reviewer #1 comment 64
64- Fig. 1f: specify type of correlation in figure legend

Authors response: We used a linear correlation or a linear regression in the figure. This information was added to the figure legend.

Reviewer #1 comment 65

65- Fig. 2b: a scale bar would be helpful in the SEM images to allow an estimation of cavity sizes and better compare these structures with those of real roots, e.g. the images in Fig. S1g, which actually also lack scale bars (or the size of the black bars, which may be scales).

Authors response: Scale bars are now visible in Fig. 2b, and they have also been added to the new supplementary Fig. S2.

Reviewer #1 comment 66
66- l. 522, 528: If I got it correctly, these incubations were done with diluted SH medium; please specify this in the text here.

Authors response: It is specified in the text now: "To emulate the wide range of nutrient availability that may be present in the natural water experiment, resulting from the different species used and their interaction with the microbiota, we grew these plant species at three nutrient concentrations using different dilutions of SH medium (1/2 SH, 1/100 SH, and 1/1000 SH) with or without inoculation with a 190-members bacterial synthetic community comprising 75.5% of the collection strains.

Reviewer #1 comment 67
67- l. 524: "con"?

Authors response: Fixed

Reviewer #1 comment 68
68- Fig. 2d: how many images were analysed here?

Authors response: We thank the reviewer for this comment. We took 3 images at two different microscopic magnifications for each treatment and plant species. This information has been added to the legend of Fig. 2d.

Reviewer #1 comment 69
69- Fig. 3a: not readable at all. Don't understand the color codes; the taxonomic information that can be extracted appears of little value for the understanding of the manuscript. Genus- and species-names would be more informative in such a figure, but even with this information, this figure does not appear relevant for the further understanding; the authors do not discuss taxonomy effects.

Authors response: We thank the reviewer for this comment. We have increased the size of the legends and labels in the figure to improve readability. This figure shows the composition of the 20 most abundant ASVs colonising *Lemnaceae* roots. In the figure, the tree tips are coloured according to bacterial taxonomic phyla, and the strains sharing the same colour shade belong to the same ASV. This figure is relevant for understanding the bacterial composition of the twenty bacterial synthetic communities we prepared by dropping out each of the top 20 most abundant ASVs. Without this information the interpretation and transparency of the manuscript will be compromised.

Reviewer #1 comment 70
70- Fig. 4b and c: x-and y-axis labelling have text formatting problems

Authors response: Fixed

Reviewer #1 comment 71
71- Fig. 4c: is a control treatment available without added metabolite that could be added here for comparison? Were these data obtained after 7 days of growth? And which plant species was used to generate these data here?

Authors response: Frond area values obtained from plants grown without metabolite treatment (control) were added to the figure. The data represent the average frond area after 7 days of growth for the following species: LJ9250: *Lemna japonica* 9250; LP0049: *Landoltia punctata* 0049; LP8539: *Lemna perpusilla* 8539; LY9208: *Lemna yungensis* 9208; SP7498: *Spirodela polyrhiza* 7498; SP9309: *Spirodela polyrhiza* 9309. This information has been added to the figure legend.

Reviewer #1 comment 72

72- Fig. 4d: x-axis labelling should read percentage, not percentage.

Authors response: It is corrected now. Thanks.

Reviewer #1 comment 73
73- Fig. 4e: the color code is not explained (red/blue) and it remains unclear why the plot on the right includes only four of the six plant species. Difficult to draw conclusions without understanding these details.

Authors response: Following the reviewer's comments, we have significantly revised this figure to improve clarity and data visualisation. It now includes only four plant species with medium to high inherent root complexity. The new figure features clear legends and labels.

Reviewer #1 comment 74
74- Supplementary material:
Page numbers would be very helpful for such an extensive document and the file size should be reduced. It slowed down Word substantially, especially when looking at the plots on page 50.

Authors response: Page numbers have been added to the document. However, we respectfully disagree with reducing the document size, as doing so could compromise the quality, transparency and reproducibility of the experiments presented here.

Reviewer #1 comment 75
75- Supplementary methods:
The methods section should be better structured, following the structuring in the results section.

Authors response: The methods section has been significantly reorganised and is now structured into five distinct sections: **1.** Experiments using natural water. **2.** Experiments using bacterial synthetic communities. **3.** Experiments using synthetic roots and root exudates. **4.** Drop-out and mono-association experiments using Lemnaceae species and bacterial synthetic communities. **5.** Identification of modulatory metabolites relevant for root plasticity in response to the microbiota. These main sections follow the order of the experiments presented in the main manuscript to facilitate the reader's experience. Within each section, detailed explanations of the experiments and analyses performed are provided.
In the revised version, redundancies in the Methods have been removed, resulting in a shorter and clearer text.

Reviewer #1 comment 76
76- Some methods appear to be described redundantly such as DNA extraction in section 2.9 or data analyses procedures, which are repeatedly described for different datasets. Also, plant growth conditions are described repeatedly. I did not check but would guess that it is always the same.

Authors response: We acknowledge that our supplemental material document is detailed and extensive with experiments and analyses explained thoroughly. This was done intentionally to increase the transparency and reproducibility of our experiments. Following the reviewer's comment, in the revised version of this document, redundancies in the methods have been removed, resulting in a shorter and clearer text.

Reviewer #1 comment 77
77- I miss information about sequencing depth and minimum read numbers or defined for rarefaction. This is not systematically given for all data sets, but important information to evaluate the quality of the presented results. The 500 reads mentioned raise questions about the robustness.

Authors response: Thank you for pointing this out. We agree that insufficient read depth can bias diversity estimates and should be carefully evaluated. Samples were rarefied to 500 reads to standardize sequencing depth across samples and maximize sample retention. This depth was sufficient to capture overall community patterns based on rarefaction analysis. As a result of this rarefaction threshold, samples with approximately 500 reads represent the lowest sequencing depth in the dataset; however, they do not constitute statistical outliers relative to the overall distribution. All other samples had substantially higher read numbers, ranging from 567 and 139838 reads (mean:

34,310.23 ± SE: 1,590.63), indicating that the majority of samples were sequenced at sufficient depth. Using this threshold, we retained 278 out of 288 samples for downstream analyses. As a proof of concept for the robustness of the dataset, we examined whether there was an association between alpha diversity and read number. We found no association between read number and alpha diversity. To further assess whether sequencing depth adequately captured microbial alpha diversity, we examined rarefaction curves, which approached asymptotes for all samples, including those with lower read counts. This suggests that alpha-diversity estimates are not driven by sequencing depth in this dataset, indicating that our dataset is robust.

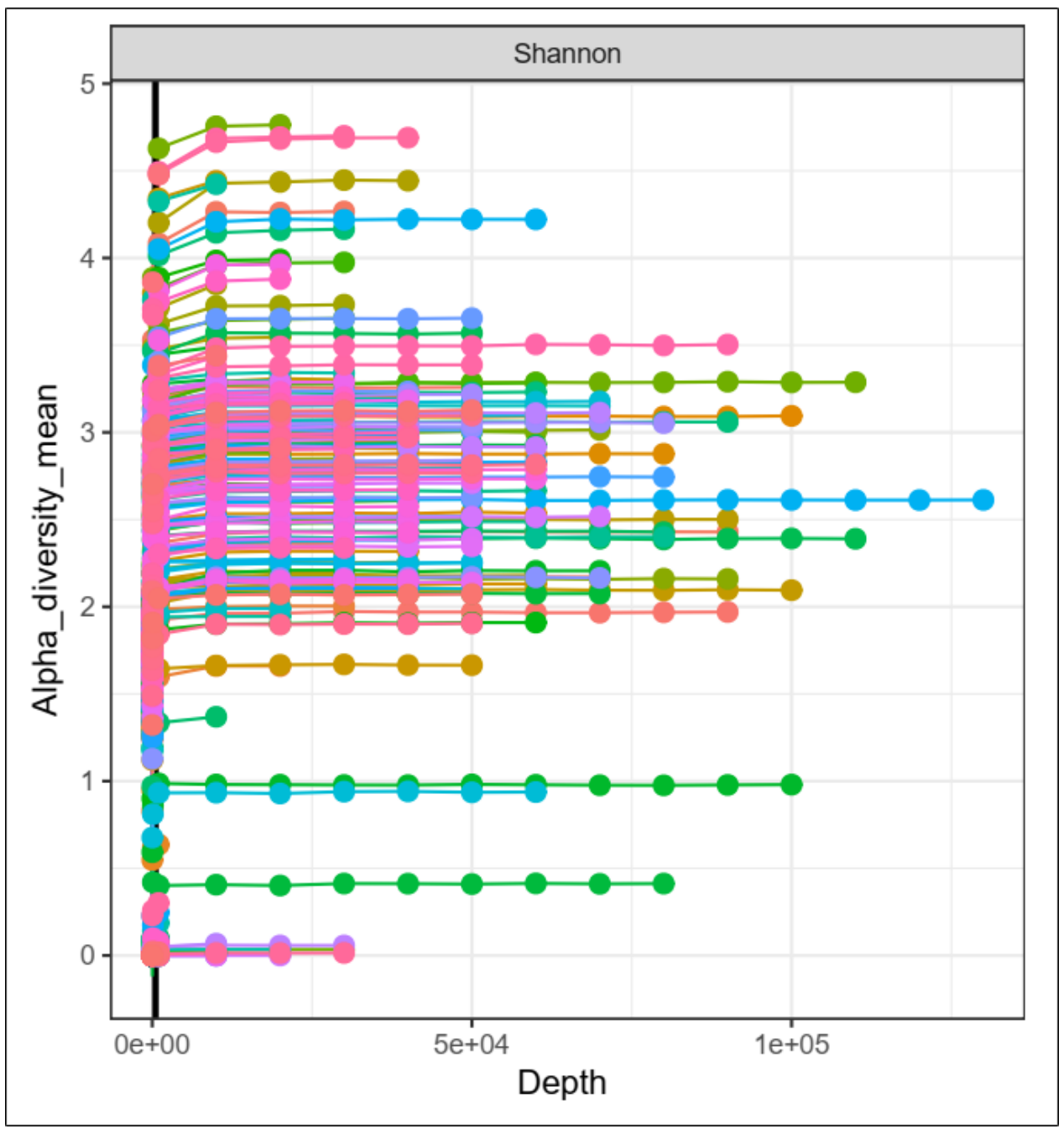

Reviewer #1 comment 78

78- 1.3: were all plants cultivated in the same way before transferring them to the flasks for the 21-d incubation experiment? Can we exclude that some plants came in with a different microbiome than others due to cultivation conditions used before?

Authors response: Prior to the experiment, all plants were surface sterilized with 10% bleach, washed with 2% sodium thiosulfate to neutralize the bleach, and then rinsed at least three times with sterile water. All plants were cultured in flasks containing ½-strength Schenk and Hildebrandt (SH) medium. To verify sterility, samples from all plants were collected, homogenized using magnesium chloride, and plated on LB and YPD media. In all cases, no microbial growth was detected. This information has been added to the Materials and Methods section.

Reviewer #1 comment 79

79- 1.3: Conviron is a company, what is the instrument? Can you add information about the light intensity?

Authors response: Thanks for the comment. Plants were grown in a Conviron growth chamber at 120 μmol m^{-2} s^{-1} light. This information has been added to the materials and methods section: “All flasks were manually randomised and placed in a Conviron growth chamber, and their positions were re-randomised every two days to minimise positional effects. Plants were grown for 21 days at 22 °C, 70% relative humidity, under a light intensity of 120 μmol m^{-2} s^{-1} with a 16 h darkness/8 h light photoperiod”.

Reviewer #1 comment 80

80- 1.6: I assume the 1.5 ml Eppendorf tubes with the samples were put in the plastic bag? Please specify accordingly, I got confused here.

Authors response: This information has been clarified in the materials and methods section. It now reads: “Prior to extraction, all tubes containing the samples, including those containing water, were

randomised by placing them in a plastic bag and shaking thoroughly. Tubes with the samples were then removed individually from the bag and placed into the DNA extraction plates. This randomization was maintained throughout library preparation and sequencing".

Reviewer #1 comment 81
81- 1.7: the Lundberg reference lacks information about the year; please give nucleotide concentrations and polymerase in U, not only volumes; in the second PCR, what was the assay composition and was a PNA-specific step indeed required here?

Authors response: The information about the references and reagent concentrations has been added to the Materials and Methods section. The second PCR reaction is described in the Methods and primarily consists of the primers and Kapa HiFi HotStart ReadyMix, following the manufacturer's recommendations. Although the PNAs used are not completely universal, they are "broad-spectrum" and, with varying efficiencies, can work across many plant species if the target rRNA region is conserved. In our case, the match was not perfect but still significantly reduced the amplification of the 16S rRNA genes in the plant tissues.

Reviewer #1 comment 82
82- 2.2: please note, 16S rRNA gene sequencing does not allow to identify redundant strains reliably, because different strains of the same species can have identical 16S rRNA gene sequences; phrasing is not precise here

Authors response: We agree with the reviewer. To improve the text, we have removed the sentence related to redundancy. The revised text now reads: "To assign taxonomy to the bacterial cultures, we PCR-amplified and Sanger sequenced the *16S rRNA* gene from each individual bacterial strain".

Reviewer #1 comment 83
83- 2.2: as before, volumes of primer solutions are not informative

Authors response: Primer concentrations have been added to the methods.

Reviewer #1 comment 84
84- 2.3: considering that 10 different media were used for enrichment and isolation, I am surprised to see that all isolates were cultured here on LB medium; did the authors validate adequate growth of the individual strains?

Authors response: Once bacterial strains have been isolated in pure culture, it is not surprising that the majority grow on LB medium. In our case, all selected bacteria used in the synthetic community experiments were able to grow on LB medium. LB is a commonly used, nutrient-rich medium that supports the growth of many bacterial species from diverse collections. For example, bacterial isolates obtained from *Arabidopsis thaliana* (doi: 10.1038/s41588-017-0012-9; 10.1038/nature16192) have been successfully cultured as pure strains on LB medium.

Reviewer #1 comment 85
85- Supplementary figures:
The text in the figure legends has language mistakes. As there are no line numbers given, but a lot of text, I do not point them out but ask for systematic proof-reading.

Authors response: Thank you for pointing this out. All figure legends have been revised and corrected.

Reviewer #1 comment 86
86- I miss detailed information that explains all elements in the figures, e.g. about statistical tests that were applied or number of replicates. Only later, I found this information in the supplementary methods section. The authors should consider adding this info to the figures directly.

Authors response: Thank you for pointing this out. The statistical methods used and the number of replicas in each case have been added to the figure legends.

Reviewer #1 comment 87

87- Similarly, several figures show dendrograms, but information is missing regarding their reconstruction, i.e. what kind of dissimilarity matrix was calculated and based on which algorithm was a dendrogram calculated.

Authors response: We have added more information to the figure legends. Unfortunately, we are unable to address this specific comment due to the lack of detail provided. We do not know which figure panels the comment refers to.

Reviewer #1 comment 88
88- Fig. 1c: I do not understand what the vertical data represent. I do get that this is the water treatment and the 19 different plant species, but the plot includes many more samples. What are these?

Authors response: In the previous Fig. S1C, we presented all individual plants (roots) analysed. To avoid confusion, we have reorganized Fig. S1C to improve data visualization and to make our conclusions clearer. Instead of displaying each replicate individually as before, we combined all replicates from the same microbial treatment (grown in sterile water or in natural water containing the microbiota) and genotype (colours) and used the resulting average values to generate a new Fig. S1C panel. In this revised panel, it is easier to observe, for example, that the values obtained for anatomical traits such as CCL2 diameter, CCL2 cell number, CCLe2 cell number, CCLe1 cell number, aerenchyma-like mean area, CCL, number of roots, CCL3 diameter, and CCL3 cell number, are higher in plants with a greater number of roots**.** We also clarified the legend and the labels used.

Reviewer #1 comment 89
89- Fig 1e: It is said here that 21 root traits were identified in panel c that were used to run the PCoA. However, Fig. 1c shows only 21 traits displayed, so there was no selection of traits beyond what is shown in Fig. 1 c and d?! (though Fig. 1d shows only 20 traits as far as I can see).

Authors response: We believe the reviewer is referring to Supplementary Figure 1. We have corrected this typo in the figure legend, which now refers to the 21 traits quantified in panel C.

Reviewer #1 comment 90
90- Figure 1e: it appears that the CCL numbers are color-coded by a continuous color gradient, this would not be meaningful if CCL numbers are integer numbers. Actually, it is unclear if the CCL numbers of individual plants are already mean values of multiple counts. If not, the color code should be adapted accordingly. Please ensure that also other plots have appropriate color gradients, CCL is quite often color-coded.

Authors response: We consider that the CCL legend used in this panel serves only as an indicative measure of root complexity to facilitate data interpretation. We would like to clarify that, since several plants were used in our analysis and not all of them exhibited exactly the same number of cortical cell layers in response to the microbiota, the resulting average number of cortical cell layers is not always an integer. In some cases, the values are fractional. Therefore, we believe that the current scale is accurate as presented. The CCL values were generated using a minimum of five roots per plant species.

Reviewer #1 comment 91
91- Fig. 1f: It is said that the data were clustered according to plant species. I assume the authors mean the sorting of species, illustrated by the dendrogram. This dendrogram is not explained in the legend, does it reflect phylogeny and based on which algorithms was it constructed? However, as the authors emphasize the decreasing number of significantly differential features for plants with less complex roots, I recommend a plant species sorting that reflects root complexity instead of phylogeny.

Authors response: We thank the reviewer for this comment. Following the reviewer's recommendation, we arranged the plant species according to root complexity in the revised Fig. S1f. Consequently, the dendrogram on the left was removed. We also improved the legend and panel labels. We believe these changes enhance the clarity and visualisation of the data.

Reviewer #1 comment 92
92- Fig. 1g: this figure is not mentioned in the text, as far as I can see.

Authors response: In the revised version of the manuscript, we removed Fig. S1g and added a new Fig. S2 containing anatomical images for all plant species used in this study.

Reviewer #1 comment 93
93- Fig 2b: I do not understand what "number of aerenchyma" means? And what has caused the authors to present these root traits from the 21 that were determined and are presented in Fig. S1? Are these the ones with significant findings?

Authors response: The term "number of aerenchyma" has been replaced by the term "amount of aerenchyma-like structures" that better reflect the anatomical trait quantified in this case. The *Lemnaceae* represent a unique family of plants, characterized by roots following a vestigial trajectory and by plants undergoing miniaturization. These processes result in the loss of some root functions and anatomical traits present in other plants, as well as the compression of anatomical structures, making it difficult to confidently assign identities to the anatomical traits quantified in this study. Accordingly, we have replaced the term aerenchyma with aerenchyma-like structure, as a second analysis revealed insufficient evidence to classify these air spaces as true aerenchyma, despite their morphological similarity. In the revised Supplementary Fig. S3 and Fig. 1c, we included analyses of all 21 root traits examined in this study, rather than only the amount of aerenchyma-like structures.

Reviewer #1 comment 94
94- Fig. 3a: what kind of statistical test was applied here and what is the overall result of it?

Authors response: We applied a post-hoc tests. In this analysis, we found significant differences in root and frond alpha diversity in a limited number of the plant species analysed (Extended Data Fig. 3a). For example, in the case of root alpha diversity, we observed significant differences between the group of *Pistia stratiotes* and *Lemna tenera* 9243 and the rest of plants analysed. In the fronds, only *Pistia stratiotes* and *Lemna yungensis* LY9208 showed significantly different alpha diversities (Extended Data Fig. 4a). This information is now included in the main manuscript.

Reviewer #1 comment 95
95- Fig. 3b: the aspect of mortality remains unexplained; what does it mean, how was it determined, and is it actually relevant here?

Authors response: In this figure, we aim to illustrate our hypothesis that microbial colonisation can influence root anatomy, with downstream consequences for plant survival in competitive environments. The panel shows a significant positive correlation between microbial alpha diversity and root anatomical complexity, suggesting a link between microbial colonisation and root anatomy. We coloured the data points (species) according to their mortality rates, as evaluated in Fig. 1e-g, to highlight the ecological relevance of this correlation. We believe that integrating these three concepts is essential for understanding the remainder of the manuscript.

Reviewer #1 comment 96
96- Fig. 3c: what is the compartment "inoculum"?

Authors response: Here, we refer to the initial inoculum used to inoculate the bacterial synthetic community into our samples. To avoid confusion, we have removed the inoculum from the compartment legend.

Reviewer #1 comment 97
97- Fig. 3 e-f: x-axis labeling should be "sequences", not "sequence"

Authors response: Corrected

Reviewer #1 comment 98
98- Fig. 4 b-d: There are several grammatical mistakes (singular/plural) in the figure legend text related to these plots.

Authors response: We thank the reviewer for this comment. These mistakes have been corrected.

Reviewer #1 comment 99
99- Fig. 4e: to keep it as intuitive as possible, I recommend adding the error bars to this figure, likewise as in Fig. 1d.

Authors response: The colour of the error bars has been enhanced to make them more visible and consistent in both figures.

Reviewer #1 comment 100
100- Fig. 4g: as far as I can see, PCoA2 was selected here for frond microbiome, whereas PCoA1 was selected for the root microbiome. Why not PCoA1 for frond microbiome, wouldn't that more likely result in significant mantel correlation?

Authors response: Following the reviewer's suggestion, we have prepared a new figure (Suppl Fig. S5g) using PCoA1 for the frond microbiome.

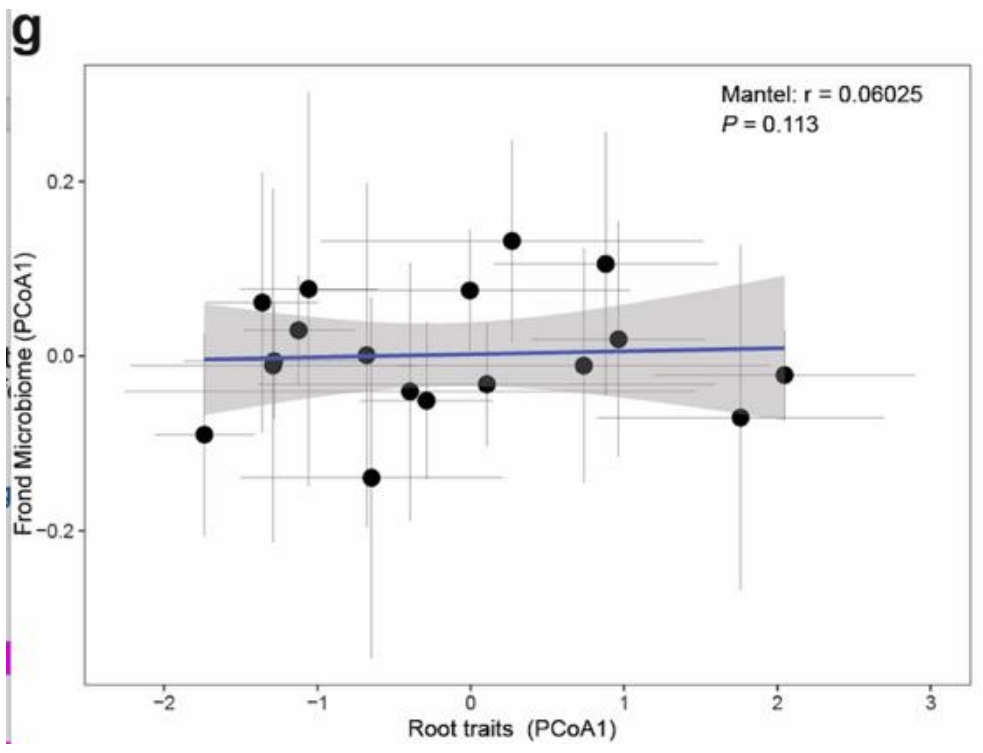

Reviewer #1 comment 101
101- Fig 5c: what type of correlation; also here, it is very difficult to differentiate significant from non-significant correlations

Authors response: We used a linear correlation. The colour of significant correlations has been changed to orange to make them easier to distinguish from non-significant ones.

Reviewer #1 comment 102
102- Fig. 6a: not readable at all; be more precise in wording it is not simply "16S rRNA", but 16S rRNA gene sequences; further, 16S rRNA should not be written in italic; what is the algorithm used for tree reconstruction here?

Authors response: Thank you for pointing this out. We have corrected the text in the figure legend by replacing "16S rRNA" with "16S rRNA gene sequences". In addition, the phylogenetic trees of the bacterial collection were constructed based on alignments of *16S rRNA* gene sequences (V1-V9 region) generated using the AlignSeqs function from the DECIPHER package (v2.30.0). These detailes are provided in the supplemental material, and the R code is available at https://github.com/JuanFrene/Duckweed

Reviewer #1 comment 103
103- Fig. 6b: this figure is not informative and should either be modified or removed

Authors response: This figure illustrates the extent to which our bacterial synthetic community represents, at the family level, the natural microbiota colonizing the different plant fractions in our natural water experiments. It is an important panel that demonstrates how representative our synthetic community is of the natural microbiota. Therefore, we prefer to retain it.

Reviewer #1 comment 104
104- Fig. 6c could be removed, the information given here appears to be redundant to Fig. 1a, in which the relevant taxa might eventually be marked; further, for the subsequent experiment (thermodynamics)

another selection of plant species was made, which is not specifically highlighted either, so why this selection here?

Authors response: Indeed, the information shown in panel 6c (new Supp Fig. 7b) is also presented in Figure 1a. However, we believe that this panel is important for readers to understand the specific selection of species used in these experiments; therefore, we prefer to retain it. Depending on the complexity and theoretical demands, we used different selection and number of species to appropriately address the biological questions. In all cases, we clearly explained both the number and the nature of the selected plants.

Reviewer #1 comment 105
105- Fig. 6d and e: how many replicates? Would it be possible to label the plant species with low, medium and high root complexity? Are the panels possibly sorted by complexity? That will help to quickly see possible changes in dependence on root complexity.

Authors response: The number of replicates has been added to the figure legends. We have also included a new legend for both figures with information on root complexity. The panels have been organized based on complexity. We hope that these changes have improved the interpretability of the figures.

Reviewer #1 comment 106
106- Fig. 6e: what is the unit of the rate?

Authors response: The figure represents the slope of the growth curves in each case. Because frond area (cm^2) was used as a proxy for plant growth and measured across multiple time points (days), the slope, representing the rate of change in plant growth, has units of cm^2/day. This information has been added to the figure legend.

Reviewer #1 comment 107
107- Fig. 6h: appears to present the same data as 6f; I don't see that both are needed. The authors never refer explicitly to only one of these.

Authors response: Although Figures 6f (new Supp Fig. 7f) and 6h (new Supp Fig. 7h) are based on the same dataset, they address different questions and are therefore analysed in distinct ways. In panel S7f, the data are compared to the full nutrient condition (½ SH) to assess the effect of nutrient concentration on root anatomical plasticity. This figure has been reorganized into two panels, each showing comparisons between the corresponding nutrient conditions (e.g., 1/100 SH vs. ½ SH and 1/1000 SH vs. ½ SH), separately for plants grown in sterile medium and for plants grown in the presence of the bacterial synthetic community.
In panel S7h, the same dataset is used to analyse the effect of the bacterial synthetic community on root plasticity. Here, within each nutrient condition (½ SH, 1/100 SH, and 1/1000 SH), samples inoculated with the synthetic community are compared against the no-bacteria control. Both figures are now cited independently in the main text.

Reviewer #1 comment 108
108- Fig. 8a: legend says "after microbial colonization", but I assume "with microbial colonization" would be more meaningful here? Do the figures show data of individual plants or replicates?

Authors response: Thank you for the comment. The figure legend has been updated and now reads "Thermogram showing the phase transition of root components from axenic plants (red) and from plants colonized by a synthetic bacterial community (blue) across different species of *Lemnaceae* with distinct levels of root complexity. Each value represents the result of analysing samples consisting of at least 10 roots. In most cases, microbial colonization altered the thermodynamic properties of the roots"

Reviewer #1 comment 109
109- Fig. 8f: "six species" is confusing, because data on 10 plant species are presented

Authors response: This typo has now been corrected.

Reviewer #1 comment 110

110- Fig. 8g: why was PCoA axis 2 chosen for the correlation analysis and not PCoA axis 1, which covers more variation? Besides, the reference to Fig. 8f appears incorrect, the PCoA plot is shown in Fig 8e.

Authors response: We thank the reviewer for the comment. Indeed, PCoA1 captures the largest proportion of variation in exudate profiles, which is likely driven by species-specific metabolic signatures, phylogenetic differences, and/or experimental variables such as batch effects, but is not related to root anatomical complexity (we did not detect a significant correlation between PCoA1 and the number of root cell layers). In contrast, PCoA2 explains a smaller proportion of the variability and represents a secondary but biologically more meaningful gradient in exudate composition, characterised by changes in specific metabolites associated with modulation of root cortical cell layer number. The significant correlation between the PCoA2 and the number of root cell layers might suggest that, across different plant species, a conserved change in the secretion of specific metabolites, rather than a general metabolic shift, is linked to root anatomical plasticity. Therefore, PCoA2 was selected because it captures variation with direct relevance to the biological question addressed in this study.

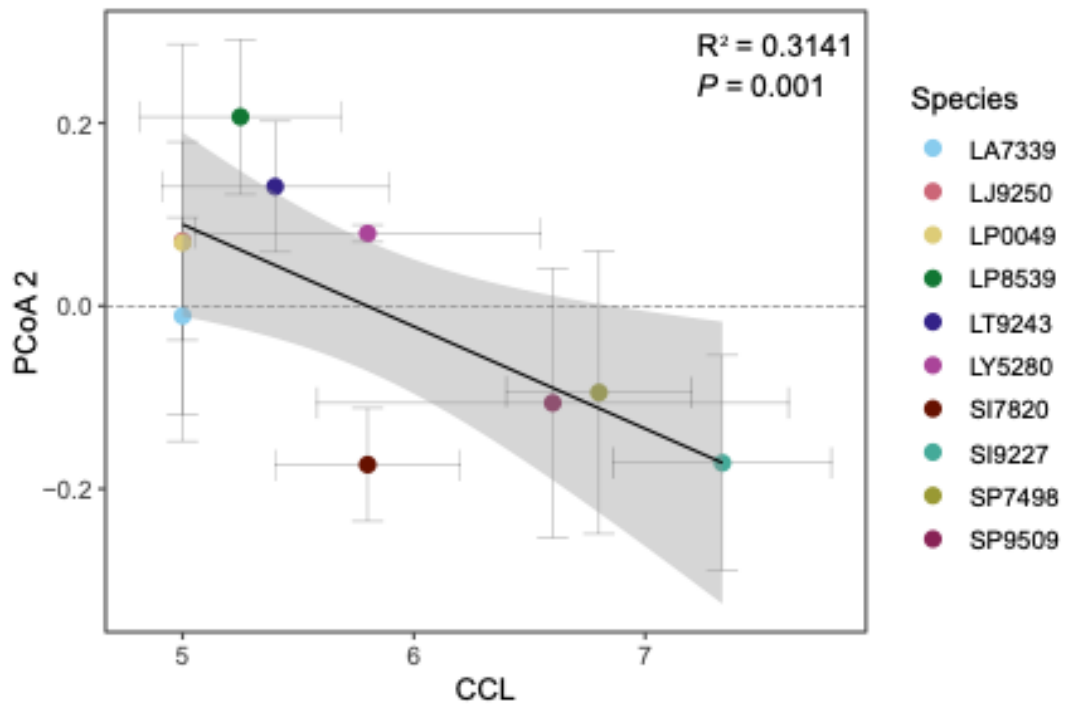

The reference to Fig. 8f (New 9f) has been corrected.

Reviewer #1 comment 111
111- Fig. 8i: it seems that the dots in the PCoA plot represent results from replicates, i.e. are mean values; explain this more clearly in the figure legend. Likewise, the statistical test applied here is not clear.

Authors response: Following the reviewer's comment, we have clarified this information in the legend of Supp Fig. S8i (now Supp Fig. S9i), which reads: "Principal coordinates analysis (PCoA) showing the projection of bacterial compositional profiles in synthetic roots of varying complexity (low, medium, and high), supplemented with Lemnaceae root exudates and inoculated with the bacterial synthetic community. Each point in the scatter plot represents the average distribution of values on both axes and is coloured according to the number of root cell layers (CCL) in the plant root from which the exudates were obtained. Error bars indicate standard deviations (SD). The bar on the right displays the percentage of variance explained by the experimental variables, as determined by Permutational Multivariate Analysis of Variance (PERMANOVA). Significant variables ($p < 0.05$) are highlighted in orange. R^2 values and their corresponding p-values from the PERMANOVA, shown within the panel, correspond to the CCL and plant exudate (PE) variables, respectively".

Reviewer #1 comment 112
112- Fig. 8k: I was already wondering when looking at Fig. 8h and 8i whether the CCL-numbers were specifically determined in this experiment for each plant sample used to collect root exudates or whether the CCL-number are taken from the first experiment, in which root traits were determined for all plant species; I initially assumed that the existing data were used, but what I find confusing here is the variation in CCL numbers for each of the individual symbols in the plot, with CCL numbers being not limited to integer numbers. This suggests that all replicates presented here were treated with root exudates from specific plant replicates, each consisting of multiple plants that were analysed individually regarding CCL to obtain mean values?

Authors response: We thank the reviewer for this comment. The CCL values used in Fig 8h and 8i (currently Fig S9h and S9i) were originally quantified in the first experiment. The reported CCL value

represents the average of the values determined from plant roots grown in sterile water and in natural water.

Reviewer #1 comment 113
113-Fig. S10: is a selection of metabolites shown here or all? Legend for fold-change does not display "-" correctly; dendrograms unclear.

Authors response: Supp Fig.S10 (now Supp Fig.S11) shows the results of our targeted metabolic analysis. Metabolites were identified by comparing experimental spectra with those of authentic reference standards analysed in-house using the same LC-MS/MS methods. Data were processed using a combination of custom Python code, manual curation, and a workflow implemented in MetAtlas (https://github.com/biorack/metatlas). Confidence scores ranging from 0 to 3 were assigned to each feature (defined by a unique mass-to-charge (m/z) ratio and retention time (RT)) based on established criteria from the Metabolomics Standards Initiative. A compound was considered positively identified when the detected *m/z* was within ≤ 5 ppm or 0.001 Da of the theoretical value and the RT was within ≤ 0.5 min of the corresponding standard. The highest confidence level (score of 3, exceeding Level 1) was assigned when the feature also exhibited matching fragmentation spectra to the standard. Identifications were invalidated when the acquired MS/MS spectra did not match those of the reference standard. A summary of compound identifications and confidence levels is provided in Table S4, along with mirror plots comparing experimental and standard MS/MS spectra (Supplementary Data 1).
The legend of the figures has been improved, and it reads: "
Extended Data Fig. 11. Root metabolic profiles change upon colonisation with the root microbiota. a. Heatmap showing the normalised (*z*-score) metabolite abundance detected using positive and **b.** negative ion modes in mass spectrometry. Metabolites were measured in the roots of two *Lemnaceae* species, *Lemmna aequinoctalis* 7339 (low anatomical root complexity) and *Spirodela polyrhiza* 9509 (high anatomical complexity). Plants were grown with the full bacterial synthetic community (Syncom), with three drop-out synthetic communities in which ASVs A289, A245, and P65 were individually removed, or in mono-association experiments with these ASVs (mA289, mA245, and mP65) compared to plants grown under axenic conditions (NB). Values are clustered according to metabolite abundance. The dendrogram represents hierarchical clustering of metabolite profiles, and the scale bar indicates the distance between identified clusters. Statistically significant metabolites relative to those in axenic plants are outlined in black ($q < 0.1$) (n = 4).

Reviewer #1 comment 114
114- Fig. S11a: this figure appears redundant to the one in Fig. 1a and without novel information. Though, following the description in the figure legend, I do not understand how the data about plants grown axenically or inoculated with Syncoms are implemented in this figure.

Authors response: Indeed, the information shown in panel S11a (Now Supp Fig. S12a) is already presented in Figure 1a. However, we believe this panel is important for readers to understand the selection of species used in these experiments and therefore prefer to retain it. In panels S11b and S11c (Now Suppl Fig. S12b and S12c), the heatmaps show the normalized abundance of total root metabolites in plants inoculated with the bacterial synthetic community compared to axenic plants (Suppl Fig. S12b). Supp Fig. S12c shows the normalized abundance of putative metabolites with a significantly different accumulation in plants inoculated with the bacterial synthetic community compared to axenic plants. This analysis serves as the first filter to identify putative metabolites involved in regulating the anatomical plasticity of roots in response to the microbiota. To clarify this, we have improved the figure legend.

Reviewer #1 comment 115
115- Fig. S11b-d: why are these heatmaps so blurry? Each metabolite should have a specific abundance in each of the plant species, color gradients between adjacent entries do not appear meaningful.

Authors response: We have submitted high-quality images to the journal. The heatmaps display the normalised abundance of putative metabolites present in the roots of nine Lemnaceae species inoculated with the full bacterial synthetic community, compared with plants grown under axenic conditions. Therefore, similarities in metabolite fold changes across different plant species are expected. We apologise to the reviewer, as we do not fully understand the reviewer's comment regarding adjacent entries.

Reviewer #1 comment 116
116- Fig. S11b and c: it is said in the legend that the data are organized according to the number of CCL, indicated by the boxes at the bottom of the plot, but these do not indicate a systematic sorting along a color gradient. Futher, I do not get what the difference between these two figures is and why both are needed.

Authors response: To avoid confusion, we have clarified this information in the figure legend. Now, we stated that plant root complexity, measured as the number of root cell layer, is shown in the bottom bar.
Figure S12b (previously Figure S11b) is a heatmap showing the normalized abundance of all putative metabolites identified in the roots of nine Lemnaceae species inoculated with the full bacterial synthetic community, compared with plants grown under axenic conditions. In Figure S12c (previously Figure S11c), the heatmap shows the normalized abundance of the 341 putative metabolites that accumulate significantly differently in plants inoculated with the bacterial synthetic community compared to axenic plants. Thus, panel S12b contains the information presented in S12c, however, we believe that panel S12c represents an important first filtering step, identifying the specific metabolites that are significantly affected in root by SynCom inoculation. This information is relevant for the interpretation of the data presented in the manuscript.

Reviewer #1 comment 117
117- Fig. S11d seems not to be relevant to support the statement in l. 351-353.

Authors response: We respectfully disagree with the reviewer's comment. While panel S12d (previous Fig. S11d) does not depict correlations, it illustrates how the concentrations of different metabolites vary across plant species with different root complexities. These data nevertheless suggest potential correlations between metabolite accumulation and root complexity, which are explicitly demonstrate in panel S12e (previous Fig. S12e). We therefore believe that the information provided in the two panels is complementary and have decided to retain both.

Reviewer #1 comment 118
118- Fig. S11e, legend: the reference to panel d does not appear meaningful. And what kind of correlation analysis was performed here?

Authors response: We removed references to panel d in the figure legend. Now it reads: "Linear regression analyses showing significant correlations between the abundances (log_2fold-change) of 15 putative metabolites identified in this study and the number of root cell layers (CCL) in the Lemnaceae species examined (indicated by colours). The proportion of variance in metabolite abundance explained by the number of root cell layers (R^2), along with the associated *p*-values, is shown in the panel". Linear regressions were used for this analysis.

Reviewer #1 comment 119
119- Fig S12a: figure legend below the six panels has text formatting problems; similarly, Fig. S12e has problems.

Authors response: This has been corrected.

Reviewer #1 comment 120
120- Fig S12d: I do not understand this plot, the color coding is problematic, there are different colors in the plot and in the legend and the syncom, represented by squares, is only depicted once despite the fact that it was applied with different concentrations of trimethyl-lysine. I cannot conclude anything here that supports the statements in the text.

Authors response: The legend for this panel has been revised. In panel S13c (previously Fig. S12d), the treatment legend now includes the following treatment: (SynCom + N6,N6,N6-Trimethyl-L-lysine, NB, and Syncom). Only the SynCom + N6,N6,N6-Trimethyl-L-lysine treatment is colour-coded. This is also clarified in the figure legend as follows: "Principal coordinates analysis (PCoA) showing the projection of root anatomical traits measured in *Spirodela polyrhiza* 9309 (high root anatomical complexity) grown with the full bacterial synthetic community, either alone (square) or supplemented

with different concentrations of N^6,N^6,N^6-Trimethyl-L-lysine (25, 50, 75 µM) (coloured circles). As a control, plants were grown under axenic conditions (NB) (triangle)".
The principal coordinate analysis shown in this panel is a projection of root anatomical traits quantified in *Spirodela polyrhiza* 9509, with an inherent complex root, exposed to increasing concentrations of N^6,N^6,N^6-Trimethyl-L-lysine in the presence of the microbiota under low nutrient conditions. At low concentrations of this compound, quantification of root anatomical features yielded results comparable to those observed in untreated plants colonised by the synthetic bacterial community (square and red circle clustering together) (Extended Data Fig. 13c-d). However, roots treated with higher doses of N^6,N^6,N^6-Trimethyl-L-lysine (50 and 75 µM) have anatomical features were in general similar to those of untreated plants grown under axenic conditions (green circles away from the black square (Extended Data Fig. 13c-d)). These conclusions were more evident when we correlated individual root anatomical features measured in plants treated with different concentrations of N^6,N^6,N^6-Trimethyl-L-lysine with those obtained from axenic roots or from roots inoculated with the bacterial synthetic community. We found that roots treated with a low concentration of N^6,N^6,N^6-Trimethyl-L-lysine (25 µM) exhibited the highest number of anatomical features that correlated with those observed in roots inoculated with the bacterial synthetic community treatment (Extended Data Fig. 13e). In contrast, higher doses of this compound (50 and 75 µM) compromised root plasticity in response to the microbiota, resulting in plants whose key anatomical traits showed reduced correlation with those of microbiota-inoculated roots and increased similarity to those measured in axenic plants (Extended Data Fig. 13e). We hope the reviewer can appreciate the importance of this figure to the paper's narrative.

Reviewer #1 comment 121
121- Fig S12e: I do not understand the control termed "NB". The legend says that fold-changes between plants with Syncoms vs. plant with Syncoms plus trimethyl-lysine are displayed. The Control is supposed to show plants without syncom, so how to calculate a fold-change here? Was a +/- trimethyl-lysine treatment applied and if so, which concentration?

Authors response: In the panel Fig. S13d (previously Fig. S12e), the NB control represents plants grown under axenic conditions (i.e., without bacteria and without N^6,N^6,N^6-Trimethyl-L-lysine treatments). This control captures the inherent root anatomical complexity of the plant used. As explained in the figure legend, the heatmap shows the fold change in root anatomical traits of *Spirodela polyrhiza* 9309 grown with the full bacterial synthetic community, either supplemented with different concentrations of N^6,N^6,N^6-Trimethyl-L-lysine (25, 50, 75 µM) or under axenic conditions (NB), relative to plants inoculated with the bacterial synthetic community alone.

Reviewer #1 comment 122
122- Fig. S13b and c: show again these diffusive color gradients that do not appear meaningful.

Authors response: We sincerely apologize to the reviewer; however, we are unable to observe the reported diffusive colour gradient in these panels. This may be due to a visualization issue related to the software used to open the figure. We suggest viewing the figure using Adobe Acrobat, where the figure is clearly visible.

Reviewer #1 comment 123
123- Fig. S13c: legend says that synthetic roots and conditions are the same as in panel c, this is panel c!

Authors response: Fixed

Reviewer #1 comment 124
124- Fig. S13d: only one of the six figures is required.

Authors response: Following the reviewer's comment, we removed the rest of the panels.

Reviewer #1 comment 125
125- Supplementary tables:
I recommend adding all smaller tables to the supplementary data file and keep only the large table as extra file, especially if each table has to be downloaded separately later.

Authors response: Following the reviewer's recommendation, we have added two small tables to the Supplementary Information document. Only the large tables are retained as Table S1 to Table S6.

Reviewer #1 comment 126
126- Table S2: I find it very useful to use R2A medium for aquatic samples, but it is unclear why there was methanol in that medium? That is not a usual component of R2A. Besides, correct the word "methanol". Check also mannitol. Lastly, I recommend harmonizing the writing of the chemicals.

Authors response: We agree with the reviewer that methanol is not a standard component of R2A medium. However, in this study, R2A was supplemented with methanol to favour the growth of facultative methylotrophic bacteria, which efficiently use methanol as a C source.
In addition, the terms methanol and mannitol have been corrected, and the writing of chemical compounds has been harmonised throughout table.

Reviewer #1 comment 127
127- Your table S4 is labelled as table S5 in the excel file.

Authors response: It is corrected now.

Reviewer #2 (Remarks to the Author):

Reviewer #2 comment 1
1- Review Frene et al The metabolic and anatomical complexity of root microhabitats modulate their interaction with the microbiota
Title stays very general but suggests a causal relationship
Abstract – only clear hard core message ".. specific metabolite N6,N6,N6-Trimethyl-L-Lysine has been identified as controlling "root anatomical plasticity"

Authors response: In addition to the specific metabolite, we also mentioned the anatomical complexity of root microhabitats as a driver of plant–microbiota interactions. These findings in the abstract are in synergy with the manuscript title.

Reviewer #2 comment 2
2- The Introduction is very short, stays very general, remains vague about the scale to be addressed and meanders between morphology and anatomy of roots and single cells. No actual state of the art is presented. No discussion of system complexity or reductionist approach is made. Very disappointing introduction. The introduction provides no information on the plant system to be investigated.

Authors response: We thank the reviewer for this comment. We have expanded the Introduction to include state-of-the-art information on how root anatomy influences microbiota recruitment and vice versa. In addition, we have added further details about the plant system investigated in this study. We believe that these additions have significantly strengthened the Introduction.

Reviewer #2 comment 3
3- Only from the results section one learns that the article is obviously about aquatic plants; i.e. plants naturally not growing in soil – this is a very poor management of readers expectations.
I continued reading until the middle of the results section without getting across any exciting novelty – there I stopped reading.

Authors responses: This manuscript represents, to our knowledge, the first systematic and comprehensive effort to link the anatomical and metabolic structure of root microhabitats with microbiota recruitment and establishment. We hope that the significance of this contribution is now clearer following the revision made to the manuscript.

Reviewer #2 comment 4
4- My conclusion:
May be the authors describe something exciting, but it is very disappointing that they failed to inform the reader, from the title, abstract and introduction, about the system they are dealing with – aquatic

plants. They completely ignore terrestrial systems, which is fine if they stated this, but they do not. I even think that they could learn a lot from reading the literature on terrestrial plants/ land plants. In my opinion, this is completely unsuitable for the journal.

Authors responses: Following the reviewer's recommendations, we have included sufficient information about the plant system in both the Abstract and the Introduction. We agree with the reviewer that reading entire manuscripts and the related literature is an important component of the learning process.

Reviewer #3 (Remarks to the Author):
Reviewer #3 comment 1
1- This manuscript explores the relationship between root anatomical complexity, metabolic profiles, and microbiota assembly in Lemnaceae. The combination of in vivo experimentation, synthetic root models, and metabolomic analysis is original and yields a convincing framework linking structural and metabolic root traits to microbial colonisation. The identification of N6,N6,N6-trimethyl-L-lysine as a regulatory metabolite represents a significant and novel finding.

Nevertheless, several areas require clarification or improvement before publication.

Authors response: We thank the reviewer for taking the time to read and comment on our manuscript, as well as for their positive opinion about our work.

Reviewer #3 comment 2
2. Mechanistic Clarity. The role of N6,N6,N6-trimethyl-L-lysine in controlling microbiota-driven root plasticity should be elaborated. Does this compound act directly on microbial physiology, on plant signalling, or both?

Authors response: Following the reviewer's comment, we have clarified our hypothesis regarding the mechanism of action of this compound in intact roots. Although we have demonstrated the role of N^6,N^6,N^6-trimethyl-L-lysine as a driver of root plasticity in response to the microbiota, we had not described its mechanism of action in intact root systems. Based on previous publications (Tsai et al, 2025, Science 390, eadu4235 (2025). DOI: 10.1126/science.adu4235) and our own data, we propose that the differential local accumulation of N^6,N^6,N^6-trimethyl-L-lysine across root microhabitats influences the spatial distribution of microbiota members. These studies showed that differential glutamine leakage across the root cylinder controls microbial spatial organisation, as glutamine can serve as a carbon source for specific microbes. A similar mechanism might operate in our system, whereby the heterogenous accumulation of N^6,N^6,N^6-trimethyl-L-lysine across anatomically complex root microhabitats modulates microbial growth by serving as a carbon source, consistent with our in vitro microbial growth analyses. This, in turn, might alter microbial activity across root tissues and ultimately lead to changes in root plasticity. This information has now been incorporated into the main manuscript.

Reviewer #3 comment 3
3-Include discussion of possible biochemical or redox mechanisms and whether this metabolite could influence methylation or stress-response pathways.

Authors response: Following the reviewer recommendation, we expand our conclusion section to discuss possible functional mechanisms for N^6,N^6,N^6-Trimethyl-L-lysine. This information included its role as a carnitine precursor, a molecule that plays a central role in plant tolerance to stress. For example, carnitine functions as antioxidant, directly protecting plant cells from the damaging effect of reactive oxygen species generated in response to various abiotic stresses. Furthermore, carnitine acts as an effective osmoprotectant, helping plants maintain cellular turgor under osmotic stress conditions. Exogenous application of carnitine has been shown to promote plant growth and stimulate mitosis under salt stress. We also discussed the indirect effect that the synthesis of N^6,N^6,N^6-Trimethyl-L-lysine can have on the cell's methylation metabolism. By affecting methyl group availability, changes in the level of N^6,N^6,N^6-Trimethyl-L-lysine could indirectly impact DNA and histone methylation, potentially altering the expression of genes related to development and stress responses. It is also known that active DNA demethylation regulates myo-inositol secretion by roots, with consequences for beneficial interactions between the plant and a member of the root microbiota. Similarly, dysfunction of the histone

demethylase IBM1 alters root microbiota composition in *Arabidopsis thaliana*. We added information about the fact that we cannot exclude the possibility that local changes in N^6,N^6,N^6-Trimethyl-L-lysine levels, resulting from its interaction with the microbiota across different Lemnaceae species, may affect the pool of available carnitine in the plant. Nor can we exclude that indirect effect of changes in N^6,N^6,N^6-Trimethyl-L-lysine concentration could impact epigenetic mechanisms controlling plant-microbiota interactions. Both scenarios might have consequences for plant abiotic stress tolerance and root plasticity.
We also, discussed the fact that our results are consistent with a recent publication demonstrating that the differential local accumulation of glutamine along the root cylinder influences the spatial distribution of microbiota members capable of using it as a carbon source. It is therefore plausible that the local accumulation of N^6,N^6,N^6-Trimethyl-L-lysine across the anatomically distinct root microhabitats might similarly influence the spatial distribution of beneficial microbiota members by serving as carbon source.

We believe that these changes have improved the quality of our manuscript, and we thank the reviewer for their valuable feedback

Reviewer #3 comment 4
4-L258-260: explain/elaborate this concept.
Authors response: Thank you for the suggestion. We have rephrased the text to clarify this concept. It now reads:
"Overall, these results suggest a feedback loop between root microhabitat traits, both anatomical and metabolic, and the composition of the microbiota they host. Specifically, the anatomy and the metabolic composition of different root microhabitat might influence microbial establishment, and, in turn, microbes might alter the anatomical and metabolic properties of the root".

Reviewer #3 comment 5
5-Root Complexity vs. Plasticity. Distinguish clearly between inherent anatomical complexity and microbiota-induced anatomical plasticity. The manuscript occasionally conflates these concepts, which could confuse readers regarding causality.

Authors response: We thank the reviewer for this comment. We have now clearly distinguished between inherent anatomical complexity and microbiota-induced anatomical plasticity throughout the manuscript.

Reviewer #3 comment 6
6- Reproducibility and Methodological Transparency. Provide detailed descriptions of synthetic-root material composition and preparation.

Authors response: We have included a detailed protocol for the synthetic root preparation in the Materials and Methods section (please see Material and Methods *5.7. Preparation of synthetic roots with root exudates).*

Reviewer #3 comment 7
7-Include LC–MS/MS parameters used for metabolite identification and confirm annotation quality.

Authors response: Following the reviewer's recommendation, we have expanded the Materials and Methods section to include details on metabolite identification. Please see Materials and methods section: *3.2. Exudates preparation for metabolic analyses; 3.3. LC-MS Analysis of the root exudates; 4.4. Metabolic analysis of Lemnaceae roots; 5.2. Samples extraction for metabolic analyses; 5.3. LC-MS Analysis of the plant root metabolic profile.*
We have also prepared two new tables describing the parameters used and the levels of confidence for metabolite identification and annotation (Table S4 and Table S5). In addition, we have included a new supplementary dataset containing all positive- and negative- ion mirror spectra from the LC-MS metabolomic analysis (Supplementary Dataset 1).

Reviewer #3 comment 8
8-State the number of biological replicates and normalisation (e.g., 16S data).

Authors response: The number of biological replicates has been added to the figure legends, and the normalization method used is now described in the Materials and Methods section.

Reviewer #3 comment 9
9- Data Presentation and Structure. The abstract and results are dense; reduce descriptive language and emphasise key findings.

Authors response: Following the reviewer's recommendation, we have significantly revised the abstract and results to reduce content density while emphasizing the main findings.

Reviewer #3 comment 10
10- Improve figure captions: define abbreviations (e.g., CCL), consolidate statistical details, and ensure clarity of legends.

Authors response: We thank the reviewer for this recommendation. We have significantly revised all figure legends, defined all abbreviations, and clearly described the statistical analyses used. These changes have substantially improved the clarity of the manuscript.

Reviewer #3 comment 11
11- Consider adding a schematic summarising the anatomical–metabolic–microbiota feedback model to aid reader understanding.

Authors response: We have added a model to facilitate reader understanding of the manuscript. This is presented as the new Fig. 4g.

Reviewer #3 comment 12
12-Minor Comments. Ensure consistent taxonomic and metabolite nomenclature throughout.

Authors response: We have harmonised taxonomic and metabolite nomenclature throughout the manuscript

Reviewer #3 comment 13
13- Revise the conclusion to focus on implications for microbiome optimisation and crop improvement.

Authors response: Following the reviewer's comments, we have added a paragraph to the conclusion section discussing how our new findings can be integrated with synthetic biology approaches to promote the development of novel microbiota-based strategies that enhance plant stress tolerance in agricultural settings.
"Although a deeper understanding of the genetic components underlying the mechanism described here is required before its application, we envision that this knowledge could contribute to optimising root microbiota function to enhance root plasticity and plant stress tolerance. Plants can be genetically engineered to optimise the production of modulatory metabolites that positively influence the recruitment of beneficial microbiota[45,46]. Alternatively, using synthetic biology strategies, we can precisely control the exudation of key metabolites that microbes use as carbon sources, thereby affecting microbial recruitment and localization[47,48,49]. These strategies could be used to effectively regulate the concentration of N^6,N^6,N^6-Trimethyl-L-lysine in roots according to their inherent anatomical complexity, maximising beneficial microbe-driven root plasticity. Beyond optimising modulatory metabolite concentrations, synthetic biology approaches hold potential to specifically enhance aspects of root anatomy without negatively impacting root function, including its interactions with the microbiota[50,51]. Combined with the direct application of N^6,N^6,N^6-Trimethyl-L-lysine, these strategies could modulate root interactions with beneficial microbes. Together, these advances, along with our growing understanding, should stimulate the development of new microbiota-based strategies with a positive impact on agricultural practices".

Reviewer #4 (Remarks to the Author):

Reviewer #4 comment 1
1- Authors in this manuscript entitled "The metabolic and anatomical complexity of root microhabitats modulate their interaction with the microbiota" have gathered a large and thorough amount of experiments and data to study the interaction and correlation between root anatomy and its plasticity

with the microbiota composition/recruitment and nutrient availability in duckweed. The experiments include using synthetic root models and synthetic microbiota with 16S rRNA sequencing and metabolomics approaches to study different variables independently.

Authors response: We thank the reviewer for taking the time to read and comment on our manuscript.

Reviewer #4 comment 2

2- My comments are largely on the metabolomics results and their significance. Unfortunately, the methodology included in the Extended Material is very scarce and does not allow me to evaluate correctly the results from the metabolomics approaches. The metabolomics processed data is also not included as supplemental material. I would also strongly recommend submitting the raw metabolomics data to a data repository such as MetaWorkbench.

Authors response: We thank the reviewer for this suggestion. We have submitted all metabolomic data to MetaWorkbench. It is available now under the Data Track ID 7037. In addition, all raw data used to generate the figures in this work are available at: https://github.com/JuanFrene/Duckweed.

Reviewer #4 comment 3

3- Major comments. Section 3.2/3.3 in the Extended Material; for the analysis of the root exudate using LC-MS with Zic-pHILIC column on QE-plus, there is no details on how the data was further processed: normalization, QC correction, blk subtraction, comparison; and statistically analyzed??

Author response: We thank the reviewer for this comment. For all untargeted analyses, pooled QC samples were used to evaluate instrument suitability and were subsequently included in the Compound Discoverer Workflow for normalisation (only peaks present in more than half of the QC were retained). In addition, the blank subtraction parameters have now been added to the supplementary materials.

For example: “Raw files were processed using Compound Discoverer v3.3 (Thermo Fisher Scientific, Bremen, Germany) for data pre-processing and molecular formula determination. Retention time alignment was performed using pooled quality control (QC) samples. Extraction blanks were used to identify and mark background features. Peak detection was performed using a minimum peak intensity threshold of 1×10^4, and features were retained only if detected in at least 2 files per class. A peak rating threshold of 2 was applied. Only features present in at least 50 % of the QC samples were included in the final dataset.

Background features were flagged using the Mark Background node based on sample-to-blank intensity ratios and excluded from downstream analysis. Missing values were addressed using the Fill Gaps algorithm with a mass tolerance of 5 ppm and a signal-to-noise (S/N) threshold of 1.5. Molecular formula prediction was performed with a mass tolerance of 5 ppm and a minimum isotopic pattern match of 30%”.

For the statistical analysis, we performed a multivariate analysis based on Principal Coordinates Analysis (PCoA) using all detected exudates (n = 1024), shown in Fig. S9e of the supplemental material. In addition, we generated a heatmap with the 250 most abundant metabolites, selected based on their average abundance across all samples. Details of these analyses are provided in the Materials and Methods.

Reviewer #4 comment 4

4- What data was used to create Extended Figure 8f (terrible resolution of the figure) and 8g. How many features are detected? How were they evaluated? How were the most abundant selected? Authors must add a Supplemental Table with this information.

Author response: We thank the reviewer for the questions. For Fig. S8f and S8g, we used results from exudate analyses of 10 Lemnaceae species. These exudate analyses were based on untargeted metabolomic analyses. In total, 1097 putative metabolites were detected, and 629 were identified with a sufficient level of confidence to assign a putative name. We added two tables: Table S3, containing metabolite abundances, and Table S4, containing metabolite identities and features detected in this experiment. All data can be found at https://github.com/JuanFrene/Duckweed.

To evaluate Lemnaceae exudate profiles, we performance a PCoA using all 1097 putative metabolites detected (Fig S9e). In addition, we generated a heatmap of the 100 most abundant metabolites (Fig S9f). Finally, we performed a linear correlation between the PCoA2 axis from Fig S9e and the root cortical cell layer number of each Lemnaceae species used in this experiment (Fig S9g).

To select the 100 metabolites used in the heatmap, we averaged across all sample replicates and selected the 100 highest-abundance features overall (Fig S9f).

Reviewer #4 comment 5
5- Section 4.4/4.5 in the Extended Material; for the analysis of the Lemnaceae roots after dropouts of individual ASVs using LC-MS with Poroshell 120 HILIC-Z column on QE, again there are almost no details on how the data was processed.

Author response: We thank the reviewer for this comment. Following the reviewer's recommendation, we have significantly expanded the methods related to the root metabolic analyses. We included detailed information on how the metabolites were extracted, how the samples were processed, and how the LC-MS/MS analysis was performed. We also added information on how the data were processed (see Materials and Methods *4.4. Metabolic analysis of Lemnaceae roots)*
.

Reviewer #4 comment 6
There is mention of MetAtlas. What is this? Are Authors referring to Metabolic Atlas. This is not a tool to analyze mass spec data.

Author response: We thank the reviewer for pointing this out. MetAtlas is our targeted metabolite annotation pipeline and not Metabolic Atlas. To avoid future confusion about the terminology used, we have clarified that, for the targeted analysis, metabolites were identified by comparing experimental spectra to authentic reference standards run in-house using the same LC-MS/MS methods. The data were processed using a combination of custom Python code (Yao et al., 2015), manual curation, and a pipeline workflow available through MetAtlas (https://github.com/biorack/metatlas). MetAtlas is not referring to Metabolic Atlas; in our case, it is a custom pipeline for the identification of metabolites.

Reviewer #4 comment 7
Also, the method seems to suggest that the annotation of the peaks was done using an in-house library of authentic reference standards (by the way how many compounds in the library).

Author response: Yes, that's correct—this pipeline is MetAtlas and is our tool for manually curating the experimental data vs. our analysis of authentic standards using the same methods and instrumentation. Our library has 5000 compounds.

Reviewer #4 comment 8
However, there are clearly mis annotations based on the Extended Fig 10a-b which shows heatmaps from the metabolite identified in pos and neg ion modes. These are meant to be complementary scans to allow the detection of greater number of compounds. However, looking at the heatmaps, there is one compound, pantothenic acid, identified by both showing contradictory changes in the same conditions (dA245 in SP9309). This seems to suggest that either the peak annotated in the pos or in the neg is wrongly annotated. Authors again need to provide the data in a supplemental table with the criteria for confidence levels of annotation.

Author response: We thank the reviewer for this comment. To address the apparent contradiction in the data shown in these figures, we have re-analysed the raw data used to generate them. In the specific case of pantothenic acid (and other compounds identified in both POS and NEG modes), we found that the identification confidence is high in both polarities. The mirror plots for pantothenic acid in POS and NEG modes (see below), as well as the ppm and retention-time values, support this observation. Furthermore, peak-height boxplots show that the intensity/abundance trends are similar between POS and NEG modes (see figures below). The same pattern was observed for a couple of other compounds we double-checked (e.g., guanine).

To resolve the apparent contradiction observed in the heatmaps, we re-analysed the data using raw values instead of the normalised ones. This change in the analysis was sufficient to eliminate the inconsistency noted by the reviewer. These revised figures have now been added as new panels in Extended Data Fig. 11a–b.

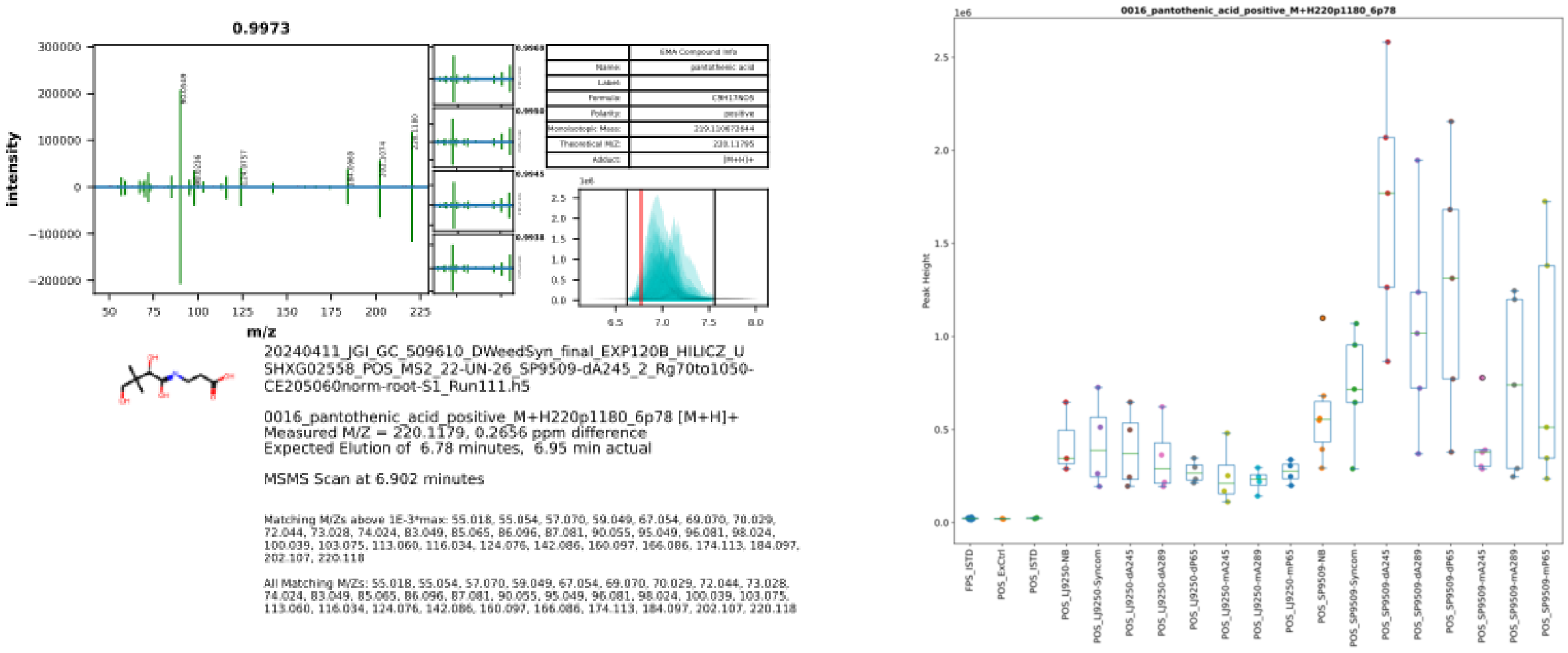

Mirror plots and peak-height boxplots for pantothenic acid in POS mode

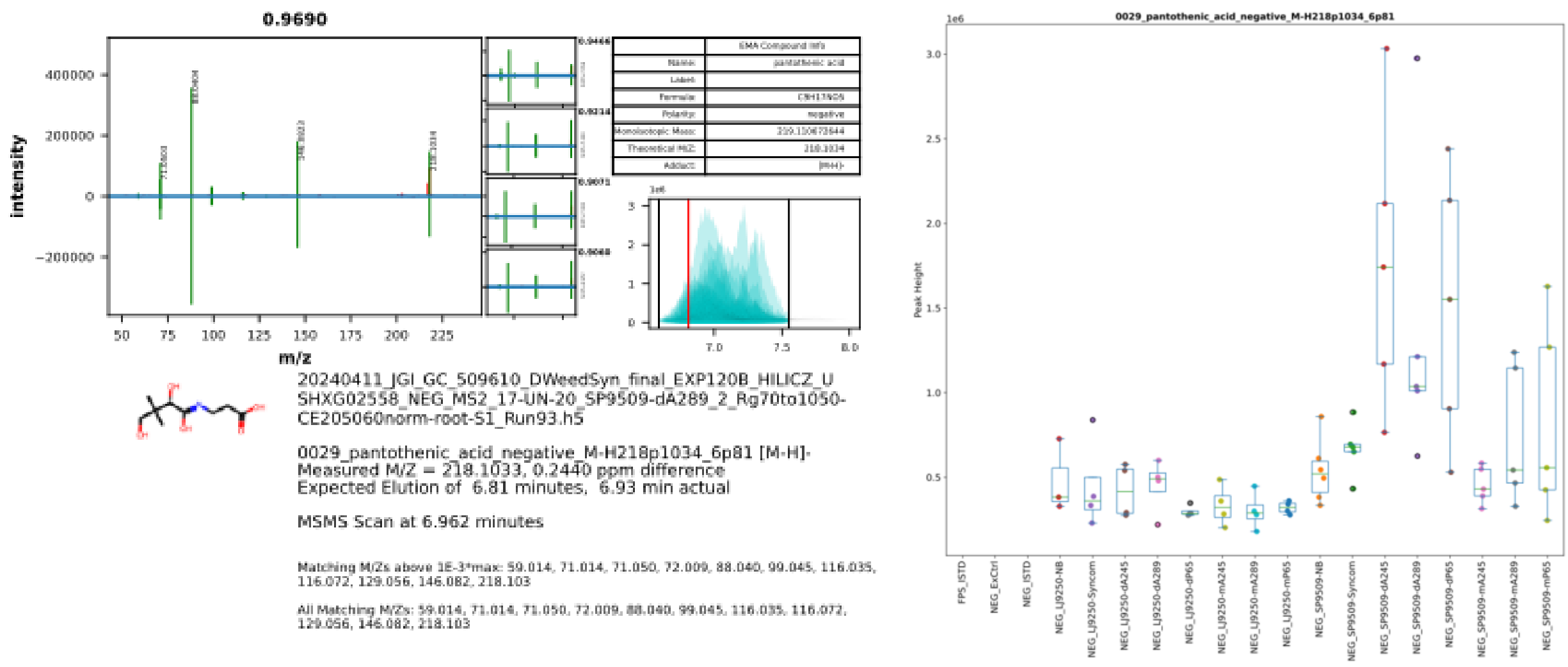

Mirror plots and peak-height boxplots for pantothenic acid in NEG mode

Reviewer #4 comment 9

6-Section 5.2/5.3; for the analysis of the Lemnaceae roots with different CCL using LC-MS with Zic-pHILIC column on QE-plus, again no details on how the data was further processed and annotated. Authors must add the data in a supplemental table and indicate level of confidence for annotations.

The few selected compounds annotated were tested for their effect on root plasticity before their annotation was confirmed. In this case, the samples had to be rerun on a Fusion Lumos, as described in Section 5.6, alongside the standard for trimethyl Lysine to confirm the peak was correctly annotated. What about the other compounds selected? Were they also run and confirmed to significantly show the data in Fig 4a, since the authors purchased all the standards?

Author response: Following the reviewer's recommendation, we have expanded the Materials and Methods sections 5.3 and 5.5 to include additional details on data processing and annotation. We have also prepared two new tables describing the parameters used and the levels of confidence for metabolite identification and annotation (Table S4 and Table S5). In addition, we have included a new supplementary dataset containing all positive- and negative- ion mirror spectra from the LC-MS metabolomic analysis (Supplementary Dataset 1).

We have now applied a stricter selection criterion for metabolite validation, including only metabolites whose identities were assigned with confidence level ID2 (Table S5). This reduced the candidates to two metabolites, L-citrulline and N^6,N^6,N^6-Trimethyl-L-lysine. After assessing the effects of these compounds on plants, only N^6,N^6,N^6-Trimethyl-L-lysine showed a significant effect on relevant plant phenotypes, and its identity was subsequently confirmed with confidence level ID1using a standard. All of this information has been incorporated into the new Fig. 4 and Extended Data Fig. 13.

Reviewer #4 comment 10
7- In addition, how did the authors select the concentration for the dose effect of the trimethylLysine? Did authors measure the concentration of the compound in the root exudate or what could be found in natural water?

The concentration used for exogenous supplementation of N6,N6,N6-Trimethyl-L-lysine were selected based on estimates of endogenous levels of this compound in Lemnaceae roots, as well as on established hormone-treatment protocols commonly used in plant physiology studies. In our system, the endogenous concentration of N6,N6,N6-Trimethyl-L-lysine in roots was roughly estimated at approximately 0.06 µM, based on the concentration of the analytical standard used. However, exogenous supplementation of N6,N6,N6-Trimethyl-L-lysine was performed at higher concentrations (25, 50, and 75 µM) to account for limited uptake efficiency, spatially distribution of the compound within the root, and metabolic turnover. This concentration range is consistent with commonly used hormone treatments known to induce anatomical and physiological effects in plants that we used as reference. For example, endogenous auxin (IAA) levels in roots typically range from ~10–100 nM, whereas exogenous auxin is routinely applied at 0.1–10 µM; endogenous abscisic acid (ABA) levels are generally ~20–200 nM, while exogenous ABA treatments commonly range from 5–50 µM. Similarly, cytokinins and gibberellins occur endogenously at low nanomolar levels but are often supplied exogenously at micromolar concentrations (0.1–10 µM). Accordingly, the exogenous-to-endogenous ratios used here (approximately 400–1,250×) fall well within accepted experimental ranges for hormonal treatments affecting plant physiology. The use of multiple concentrations further enabled assessment of dose-dependent effects.
The actual concentration of the N6,N6,N6-Trimethyl-L-lysine in root exudate was not measured in this study; however, this compound has been reported as a component of the exudate core metabolome of *Arabidopsis thaliana*, *Brachypodium distachyon*, and *Medicago truncatula* (McLaughlin, S. *et al*. The core metabolome and root exudation dynamics of three phylogenetically distinct plant species. *Nat Commun* **14**, 1649 (2023)).

Reviewer #4 comment 11

8- Minor comments. There is a lot of redundancy in the methodology. The method section could be shortened if the authors would refer to sections already described once. This document just looks like a copy and paste from a PhD dissertation. As an obvious example: Sections 3.2/3.3 and 5.2/5.3 are exactly the same.

Author response: Following the reviewer's comments, the Materials and Methods section has been significantly streamlined to avoid unnecessary duplications.

Reviewer #4 comment 12
9- Figure 3 is of poor resolution.

Author response: We have increased the resolution of figure 3.

Reviewer #4 comment 13
10- Extended Section 5.3 – change root exudate to root.

Author response: This typo has been corrected.

Reviewer #4 comment 14
11- Line 349. "This analysis identified 341 metabolites". This was confusing to me. It suggested that 341 metabolites were annotated but this is for the correlation of CCL and their abundance profile. Please rephrase it.

Author response: We thank the reviewer for this comment. In response, we have revised the text to better reflect our analysis. The updated text now reads: “To identify metabolites that modulate microbiota-driven root anatomical plasticity, we first selected those putative metabolites that differed significantly between axenic plants and plants inoculated with the bacterial synthetic community (Extended Data Fig. 12b-c), identifying 341 putative metabolites (Extended Data Fig.12c)”.

Reviewer #4 comment 15
12- Extended Fig 11. The heatmap in panel d) shows features # that do not match with the ones in panel e), which are supposedly the same 15 selected metabolites?

Author response: We thank the reviewer for pointing this out. In the revised version of the manuscript, we have corrected these mismatches.

Answer to Reviewers

Reviewer #1:

1- The authors addressed most of my previous comments convincingly. At this stage, I first read the revised manuscript and evaluated the responses to my comments afterwards. Accordingly, you will find a list of new issues first, followed by some comments from last time that require further clarification.

Authors response: We would like to thank the reviewer again for their insightful comments and suggestions, which have helped improve our manuscript. In response, we have revised the relevant sections of the manuscript to address all the new reviewers' concerns and to further improve the points raised previously.

2- 132-133: statement unclear

Authors response: The statement has been reworded for clarity. It now reads: “Significant differences in root and frond alpha diversity were detected in only a small number of the plant species analysed”.

3- 148: bacterial, not microbiota; here and elsewhere: don’t use the terms microbiota or microbial when referring to your own data, which are limited to the bacterial community.

Authors response: We thank the reviewer for this comment. The term has been corrected throughout the manuscript.

4- 178: sterile water means tap water, distilled water or sterilized natural water? This could become clearer here.

Authors response: It is clarified now: “We then evaluated plant growth kinetics, using total frond area as a proxy for plant growth, in natural water and in sterilised natural water”.

5- 198: better: “microbiota-induced complex root anatomy”

Authors response: We thank the reviewer for the suggestion. The text has been revised accordingly and now reads: “These results suggest that microbiota-induced complex root anatomy could enhance plant growth, improving plant survival under these challenging growth conditions”.

6- 264-266: sentence unclear

Authors response: We have revised the sentence to improve clarity. Now it reads: “The presence of these mechanisms in species with inherently complex root anatomy, for example a higher number of root cell layers, could increase plant survival under nutrient-limiting conditions”.

7- 301: specify type of change (increase with increasing complexity).

Authors response: Following the reviewer recommendation, we have modified the text and now reads: “All these results indicate that the microbiota increases anatomical complexity in roots that are inherently more complex and/or alter the metabolic properties of root microhabitats”.

8- 332: microhabitats.

Authors response: Corrected

9- 542: microbial, not microbiota.

Authors response: Corrected

10- 602: plants.

Authors response: Corrected

11- 669: reported p-value for post-hoc tests correct?

Authors response: Corrected

12- 671: delete “total”

Authors response: Deleted

13- 677: it is stated that a minimum of six samples was available, but the plot (2e) allows to see max. 5 per complexity level, do some replicates have identical communities (especially with decreasing complexity) or is there perhaps still a problem with this plot? Please check.

Authors response: Although the initial number of samples in this experiment was six, technical issues found during the sequencing and sequence analysis meant that some samples did not pass quality control. As a result, the total number of samples analysed was lower than originally planned. We have corrected this in the figure legends, which now state that the minimum number of samples used was three.

14- 682: if the author list changes, the author contributions should also be updated; further, not all authors of this manuscript appear in the contribution list, which raises questions.

Authors response: We thank the reviewer for pointing out this important issue. We inadvertently omitted the contributions of our collaborators from the DOE Joint Genome Institute in the author contribution list, who performed part of the metabolic analyses presented in the manuscript. Likewise, we have removed one author from the author list who was mistakenly included as a part of the team performing the metabolic analyses at the University of Nottingham. We have also added a new author whose intellectual contribution was significant in the preparation of the current version of the manuscript. All these oversights have now been corrected.

15- 848: axis labelling of Fig 4a says PCA, not PCoA as stated in fig. legend; is this indeed a principal coordinate analysis or a principle component analysis? Further, and in contrast to Fig 4a, the y-axis in Fig 4b is labelled as PCoA, though it was taken from Fig. 4a; what is correct?

Authors response: Thank you for pointing out this error. The Figure 4a is indeed a principal component analysis (PCA). Principal component 1 (PC1) derived from this analysis was used to assess the correlation between metabolite composition and the number of root cell layer in both sterile samples and samples inoculated with the bacterial synthetic community. Therefore, the y-axis in Fig. 4b has been labelled accordingly. This mistake has been now corrected.

16- 850 and 851: should probably be “Samples” instead of “Plant species”, as individual dots represent samples, not plant species.

Authors response: We thank the reviewer for the suggestion. The sentence has been corrected and reads now: “Samples are coloured according to the number of root cell layers (CCLs) present in the plant species from which they were collected”.

17- 851-852: I don’t see any error bars in figure 4a; however, error bars are shown in Fig 4b, which remain unexplained; also, add number or replicates.

Authors response: Figure 4a shows all samples used in the analysis; because of the large number of data point, error bars were omitted for clarity. In Figure 4b, we present the sample means with their corresponding error bars. In both cases, six replicates per plant species were used. This information has now been included in the figure legend.

18- 865: p-value cut-off for post-hoc tests?

Authors response: The cut-off for post-hoc tests was $p < 0.05$. This information was added to the Figure 4c legend.

19- 881-883: statement unclear; according to this description, there would be three treatments (I) exudates of S. polyrhiza, (II) exudates of L. japonica and (III) Trimethyl-lysine, but this is not the case, according to the figure, it is a 2-factorial design; clarify the description in this sentence.

Authors response: Our experimental design included two control conditions: synthetic roots supplemented with root exudates from two *Lemnaceae* species (*Spirodela polyrhiza* 9509 and *Lemna japonica* 9250) and inoculated with the bacterial synthetic community, and the experiment conditions consisting of the same setup but additionally treated with 50 µM N^6,N^6,N^6-Trimethyl-L-lysine. To clarify the interpretation of the figure, we have improved both the labels within the panel and the explanatory text in the figure legend.

20- 884: I assume it is “roots” here, if n=6

Authors response: Yes, it is “roots”.

21- 887: delete “of”

Authors response: The sentence has been corrected.

22- The supplementary material still includes a very comprehensive description of materials and methods. Redundancies have to some extent been eliminated. However, the description of synthetic roots should be described earlier, in the context of the first experiments using these, not at the end.

Authors response: Following the reviewer’s recommendation, we have moved the method describing the preparation of “synthetic roots” to the material and methods section 2.5, placing it before the description of the methods involving the use of “synthetic roots”.

23- Suppl. l. 1297: what type of correlation coefficient?

Authors response: The analysis used a Pearson correlation. This information has been added to the new Extended Data Fig. 11c.

24- Suppl. l. 1340: “A minimum of five roots was analysed for each condition (n=4).” This statement appears contradictory.

Authors response: We thank the reviewer for pointing this out. The inconsistency has now been resolved. The minimum number of replicates used was four.

25- Suppl. l. 1494: delete “absolute”; this remains a relative measure, as it refers to a specific microscopic area, where cells were counted on an artificial root surface; the differences in root complexity may contribute to an underestimation of counts, especially in the most complex roots, as I assume you cannot quantify cells deeper within the structure; absolute estimates from such images appear thus unlikely. (please correct this wording systematically in the manuscript).

Authors response: The term absolute has been removed from the manuscript.

26- Suppl. fig. 11: some fields in the heatmap appear to be completely black; what does that mean?

Authors response: The black squares in the heatmaps represent samples in which the analysed metabolite was not detected. This information has been added to the legend of the new Extended Data Fig. 21.

27- Suppl. l. 1705: I don’t see in the figure that plant species are organized according to root complexity; the information about complexity is given by a color coding bar, but there is no obvious sorting according to complexity in fig 12c.

Authors response: We have prepared new versions of the heatmaps shown in Extended Data Figure 12 (now Extended Data Figure 22), displaying the data ordered by root complexity. This ordering has been maintained across panel b, c, and d.

28- Likewise in l. 1714 and fig. 12d. Such sorting would be helpful here.

Authors response: Following the reviewer's suggestion, we have sorted the plant species according to their root complexity (see new Extended Data Figure 22d).

29- The formatting of the last two supplementary tables could be improved, not all text is visible and in part not well readable.

Authors response: We have increased the font and cell sizes of both tables to improve visibility and readability.

Related to former reviewer comments:
30- Reviewer #1 comment 9 9- l. 87: I do not see this in Fig. S1c, this type of presentation does not allow to see this easily. Remove reference to Fig. S1c.
The problem remains despite the modified figure. It would require a sorting of plant species according to root number (complexity) to clearly see the described differences. The hierarchical clustering, used at present to sort species/treatments, does not provide this information.

Authors response: We redesigned Fig. S1c (now Extended Data Fig. 2a, 2b) to better visualize differences in root anatomy between plants grown in sterile versus natural water. The revised figure contains two separate panels: one for plants grown under sterile conditions (Extended Data Fig. 2a) and one for plants grown in natural water (Extended Data Fig. 2b). In both panels, species are ordered by inherent root complexity. The updated figure shows that the presence of microbiota in the water induces clear changes in the measured root traits compared with those in sterile conditions. These differences are now more evident; for example, the number of clusters of anatomical features changes from three main clusters in sterile water to two in natural water.

31- Reviewer #1 comment 12 12- l. 95-96: I found in the supplementary methods the information that the weakest sample was represented by 500 reads. This is not much, does it represent one of the outliers in Fig. S3a? Usually, samples are rarefied and samples with insufficient coverage are removed to have robust comparisons of alpha diversity. How many reads did the other samples have and can you do a robust comparison here without rarefying the data; i.e. do the obtained read numbers cover the diversity in all samples adequately? Please provide more evidence that you have robust datasets.
The presented figure in the response letter is not convincing. It clearly shows that many samples reach saturation only with 10.000 reads. Thus, 500 reads are likely not sufficient to obtain robust information on Shannon diversity. What appears more convincing is the statement that alpha diversity was unaffected by read number. It would also be important to evaluate whether samples with low read numbers represent a specific set of samples, as this may lead to biased conclusions.

Authors response: To address this concern, we used different cut-off to rarefy our samples. We evaluated whether changes in read depth, particularly low read number, could affect our results. Specifically, we assessed the relationship between sequencing depth and Shannon diversity. As shown in the figure bellow, we found no significant correlation between read number and Shannon diversity ($R^2 = 0.07$, $p = 0.7898$), and low-depth samples were not systematically associated with any particular treatment group, suggesting that they do not introduce systematic bias.

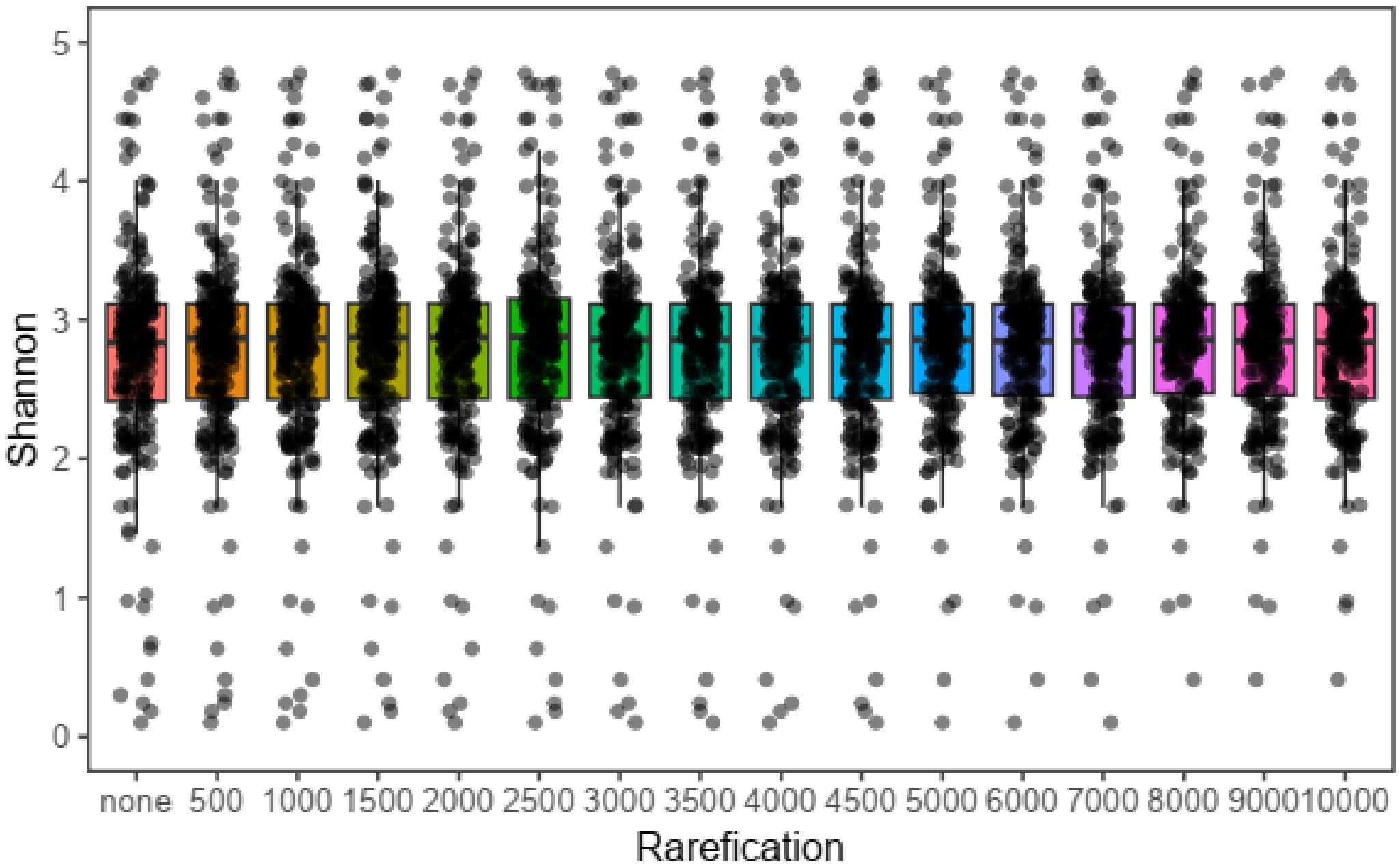

To ensure the reliability of our results, we re-ran all alpha diversity analyses using three rarefication cut-offs: 500, 5000, and 10000. As shown in the figure bellow, in general the alpha diversity trends and statistical inferences remained stable, indicating that our conclusions are robust to variation in sequencing depth.

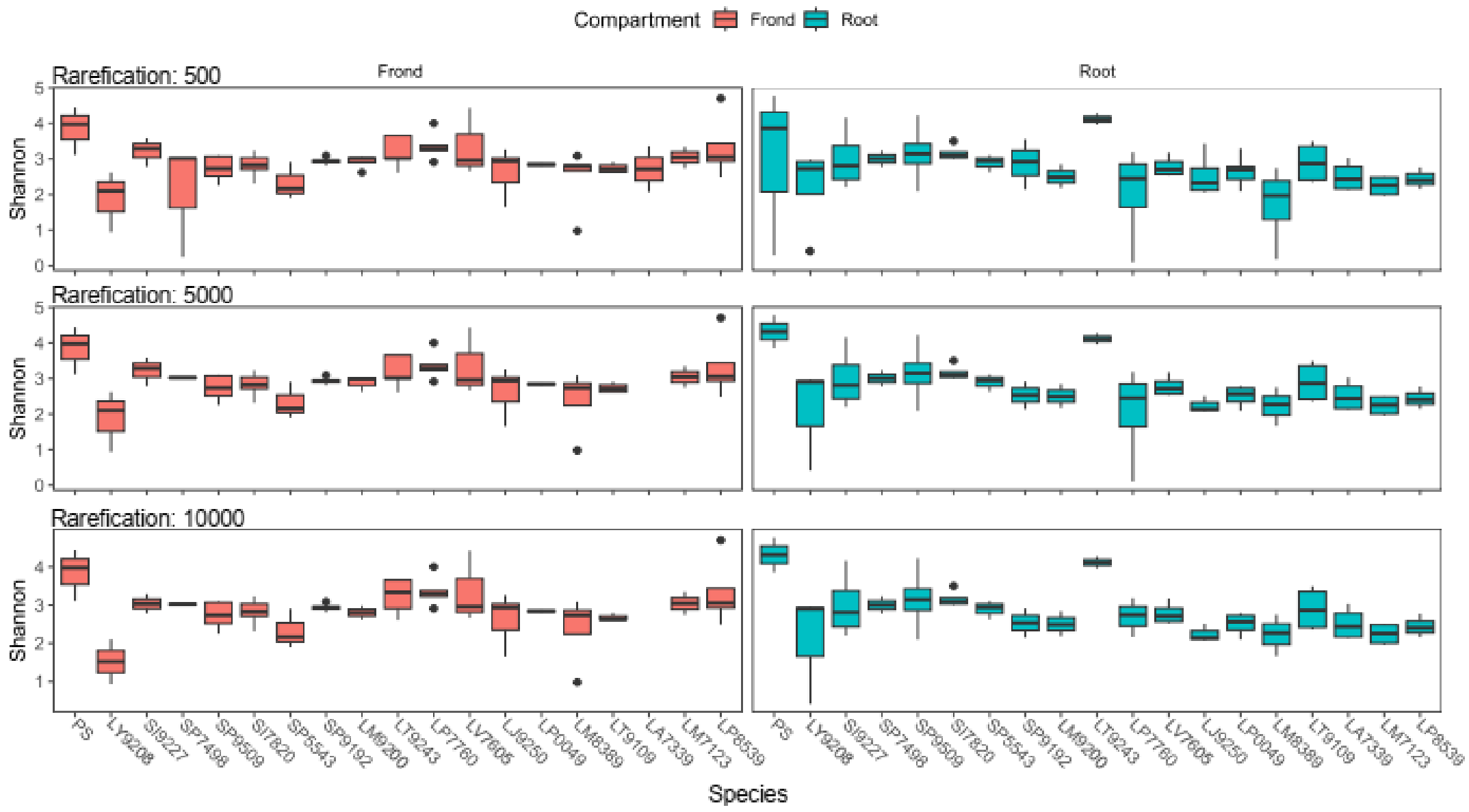

32- I do not agree to the solution that the information about data rarefaction has now been removed from the supplementary material in the revised version. I actually requested this information for all amplicon datasets in one of my other comments. This is important information for readers to allow the assessment of data quality. You should demonstrate convincingly that your data are robust despite weak samples or select a higher read number for rarefaction, even if some more samples are lost.

Authors response: The rarefaction information has been added to the material and methods sections 1.8, 2.10, and 3.4

Reviewer #1 comment 46

33- l. 284-289 and Fig. 3b: does the clustering seen in Fig. 3b support the grouping of correlated variables into three groups? Can you specify what criteria or metrics were used to define the three groups in the clustering?

Please add this information about the definition of 3 clusters in the supplementary methods section.

Authors response: The number of clusters was determined using the 'Elbow method' for k-means clustering in R, a heuristic that indicates the optimal number of clusters. This information has been added to the material and methods section 4.5

Reviewer #1 comment 51
34- l. 358-360: Why is trimethyl-lysine highlighted here and subsequently in the focus of this study? Figure 4c lacks important information to understand this (see comments below) and Fig. S12a suggests that other components showed even stronger inhibitory effects than this one across the tested plant species. Further, Fig. S12b does not indicate a survival problem related to this compound, compounds such as glutaric acid appear to be much more detrimental. Where does growth reduction end and toxicity begin? Why not going for pyrone, which reduces growth as well? The reason for a focus on trimethyl-lysine as key component requires more convincing justification or better explanation of the results.
The manuscript is now very clear regarding the focus on trimethyl-lysine. However, I wonder if some of the other compounds may likewise play a role, but are now excluded from any further discussion, simply based on the fact that their identification is not as robust? This aspect could be worth being discussed. Is there more to discover in the future, or does the trimethyl-lysine explain it all?

Authors response: We thank the reviewer for following up on this discussion. In this work, we focused on characterising the effects of the two putative metabolites that are commercially available, whose identities were assigned with confidence level ID2 in our analyses. This approached limited our study to two putative metabolites: L-citrulline and N^6,N^6,N^6-Trimethyl-L-lysine. We confirmed that the exogenous addition of N^6,N^6,N^6-Trimethyl-L-lysine significantly reduced the total area of plant fronds (Fig. 4c and Extended Data Fig. 23). In contrast, we did not observe a significant effect of L-citrulline on the growth of the plant species analysed (Fig. 4c and Extended Data Fig. 23), and therefore focused exclusively on the characterisation of N^6,N^6,N^6-trimethyl-L-lysine.

We cannot exclude the possibility that other compounds, not identified with sufficient confidence using the non-targeted metabolomics pipeline employed, may also play a role in the molecular mechanism described in this paper. We hope that the list of metabolite features identified in this work will help guide the discovery of additional modulatory compounds in future research. We anticipate that further modulatory compounds influencing the interaction between the microbiota and the root microhabitats will be identified.

Reviewer #1 comment 113
35- Fig. S10: is a selection of metabolites shown here or all?
Refers to Fig. S11 now; the question remains whether the authors focus on a selection of metabolites here or if the figure includes all compounds that were identified. If a selection was made, based on which criteria? This info could be added to the legend.

Authors response: We thank the reviewer for this comment. We included in this figure all identified compounds, except for four that were excluded (See Table S5). These were removed due to issues such as poor peak shape, higher-than-expected ppm error, MS/MS mismatch, absence of MS/MS data, or only partial of fragment matching. These compounds are highlighted in red in Table S5.

Reviewer #1 comment 115
36- Fig. S11b-d: why are these heatmaps so blurry? Each metabolite should have a specific abundance in each of the plant species, color gradients between adjacent entries do not appear meaningful.

Authors response: We apologise for the poor quality of the previous heatmaps, which we attributed to the software used for their visualisation. In the revised manuscript, this issue has now been resolved.

Reviewer #1 comment 122
37- Fig. S13b and c: show again these diffusive color gradients that do not appear meaningful.
Just to clarify, some of the heatmaps in the preceding word document (which I opened with Microsoft Word) had smooth colour transitions from one field/tile to the surrounding ones, rather than fields/tiles with clearly defined borders; the new version is not affected by this problem. I recommend paying attention that this does not happen again.

Authors response: Thanks for pointing this out. We will pay special attention to this issue when preparing the documents.

Reviewer #3

1- I congratulate the authors for addressing the comments and remarks made during the previous review with seriousness. The manuscript is now much improved.

Authors response: We sincerely thank the reviewer again for their valuable comments and suggestions, which have greatly contributed to improving our manuscript.

Reviewer #4

1- I appreciate for having addressed most of the comments, including the detailed description of the metabolomics methodology, the submission of raw data to a public repository, and the careful manual curation to ensure inclusion of only confidently annotated metabolites. I have a few minor comments:

Authors response: We gratefully thank the reviewer for their valuable comments and suggestions, which have greatly helped enhance our manuscript.

2- Table S4: The table is unclear. It appears to list isotopically labeled internal standards, but the manuscript does not describe how these standards were used during data processing. Their role in normalization or in validating annotated metabolites is not mentioned. If the standards were not incorporated into the analysis, it may be best to remove this table.

Authors response: We added a description of how these isotopically labelled internal standards were used during data processing to the materials and methods section. Now it reads: "For targeted analysis, metabolites were identified by comparing experimental spectra with those of authentic isotopically labelled internal reference standards analysed in-house using the same LC-MS/MS methods" and "A compound was considered positively identified when the detected *m/z* was within ≤ 5 ppm or 0.001 Da of the theoretical value and the RT was within ≤ 0.5 min of the corresponding isotopically labelled internal standard".

3- Table Numbering: In the Table S5 file, the table is referred to as Table S4 for POS mode and Table S6 for NEG mode. Please correct these inconsistencies.

Authors response: Corrected

4- Extended Data Fig. 11:
o The legend is incorrect—Panel a corresponds to metabolites detected in negative mode, and Panel b corresponds to positive mode.
o Some metabolites removed from Table S5 due to low-confidence annotation are still shown in the figure (e.g., tryptophan in NEG, panel a). Please remove these entries from the figure.

Authors response: Thank you for pointing this out. It has now been corrected.

5- Result:
The paragraph beginning "To identify metabolites that modulate microbiota driven root anatomical plasticity…" remains unclear. As written, it implies that all 341 metabolites were confidently annotated, although only one of the final 15 is confidently identified. Please revise for clarity.

Authors response: Following the reviewer's comment, we have reworded the paragraph for clarify. It now reads: "To identify metabolites that modulate microbiota-driven root anatomical plasticity, we first selected metabolite features that differed significantly between axenic plants and plants inoculated with the bacterial synthetic community (Extended Data Fig. 12b-c), identifying 341 putatively annotated metabolites (Extended Data Fig. 12c). From this set, we further narrowed this selection to 15 putative metabolites whose concentrations were significantly negatively correlated with root anatomical complexity across the *Lemnaceae* species studied (Extended Data Fig. 12d, 12e, and Table S6). Among these 15 candidates, only two metabolites could be identified with confidence level ID2, whereas the

others remain putatively annotated based on spectral features. This negative correlation suggests that these compounds might differentially influence root plasticity depending on the root's inherent anatomical complexity. High levels of these compounds might limit microbiota-driven plasticity in roots that are inherently more complex and exhibit lower concentrations of these metabolites".

6- In addition, please provide a supplemental table listing all metabolites that were confidently annotated for this experiment. This would help evaluate whether additional metabolites related to carnitine metabolism (especially lysine and methionine, precursors of TML) are present and further support the authors' conclusions.

Authors response: Following the reviewer's recommendation, we have prepared a new Table S6 listing the 341 metabolite features that show significantly different accumulation in plants inoculated with the bacterial synthetic community compared to axenic plants (See Extended Data Fig. 22). The level of identification used to annotate each metabolite feature is also indicated in the table. Among the metabolite features listed, we identified additional metabolites related to carnitine metabolism, such as methionine, a precursor of TML.

A point-by-point response to the remaining referee concerns

REVIEWERS' COMMENTS

Reviewer #1 (Remarks to the Author):

1-The authors have adequately addressed my concerns and I congratulate to this comprehensive study with very valuable new insights.

Authors' response: We reiterate our gratitude to the reviewer for the thoughtful comments and suggestions provided during the revision process. They have significantly improved our manuscript, making it much stronger and more robust.

2- I only spotted a last minor grammatical issue in the manuscript text: in l. 55, the word "was" should be deleted.

Authors' response: This mistake has been corrected.

3- However, regarding the data provided on Github, it appears that the rearrangement of plots in figures and of complete figures in manuscript and supplement during the review process has not been considered in this data repository. Thus, data and scripts for several of the presented figures cannot be found anymore. It appears that it has not yet been (fully) updated.

Authors' response: We thank the reviewer for checking the code availability. The GitHub information has been updated and organised according to the new version of the manuscript.

Reviewer #1 (Remarks on code availability):

1- I only checked for availability and identified the problem mentioned in my response to the authors.

Authors' response: Please see the comment above.

Reviewer #4 (Remarks to the Author):

1- All of my comments have been addressed, and the revisions are satisfactory. Thank you for your careful attention to the feedback.

Authors' response: We are incredibly grateful to the reviewer for their thoughtful comments, insights, and suggestions throughout the revision process. Addressing feedback has significantly enhanced the overall quality of our manuscript.
