# Supplementary material for: The metabolic and anatomical complexity of root microhabitats modulate their interaction with the microbiota
Source: Nat Commun. 2026 Aug 13;17:9738. doi: 10.1038/s41467-026-76440-4 (PMC13562559; doi:10.1038/s41467-026-76440-4)
Supplement: Supplementary file 3 [file 41467_2026_76440_MOESM1_ESM.pdf]

Supplementary Material for

The metabolic and anatomical complexity of the roots modulate their interaction with the microbiota

Juan P. Frene, Valéria Custódio, Helena Rouco, Sandra Martinez-Jarquin, Yi-Qun Gao, Vincenzo di Bari, Niokhor Bakhoum, Trent R. Northen, Benjamin P. Bowen, Katherine B. Louie, Katerina Velchova, Alexander Ware, Anthony Bishopp, Alvaro Mata, and Gabriel Castrillo

Correspondence to gabriel.castrillo@nottingham.ac.uk

Supplementary Figures

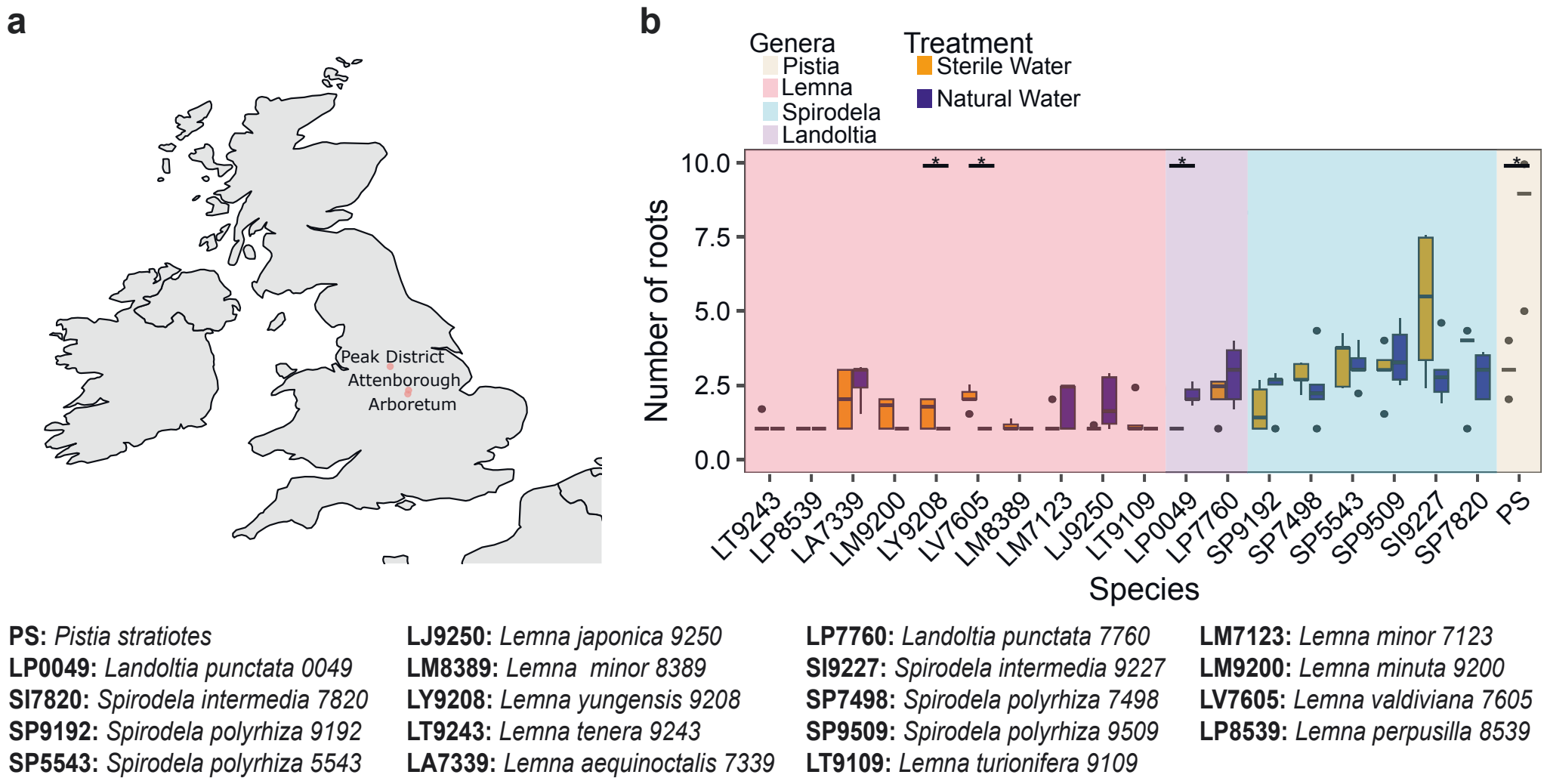

Supplementary Figure 1. The root number gradient is conserved in plants grown in natural water. a. Map showing natural water collection sites (Peak District (53.365604, -1.697046), Attenborough Nature Reserve (52.904268, -1.233926), and the Arboretum of Sutton-Bonington Campus, University of Nottingham (52.834139, -1.253199)) across the UK used in this work for microbiota studies and the isolation of bacterial strains. **b**. Box plot showing the number of roots quantified in the different *Lemnaceae* species and *Pistia stratiotes* plants grown in sterile (orange) and natural (blue) waters. Coloured shading represents different plant genera. In the box plots, the centre lines indicate the median, the box limits represent the first and third quartiles, and the whiskers extend to the most extreme values within 1.5 × the interquartile range (IQR). A total of n = 5 independent biological replicates were used. Significant differences between treatments within each plant species were determined using a two-sided *t*-test ($p < 0.05$).

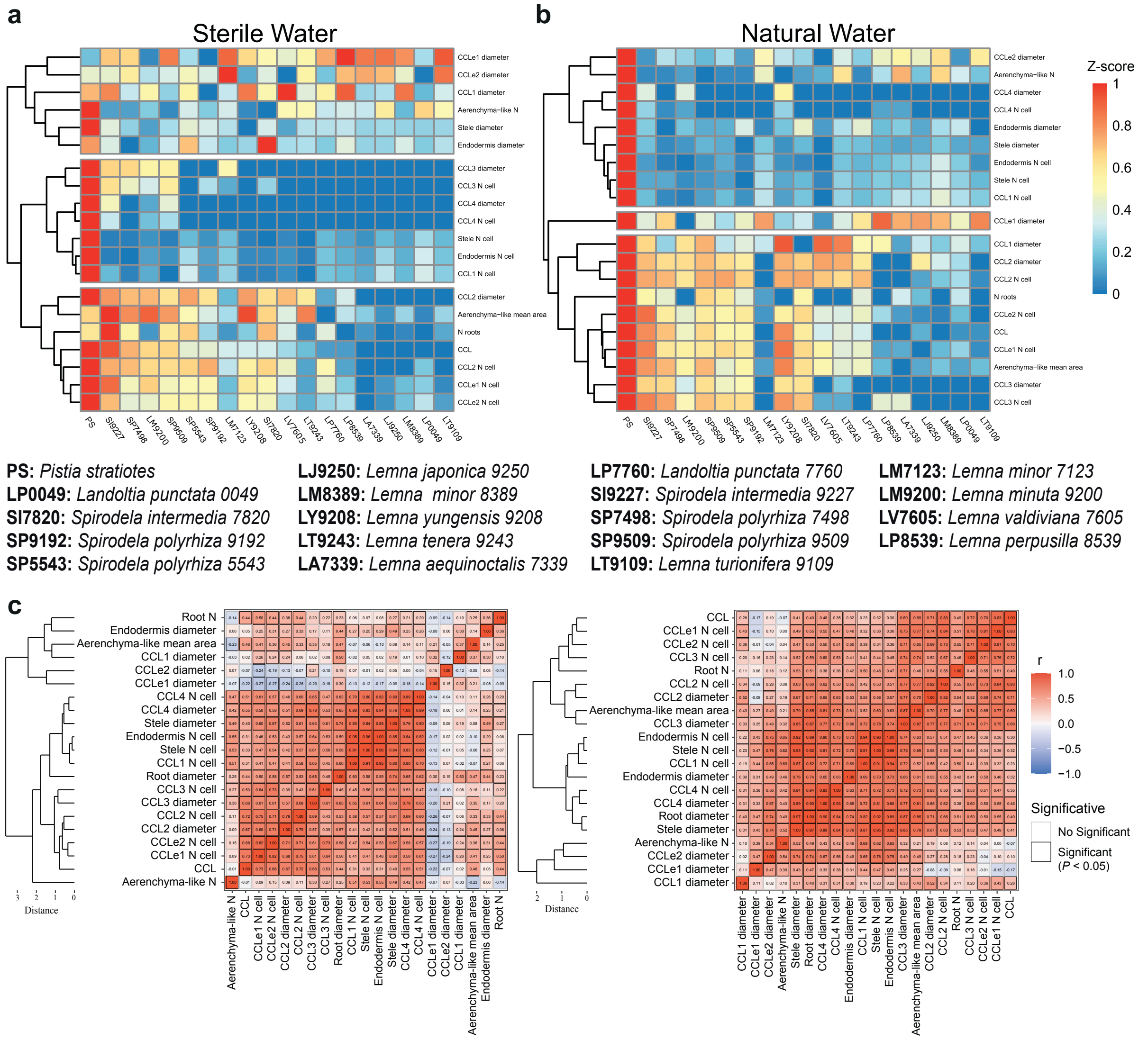

Supplementary Figure 2. Root anatomy changes in plants grown in a natural water. a. Heatmap showing the normalized (*z*-score) anatomical features measured in roots from a collection of *Lemnaceae* and *Pistia stratiotes* plants grown in sterile and **b.** natural water (see Material and Methods section for details). For each species, the normalised mean value of n = 5 independent biological replicates, was clustered according to anatomical features. **c**. Heatmap showing the Pearson's correlation coefficient from each pairwise comparison between the root anatomical features quantified in plants grown in sterile (left) and natural (right) water. Values have been clustered according to the features. Correlation coefficient (r) values are shown within each grid in each case. Significant correlations (*p*-value < 0.05) are highlighted in black.

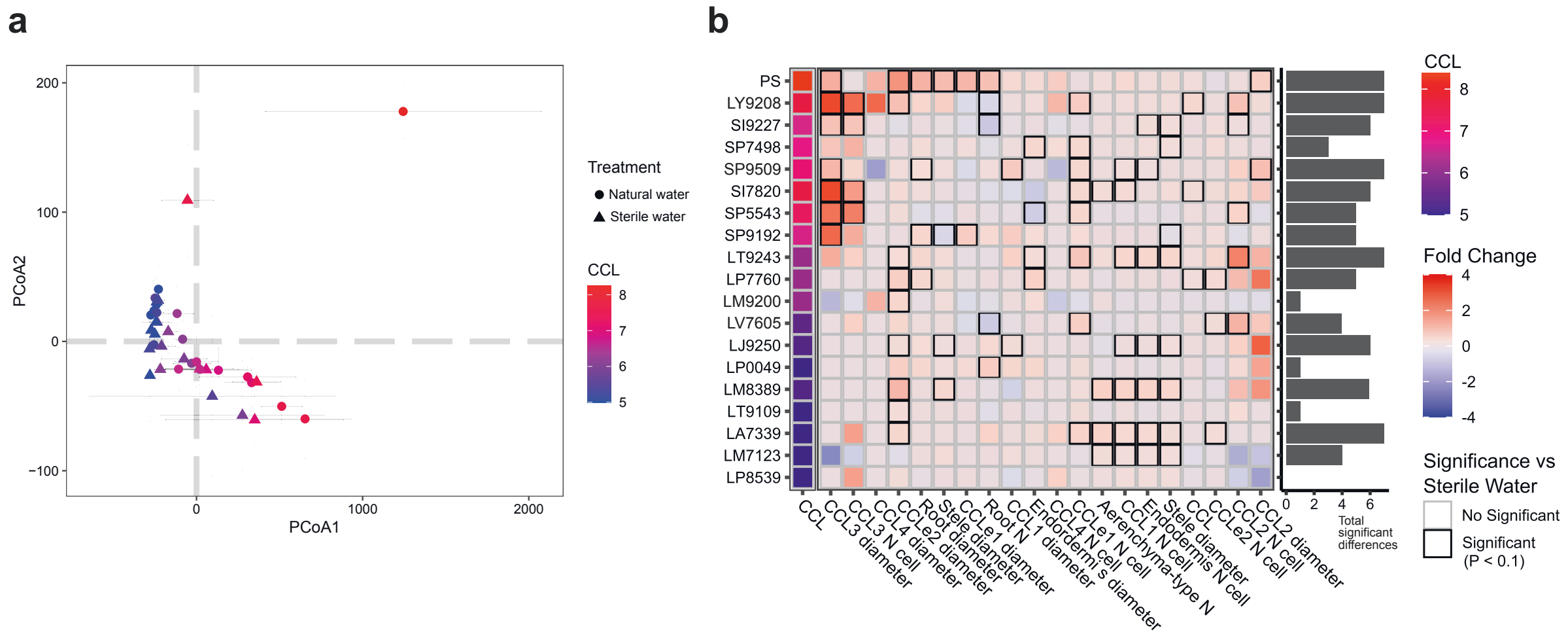

PS: *Pistia stratiotes*
LP0049: *Landoltia punctata 0049*
SI7820: *Spirodela intermedia 7820*
SP9192: *Spirodela polyrhiza 9192*
SP5543: *Spirodela polyrhiza 5543*
LJ9250: *Lemna japonica 9250*
LM8389: *Lemna minor 8389*
LY9208: *Lemna yungensis 9208*
LT9243: *Lemna tenera 9243*
LA7339: *Lemna aequinoctalis 7339*
LP7760: *Landoltia punctata 7760*
SI9227: *Spirodela intermedia 9227*
SP7498: *Spirodela polyrhiza 7498*
SP9509: *Spirodela polyrhiza 9509*
LT9109: *Lemna turionifera 9109*
LM7123: *Lemna minor 7123*
LM9200: *Lemna minuta 9200*
LV7605: *Lemna valdiviana 7605*
LP8539: *Lemna perpusilla 8539*

Supplementary Figure 3. Anatomical changes in the roots of plants grown in natural water were less evident in species with simpler inherent root anatomy. a. Principal coordinate analysis (PCoA) of the 21 root anatomical traits identified in Supplementary Figure 2, showing the root anatomical traits projection of individual plant species grown in natural (circle) or sterile (triangle) waters. Each point represents the mean of n = 5 independent biological replicates and is coloured according to the number of root cell layers (CCL) present in each plant. Error bars represent the standard deviation (SD). **b.** Heatmap analysis showing the standardised fold changes in root anatomical traits between plants grown in natural and sterile waters. The values have been organised according to the number of root cell layers (CCL) present in each plant, a proxy for root anatomical complexity. Values significantly different from the sterile water control, identified using a two-sided *t*-test (*p*-value < 0.1), are outlined in black. The bar graph on the right shows the total number of significantly different anatomical features between the roots of plants grown in natural water compared to those of the same plant grown in sterilised water. Note that the number of significantly different features between plants grown in natural and sterilized water decreases towards plants with less complex roots.

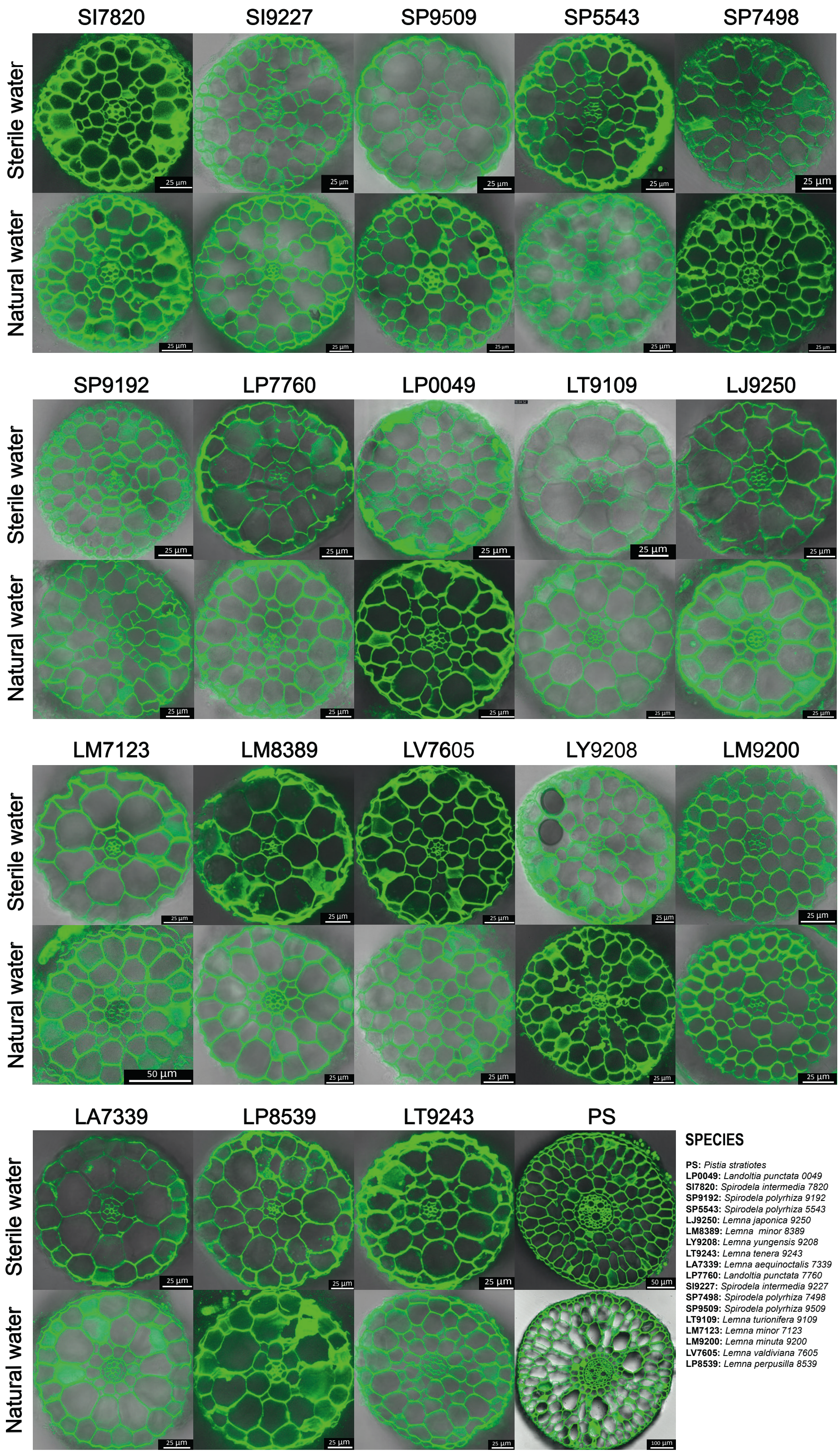

Supplementary Figure 4. Representative images of the root anatomy of all plant species used in this study, grown either in sterile water or in natural water containing the native microbiota. Images were acquired using a Leica SP5 confocal microscope with a 40× objective (NA = 0.8), using 405 nm excitation and a 620–630 nm emission band-pass filter. Scale bars are shown in each image. Representative images are from n = 5 independent biological replicates.

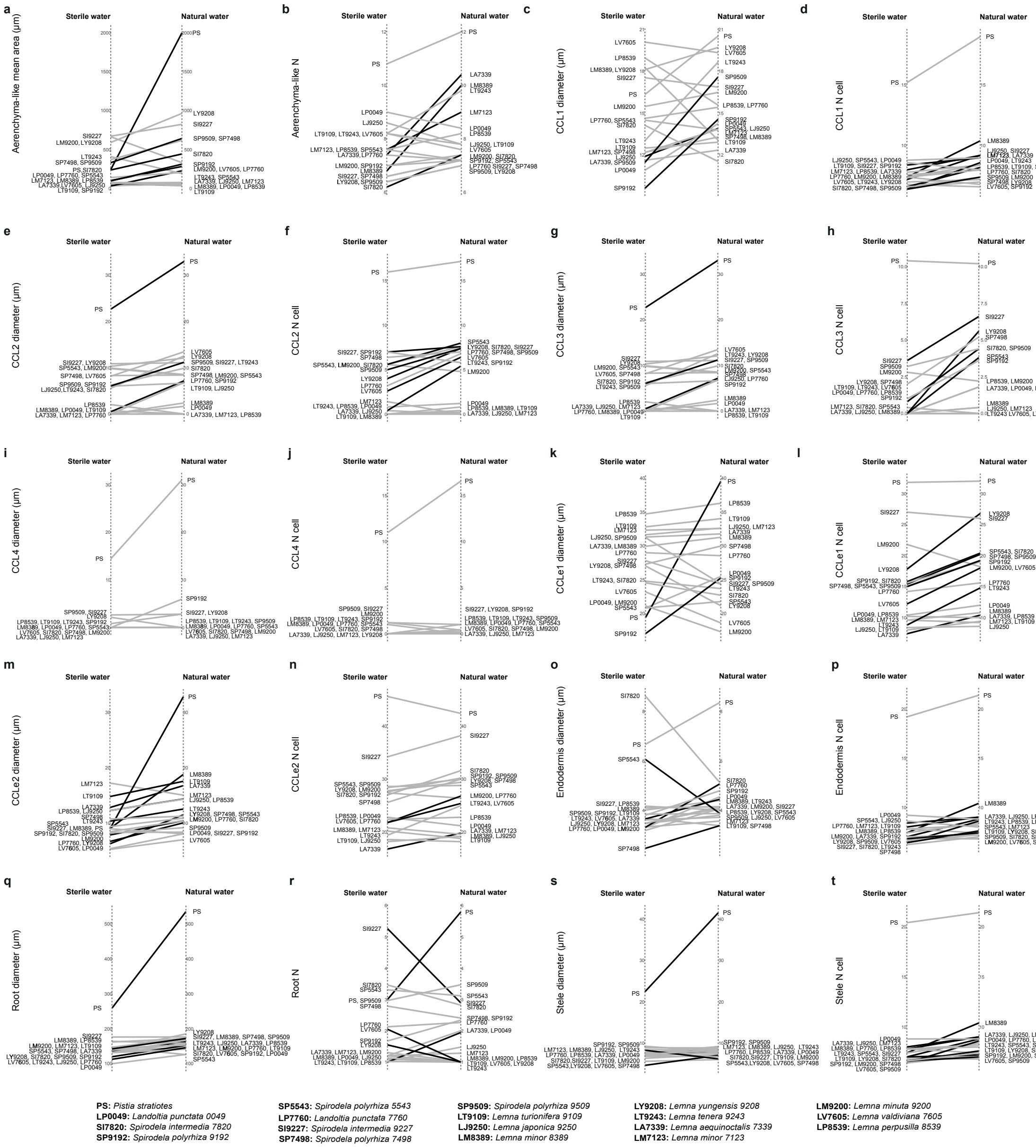

Supplementary Figure 5. The complexity of root anatomy changes in plants grown in natural water with the microbiota present. a. Comparative analysis of aerenchyma-like mean area. **b.** Number of aerenchyma-like structures. **c.** Diameter of the cortical cell layer 1 (CCL1)**. d.** Number of cells in the cortical cell layer 1 (CCL1). **e.** Diameter of the cortical cell layer 2 (CCL2). **f.** Number of cells in the cortical cell layer 2 (CCL2). **g.** Diameter of the cortical cell layer 3 (CCL3). **h**. Number of cells in the cortical cell layer 3 (CCL3). **i.** Diameter of the cortical cell layer 4 (CCL4). **j.** Number of cells in the cortical cell layer 4 (CCL4). **k.** Diameter of the external cortical cell layer 1 (CCLe1)**. l.** Number of cells in the external cortical cell layer 1 (CCLe1). **m**. Diameter of the external cortical cell layer 2 (CCLe2). **n**. Number of cells in the external cortical cell layer 2 (CCLe2). **o**. Endodermis diameter. **p**. Number of cells in the endodermis. **q**. Root diameter. **r**. Number of roots. **s**. Stele diameter, and **t**. Number of cells in the stele. Each value represents the mean of n = 5 independent biological replicates. The line connecting the two points indicates the difference in the root trait between sterile and natural water. Black lines indicate statistically significant differences ($q < 0.05$).

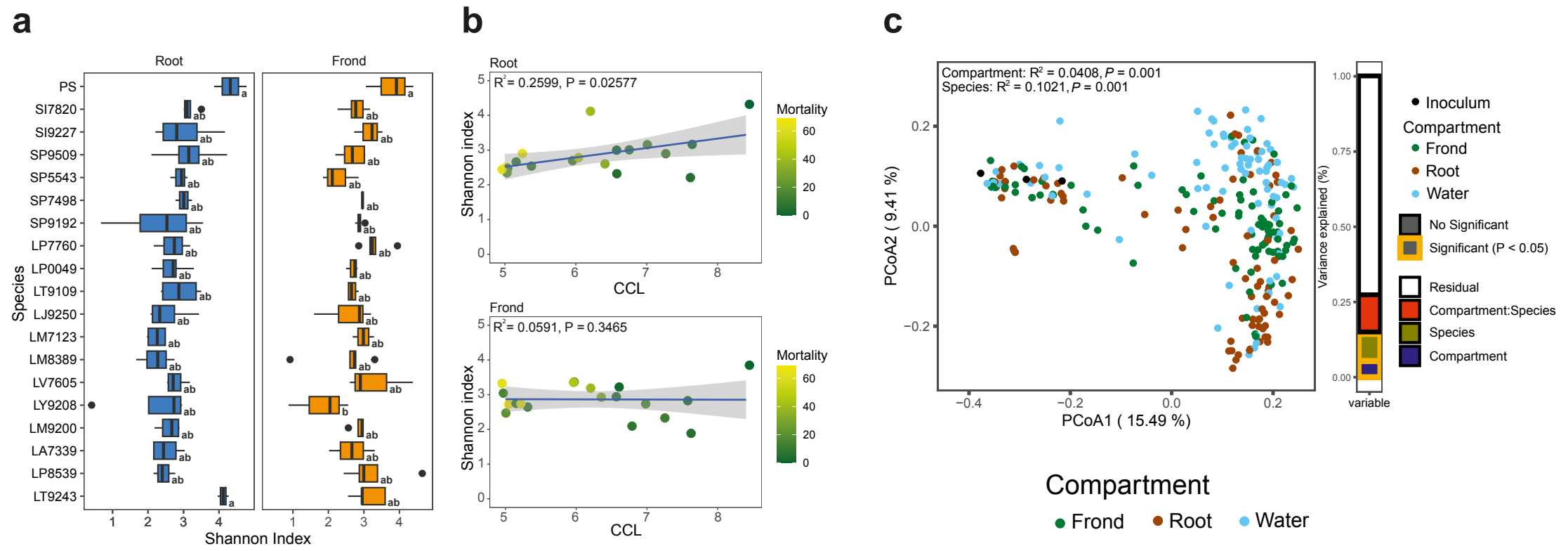

PS: *Pistia stratiotes*
LP0049: *Landoltia punctata 0049*
SI7820: *Spirodela intermedia 7820*
SP9192: *Spirodela polyrhiza 9192*
SP5543: *Spirodela polyrhiza 5543*
LP7760: *Landoltia punctata 7760*
SI9227: *Spirodela intermedia 9227*
SP7498: *Spirodela polyrhiza 7498*
SP9509: *Spirodela polyrhiza 9509*
LT9109: *Lemna turionifera 9109*
LJ9250: *Lemna japonica 9250*
LM8183: *Lemna minor 8183*
LY9208: *Lemna yungensis 9208*
LT9243: *Lemna tenera 9243*
LA7339: *Lemna aequinoctalis 7339*
LM7123: *Lemna minor 7123*
LM9200: *Lemna minuta 9200*
LV7605: *Lemna valdiviana 7605*
LP8539: *Lemna perpusilla 8539*

Supplementary Figure 6. The root's anatomical complexity could control root colonization by specific bacterial taxa. a. Graph shows the alpha diversity, estimated using the Shannon Diversity index, in roots and fronds across plant species used in this work. We used n = 5 independent biological replicates, except for the root samples of LM9200, LM8183, LP7760, and LT9243 and the frond samples of SI9227 and LA7339, where n = 4. **b** Linear regression analysis between (top) root and (bottom) frond alpha diversity indices and the number of root cell layers in different plant species grown in natural water. The proportion of variation in alpha diversity explained by the number of root cell layers (R^2), along with the associated *p*-values, is shown in each panel. Plant species (points) are coloured according to the percentage of plant mortality. Shaded areas represent the standard deviation (SD). **c.** Principal coordinate analysis (PCoA) of microbiota composition showing the projected microbiota assembly across the different plant species used within the sample fractions (water, root, frond) and the initial water (inoculum) used. Plants were grown in natural water. Different colours represent different sample fractions, and the inoculum samples are represented in black. The bar on the right denotes the percentage of the variance explained by the experimental variables using a Permutational Multivariate Analysis of Variance (PERMANOVA). Significant variables are highlighted in orange (*p*-value < 0.05). Fraction of the variance explained (R^2 and *p*-values, PERMANOVA) by the variables sample fraction ('compartment') and the plant species used ('species') are shown within the panel. We used n = 5 independent biological replicates, except for the root samples of LM9200, LM8183, LP7760, and LT9243; the frond samples of SI9227 and LA7339; and the water samples related to LY9208, SI9227, and SP9192, where n = 4.

a

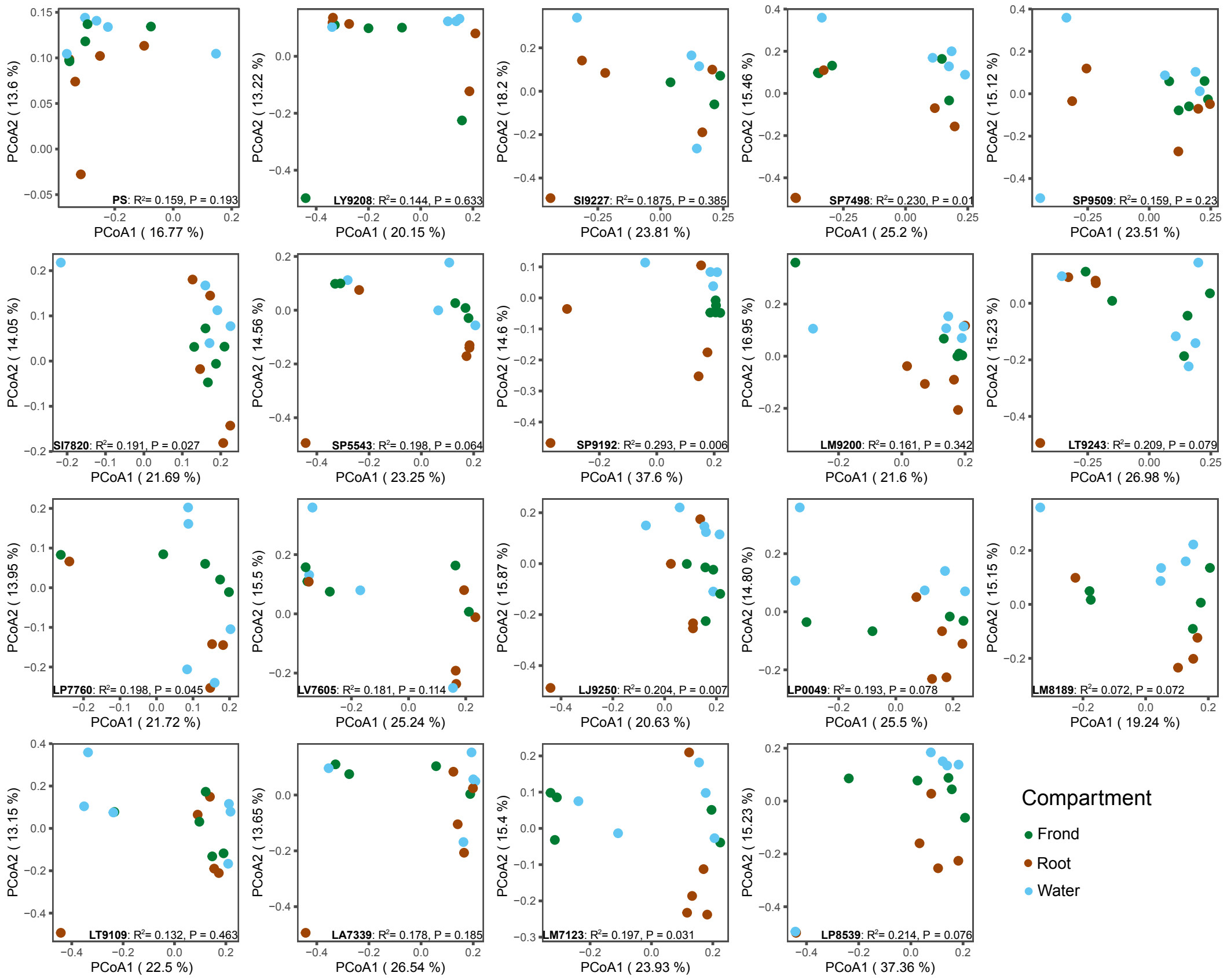

PS: *Pistia stratiotes*
LP0049: *Landoltia punctata 0049*
SI7820: *Spirodela intermedia 7820*
SP9192: *Spirodela polyrhiza 9192*
SP5543: *Spirodela polyrhiza 5543*
LP7760: *Landoltia punctata 7760*
SI9227: *Spirodela intermedia 9227*
SP7498: *Spirodela polyrhiza 7498*
SP9509: *Spirodela polyrhiza 9509*
LT9109: *Lemna turionifera 9109*
LJ9250: *Lemna japonica 9250*
LM8183: *Lemna minor 8183*
LY9208: *Lemna yungensis 9208*
LT9243: *Lemna tenera 9243*
LA7339: *Lemna aequinoctalis 7339*
LM7123: *Lemna minor 7123*
LM9200: *Lemna minuta 9200*
LV7605: *Lemna valdiviana 7605*
LP8539: *Lemna perpusilla 8539*

Supplementary Figure 7. Under our conditions, microbial community differences among sample fractions were less pronounced. Principal coordinate analysis (PCoA) of microbiota composition showing the projected microbiota assembly of individual plant species within the sample fractions (water, root, frond). Plants were grown in natural water. Different colours represent different sample fractions. The plant species identifier and the variance explained (R^2 and *p*-values, PERMANOVA) by the variable sample fraction ('compartment') are shown within each panel. We used n = 5 independent biological replicates, except for the root samples of LM9200, LM8183, LP7760, and LT9243; the frond samples of SI9227 and LA7339; and the water samples related to LY9208, SI9227, and SP9192, where n = 4.

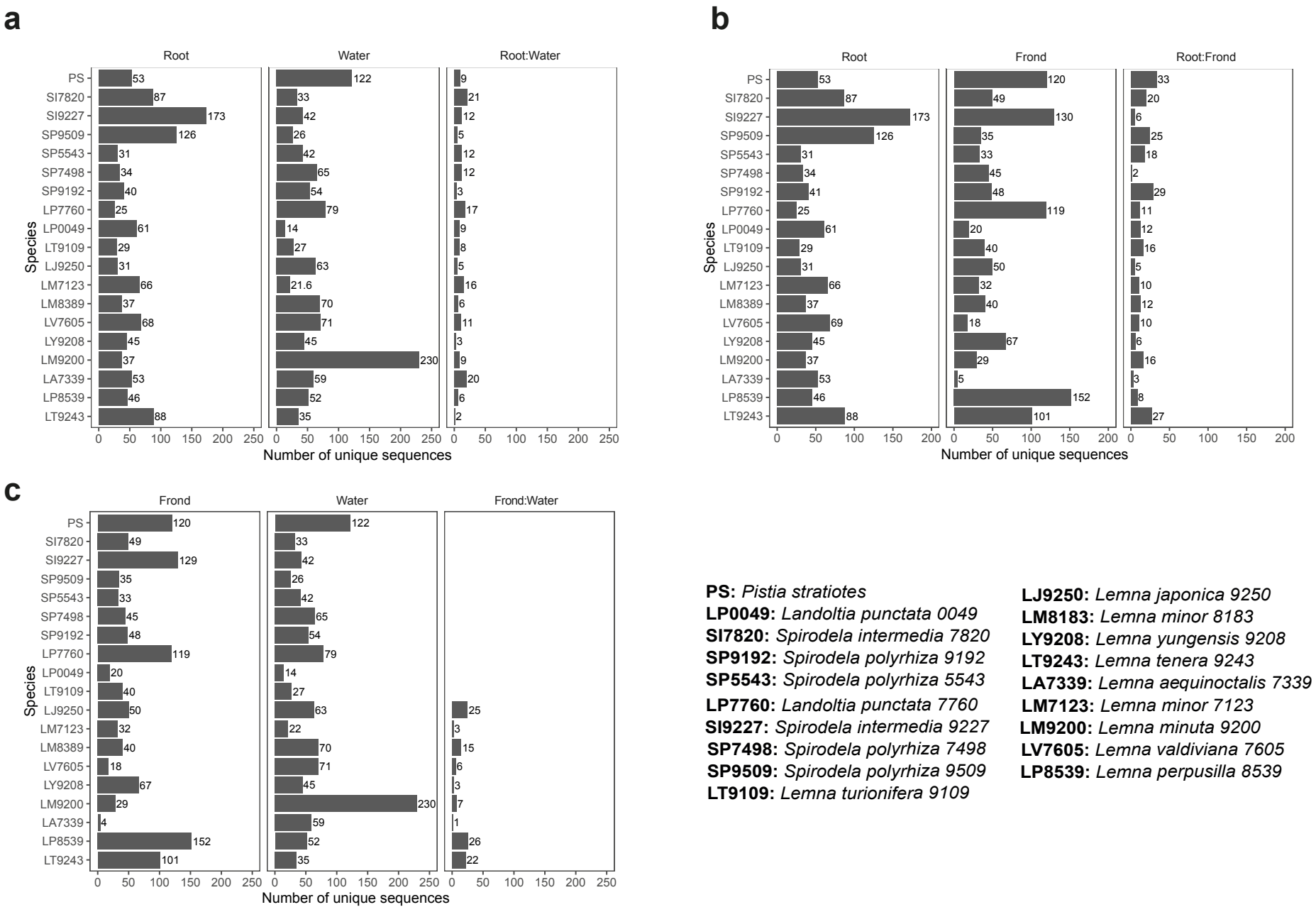

Supplementary Figure 8. Reduced overlap of unique ASVs among different sample fractions. a. Bar-graph showing the number of unique ASVs identified in roots, water, and the number of ASVs shared between roots and water, **b.** in roots, fronds, and the number of ASVs shared between roots and fronds, and **c.** in fronds, water, and the number of ASVs shared between fronds and water across the plant species used. We used n = 5 independent biological replicates, except for the root samples of LM9200, LM8183, LP7760, and LT9243; the frond samples of SI9227 and LA7339; and the water samples related to LY9208, SI9227, and SP9192, where n = 4.

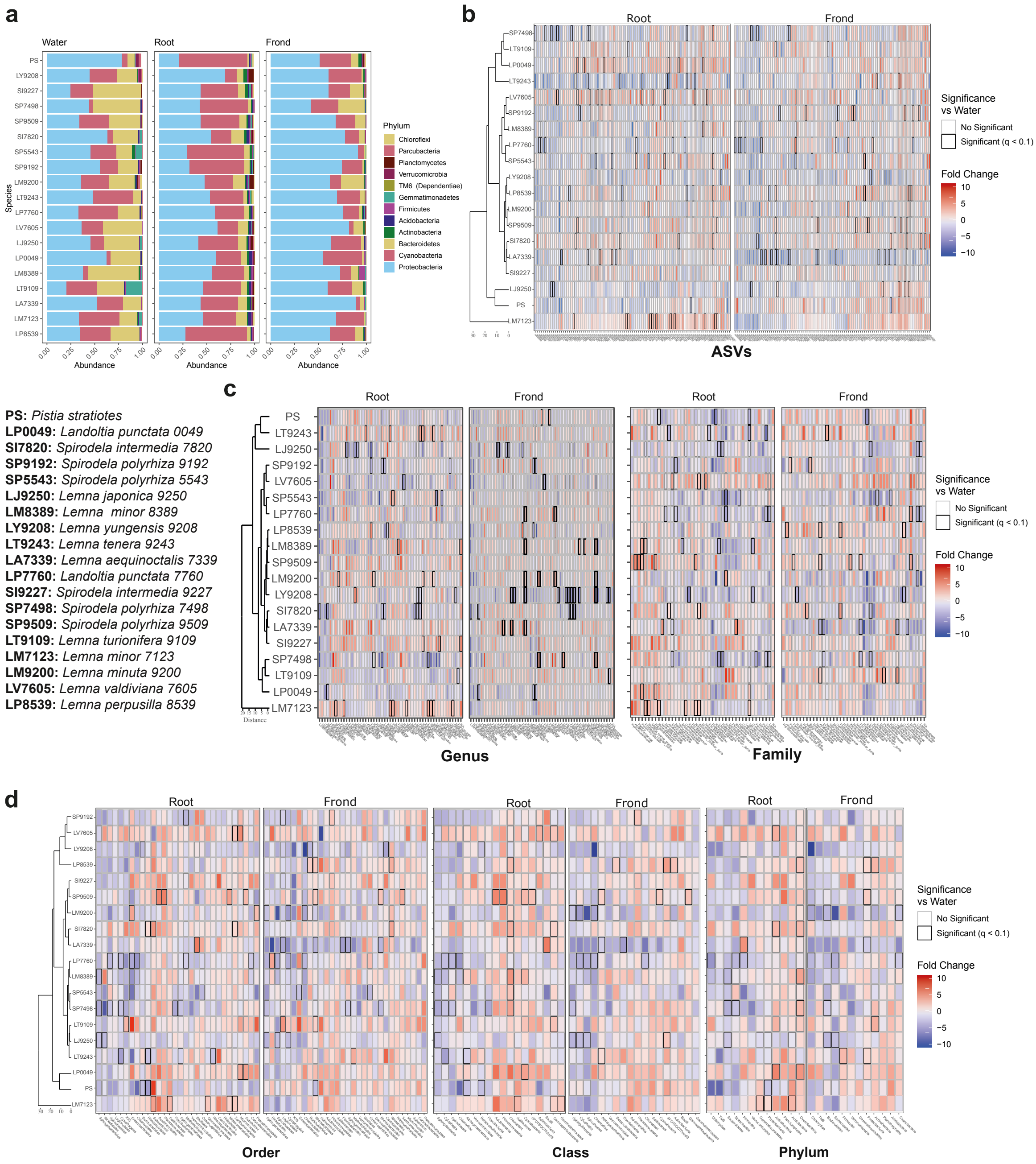

Supplementary Figure 9. The anatomical complexity of the roots influences the assembly of the microbiota. a. Phylogram showing the relative abundance profiles of the major bacterial phyla detected in water, roots, fronds of the different plant species used. Colours denote bacterial phyla. **b.** Heatmaps showing root and frond enrichment patterns (relative to water) for the 100 most abundant ASVs. **c.** the 60 most abundant bacterial genera and the 40 most abundant bacterial families, and **d.** the 30 most abundant bacterial orders, the 18 most abundant bacterial classes, and the 12 most abundant bacterial phyla colonising the roots and fronds of the plant species used in this study. Heatmap tiles are coloured according to the $\log_2$ fold change estimated from a generalized linear model comparing the abundance of each taxonomic unit (x-axis) in a given plant species (y-axis) with its abundance in water. Plant species (y-axis) are clustered, and the dendrogram indicates the hierarchical clustering of taxonomic categories, with the scale representing the distance between the identified clusters. Tiles outlined in black denote taxonomic units that are significantly enriched (red) or depleted (blue) relative to water (false discovery rate (FDR)-adjusted $q < 0.1$). We used n = 5 independent biological replicates, except for the root samples of LM9200, LM8183, LP7760, and LT9243; the frond samples of SI9227 and LA7339; and the water samples related to LY9208, SI9227, and SP9192, where n = 4.

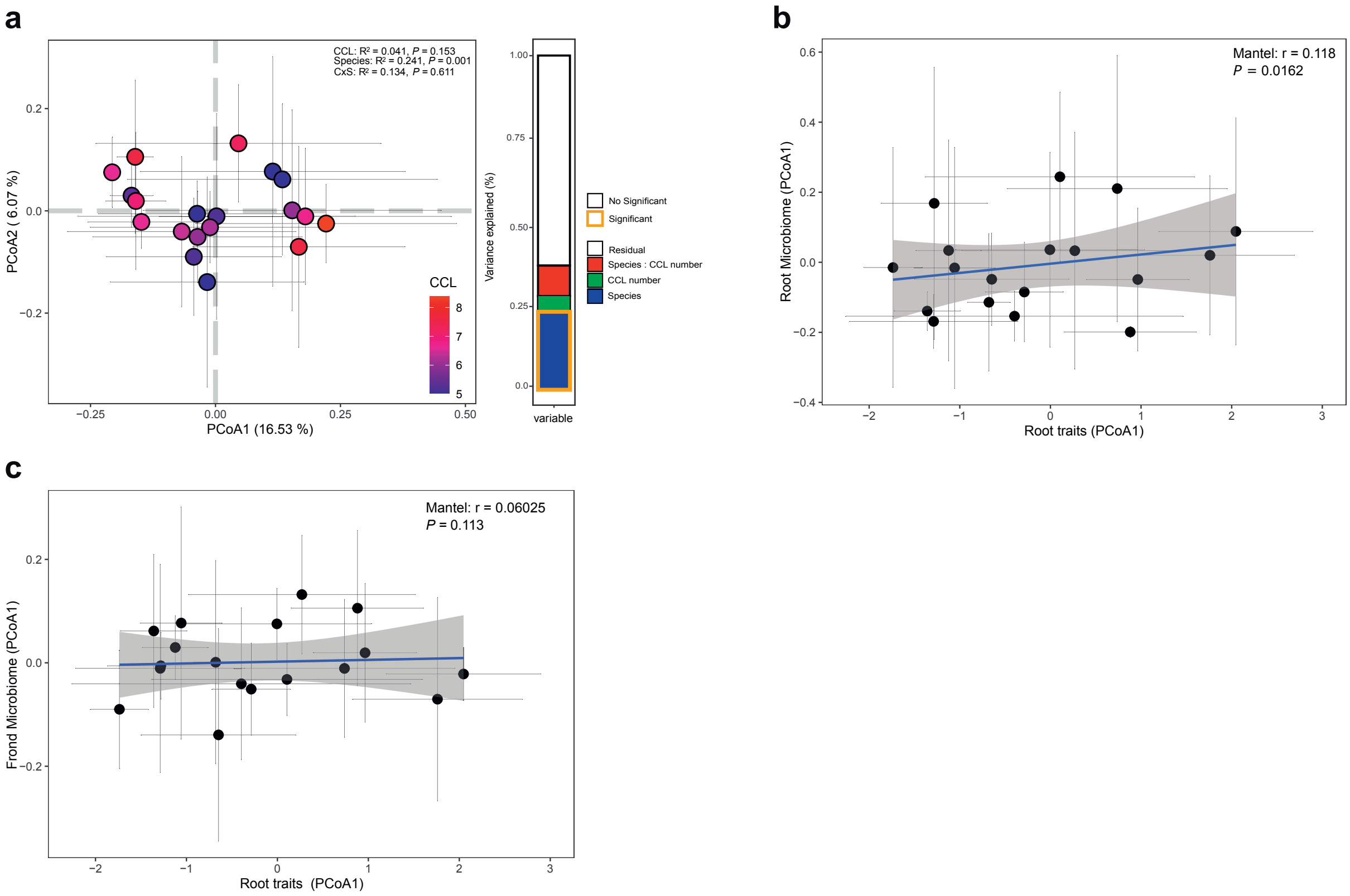

Supplementary Figure 10. The root anatomical complexity might influence the assembly of root microbial communities, but not frond microbial communities. a. Principal coordinates analysis (PCoA) showing the projection of the frond bacterial composition profiles of *Lemnaceae* species and *Pistia stratiotes* plants grown in natural water. Each point represents the mean of n = 5 independent biological replicates, except for the samples of SI9227 and LA7339 where n = 4 and is coloured according to the number of root cell layers (CCLs) present in each plant. Error bars represent the standard deviation (SD). The bar on the right indicates the percentage of variance explained by the experimental variables, estimated using Permutational Multivariate Analysis of Variance (PERMANOVA), with significant variables highlighted in orange (p-value < 0.05). **b.** Pairwise correlation analysis between a dissimilarity matrix based on root microbiota composition (PCoA1) and a dissimilarity matrix based on root anatomical traits (PCoA1), and between **c**. a dissimilarity matrix based on frond microbiota composition (PCoA1) and a dissimilarity matrix based on root anatomical traits (PCoA1) in the different plant species grown in natural water. Mantel's r statistic and the p-value, obtained from 10,000 permutations, are shown. The shaded area represents the standard deviation (SD) across biological replicates. Notably, only the dissimilarities in root anatomical traits and root microbiota composition show a significant correlation, supporting the conclusion that mechanisms controlling root anatomical plasticity might also influence root microbiota assembly.

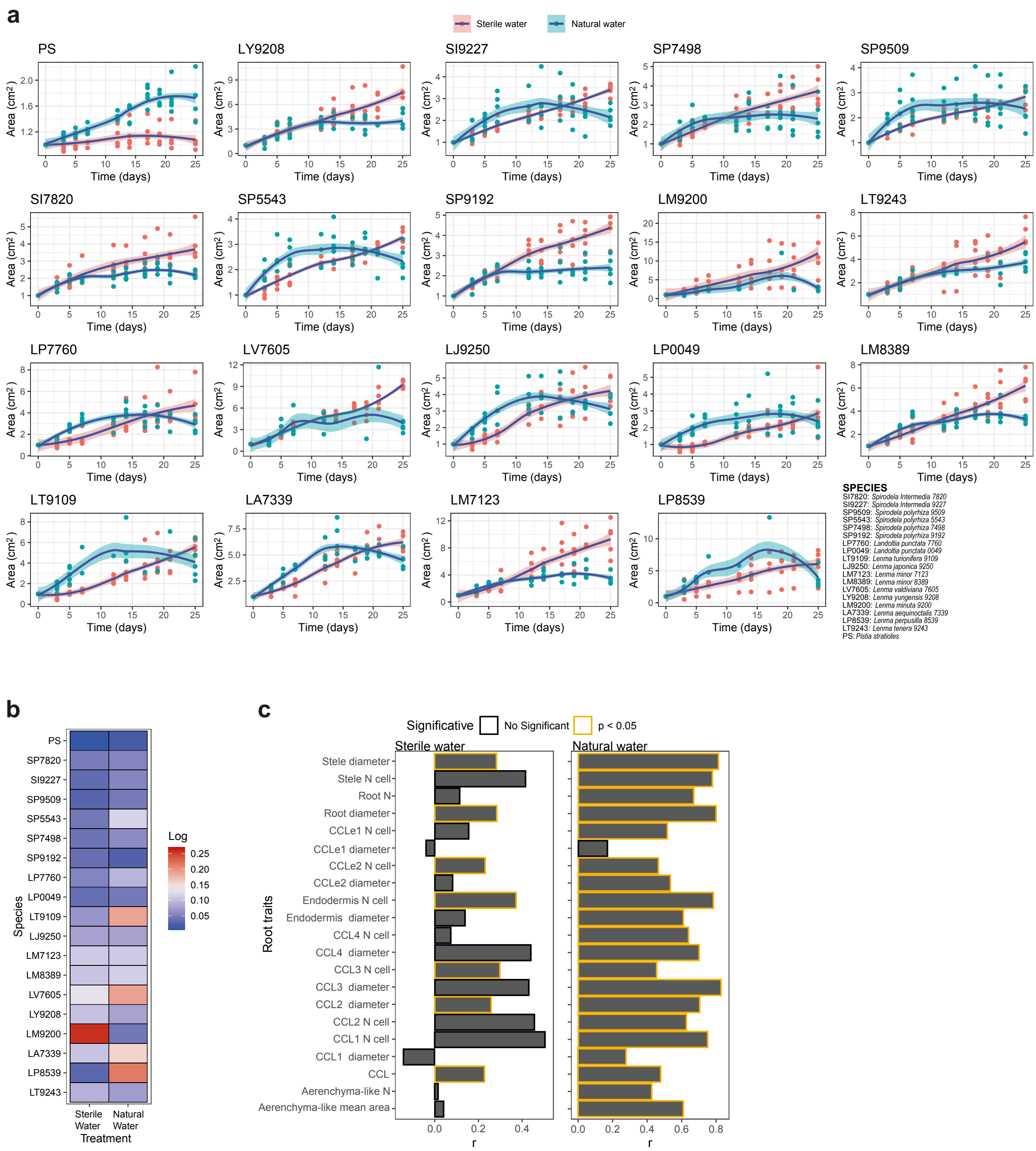

Supplementary Figure 11. The presence of microbiota impacts plant growth kinetics and root anatomical plasticity. a. Reproduction kinetics of the different plant species grown in natural water (blue) and in the same water autoclaved to eliminate the microbiota (red). We quantified the total frond area in each sample at different time points as a proxy for plant reproduction. The shaded area represents the standard deviation (SD) across n = 5 independent biological replicates. **b.** Heatmap showing the rate of plant growth change (slope) determined in the linear phase of each curve generated in panel a. Values were normalized using a logarithmic scale. We used n = 5 independent biological replicates. **c.** Bar graph showing the Pearson correlation coefficient (r) between each root feature measured across all plant species used and the rate of plant growth (panel b) in sterile or natural water. Statistically significant linear correlations are highlighted in orange ($p <$ 0.05). We used n = 5 independent biological replicates. Note the strong correlations between anatomical traits and plant growth in natural water, suggesting that microbiota-induced anatomical changes in roots could be important for plant growth under natural conditions.

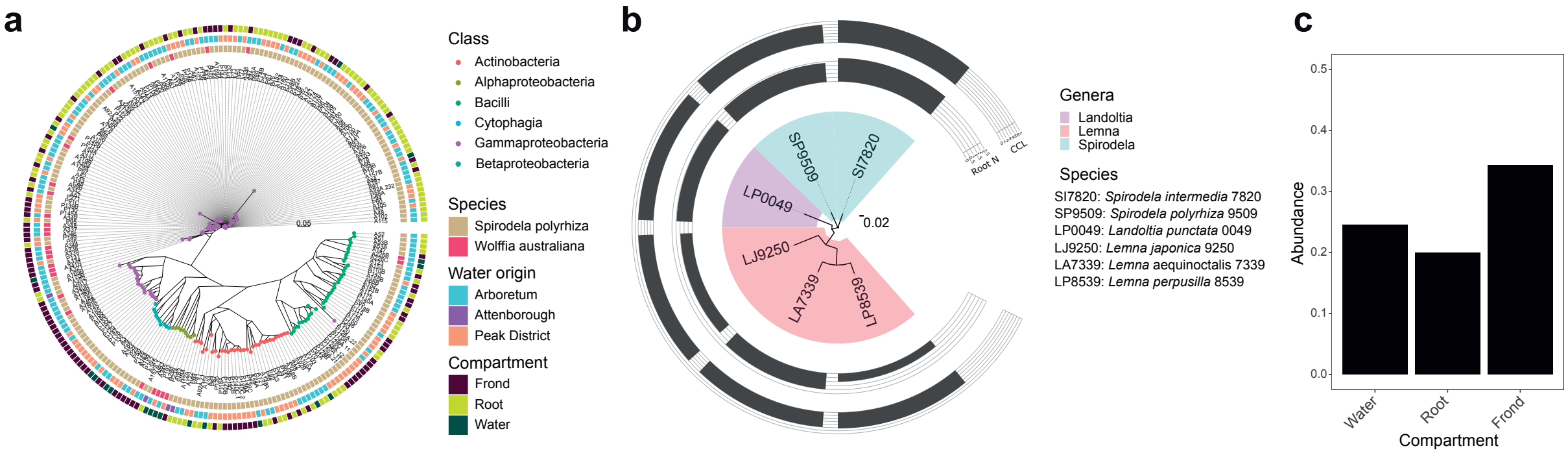

Supplementary Figure 12. Collection of bacterial strains isolated from Lemnaceae species grown in natural waters from various locations. a. Phylogenetic tree based on *16S rRNA* gene sequences of the 245 bacterial strains from the collection used in this work. Tree tips are coloured according to bacterial taxonomic classes. The inner, middle, and outer rings indicate the *Lemnaceae* species from which the bacterial strains were isolated, the origin of the water used to grow the plants for the bacterial isolation, and the sample compartments (fronds, roots, and water) from which the bacterial strains were isolated, respectively. **b.** Phylogenetic tree showing the distribution of six *Lemnaceae* species (coloured) with differing root anatomical complexity. The tree was built using variations in chloroplast *RPL16* gene sequences, which encode the L16 Ribosomal Protein, a component of the large (50S) ribosomal subunit in chloroplasts of all plants used. The inner and the outer rings represent, respectively, the total number of roots and root cell layers (CCL) observed in plants grown in sterile natural water. **c.** Bar graph showing the cumulative relative abundance, at the family level, of bacterial strains included in the synthetic community across water, root, and frond samples defined in our experiment using plants grown in natural water.

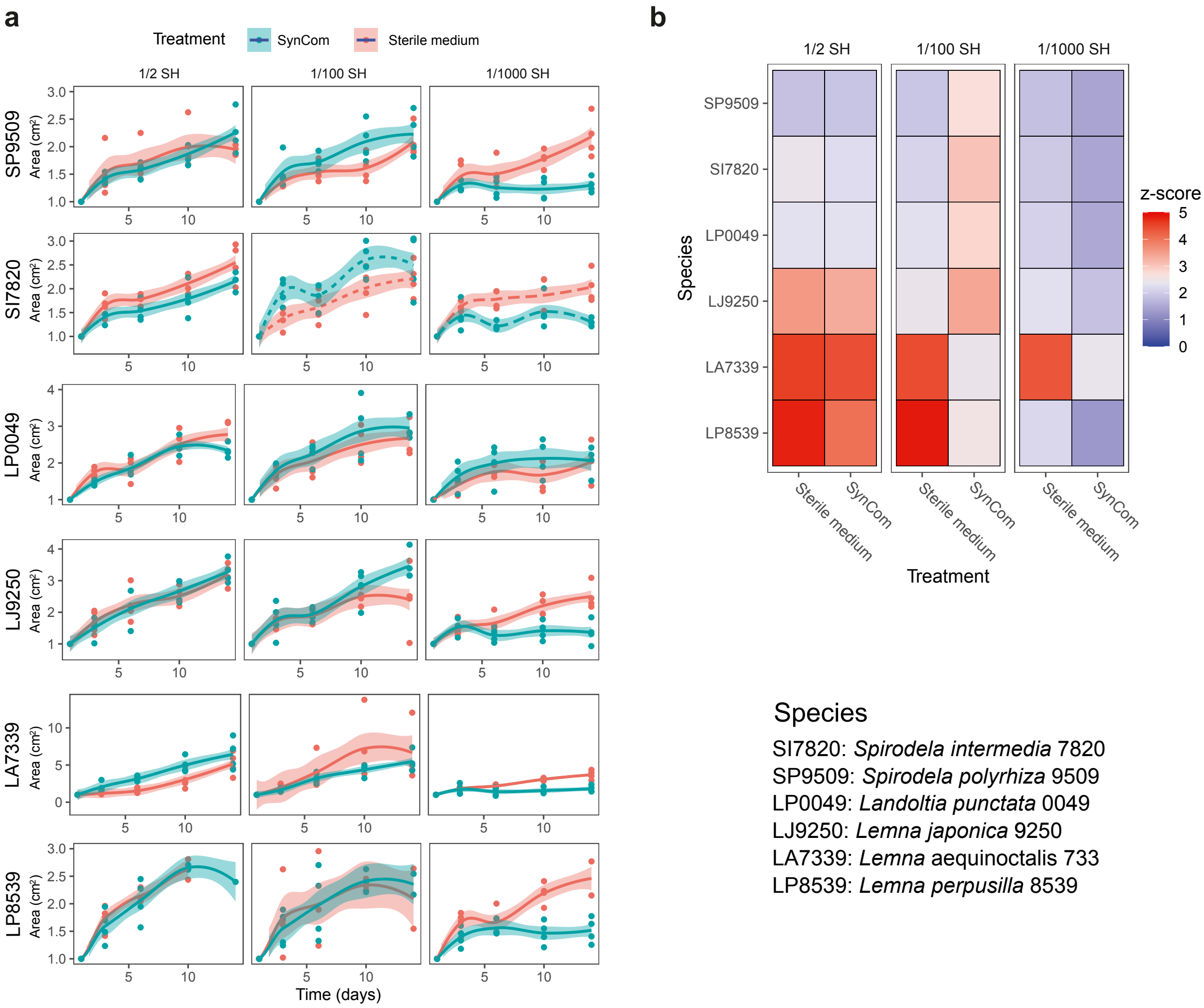

Supplementary Figure 13. The bacterial synthetic community induces saturation-type growth kinetics in plants grown under low-nutrient conditions. a. Reproduction kinetics of the six plant species used, grown either in sterile SH medium (red) or inoculated with the bacterial synthetic community (blue). From left to right, each column represents the reproduction kinetics of plant species grown in SH medium with high (1/2 SH), medium (1/100 SH), and low (1/1000 SH) nutrient concentrations. Plant reproduction was quantified by measuring the total frond area in each sample. The shaded area represents the standard deviation (SD). We used n = 4 independent biological replicates. Note that only low nutrient concentration recreates the competitive nutrient conditions observed in natural waters. **b.** Heatmap showing the normalised (*z*-score) rate of plant growth change (slope, cm^2/days) calculated during the linear phase of each plant growth curve shown in panel a, across the different bacterial and nutrient conditions.

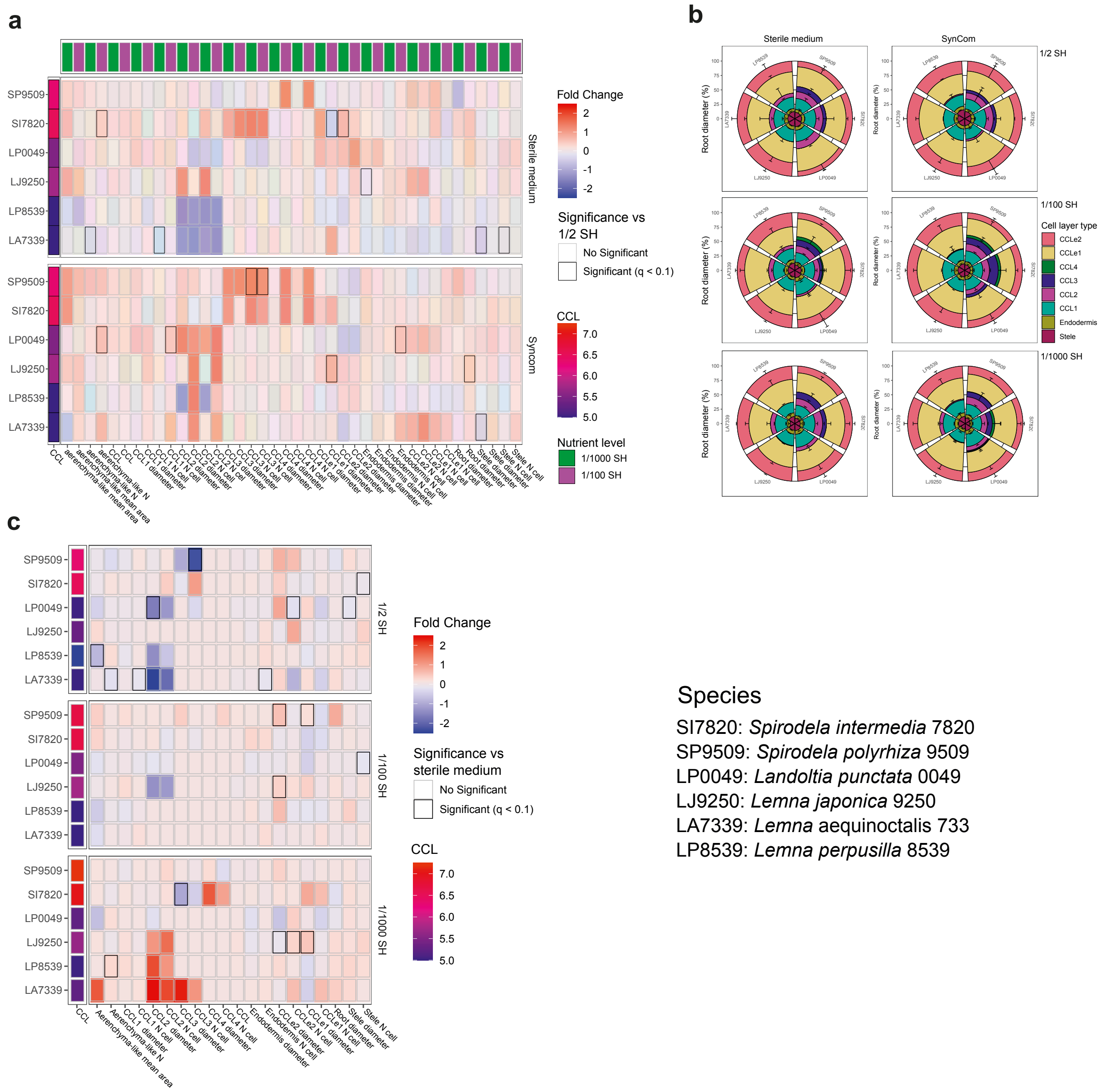

Supplementary Figure 14. A bacterial synthetic community alters root anatomy under low-nutrient conditions. a. Heatmap of normalized anatomical traits determined in roots of the six *Lemnaceae* species grown in sterile SH medium (top panel) and SH medium supplemented with the bacterial synthetic community (bottom panel) across the nutrient conditions. The number of root cell layers present in each species under sterile conditions is shown in the bar on the left. Statistically significant enriched (red) and depleted (blue) root anatomical traits with respect to plants grown in 1/2 SH medium are outlined in black ($q < 0.1$; n = 4 independent biological replicates). **b.** Rose diagram displaying the average diameter of each root cell layer (colours), quantified from confocal images of root sections from the six *Lemnaceae* species grown in sterile SH medium (sterile medium) or in SH medium inoculated with the bacterial synthetic community (SynCom), across the three nutrient conditions used (1/2 SH, 1/100 SH, and 1/1000 SH; rows). We used n = 4 independent biological replicates. **c**. Heatmap analysis showing the standardised fold changes in root anatomical features of six *Lemnaceae* species grown in SH medium supplemented with the bacterial synthetic community compared with the same plants grown in sterile SH medium, across different nutrient concentrations (1/2 SH, 1/100 SH, and 1/1000 SH). Values of anatomical features that differ significantly in response to the bacterial synthetic community relative to the sterile SH medium control (*q*-value < 0.1) are outlined in black.

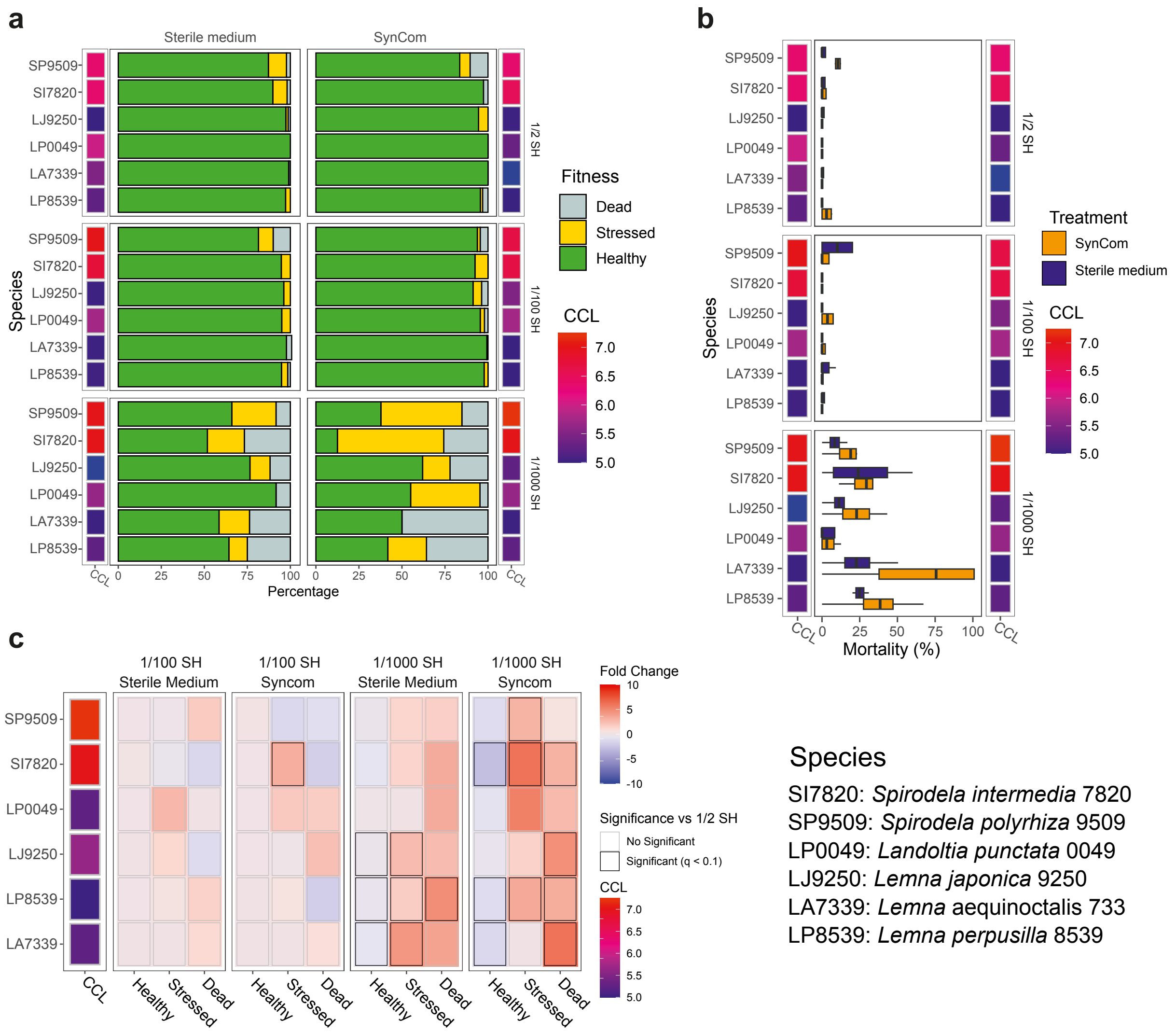

Supplementary Figure 15. The root microbiota controls root anatomy to increase plant survival under nutrient-limited conditions. a. Stacked bar plot showing the percentage of dead (grey), stressed (yellow), and healthy (green) plants for each of the six plant species grown either in sterile SH medium or in SH medium inoculated with the bacterial synthetic community, across the three nutrient conditions (1/2 SH, 1/100 SH, 1/1000 SH). We used n = 4 independent biological replicates. **b**. Box plots showing the percentage of dead plants quantified for each plant species grown in panel a. The heatmaps on the left and right of panels a and b represent the number of root cell layers counted in each plant grown under the conditions described in panel a. In the box plot, the centre line indicates the median, the box limits represent the first and third quartiles, the whiskers extend to the most extreme values within 1.5 × the interquartile range (IQR) **c.** Heatmap analysis showing the standardised fold changes in the percentage of dead, stressed, and healthy plants for each of the six plant species used, grown in 1/100 SH and 1/1000 SH medium inoculated or not with the bacterial synthetic community and compared with the same plant species grown in sterile 1/2 SH medium under the same treatments. Values significantly different from the sterile 1/2 SH medium control (*q*-value < 0.1) are outlined in black. We used n = 4 independent biological replicates.

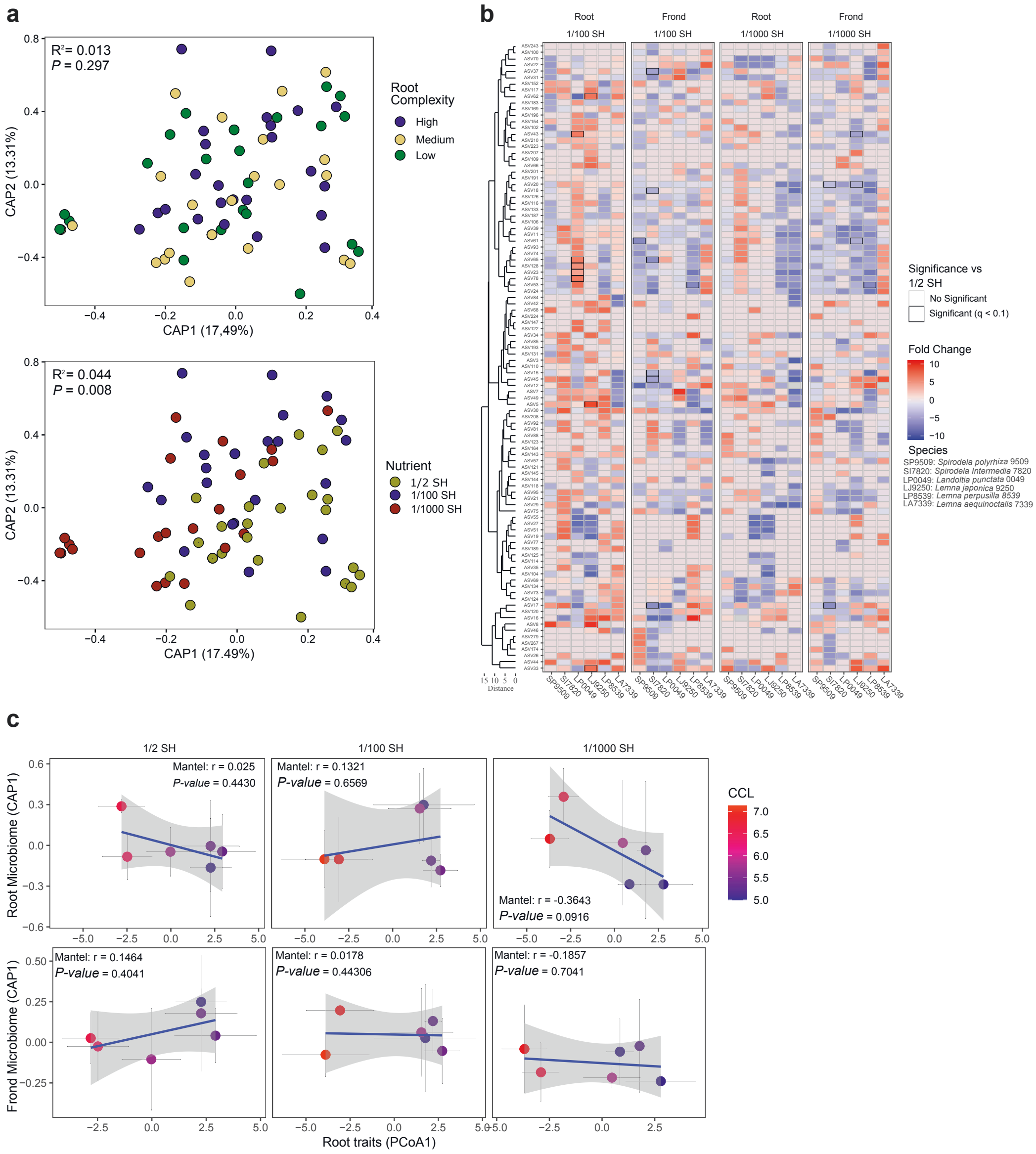

Supplementary Figure 16. The root microbiota influences root anatomy under nutrient-limited conditions. a. Canonical analysis of principal coordinates (CAP) showing the influence of (top) different root anatomical complexity (high, medium, and low) and (bottom) nutrient content in the growth medium (1/2 SH, 1/100 SH, and 1/1000 SH) over the bacterial communities colonising the plant fronds. The R^2 and *p*-values shown within each panel were obtained using a PERMANOVA, n = 4. **b.** Heatmap showing normalized fold changes in the relative abundance of the most abundant ASVs colonising roots and fronds of the six *Lemnaceae* species grown in 1/100 SH and 1/1000 SH medium inoculated with the bacterial synthetic community, compared with the same plant species grown in 1/2 SH medium inoculated with the bacterial synthetic community. ASVs that are significantly enriched (red) and depleted (blue) relative to the sterile 1/2 SH medium control (*q*-value < 0.1) are outlined in black. ASVs relative abundance fold-change values were clustered. The dendrogram represents the hierarchical clustering of ASVs, and the scale indicates the distance between the identified clusters. We used n = 3 in the case of the root samples, and n = 4 in the case of the frond samples. **c.** Pairwise correlation analysis between (top) a dissimilarity matrix based on the root microbiota composition (CAP1) and a dissimilarity matrix based on root anatomical traits (PCoA1) and (bottom) a dissimilarity matrix based on the frond microbiota composition (CAP1) and a dissimilarity matrix based on root anatomical

traits (PCoA1). Analyses were performed on six plant species with different root complexities grown in SH medium with different nutrient concentrations (1/2 SH, 1/100 SH, 1/1000 SH). The error bars represent the standard deviation (SD) Mantel's *r* statistic and the corresponding *p*-values obtained from 10,000 permutations are shown. We used n = 3 in the case of the root samples, and n = 4 in the case of the frond samples.

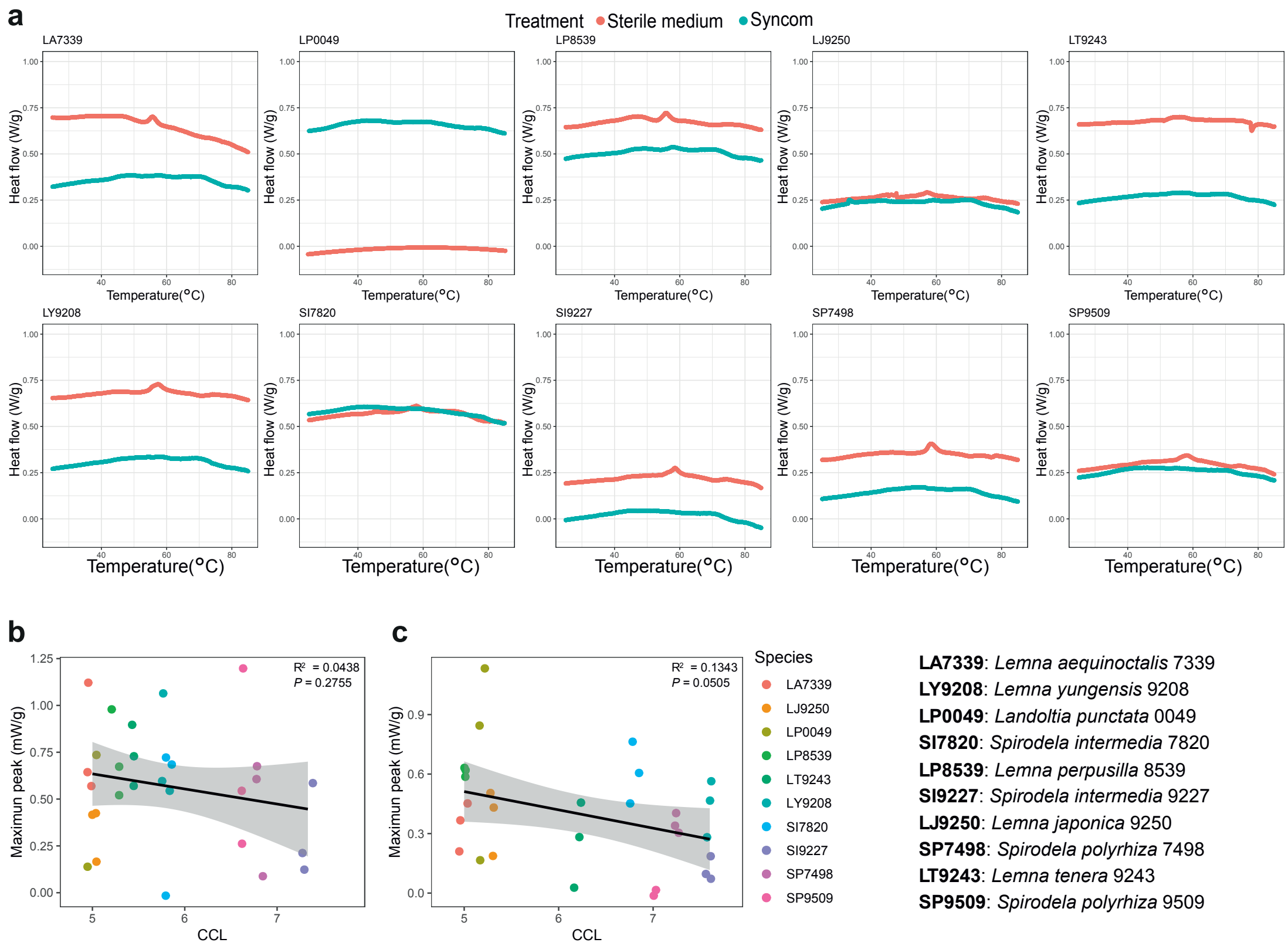

Supplementary Figure 17. Bacterial colonisation alters the anatomical and metabolic properties of root microhabitats. a. Thermogram showing the phase transition of root components from axenic plants (red) and from plants colonized by a bacterial synthetic community (blue) across different species of *Lemnaceae* with distinct levels of root complexity. Each value represents the result of analysing n = 3 independent biological replicates in each case, consisting of at least 10 roots. In most cases, microbial colonization altered the thermodynamic properties of the roots **b.** Linear regression analyses were performed between the maximum values of the phase-transition peaks (extracted from panel a) and the number of root cell layers in different *Lemnaceae* species grown under axenic conditions, and **c.** between the maximum values of the phase-transition peaks (extracted from panel a) and the number of root cell layers in different *Lemnaceae* species colonized by the bacterial synthetic community. The proportion of variation in peak intensity explained by the number of root cell layers (R^2), along with the associated *p*-values, are shown in each panel. Notably, the linear regression is statistically significant only for roots colonised by microbes ($p < 0.1$). We used n = 3 independent biological replicates per plant species in each case.

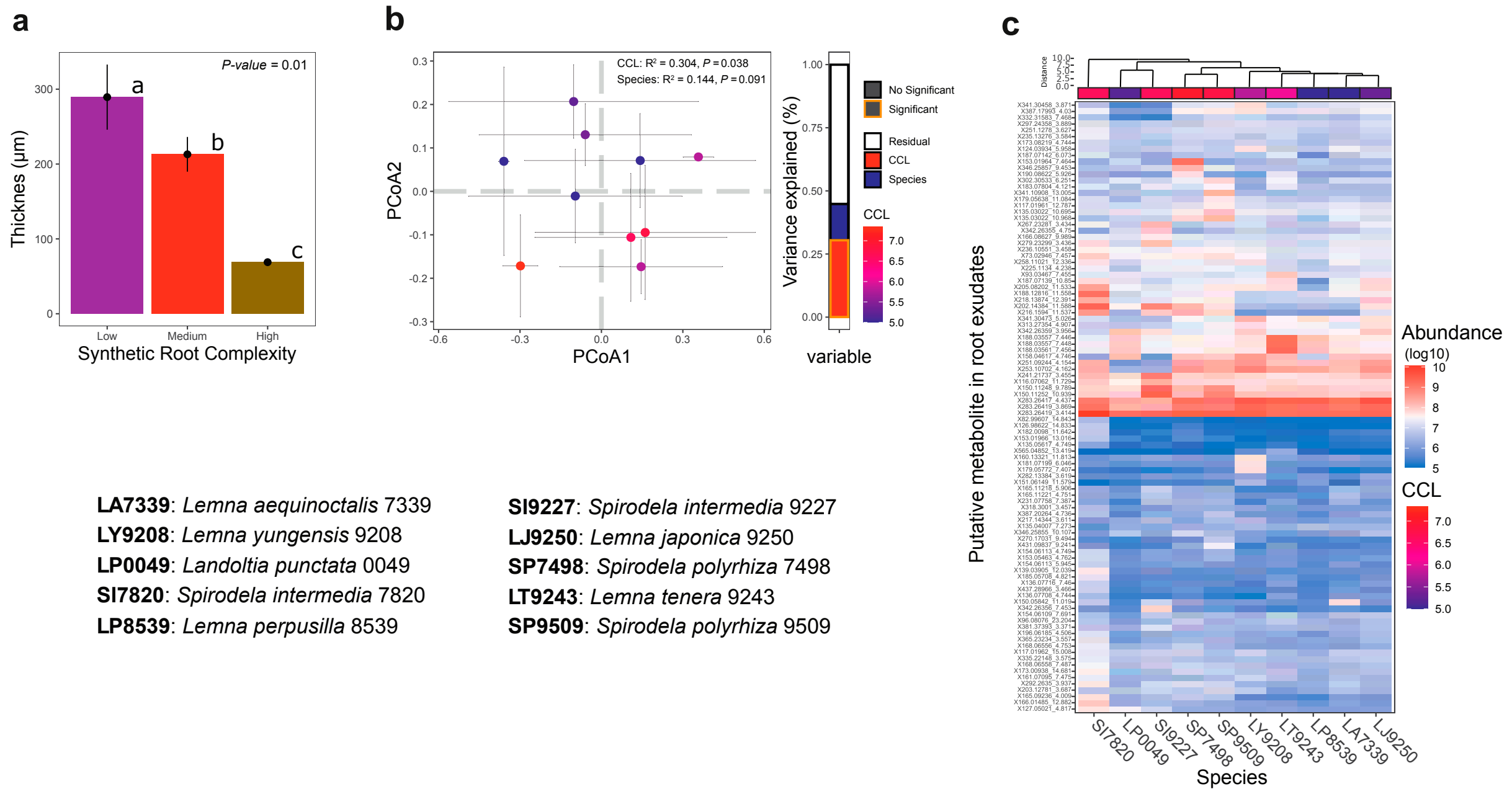

Supplementary Figure 18. Synthetic roots allow the disentangling of anatomical and metabolic complexity. **a**. Graph showing the thickness of the different “synthetic roots” as determined by scanning electron microscopy. Differences in thickness among the “synthetic roots” were assessed using ANOVA (p < 0.05; n = 3 independent biological replicates in each case). **b.** Principal coordinates analysis (PCoA) of metabolite composition showing the metabolic profiles of exudates from different plant species grown under axenic conditions. Each point represents the mean distribution of values for a species along both axes and is coloured according to the number of root cell layers (CCL) present in each plant. The error bars represent the standard deviation (SD). The bar on the right indicates the percentage of variance explained by the experimental variables, as determined by a Permutational Multivariate Analysis of Variance (PERMANOVA). Significant variables are highlighted in orange (p-value < 0.05). The R^2 values and their corresponding p-values from the PERMANOVA model, shown within the panel, correspond to the CCL and Species variables, respectively. We used n = 3 independent biological replicates in each case. **c.** Heatmap showing the normalized abundance of the most abundant putative metabolites detected in root exudates of ten *Lemnaceae* species grown under axenic conditions in ½ SH medium. Data are clustered according to the number of root cell layers (CCLs) of the different plant species (top). The dendrogram represents the hierarchical clustering of traits, and the scale indicates the distance between the identified clusters. We used n = 3 independent biological replicates in each case.

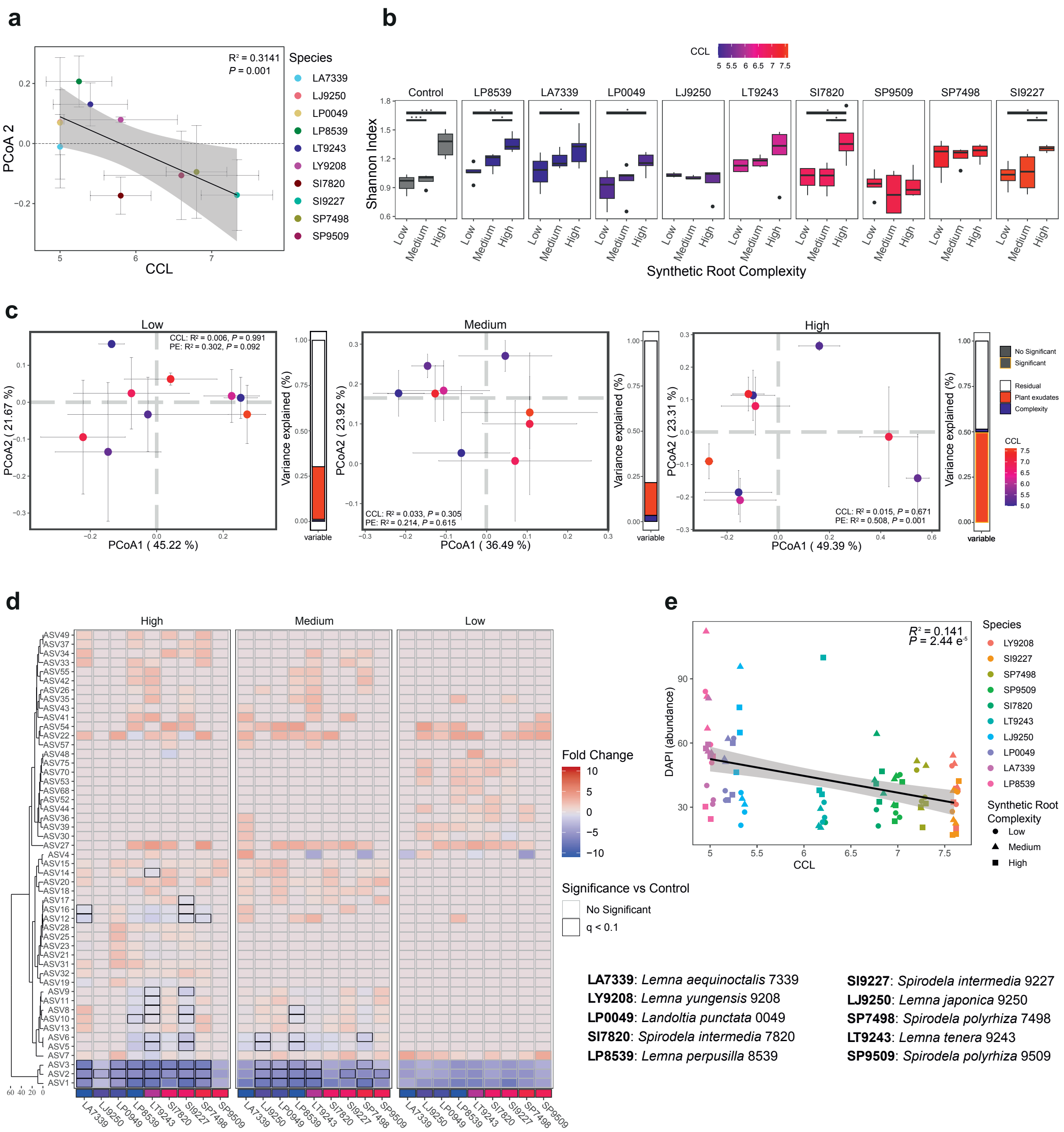

Supplementary Figure 19. The anatomical and metabolic properties of microhabitats determine microbial colonisation. a. Linear regression analysis between the metabolic composition of exudates, based on the PCoA2 metabolic component, and the number of root cell layers across ten *Lemnaceae* species. The panel shows the proportion of variation (R^2) in metabolic complexity explained by the number of cortical cell layers (CCLs), along with the associated *p*-values. The error bars represent the standard deviation (SD). We used n = 3 independent biological replicates in each case. **b**. The boxplots show alpha diversity, estimated using the Shannon diversity index, in “synthetic roots” with different levels of morphological complexity (low, medium, and high) supplemented with root exudates collected from the plant species used and inoculated with the bacterial synthetic community. As a control, “synthetic roots” supplemented with 1/2 SH medium, along with the bacterial synthetic community, were included. Boxplots are coloured according to the number of root cell layers (CCLs) present in the roots of the species from which the exudates were collected. In the box plots, the centre line indicates the median, the box limits represent the first and third quartiles, the whiskers extend to the most extreme values within 1.5 × the interquartile range (IQR), and points beyond the whiskers indicate outliers. In all cases,

alpha diversity in “synthetic roots” was compared across “synthetic root” complexity using a *t*-test ($p < 0.05$). **c**. Principal coordinates analysis (PCoA) showing the projection of bacterial compositional profiles in “synthetic roots” of varying complexity (low, medium, and high), supplemented with *Lemnaceae* root exudates and inoculated with the bacterial synthetic community. Each point in the scatter plot represents the mean distribution of values on both axes and is coloured according to the number of root cell layers (CCL) in the plant root from which the exudates were obtained. Each point represents the mean of n = 6 independent biological replicates. Error bars represent the standard deviation (SD). The bar on the right displays the percentage of variance explained by the experimental variables, as determined by Permutational Multivariate Analysis of Variance (PERMANOVA). Significant variables ($p < 0.05$) are highlighted in orange. R^2 values and their corresponding *p*-values from the PERMANOVA, shown within the panel, correspond to the CCL and plant exudate (PE) variables, respectively. We used n = 6 independent biological replicates in each case. Notably, only the high complexity “synthetic root” assembles a distinct microbial composition across the different exudates tested. **d**. Heatmaps showing the enrichment patterns (relative to the control) of the most abundant ASVs colonizing “synthetic roots” of distinct morphological complexities and exposed to various root exudates. Tile colours represent the $\log_2$ fold change estimated from a generalized linear model comparing the abundance of each ASV (Y-axis) in “synthetic roots” exposed to the various exudates (X-axis) with their abundance in roots exposed to 1/2 SH medium alone. The X-axis is organised according to the number of root cell layers (CCL) in the plant species used for exudate extraction, and the Y-axis is clustered based on ASVs abundance. The dendrogram represents hierarchical clustering of ASVs, and the scale indicates the distance between identified clusters. Tiles outlined in black indicate ASVs that are significantly enriched (red) or depleted (blue) relative to the control ($q < 0.1$; n = 6 independent biological replicates in each case. **e**. Linear regression analysis between the microbial abundance, determined by DAPI (4',6-diamidino-2-phenylindole) staining, in “synthetic roots” of varying morphological complexities (shapes) exposed to different plant exudates, and the number of root cell layers (CCL) in the *Lemnaceae* species (colours) used to produce those exudates. The proportion of variation in microbial abundance explained by CCL (R^2), along with the associated *p*-values, are shown in the panel (n = 6 independent biological replicates).

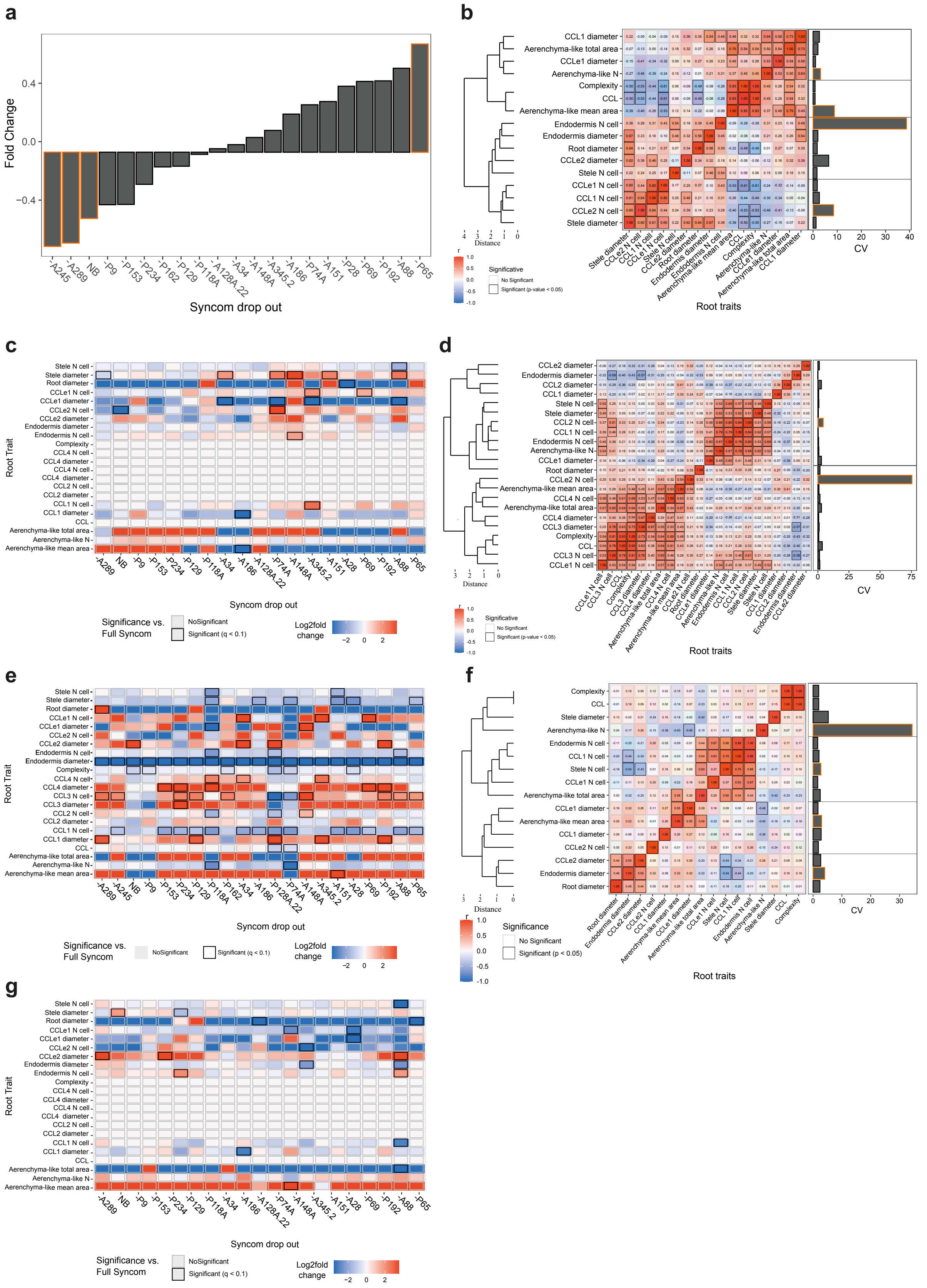

Supplementary Figure 20. Root complexity and root microbiota members influence root anatomical plasticity under low nutrient availability. a. Bar graph showing the $\log_2$ fold change in the number of root cell layers in *Spirodela polyrhiza* 9509 (high root anatomical complexity) exposed or not (NB) to 20 bacterial synthetic communities. Each community lacks one of the 20 most abundant ASVs colonizing the roots, and results are shown relative to the same plant exposed to the full bacterial synthetic community. Bars

highlighted in orange represent roots with a significantly different number of root cell layers between plants inoculated or not (NB) with the dropout synthetic communities and those inoculated with the full synthetic community ($q < 0.05$; n = 4 independent biological replicates). **b.** Heatmap showing the Pearson correlation coefficient from pairwise comparison among root anatomical features quantified in *Lemna aequinoctalis* 7339 (low root anatomical complexity) grown with the different synthetic communities tested (full and dropout communities). Values are clustered according to the features. The dendrogram represents the hierarchical clustering of traits, and the scale indicates the distance between the identified clusters. Correlation coefficient values are displayed within each cell, and significant correlations are highlighted in black ($p < 0.05$). The bar graph on the right shows the coefficient of variation (CV) for each root trait in *Lemna aequinoctalis* 7339 across all bacterial synthetic communities tested (full and dropouts). CV values highlighted in orange represent the highest within each cluster (n = 4 independent biological replicates). **c**. Heatmap showing the $\log_2$ fold change of root trait values in *Lemna aequinoctalis* 7339 (low root anatomical complexity) exposed to bacterial synthetic communities in which each of the 20 most abundant ASVs colonising the roots was individually dropped out, compared with root trait values measured in plants exposed to the full bacterial synthetic community. As a control, plants grown under axenic conditions (NB) were included. Statistically significant enriched (red) and depleted (blue) root trait values, relative to those in plants exposed to the full bacterial synthetic community, are outlined in black ($q < 0.1$; n = 4 independent biological replicates). **d**. Heatmap showing the Pearson correlation coefficients for pairwise comparisons among root anatomical features quantified in *Spirodela polyrhiza* 9509 (high root anatomical complexity) grown with the full synthetic community and in mono-association experiments with each of the 20 most abundant ASVs. Values are clustered according to the features. The dendrogram represents the hierarchical clustering of traits, and the scale indicates the distance between the identified clusters. Correlation coefficient values are shown within each cell, and significant correlations are highlighted in black ($p < 0.05$). The bar graph on the right shows the coefficient of variation (CV) for each root trait in *Spirodela polyrhiza* 9509 across all bacterial treatments applied. CV values highlighted in orange represent the highest within each cluster (n = 4 independent biological replicates). **e.** Heatmap showing the log2 fold change of root trait values in *Spirodela polyrhiza* 9509 (high root anatomical complexity) grown in mono-association experiments with each of the 20 most abundant ASVs, compared with plants inoculated with the full bacterial synthetic community. Plants grown under axenic conditions (NB) were used as a control. Statistically significant enriched (red) and depleted (blue) root trait values, relative to those observed in plants exposed to the full bacterial synthetic community, are outlined in black ($q < 0.1$; n = 4 independent biological replicates). **f.** Heatmap showing the Pearson correlation coefficients for pairwise comparisons among root anatomical features quantified in *Lemna aequinoctalis* 7339 (low root anatomical complexity) grown with the full synthetic community and in mono-association experiments with each of the 20 most abundant ASVs. Values are clustered according to the features. The dendrogram represents the hierarchical clustering of traits, and the scale indicates the distance between the identified clusters. Correlation coefficient values are shown within each cell, and significant correlations are highlighted in black ($p < 0.05$). The bar graph on the right shows the coefficient of variation (CV) for each root trait in *Lemna aequinoctalis* 7339 across all bacterial treatments applied. CV values highlighted in orange represent the highest within each cluster (n = 4 independent biological replicates). **g**. Heatmap showing the log2 fold change of root trait values in *Lemna aequinoctalis* 7339 (low root anatomical complexity) grown in mono-association experiments with each of the 20 most abundant ASVs, compared with plants inoculated with the full bacterial synthetic community. Plants grown under axenic conditions (NB) were used as a control. Statistically significant enriched (red) and depleted (blue) root trait values, relative to those observed in plants exposed to the full bacterial synthetic community, are outlined in black ($q < 0.1$; (n = 4 independent biological replicates).

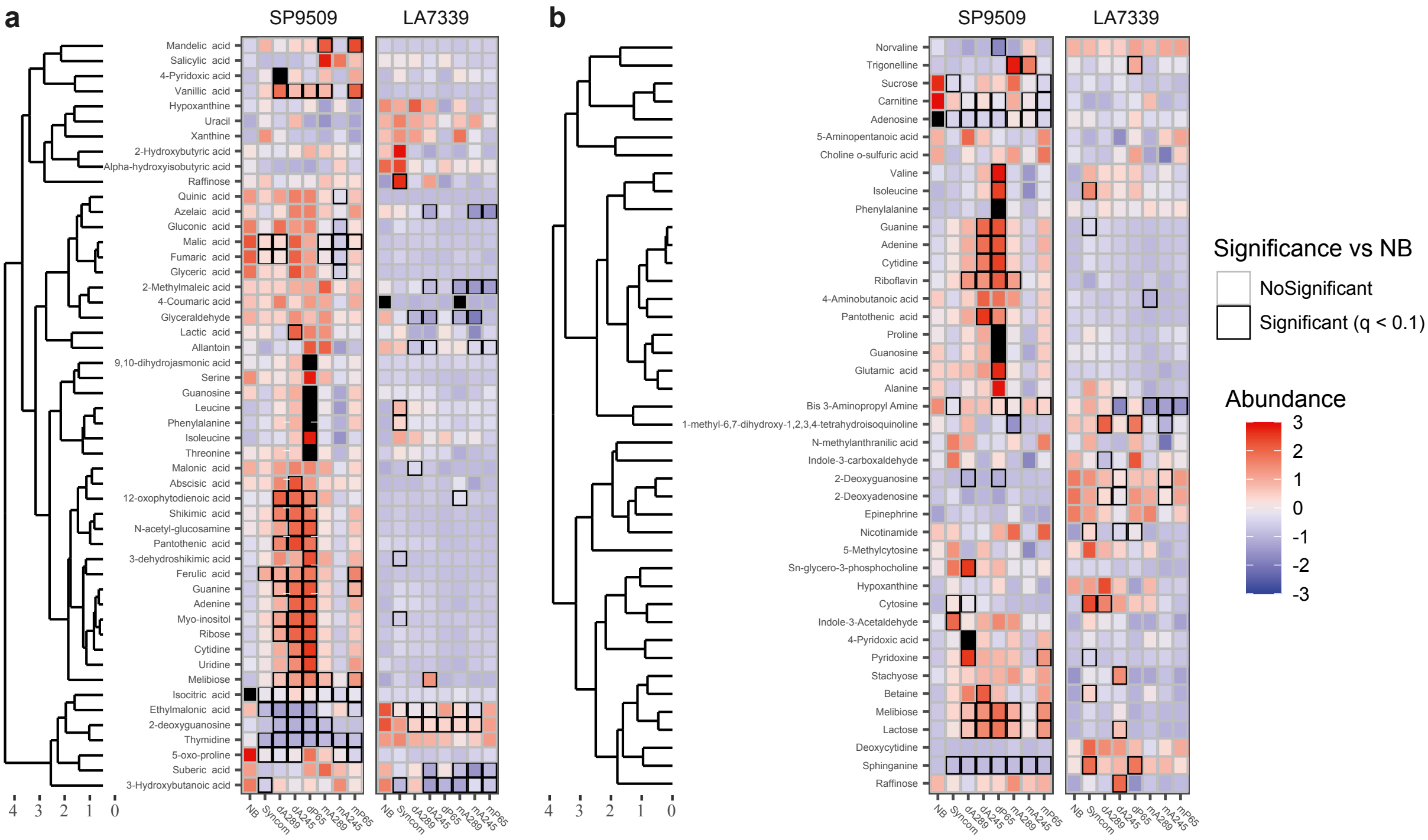

Supplementary Figure 21. Root metabolic profiles change upon colonisation with the root microbiota. a. Heatmap showing the normalised (*z*-score) metabolite abundance detected using negative and **b.** positive ion modes in mass spectrometry. Metabolites were measured in the roots of two *Lemnaceae* species, *Lemna aequinoctalis* 7339 (low inherent anatomical root complexity) and *Spirodela polyrhiza* 9509 (high inherent anatomical complexity). Plants were grown with the full bacterial synthetic community (Syncom), with three drop-out synthetic communities in which ASVs A289, A245, and P65 were individually removed, or in mono-association experiments with these ASVs (mA289, mA245, and mP65) compared to plants grown under axenic conditions (NB). Values are clustered according to metabolite abundance. The dendrogram represents hierarchical clustering of metabolite profiles, and the scale bar indicates the distance between identified clusters. Statistically significant metabolites relative to those in axenic plants are outlined in black ($q < 0.1$; n = 5 independent biological replicates). The black squares in the heatmaps represent samples in which the analysed metabolite was not detected.

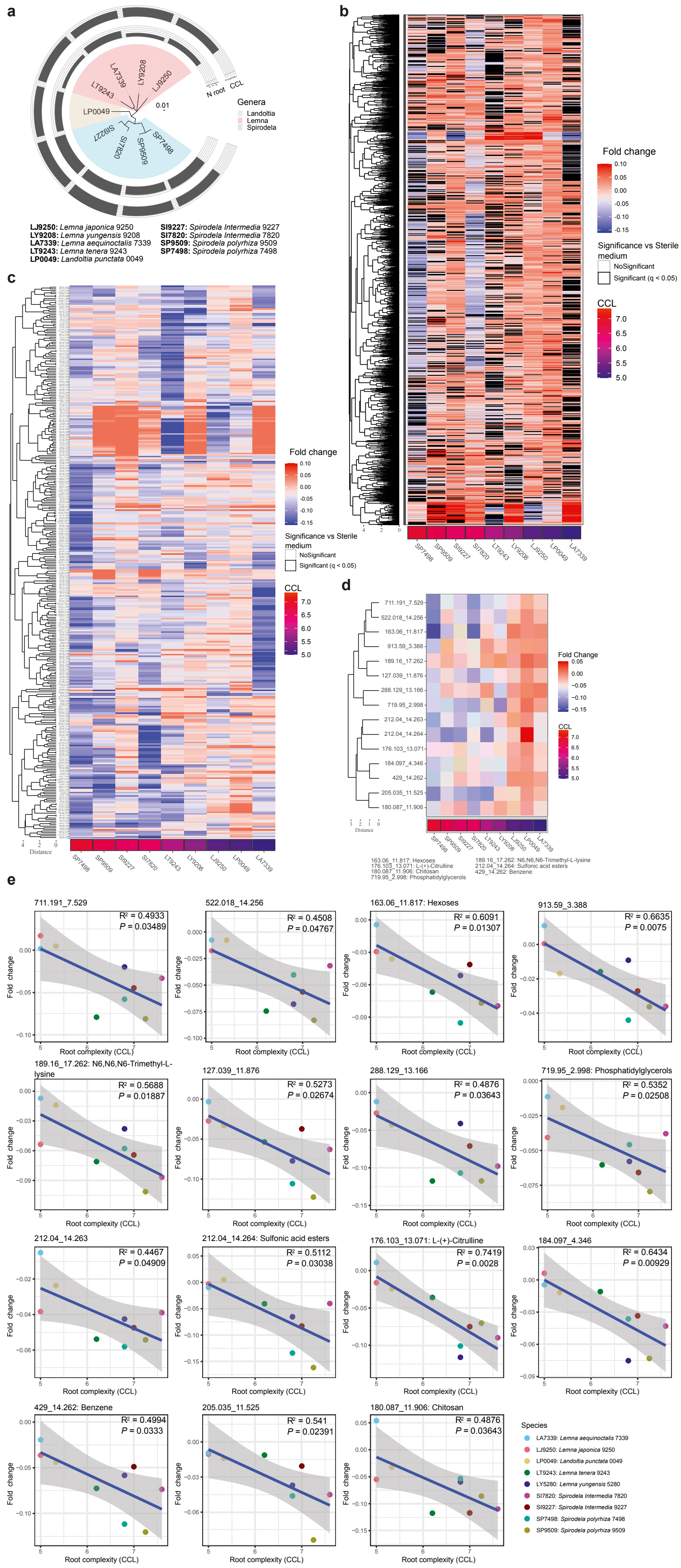

Supplementary Figure 22. The metabolic composition of roots changes upon bacterial colonisation, and the concentrations of some putative metabolites correlate with root anatomical complexity. a. Phylogenetic tree showing the distribution of nine *Lemnaceae* species with different root anatomical complexity used in these experiments. The tree was built using variations in the chloroplast *RPL16* gene sequences, which encode the L16 ribosomal protein, a component of the large (50S) ribosomal subunit in chloroplasts of all

plants. The inner and the outer rings represent, respectively, the total number of roots and the number of root cell layers (CCL) observed in plants grown in sterile natural water. Background colours indicate plant genera. **b.** Heatmap showing the normalized abundance of putative metabolites present in the roots of nine *Lemnaceae* species inoculated with the full bacterial synthetic community, compared to plants grown under axenic conditions. Values are clustered based on putative metabolite abundance. The dendrogram represents hierarchical clustering of putative metabolite profiles, and the scale bar indicates the distance between identified clusters. The data are ordered according to plant root complexity, measured as the number of root cell layers, and this is shown in the bottom bar. Statistically significant enriched (red) and depleted (blue) putative metabolites relative to axenic plants are outlined in black (q < 0.05; n = 6 independent biological replicates). **c.** Heatmap showing the normalized abundance of 341 putative metabolites, represented as m/z-RT (retention time, min), with a significantly different accumulation in plants inoculated with the bacterial synthetic community compared to axenic plants. This analysis serves as the first filter to identify putative metabolites involved in regulating the anatomical plasticity of roots in response to the microbiota. The dendrogram represents hierarchical clustering of putative metabolite profiles, and the scale bar indicates the distance between identified clusters. Plant species are organised according to their root complexity, as shown in the bottom bar. Statistically significant enriched (red) and depleted (blue) putative metabolites relative to those found in axenic plants are outlined in black (q < 0.05; n = 6 independent biological replicates). **d.** Heatmap showing the normalized abundance of 15 putative metabolites, represented as m/z-RT (retention time, min), in the roots of nine *Lemnaceae* species. These 15 putative metabolites were selected because their changes in abundance significantly correlate with the anatomical complexity of the roots present in these plants (panel e). This analysis represents the second filter used to identify putative metabolites that regulate root anatomical plasticity in response to the microbiota. The dendrogram illustrates the hierarchical clustering of putative metabolite profiles, with the scale bar indicating the distance between identified clusters. Plant species are organised according to their root complexity, as shown in the bottom bar (n = 6 independent biological replicates). **e**. Linear regression analyses showing significant correlations between the abundances ($\log_2$fold-change) of 15 putative metabolites identified in this study and the number of root cell layers (CCL) in the *Lemnaceae* species examined (indicated by colours). The proportion of variance in metabolite abundance explained by the number of root cell layers (R^2), along with the associated p-values, is shown in the panel (n = 6 independent biological replicates).

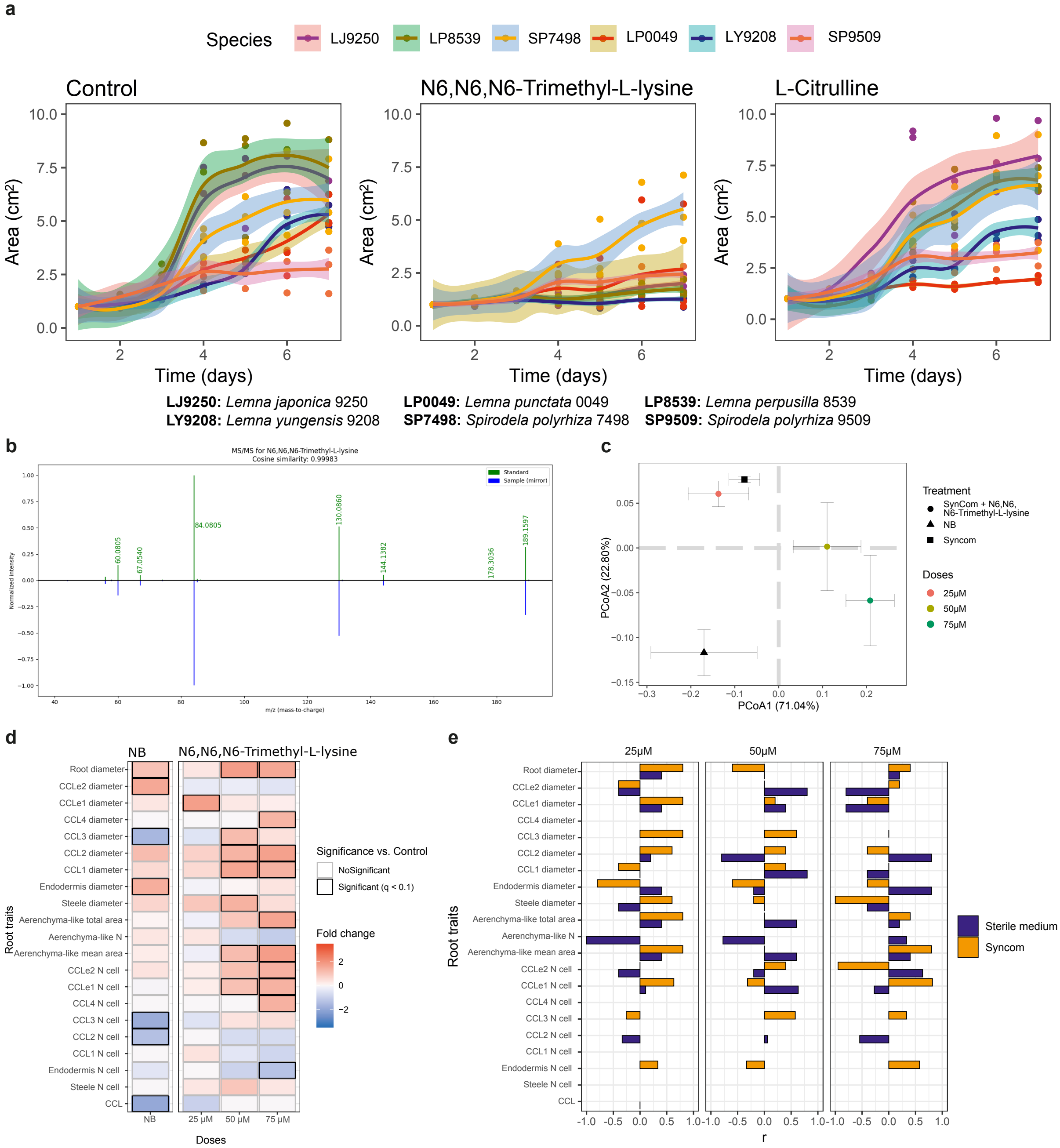

Supplementary Figure 23. A modulatory compound regulates root plasticity in the presence of the microbiota. a. Kinetic of reproduction of six *Lemnaceae* plant species (LJ9250: *Lemna japonica* 9250; LP0049: *Landoltia punctata* 0049; LP8539: *Lemna perpusilla* 8539; LY9208: *Lemna yungensis* 9208; SP7498: *Spirodela polyrhiza* 7498; SP9509: *Spirodela polyrhiza* 9509) used in this study (different colours), grown with the full bacterial synthetic community in 1/1000 SH medium (control) or supplemented with the purified metabolites N^6,N^6,N^6-Trimethyl-L-lysine or L-Citrulline. Total frond area (cm^2) was quantified as a proxy for plant reproduction. We used n = 6 independent biological replicates. **b.** Representative MS^2 comparison between the N^6,N^6,N^6-Trimethyl-L-lysine standard (top) and a root sample (bottom), showing coincident fragmentation of the m/z 189.1596 peak at a retention time of 14.78 min. **c.** Principal coordinates analysis (PCoA) showing the projection of root anatomical traits (see panel d) measured in *Spirodela polyrhiza* 9509 (high root anatomical complexity) grown with the full bacterial synthetic community, either alone (square) or supplemented with different concentrations of N^6,N^6,N^6-Trimethyl-L-lysine (25, 50, 75 μM) (circle). As a control, plants were grown under axenic conditions (NB) (triangle). The error bars represent the standard deviation (SD). We used n = 6 independent biological replicates. **d.** Heatmap showing the fold change in root

anatomical traits of *Spirodela polyrhiza* 9509 grown with the full bacterial synthetic community supplemented with different concentrations of N^6,N^6,N^6-Trimethyl-L-lysine (25, 50, 75 µM) or under axenic conditions (NB), relative to plants inoculated with the bacterial synthetic community alone. Statistically significant enriched (red) and depleted (blue) root trait values relative to plants exposed to the bacterial synthetic community are outlined in black ($q < 0.1$). Note that differences in anatomical traits between plants inoculated with the synthetic community and those treated with a low concentration of N^6,N^6,N^6-Trimethyl-L-lysine are minimal. At high concentrations, the root anatomical traits differ significantly from those of plants inoculated with the bacterial synthetic community and resemble more closely those of the no-bacteria control (NB). We used n = 6 independent biological replicates. **e.** The panel shows the correlation coefficient (r) from pairwise comparisons between individual root anatomical traits quantified in axenic roots (blue) or inoculated with the full bacterial synthetic community alone (orange) and those measured in plants exposed to different concentrations of N^6,N^6,N^6-Trimethyl-L-lysine. We used n = 6 independent biological replicates).

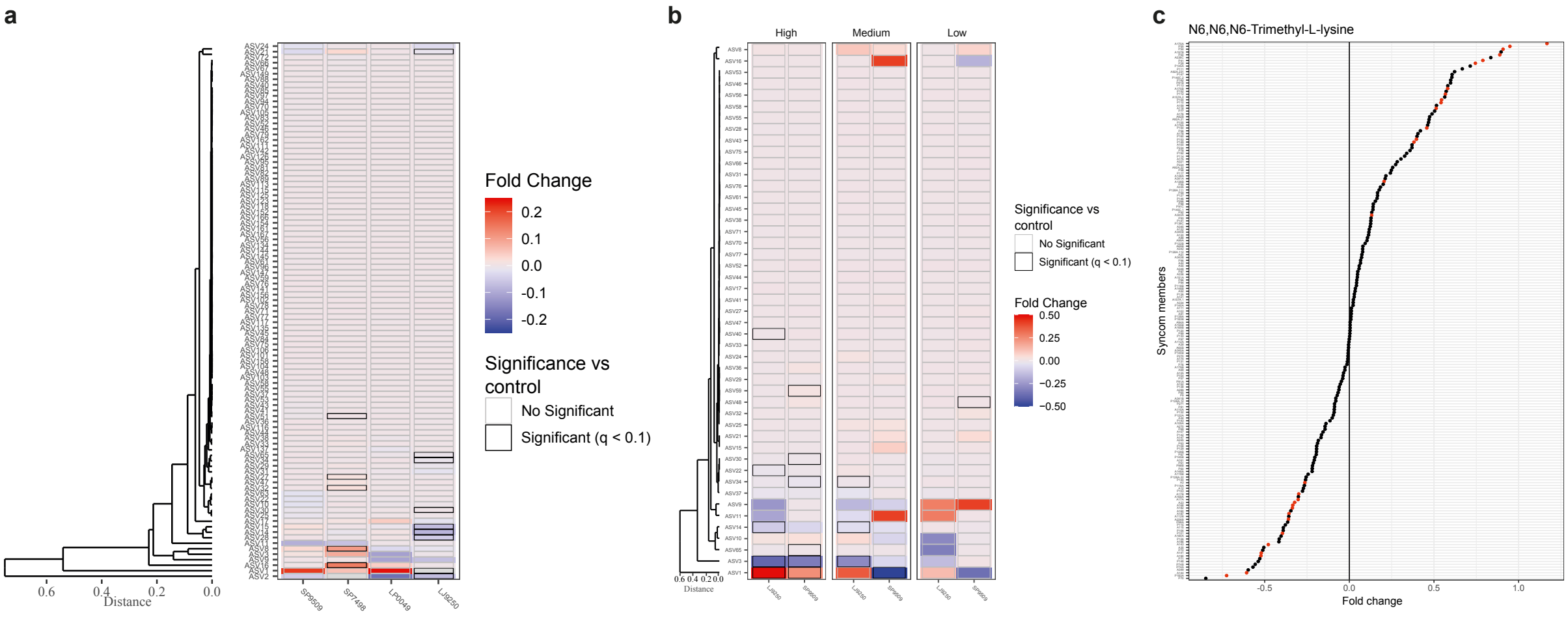

Supplementary Figure 24. The modulatory metabolite influences the structure of the root microbiota community. a. Heatmap showing the fold change in relative abundance of the most abundant ASVs in the roots of four *Lemnaceae* species with medium to high inherent root complexities (SP9509: *Spirodela polyrhiza* 9509; SP7498: *Spirodela polyrhiza* 7498; LP0049: *Landoltia punctate* 0049; LJ9250: *Lemna japonica* 9250), grown in 1/1000 SH medium inoculated with the full bacterial synthetic community, and in plants additionally supplemented with the modulatory metabolite N^6,N^6,N^6-Trimethyl-L-lysine. Data were grouped by ASVs. The dendrogram illustrates the hierarchical clustering of ASVs, with the scale bar indicating the distance between identified clusters. Statistically significant enriched (red) and depleted (blue) root trait values relative to plants exposed to the bacterial synthetic community are outlined in black ($q < 0.1$; n = 6 independent biological replicates). **b.** Heatmap showing the fold change in relative abundance of the most abundant ASVs in "synthetic roots" supplemented with root exudates from two plants with contrasting root complexity (LJ9250 (*Lemna japonica* 9250; low inherent root complexity) and SP9509 (*Spirodela polyrhiza* 9509; high inherent root complexity)) inoculated with the bacterial synthetic community, and in samples additionally supplemented with the modulatory metabolite N^6,N^6,N^6-Trimethyl-L-lysine. Data were grouped by ASVs. The dendrogram illustrates hierarchical clustering of ASVs, with the scale bar indicating the distance between identified clusters. We used n = 6 independent biological replicates. **c.** The curves show the fold change between the slopes of the linear phase of the growth curves for each bacterial strain grown in 1/1000 SH medium and in 1/1000 SH medium supplemented with N^6,N^6,N^6-Trimethyl-L-lysine for 24 h. Statistically significant fold changes are highlighted in red (*t*-test, p-value < 0.05; n = 3).
