# Supplementary material for: The metabolic and anatomical complexity of root microhabitats modulate their interaction with the microbiota
Source: Nat Commun. 2026 Aug 13;17:9738. doi: 10.1038/s41467-026-76440-4 (PMC13562559; doi:10.1038/s41467-026-76440-4)
Supplement: Supplementary file 4 [file 41467_2026_76440_MOESM2_ESM.pdf]

Description of Additional Supplementary Files

Supplementary Data 1. Composition of the growth media used for the isolation of bacterial strains.

Supplementary Data 2. Bacterial strain collection built in this work. The columns despite from left to right the bacterial strain ID, the number of the *Lemnaceae* accession, and the name of the *Lemnaceae* specie used for the bacterial isolation, the origin of the water used to grow the plants, the organ (compartment) of the plant from which we isolate the bacterial strains, the medium we used for the strain isolation, the length of the *16S rRNA* gene sequences we generated, the number of Ns in the sequence, the taxonomical attributes of the individual strains (From column I to N), the *16S rRNA* gene sequences in each case, and if the strain was used in the full synthetic community (yes) or not (no), in the different drop-out synthetic communities (yes (drop-out x), and mono-association experiments (yes).

Supplementary Data 3. Metabolic features of root exudates. Chemical metabolic profiles from root exudates analyses of ten Lemnaceae species (*Spirodela intermedia* SI7820, *Spirodela polyrhiza* SP9509, *Spirodela polyrhiza* SP7498, *Landoltia punctata* LP0049, *Spirodela intermedia* SI9227, *Lemna aequinoctalis* LA7339, *Lemna japonica* LJ9250, *Lemna tenera* LT9243, *Lemna yungensis* LY9208, *Lemna perpusilla* LP8539). For each species (three replicates), columns represent peak heights for the LC-MS analysis. For each feature m/z and RT, molecular formula and the reference ion used to calculated are indicated.

Supplementary Data 4. List of isotopically labelled internal standards. Isotopically labelled internal standards used for the identification of metabolites present in the root of of *Spirodela polyrhiza* 9509 and *Lemna aequinoctialis* 7339, inoculated or not with the full synthetic community and with three synthetic communities lacking isolates A289, A245, and P65.

Supplementary Data 5. Metabolite identification and identification confidence in duckweed roots. Summary of the identifications and confidence levels for metabolites detected in the roots of *Spirodela polyrhiza* 9509 and *Lemna aequinoctialis* 7339, inoculated or not with the full synthetic community and with three synthetic communities lacking isolates A289, A245, and P65. The data were acquired using MS in positive and negative ion modes.

Supplementary Data 6. Differentially accumulated root metabolite features across nine Lemnaceae species following bacterial synthetic community inoculation. List of the 341 metabolite features with a significantly different accumulation in the roots of nine *Lemnaceae* species (LJ9250: *Lemna japonica* 9250, LY9208: *Lemna yungensis* 9208, LA7339: *Lemna aequinoctalis* 7339, LT9243: *Lemna tenera* 9243, LP0049: *Landoltia punctata* 0049, SI9227: *Spirodela intermedia* 9227, SI7820: *Spirodela intermedia* 7820, SP9509: *Spirodela polyrhiza* 9509, SP7498: *Spirodela polyrhiza* 7498) inoculated with the bacterial synthetic community compared to axenic plants (See Supplementary Figure 22c). Annotation was made using MzCloud (Thermo Scientific) and Sirius (Version 6.3.4) for MS2 fragmentation a threshold of 80% match (MzCloud) and 0.64 confidence score (Sirius) was used to report the names. For the compounds with MS2 data below the Sirius threshold chemical class is reported with ClassyFire classes (level reported after the class). For level 4 compounds with an exact mass match in Methabolika Pathways database are reported.

Supplementary Data 7. Putative root metabolites linking microbial colonisation to Lemnaceae anatomical complexity. Summary of the identifications and confidence levels of the 15 putative metabolites detected in the roots of nine *Lemnaceae* species (LJ9250: *Lemna japonica* 9250, LY9208: *Lemna yungensis* 9208, LA7339: *Lemna aequinoctalis* 7339, LT9243: *Lemna tenera* 9243, LP0049: *Landoltia punctata* 0049, SI9227: *Spirodela intermedia* 9227, SI7820: *Spirodela intermedia* 7820, SP9509: *Spirodela polyrhiza* 9509, SP7498: *Spirodela polyrhiza* 7498) that showed significantly different accumulation in plants colonised with the full synthetic community compared with axenic plants, and whose changes in abundance significantly correlated with root anatomical complexity. Data were acquired using LC-MS in both positive and negative ion modes.

Supplementary Data 8. List of Lemnaceae species and *Pistia stratiotes* used in this work.

Supplementary Data 9. Composition of the Schenck-Hildebrandt (SH) medium used for in vitro cultivation of the plants in this study.

Supplementary Data 10. Overview of LC-MS metabolomic mirror spectra data. The folders contain both positive- and negative- ion mirror spectra for the LC-MS metabolomic analysis. For each standard used in the analysis, the matching score is displayed in the graph title, and a comparison of fragmentation patterns between the measured sample and the standard is shown on the left. All fragmentations acquired within the same retention-time window are displayed in the smaller graphs, together with their similarity scores. On the right, information on the standard is shown, along with the monoisotopic mass and theoretical *m/z* of the detected adduct. At the bottom of the page, the structure of the standard, the number of quality-matching peaks, and the total number of detected peaks are listed for the fragmentation with the highest score.
