# Supplementary material for: The metabolic and anatomical complexity of root microhabitats modulate their interaction with the microbiota
Source: Nat Commun. 2026 Aug 13;17:9738. doi: 10.1038/s41467-026-76440-4 (PMC13562559; doi:10.1038/s41467-026-76440-4)
Supplement: Supplementary file 5 [file 41467_2026_76440_MOESM12_ESM.zip › Supplementary Data 10/NEG_msms_mirror_plots/0035_valine_negative_M-H116p0717_10p92.pdf]

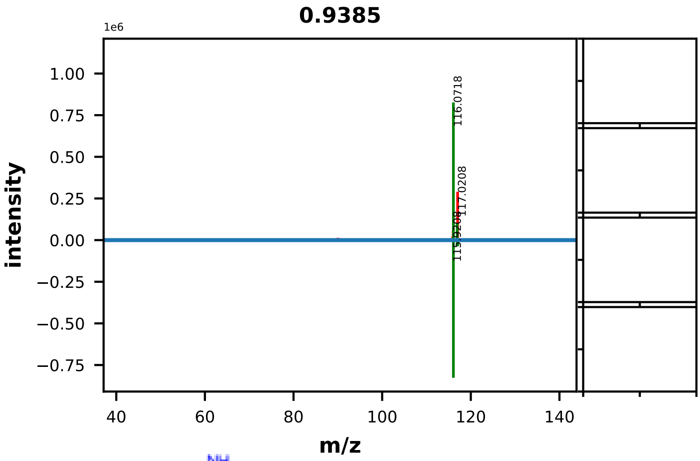

	EMA Compound Info
Name:	valine
Label:	
Formula:	C5H11NO2
Polarity:	negative
Monoisotopic Mass:	117.078978592
Theoretical M/Z:	116.0717
Adduct:	[M-H]-

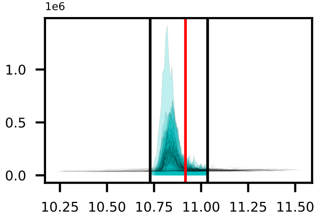

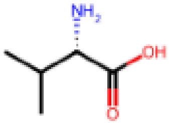

20240411_JGI_GC_509610_DWeedSyn_final_EXP120B_HILICZ_USHXG02558_NEG_MS2_14-UN-16_SP9509-dP65_4_Rg70to1050-CE102040norm-root-S1_Run188.h5

0035_valine_negative_M-H116p0717_10p92 [M-H]-
Measured M/Z = 116.0717, 0.3206 ppm difference
Expected Elution of 10.92 minutes, 10.82 min actual

MSMS Scan at 10.835 minutes

Matching M/Zs above 1E-3*max: 99.926, 116.072, 116.929, 117.075

All Matching M/Zs: 99.926, 116.072, 116.929, 117.075
