# Supplementary material for: The metabolic and anatomical complexity of root microhabitats modulate their interaction with the microbiota
Source: Nat Commun. 2026 Aug 13;17:9738. doi: 10.1038/s41467-026-76440-4 (PMC13562559; doi:10.1038/s41467-026-76440-4)
Supplement: Supplementary file 5 [file 41467_2026_76440_MOESM12_ESM.zip › Supplementary Data 10/NEG_msms_mirror_plots/0026_2deoxyguanosine_negative_M-H266p0895_6p73.pdf]

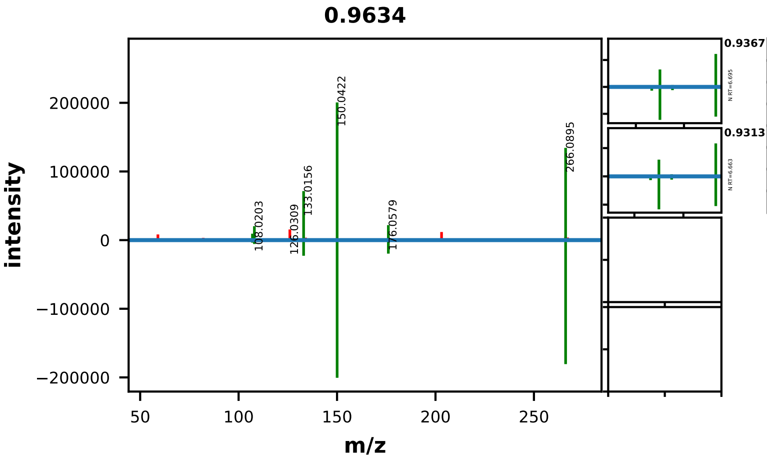

	EMA Compound Info
Name:	2'-deoxyguanosine
Label:	
Formula:	C10H13N5O4
Polarity:	negative
Monoisotopic Mass:	267.096753896
Theoretical M/Z:	266.08948
Adduct:	[M-H]-

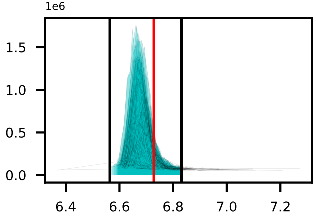

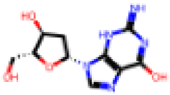

20240411_JGI_GC_509610_DWeedSyn_final_EXP120B_HILICZ_USHXG02558_NEG_MS2_54-UN-64_LJ9250-dA289_2_Rg70to1050-CE205060norm-root-S1_Run108.h5

0026_2deoxyguanosine_negative_M-H266p0895_6p73 [M-H]-
Measured M/Z = 266.0894, 0.3439 ppm difference
Expected Elution of 6.73 minutes, 6.69 min actual

MSMS Scan at 6.708 minutes

Matching M/Zs above 1E-3*max: 107.036, 108.020, 133.016, 150.042, 176.058, 266.090

All Matching M/Zs: 107.036, 108.020, 133.016, 150.042, 176.058, 266.090
