# Supplementary material for: The metabolic and anatomical complexity of root microhabitats modulate their interaction with the microbiota
Source: Nat Commun. 2026 Aug 13;17:9738. doi: 10.1038/s41467-026-76440-4 (PMC13562559; doi:10.1038/s41467-026-76440-4)
Supplement: Supplementary file 5 [file 41467_2026_76440_MOESM12_ESM.zip › Supplementary Data 10/NEG_msms_mirror_plots/0013_ribose_negative_M-H149p0455_2p77.pdf]

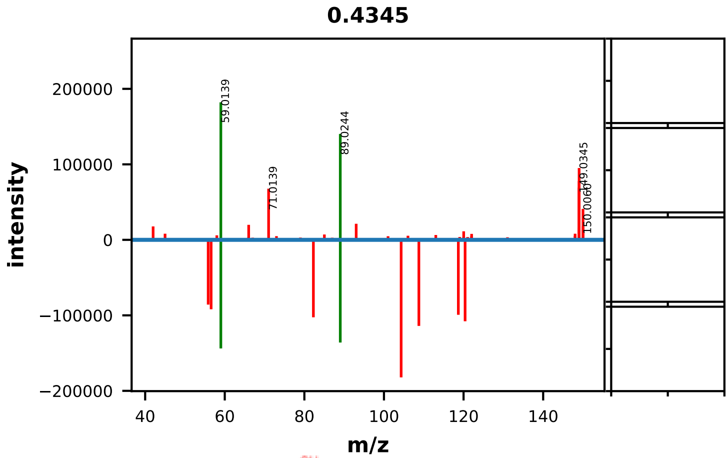

	EMA Compound Info
Name:	ribose
Label:	
Formula:	C5H10O5
Polarity:	negative
Monoisotopic Mass:	150.05282342
Theoretical M/Z:	149.04555
Adduct:	[M-H]-

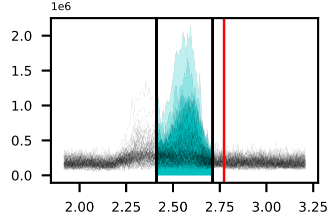

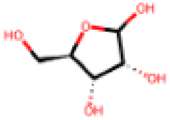

20240411_JGI_GC_509610_DWeedSyn_final_EXP120B_HILICZ_USHXG02558_NEG_MS2_14-UN-16_SP9509-dP65_4_Rg70to1050-CE102040norm-root-S1_Run188.h5

0013_ribose_negative_M-H149p0455_2p77 [M-H]-
Measured M/Z = 149.0455, 0.0979 ppm difference
Expected Elution of 2.77 minutes, 2.59 min actual

MSMS Scan at 2.583 minutes

Matching M/Zs above 1E-3*max: 59.014, 89.024

All Matching M/Zs: 59.014, 89.024
