# Supplementary material for: The metabolic and anatomical complexity of root microhabitats modulate their interaction with the microbiota
Source: Nat Commun. 2026 Aug 13;17:9738. doi: 10.1038/s41467-026-76440-4 (PMC13562559; doi:10.1038/s41467-026-76440-4)
Supplement: Supplementary file 5 [file 41467_2026_76440_MOESM12_ESM.zip › Supplementary Data 10/NEG_msms_mirror_plots/0022_allantoin_negative_M-H157p0367_4p86.pdf]

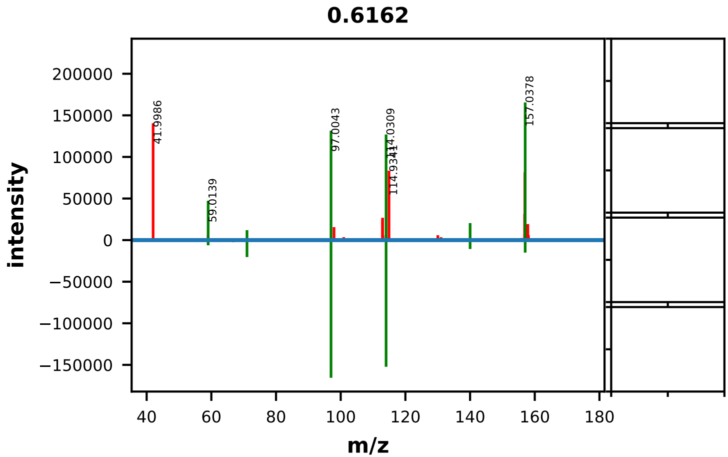

	EMA Compound Info
Name:	allantoin
Label:	
Formula:	C4H6N4O3
Polarity:	negative
Monoisotopic Mass:	158.043990052
Theoretical M/Z:	157.03671
Adduct:	[M-H]-

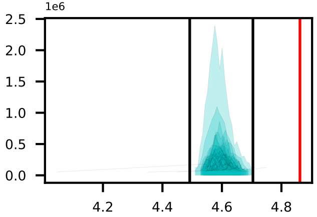

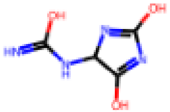

20240411_JGI_GC_509610_DWeedSyn_final_EXP120B_HILICZ_USHXG02558_NEG_MS2_14-UN-16_SP9509-dP65_4_Rg70to1050-CE102040norm-root-S1_Run188.h5

0022_allantoin_negative_M-H157p0367_4p86 [M-H]-
Measured M/Z = 157.0365, 1.3872 ppm difference
Expected Elution of 4.86 minutes, 4.58 min actual

MSMS Scan at 4.566 minutes

Matching M/Zs above 1E-3*max: 59.014, 71.025, 97.004, 114.031, 140.010, 157.038

All Matching M/Zs: 59.014, 71.025, 97.004, 114.031, 140.010, 157.038
