# Supplementary material for: The metabolic and anatomical complexity of root microhabitats modulate their interaction with the microbiota
Source: Nat Commun. 2026 Aug 13;17:9738. doi: 10.1038/s41467-026-76440-4 (PMC13562559; doi:10.1038/s41467-026-76440-4)
Supplement: Supplementary file 5 [file 41467_2026_76440_MOESM12_ESM.zip › Supplementary Data 10/NEG_msms_mirror_plots/0012_xanthine_negative_M-H151p0262_2p75.pdf]

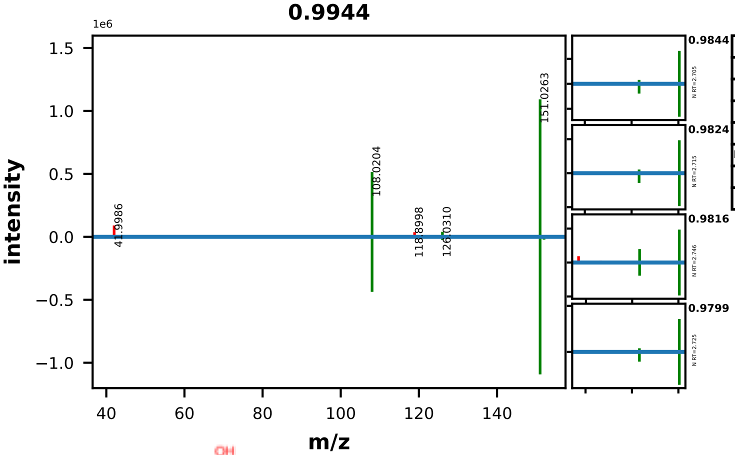

	EMA Compound Info
Name:	xanthine
Label:	
Formula:	C5H4N4O2
Polarity:	negative
Monoisotopic Mass:	152.033425368
Theoretical M/Z:	151.02615
Adduct:	[M-H]-

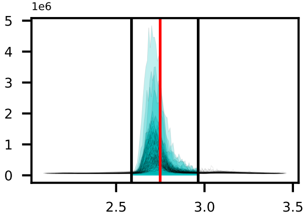

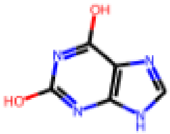

20240411_JGI_GC_509610_DWeedSyn_final_EXP120B_HILICZ_USHXG02558_NEG_MS2_10-UN-11_SP9509-Syncom_5_Rg70to1050-CE205060norm-root-S1_Run243.h5

0012_xanthine_negative_M-H151p0262_2p75 [M-H]-
Measured M/Z = 151.0261, 0.2217 ppm difference
Expected Elution of 2.75 minutes, 2.70 min actual

MSMS Scan at 2.666 minutes

Matching M/Zs above 1E-3*max: 108.020, 109.016, 126.031, 133.016, 151.026, 152.030

All Matching M/Zs: 108.020, 109.016, 126.031, 133.016, 151.026, 152.030
