# Supplementary material for: The metabolic and anatomical complexity of root microhabitats modulate their interaction with the microbiota
Source: Nat Commun. 2026 Aug 13;17:9738. doi: 10.1038/s41467-026-76440-4 (PMC13562559; doi:10.1038/s41467-026-76440-4)
Supplement: Supplementary file 5 [file 41467_2026_76440_MOESM12_ESM.zip › Supplementary Data 10/NEG_msms_mirror_plots/0018_hypoxanthine_negative_M-H135p0312_3p11.pdf]

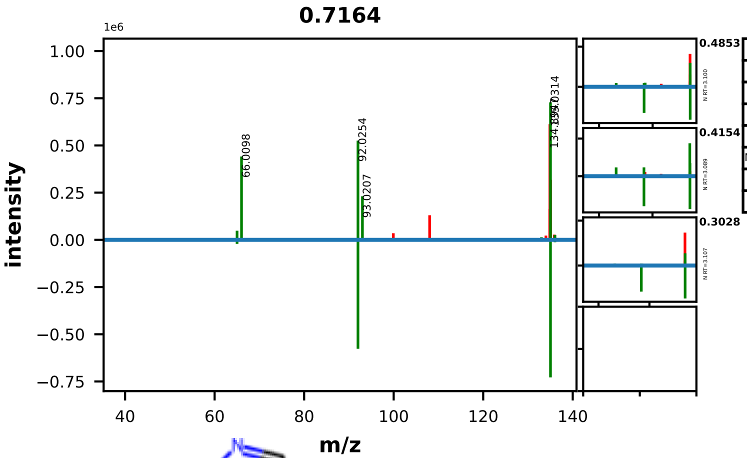


| | EMA Compound Info |
|---|---|
| Name: | hypoxanthine |
| Label: | |
| Formula: | C5H4N4O |
| Polarity: | negative |
| Monoisotopic Mass: | 136.038510748 |
| Theoretical M/Z: | 135.03123 |
| Adduct: | [M-H]- |

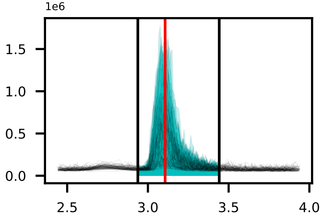


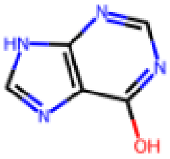


20240411_JGI_GC_509610_DWeedSyn_final_EXP120B_HILICZ_USHXG02558_NEG_MS2_10-UN-11_SP9509-Syncom_5_Rg70to1050-CE205060norm-root-S1_Run243.h5

0018_hypoxanthine_negative_M-H135p0312_3p11 [M-H]-
Measured M/Z = 135.0313, 0.1486 ppm difference
Expected Elution of 3.11 minutes, 3.09 min actual

MSMS Scan at 3.095 minutes

Matching M/Zs above 1E-3*max: 65.015, 66.010, 92.025, 93.021, 133.016, 135.031, 136.027

All Matching M/Zs: 65.015, 66.010, 92.025, 93.021, 133.016, 135.031, 136.027
