# Supplementary material for: The metabolic and anatomical complexity of root microhabitats modulate their interaction with the microbiota
Source: Nat Commun. 2026 Aug 13;17:9738. doi: 10.1038/s41467-026-76440-4 (PMC13562559; doi:10.1038/s41467-026-76440-4)
Supplement: Supplementary file 5 [file 41467_2026_76440_MOESM12_ESM.zip › Supplementary Data 10/NEG_msms_mirror_plots/0003_4-coumaric_acid_negative_M-H163p0401_1p62.pdf]

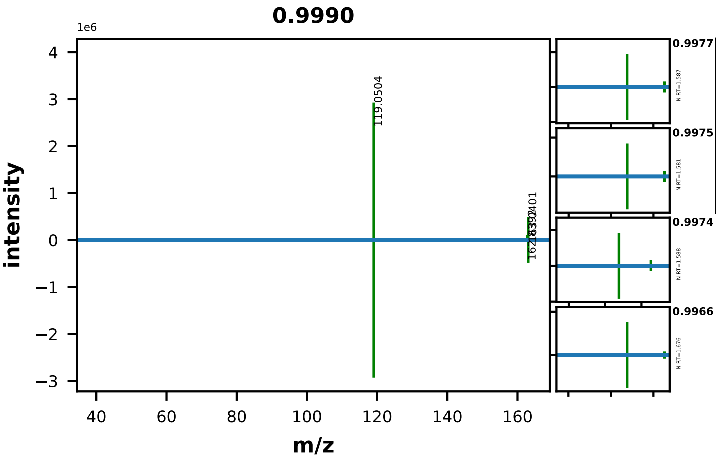

	EMA Compound Info
Name:	4-coumaric acid
Label:	
Formula:	C9H8O3
Polarity:	negative
Monoisotopic Mass:	164.047344116
Theoretical M/Z:	163.04007
Adduct:	[M-H]-

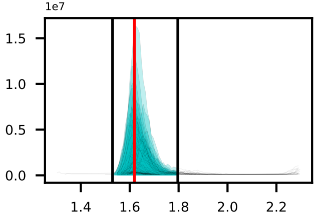

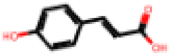

20240411_JGI_GC_509610_DWeedSyn_final_EXP120B_HILICZ_USHXG02558_NEG_MS2_27-UN-32_SP9509-mP65_2_Rg70to1050-CE205060norm-root-S1_Run118.h5

0003_4-coumaric_acid_negative_M-H163p0401_1p62 [M-H]-
Measured M/Z = 163.0401, 0.3142 ppm difference
Expected Elution of 1.62 minutes, 1.62 min actual

MSMS Scan at 1.594 minutes

Matching M/Zs above 1E-3*max: 119.050, 162.839, 163.040

All Matching M/Zs: 119.050, 162.839, 163.040
