# Supplementary material for: The metabolic and anatomical complexity of root microhabitats modulate their interaction with the microbiota
Source: Nat Commun. 2026 Aug 13;17:9738. doi: 10.1038/s41467-026-76440-4 (PMC13562559; doi:10.1038/s41467-026-76440-4)
Supplement: Supplementary file 5 [file 41467_2026_76440_MOESM12_ESM.zip › Supplementary Data 10/NEG_msms_mirror_plots/0002_uracil_negative_M-H111p0200_1p50.pdf]

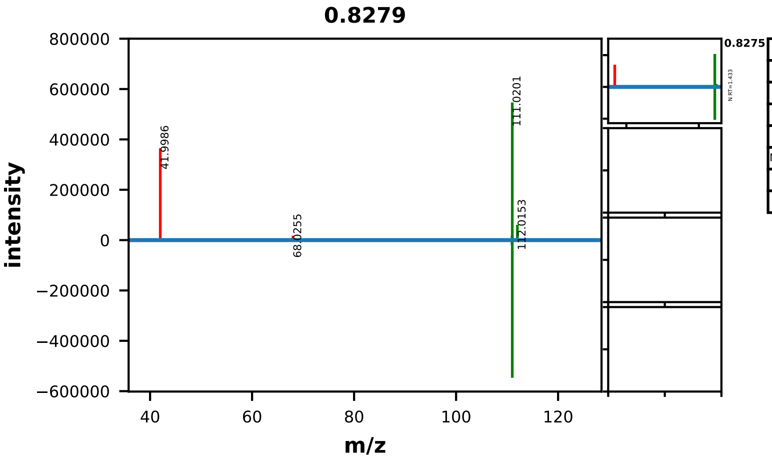


| | EMA Compound Info |
|---|---|
| Name: | uracil |
| Label: | |
| Formula: | C4H4N2O2 |
| Polarity: | negative |
| Monoisotopic Mass: | 112.027277368 |
| Theoretical M/Z: | 111.02 |
| Adduct: | [M-H]- |

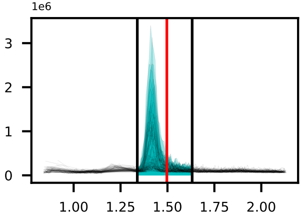


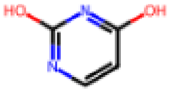


20240411_JGI_GC_509610_DWeedSyn_final_EXP120B_HILICZ_USHXG02558_NEG_MS2_10-UN-11_SP9509-Syncom_5_Rg70to1050-CE205060norm-root-S1_Run243.h5

0002_uracil_negative_M-H111p0200_1p50 [M-H]-
Measured M/Z = 111.0201, 0.4606 ppm difference
Expected Elution of 1.50 minutes, 1.41 min actual

MSMS Scan at 1.440 minutes

Matching M/Zs above 1E-3*max: 102.501, 110.976, 111.020, 112.015

All Matching M/Zs: 102.501, 110.976, 111.020, 112.015
