# Supplementary material for: The metabolic and anatomical complexity of root microhabitats modulate their interaction with the microbiota
Source: Nat Commun. 2026 Aug 13;17:9738. doi: 10.1038/s41467-026-76440-4 (PMC13562559; doi:10.1038/s41467-026-76440-4)
Supplement: Supplementary file 5 [file 41467_2026_76440_MOESM12_ESM.zip › Supplementary Data 10/NEG_msms_mirror_plots/0000_abscisic_acid_negative_M-H263p1289_1p27.pdf]

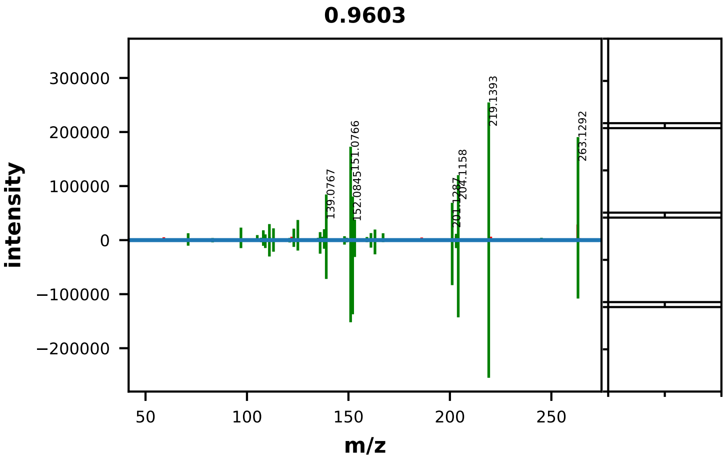

	EMA Compound Info
Name:	abscisic acid
Label:	
Formula:	C15H20O4
Polarity:	negative
Monoisotopic Mass:	264.13615912
Theoretical M/Z:	263.12888
Adduct:	[M-H]-

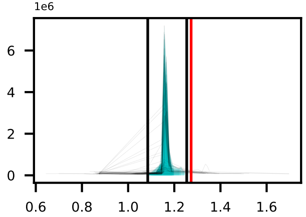

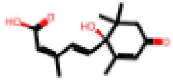

20240411_JGI_GC_509610_DWeedSyn_final_EXP120B_HILICZ_USHXG02558_NEG_MS2_0_ISTD_Post_Rg70to1050-CE102040norm--ISTD_Run259.h5

0000_abscisic_acid_negative_M-H263p1289_1p27 [M-H]-
Measured M/Z = 263.1290, 0.5002 ppm difference
Expected Elution of 1.27 minutes, 1.16 min actual

MSMS Scan at 1.162 minutes

Matching M/Zs above 1E-3*max: 71.014, 83.050, 97.030, 105.071, 107.050, 108.058, 109.030, 109.066, 111.045, 111.082, 113.061, 121.066, 123.082, 124.053, 125.061, 135.082, 136.054, 137.061, 138.069, 139.077, 148.053, 149.061, 151.077, 152.048, 152.084, 153.092, 159.118, 161.097, 163.076, 167.072, 170.059, 201.129, 203.108, 204.116, 219.139, 245.119, 263.129, 263.166

All Matching M/Zs: 71.014, 83.050, 97.030, 105.071, 107.050, 108.058, 109.030, 109.066, 111.045, 111.082, 113.061, 121.066, 123.082, 124.053, 125.061, 135.082, 136.054, 137.061, [illegible]
