# Supplementary material for: The metabolic and anatomical complexity of root microhabitats modulate their interaction with the microbiota
Source: Nat Commun. 2026 Aug 13;17:9738. doi: 10.1038/s41467-026-76440-4 (PMC13562559; doi:10.1038/s41467-026-76440-4)
Supplement: Supplementary file 5 [file 41467_2026_76440_MOESM12_ESM.zip › Supplementary Data 10/NEG_msms_mirror_plots/0046_raffinose_negative_M-H503p1618_15p41.pdf]

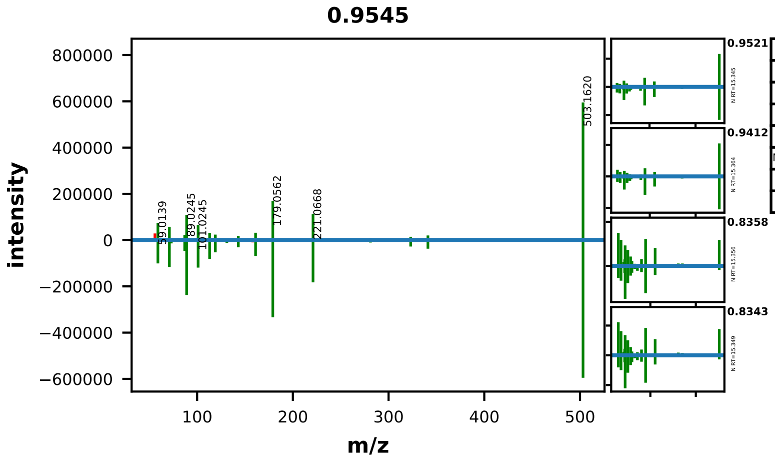

	EMA Compound Info
Name:	raffinose
Label:	
Formula:	C18H32O16
Polarity:	negative
Monoisotopic Mass:	504.169034944
Theoretical M/Z:	503.16176
Adduct:	[M-H]-

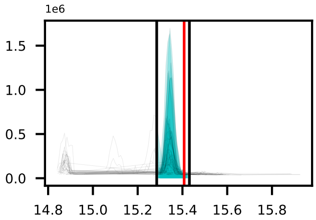

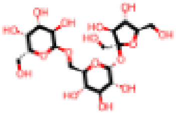

20240411_JGI_GC_509610_DWeedSyn_final_EXP120B_HILICZ_USHXG02558_NEG_MS2_45-UN-53_LJ9250-Syncom_1_Rg70to1050-CE102040norm-root-S1_Run41.h5

0046_raffinose_negative_M-H503p1618_15p41 [M-H]-
Measured M/Z = 503.1616, 0.3680 ppm difference
Expected Elution of 15.41 minutes, 15.35 min actual

MSMS Scan at 15.354 minutes

Matching M/Zs above 1E-3*max: 59.014, 71.014, 73.029, 87.009, 89.024, 101.025, 113.025, 119.035, 131.035, 143.035, 161.046, 179.056, 221.067, 281.088, 323.099, 341.109, 503.162

All Matching M/Zs: 59.014, 71.014, 73.029, 87.009, 89.024, 101.025, 113.025, 119.035, 131.035, 143.035, 161.046, 179.056, 221.067, 281.088, 323.099, 341.109, 503.162
