# Supplementary material for: The metabolic and anatomical complexity of root microhabitats modulate their interaction with the microbiota
Source: Nat Commun. 2026 Aug 13;17:9738. doi: 10.1038/s41467-026-76440-4 (PMC13562559; doi:10.1038/s41467-026-76440-4)
Supplement: Supplementary file 5 [file 41467_2026_76440_MOESM12_ESM.zip › Supplementary Data 10/NEG_msms_mirror_plots/0042_shikimic_acid_negative_M-H173p0455_13p57.pdf]

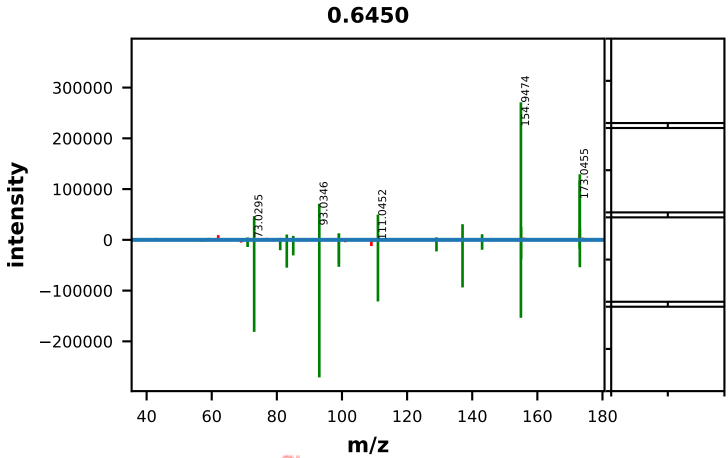

	EMA Compound Info
Name:	shikimic acid
Label:	
Formula:	C7H10O5
Polarity:	negative
Monoisotopic Mass:	174.05282342
Theoretical M/Z:	173.04555
Adduct:	[M-H]-

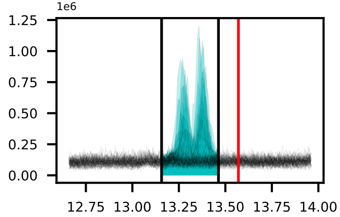

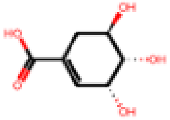

20240411_JGI_GC_509610_DWeedSyn_final_EXP120B_HILICZ_USHXG02558_NEG_MS2_14-UN-16_SP9509-dP65_4_Rg70to1050-CE102040norm-root-S1_Run188.h5

0042_shikimic_acid_negative_M-H173p0455_13p57 [M-H]-
Measured M/Z = 173.0455, 0.3087 ppm difference
Expected Elution of 13.57 minutes, 13.36 min actual

MSMS Scan at 13.372 minutes

Matching M/Zs above 1E-3*max: 55.019, 57.035, 59.014, 71.014, 73.030, 73.032, 81.035, 83.050, 85.030, 93.035, 99.009, 99.045, 111.045, 129.019, 137.024, 143.035, 154.947, 155.035, 172.958, 173.045

All Matching M/Zs: 55.019, 57.035, 59.014, 71.014, 73.030, 73.032, 81.035, 83.050, 85.030, 93.035, 99.009, 99.045, 111.045, 129.019, 137.024, 143.035, 154.947, 155.035, 172.958, 173.045
