# Supplementary material for: The metabolic and anatomical complexity of root microhabitats modulate their interaction with the microbiota
Source: Nat Commun. 2026 Aug 13;17:9738. doi: 10.1038/s41467-026-76440-4 (PMC13562559; doi:10.1038/s41467-026-76440-4)
Supplement: Supplementary file 5 [file 41467_2026_76440_MOESM12_ESM.zip › Supplementary Data 10/NEG_msms_mirror_plots/0043_serine_negative_M-H104p0353_14p16.pdf]

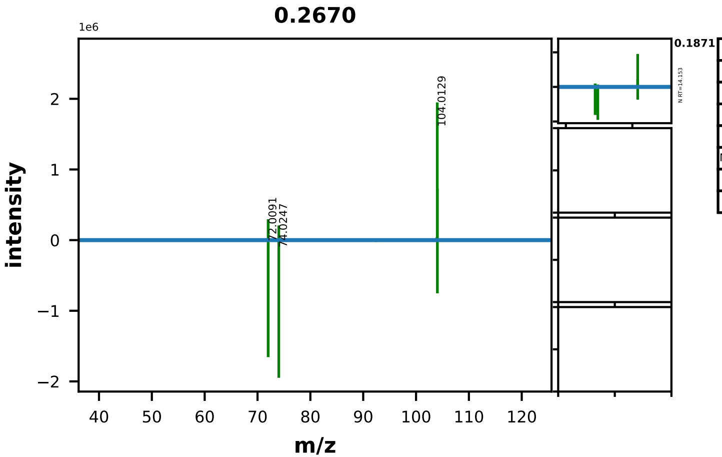

	EMA Compound Info
Name:	serine
Label:	
Formula:	C3H7NO3
Polarity:	negative
Monoisotopic Mass:	105.042593084
Theoretical M/Z:	104.03532
Adduct:	[M-H]-

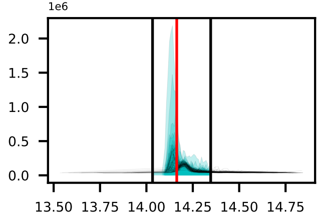

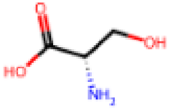

20240411_JGI_GC_509610_DWeedSyn_final_EXP120B_HILICZ_USHXG02558_NEG_MS2_14-UN-16_SP9509-dP65_4_Rg70to1050-CE102040norm-root-S1_Run188.h5

0043_serine_negative_M-H104p0353_14p16 [M-H]-
Measured M/Z = 104.0353, 0.0386 ppm difference
Expected Elution of 14.16 minutes, 14.14 min actual

MSMS Scan at 14.128 minutes

Matching M/Zs above 1E-3*max: 72.009, 74.025, 104.013, 104.035

All Matching M/Zs: 72.009, 74.025, 104.013, 104.035
