# Supplementary material for: The metabolic and anatomical complexity of root microhabitats modulate their interaction with the microbiota
Source: Nat Commun. 2026 Aug 13;17:9738. doi: 10.1038/s41467-026-76440-4 (PMC13562559; doi:10.1038/s41467-026-76440-4)
Supplement: Supplementary file 5 [file 41467_2026_76440_MOESM12_ESM.zip › Supplementary Data 10/NEG_msms_mirror_plots/0008_salicylic_acid_negative_M-H137p0244_2p25.pdf]

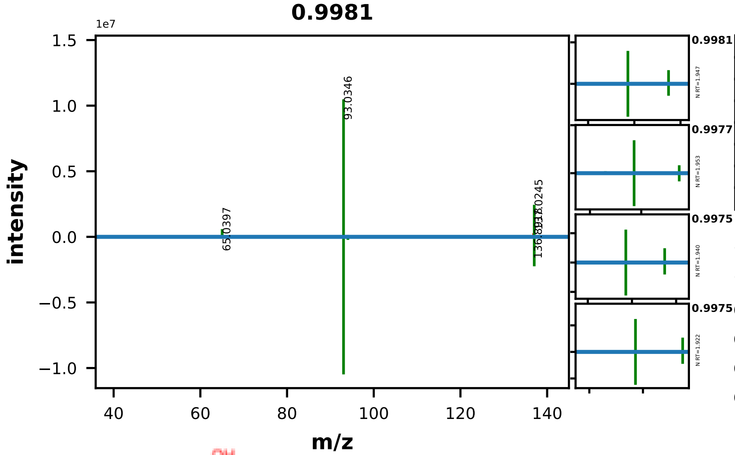

	EMA Compound Info
Name:	salicylic acid
Label:	
Formula:	C7H6O3
Polarity:	negative
Monoisotopic Mass:	138.031694052
Theoretical M/Z:	137.02442
Adduct:	[M-H]-

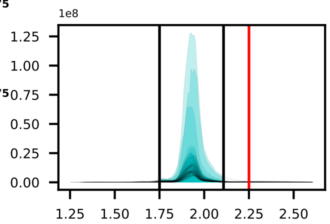

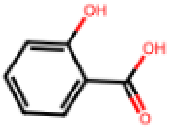

20240411_JGI_GC_509610_DWeedSyn_final_EXP120B_HILICZ_USHXG02558_NEG_MS2_55-UN-66_LJ9250-dA289_3_Rg70to1050-CE102040n205060abs-root-S1_Run124.h5

0008_salicylic_acid_negative_M-H137p0244_2p25 [M-H]-
Measured M/Z = 137.0244, 0.1238 ppm difference
Expected Elution of 2.25 minutes, 1.93 min actual

MSMS Scan at 1.953 minutes

Matching M/Zs above 1E-3*max: 65.040, 93.035, 94.038, 137.025, 138.028

All Matching M/Zs: 65.040, 93.035, 94.038, 137.025, 138.028
