# Supplementary material for: The metabolic and anatomical complexity of root microhabitats modulate their interaction with the microbiota
Source: Nat Commun. 2026 Aug 13;17:9738. doi: 10.1038/s41467-026-76440-4 (PMC13562559; doi:10.1038/s41467-026-76440-4)
Supplement: Supplementary file 5 [file 41467_2026_76440_MOESM12_ESM.zip › Supplementary Data 10/NEG_msms_mirror_plots/0016_uridine_negative_M-H243p0623_2p90.pdf]

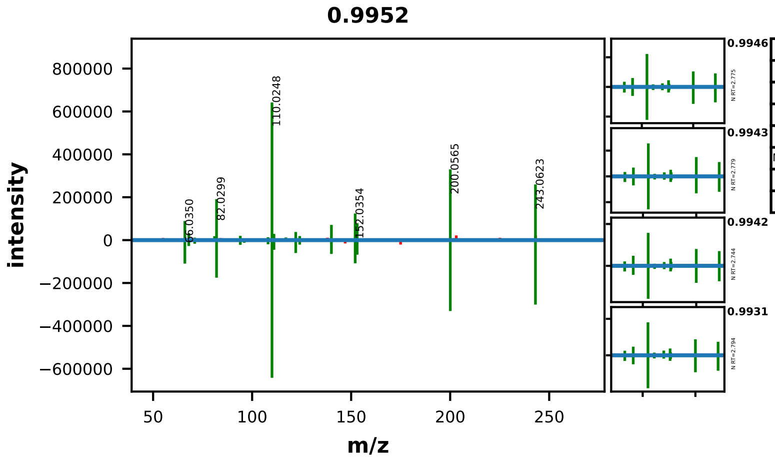

	EMA Compound Info
Name:	uridine
Label:	
Formula:	C9H12N2O6
Polarity:	negative
Monoisotopic Mass:	244.069536104
Theoretical M/Z:	243.06226
Adduct:	[M-H]-

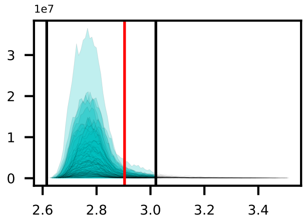

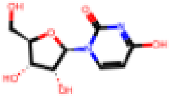

20240411_JGI_GC_509610_DWeedSyn_final_EXP120B_HILICZ_USHXG02558_NEG_MS2_18-UN-21_SP9509-dA289_3_Rg70to1050-CE102040n205060abs-root-S1_Run164.h5

0016_uridine_negative_M-H243p0623_2p90 [M-H]-
Measured M/Z = 243.0622, 0.0605 ppm difference
Expected Elution of 2.90 minutes, 2.77 min actual

MSMS Scan at 2.792 minutes

Matching M/Zs above 1E-3*max: 66.035, 68.014, 71.014, 81.022, 82.030, 83.014, 94.030, 96.046, 108.045, 110.025, 111.020, 117.019, 122.025, 124.041, 140.035, 152.035, 153.031, 182.046, 200.056, 243.062

All Matching M/Zs: 66.035, 68.014, 71.014, 81.022, 82.030, 83.014, 94.030, 96.046, 108.045, 110.025, 111.020, 117.019, 122.025, 124.041, 140.035, 152.035, 153.031, 182.046, 200.056, 243.062
