# Supplementary material for: The metabolic and anatomical complexity of root microhabitats modulate their interaction with the microbiota
Source: Nat Commun. 2026 Aug 13;17:9738. doi: 10.1038/s41467-026-76440-4 (PMC13562559; doi:10.1038/s41467-026-76440-4)
Supplement: Supplementary file 5 [file 41467_2026_76440_MOESM12_ESM.zip › Supplementary Data 10/NEG_msms_mirror_plots/0028_N-acetyl-glucosamine_negative_M+Cl256p0593_6p80.pdf]

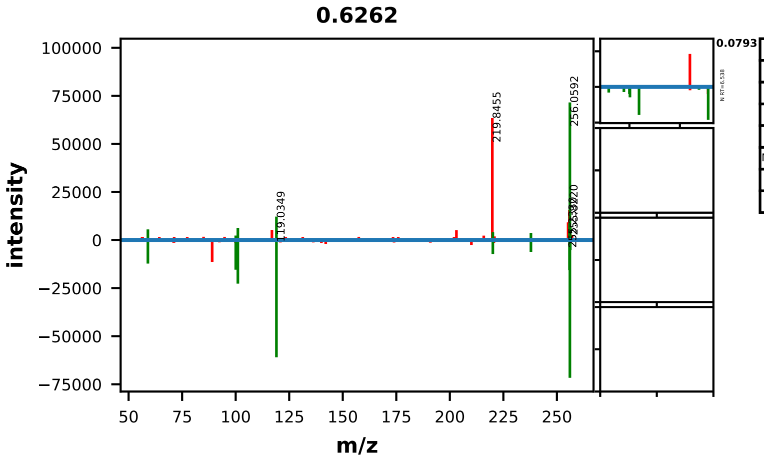

	EMA Compound Info
Name:	N-Acetyl-D-Glucosamine
Label:	
Formula:	C8H15NO6
Polarity:	negative
Monoisotopic Mass:	221.0899372
Theoretical M/Z:	256.05934
Adduct:	[M+Cl]-

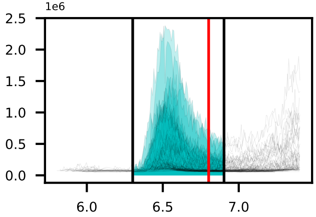

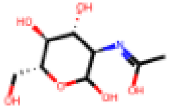

20240411_JGI_GC_509610_DWeedSyn_final_EXP120B_HILICZ_USHXG02558_NEG_MS2_14-UN-16_SP9509-dP65_4_Rg70to1050-CE102040norm-root-S1_Run188.h5

0028_N-acetyl-glucosamine_negative_M+Cl256p0593_6p80 [M+Cl]-
Measured M/Z = 256.0592, 0.3761 ppm difference
Expected Elution of 6.80 minutes, 6.51 min actual

MSMS Scan at 6.497 minutes

Matching M/Zs above 1E-3*max: 59.014, 100.040, 101.024, 119.035, 220.083, 237.870, 255.880, 256.059, 256.237

All Matching M/Zs: 59.014, 100.040, 101.024, 119.035, 220.083, 237.870, 255.880, 256.059, 256.237
