# Supplementary material for: The metabolic and anatomical complexity of root microhabitats modulate their interaction with the microbiota
Source: Nat Commun. 2026 Aug 13;17:9738. doi: 10.1038/s41467-026-76440-4 (PMC13562559; doi:10.1038/s41467-026-76440-4)
Supplement: Supplementary file 5 [file 41467_2026_76440_MOESM12_ESM.zip › Supplementary Data 10/NEG_msms_mirror_plots/0004_vanillic_acid_negative_M-H167p0350_1p65.pdf]

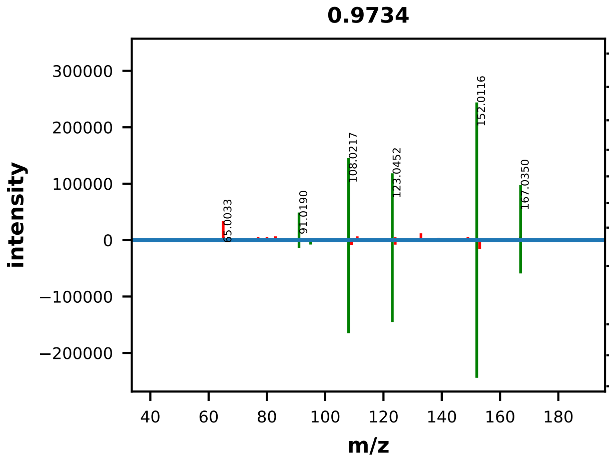

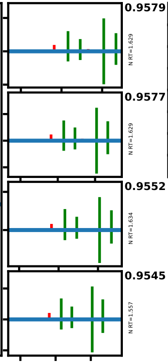

	EMA Compound Info
Name:	vanillic acid
Label:	
Formula:	C8H8O4
Polarity:	negative
Monoisotopic Mass:	168.042258736
Theoretical M/Z:	167.03498
Adduct:	[M-H]-

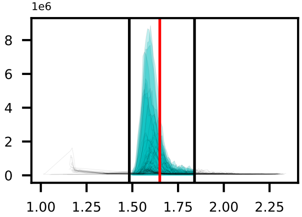

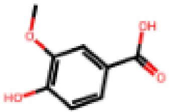

20240411_JGI_GC_509610_DWeedSyn_final_EXP120B_HILICZ_USHXG02558_NEG_MS2_18-UN-21_SP9509-dA289_3_Rg70to1050-CE102040n205060abs-root-S1_Run164.h5

0004_vanillic_acid_negative_M-H167p0350_1p65 [M-H]-
Measured M/Z = 167.0350, 0.1607 ppm difference
Expected Elution of 1.65 minutes, 1.57 min actual

MSMS Scan at 1.612 minutes

Matching M/Zs above 1E-3*max: 91.019, 95.014, 108.022, 123.009, 123.045, 152.012, 167.035

All Matching M/Zs: 91.019, 95.014, 108.022, 123.009, 123.045, 152.012, 167.035
