# Supplementary material for: The metabolic and anatomical complexity of root microhabitats modulate their interaction with the microbiota
Source: Nat Commun. 2026 Aug 13;17:9738. doi: 10.1038/s41467-026-76440-4 (PMC13562559; doi:10.1038/s41467-026-76440-4)
Supplement: Supplementary file 5 [file 41467_2026_76440_MOESM12_ESM.zip › Supplementary Data 10/NEG_msms_mirror_plots/0020_suberic_acid_negative_M-H173p0819_3p36.pdf]

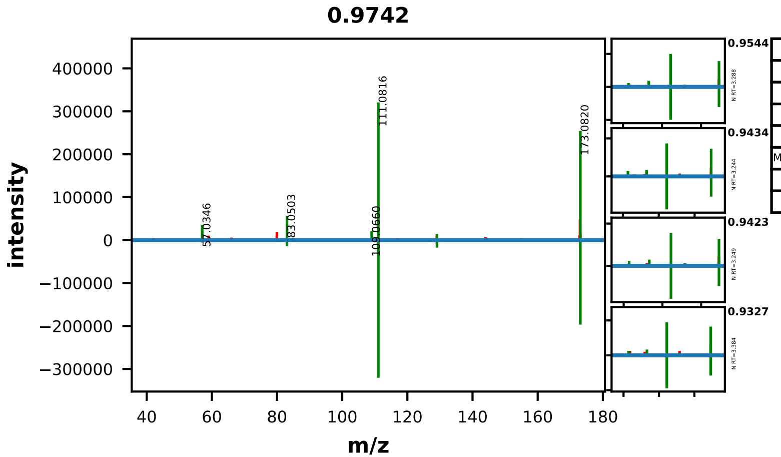


| | EMA Compound Info |
|---|---|
| Name: | suberic acid |
| Label: | |
| Formula: | C8H14O4 |
| Polarity: | negative |
| Monoisotopic Mass: | 174.089208928 |
| Theoretical M/Z: | 173.08193 |
| Adduct: | [M-H]- |

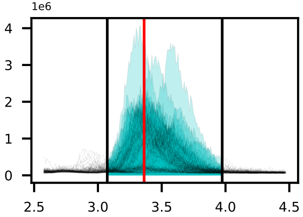


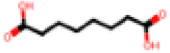

20240411_JGI_GC_509610_DWeedSyn_final_EXP120B_HILICZ_USHXG02558_NEG_MS2_12-UN-14_SP9509-dP65_2_Rg70to1050-CE205060norm-root-S1_Run102.h5

0020_suberic_acid_negative_M-H173p0819_3p36 [M-H]-
Measured M/Z = 173.0819, 0.1910 ppm difference
Expected Elution of 3.36 minutes, 3.42 min actual

MSMS Scan at 3.420 minutes

Matching M/Zs above 1E-3*max: 57.035, 69.035, 83.050, 109.066, 111.082, 129.092, 173.082

All Matching M/Zs: 57.035, 69.035, 83.050, 109.066, 111.082, 129.092, 173.082
