# Supplementary material for: The metabolic and anatomical complexity of root microhabitats modulate their interaction with the microbiota
Source: Nat Commun. 2026 Aug 13;17:9738. doi: 10.1038/s41467-026-76440-4 (PMC13562559; doi:10.1038/s41467-026-76440-4)
Supplement: Supplementary file 5 [file 41467_2026_76440_MOESM12_ESM.zip › Supplementary Data 10/NEG_msms_mirror_plots/0006_3-hydroxybutanoic_acid_negative_M-H103p0401_1p76.pdf]

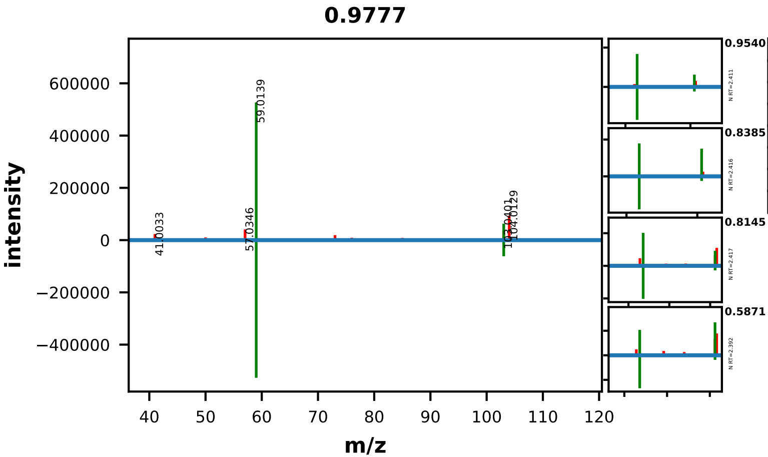

	EMA Compound Info
Name:	3-hydroxybutanoic acid
Label:	
Formula:	C4H8O3
Polarity:	negative
Monoisotopic Mass:	104.047344116
Theoretical M/Z:	103.04007
Adduct:	[M-H]-

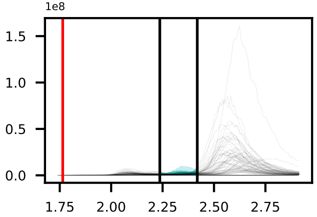

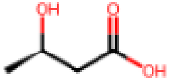

20240411_JGI_GC_509610_DWeedSyn_final_EXP120B_HILICZ_USHXG02558_NEG_MS2_43-UN-51_LJ9250-NB_3_Rg70to1050-CE102040n205060abs-root-S1_Run161.h5

0006_3-hydroxybutanoic_acid_negative_M-H103p0401_1p76 [M-H]-
Measured M/Z = 103.0401, 0.1351 ppm difference
Expected Elution of 1.76 minutes, 2.42 min actual

MSMS Scan at 2.416 minutes

Matching M/Zs above 1E-3*max: 59.014, 103.040

All Matching M/Zs: 59.014, 103.040
