# Supplementary material for: The metabolic and anatomical complexity of root microhabitats modulate their interaction with the microbiota
Source: Nat Commun. 2026 Aug 13;17:9738. doi: 10.1038/s41467-026-76440-4 (PMC13562559; doi:10.1038/s41467-026-76440-4)
Supplement: Supplementary file 5 [file 41467_2026_76440_MOESM12_ESM.zip › Supplementary Data 10/NEG_msms_mirror_plots/0053_Gibberellin_A7_negative_M-H329p1395_1p41.pdf]

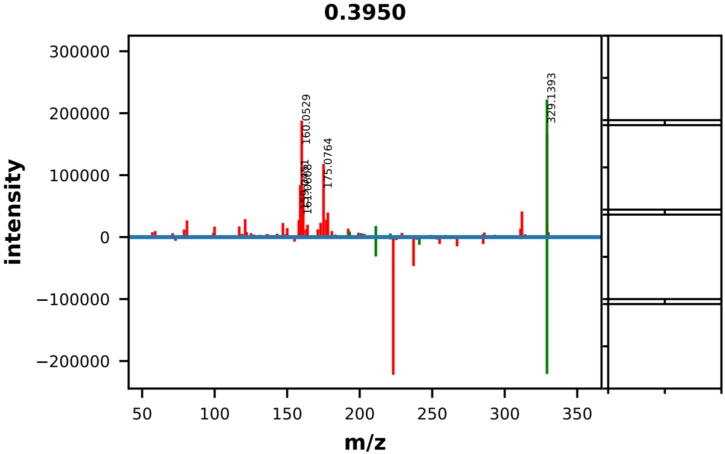

	EMA Compound Info
Name:	Gibberellin A7
Label:	
Formula:	C19H22O5
Polarity:	negative
Monoisotopic Mass:	330.146723804
Theoretical M/Z:	329.13945
Adduct:	[M-H]-

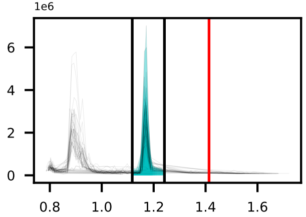

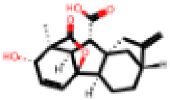

20240411_JGI_GC_509610_DWeedSyn_final_EXP120B_HILICZ_USHXG02558_NEG_MS2_15-UN-17_SP9509-dP65_5_Rg70to1050-CE205060norm-root-S1_Run237.h5

0053_Gibberellin_A7_negative_M-H329p1395_1p41 [M-H]-
Measured M/Z = 329.1394, 0.2556 ppm difference
Expected Elution of 1.41 minutes, 1.17 min actual

MSMS Scan at 1.187 minutes

Matching M/Zs above 1E-3*max: 71.050, 193.087, 197.118, 201.113, 211.134, 221.154, 241.145, 329.139

All Matching M/Zs: 71.050, 193.087, 197.118, 201.113, 211.134, 221.154, 241.145, 329.139
