# Supplementary material for: The metabolic and anatomical complexity of root microhabitats modulate their interaction with the microbiota
Source: Nat Commun. 2026 Aug 13;17:9738. doi: 10.1038/s41467-026-76440-4 (PMC13562559; doi:10.1038/s41467-026-76440-4)
Supplement: Supplementary file 5 [file 41467_2026_76440_MOESM12_ESM.zip › Supplementary Data 10/NEG_msms_mirror_plots/0010_adenine_negative_M-H134p0472_2p59.pdf]

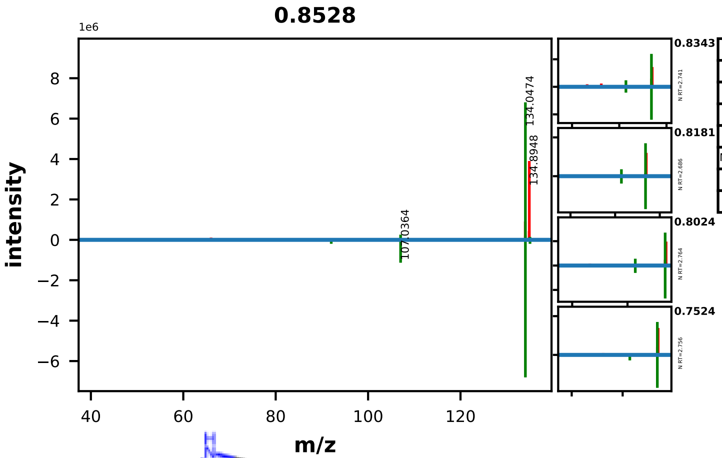

	EMA Compound Info
Name:	adenine
Label:	
Formula:	C5H5N5
Polarity:	negative
Monoisotopic Mass:	135.05449516
Theoretical M/Z:	134.04722
Adduct:	[M-H]-

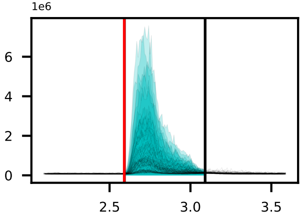

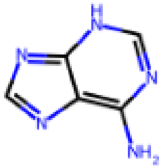

20240411_JGI_GC_509610_DWeedSyn_final_EXP120B_HILICZ_USHXG02558_NEG_MS2_14-UN-16_SP9509-dP65_4_Rg70to1050-CE102040norm-root-S1_Run188.h5

0010_adenine_negative_M-H134p0472_2p59 [M-H]-
Measured M/Z = 134.0472, 0.1656 ppm difference
Expected Elution of 2.59 minutes, 2.74 min actual

MSMS Scan at 2.754 minutes

Matching M/Zs above 1E-3*max: 92.025, 107.024, 107.036, 134.047, 135.043

All Matching M/Zs: 92.025, 107.024, 107.036, 134.047, 135.043
