# Supplementary material for: The metabolic and anatomical complexity of root microhabitats modulate their interaction with the microbiota
Source: Nat Commun. 2026 Aug 13;17:9738. doi: 10.1038/s41467-026-76440-4 (PMC13562559; doi:10.1038/s41467-026-76440-4)
Supplement: Supplementary file 5 [file 41467_2026_76440_MOESM12_ESM.zip › Supplementary Data 10/NEG_msms_mirror_plots/0011_alpha-hydroxyisobutyric_acid_negative_M-H103p0401_2p73.pdf]

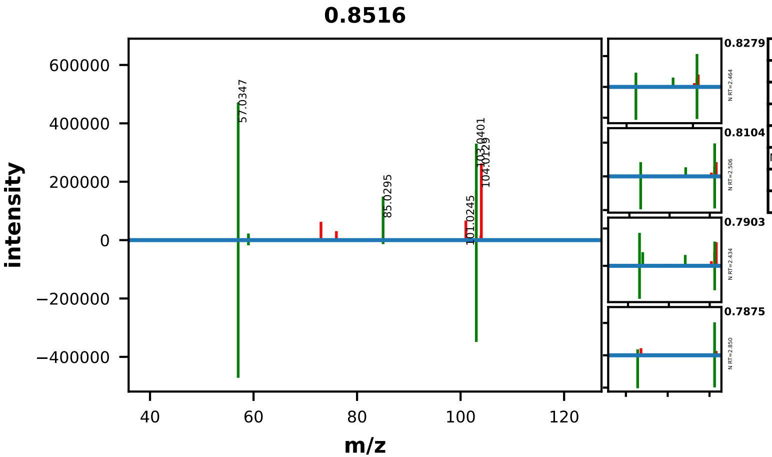

	EMA Compound Info
Name:	alpha-hydroxyisobutyric acid
Label:	
Formula:	C4H8O3
Polarity:	negative
Monoisotopic Mass:	104.047344116
Theoretical M/Z:	103.04007
Adduct:	[M-H]-

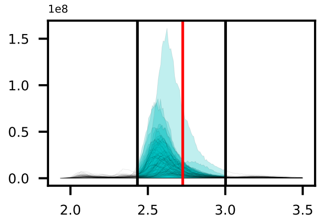

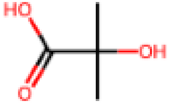

20240411_JGI_GC_509610_DWeedSyn_final_EXP120B_HILICZ_USHXG02558_NEG_MS2_12-UN-14_SP9509-dP65_2_Rg70to1050-CE205060norm-root-S1_Run102.h5

0011_alpha-hydroxyisobutyric_acid_negative_M-H103p0401_2p73 [M-H]-
Measured M/Z = 103.0401, 0.1232 ppm difference
Expected Elution of 2.73 minutes, 2.62 min actual

MSMS Scan at 2.435 minutes

Matching M/Zs above 1E-3*max: 57.035, 59.014, 85.030, 103.040

All Matching M/Zs: 57.035, 59.014, 85.030, 103.040
