# Supplementary material for: The metabolic and anatomical complexity of root microhabitats modulate their interaction with the microbiota
Source: Nat Commun. 2026 Aug 13;17:9738. doi: 10.1038/s41467-026-76440-4 (PMC13562559; doi:10.1038/s41467-026-76440-4)
Supplement: Supplementary file 5 [file 41467_2026_76440_MOESM12_ESM.zip › Supplementary Data 10/NEG_msms_mirror_plots/0007_azelaic_acid_negative_M-H187p0976_2p19.pdf]

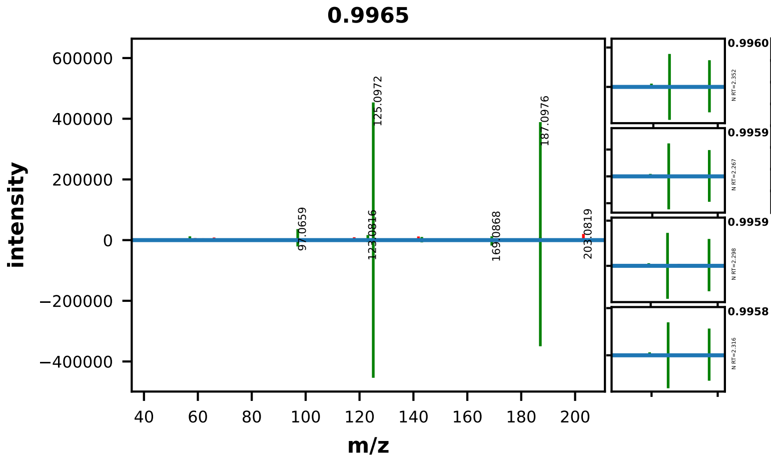

	EMA Compound Info
Name:	azelaic acid
Label:	
Formula:	C9H16O4
Polarity:	negative
Monoisotopic Mass:	188.104858992
Theoretical M/Z:	187.09758
Adduct:	[M-H]-

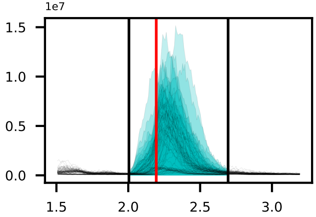

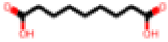

20240411_JGI_GC_509610_DWeedSyn_final_EXP120B_HILICZ_USHXG02558_NEG_MS2_47-UN-56_LJ9250-Syncom_3_Rg70to1050-CE102040n205060abs-root-S1_Run145.h5

0007_azelaic_acid_negative_M-H187p0976_2p19 [M-H]-
Measured M/Z = 187.0976, 0.0626 ppm difference
Expected Elution of 2.19 minutes, 2.30 min actual

MSMS Scan at 2.298 minutes

Matching M/Zs above 1E-3*max: 57.035, 97.066, 123.082, 125.097, 143.108, 169.087, 187.098

All Matching M/Zs: 57.035, 97.066, 123.082, 125.097, 143.108, 169.087, 187.098
