# Supplementary material for: The metabolic and anatomical complexity of root microhabitats modulate their interaction with the microbiota
Source: Nat Commun. 2026 Aug 13;17:9738. doi: 10.1038/s41467-026-76440-4 (PMC13562559; doi:10.1038/s41467-026-76440-4)
Supplement: Supplementary file 5 [file 41467_2026_76440_MOESM12_ESM.zip › Supplementary Data 10/NEG_msms_mirror_plots/0050_12-oxophytodienoic_acid_negative_M-H291p1966_1p13.pdf]

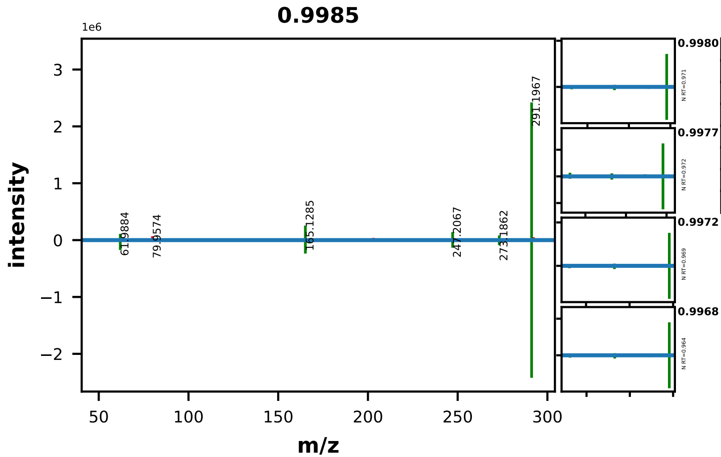

	EMA Compound Info
Name:	12-oxophytodienoic acid
Label:	
Formula:	C18H28O3
Polarity:	negative
Monoisotopic Mass:	292.203844756
Theoretical M/Z:	291.19657
Adduct:	[M-H]-

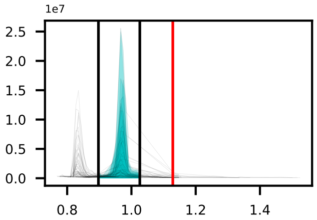

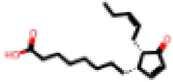

20240411_JGI_GC_509610_DWeedSyn_final_EXP120B_HILICZ_U
SHXG02558_NEG_MS2_35-UN-41_SP9509-mA289_5_Rg70to1050-
CE205060norm-root-S1_Run250.h5

0050_12-oxophytodienoic_acid_negative_M-H291p1966_1p13 [M-H]-
Measured M/Z = 291.1965, 0.0904 ppm difference
Expected Elution of 1.13 minutes, 0.97 min actual

MSMS Scan at 0.969 minutes

Matching M/Zs above 1E-3*max: 59.014, 61.988, 72.800, 148.090, 151.113, 165.128, 247.207, 273.186, 291.197

All Matching M/Zs: 59.014, 61.988, 72.800, 148.090, 151.113, 165.128, 247.207, 273.186, 291.197
