# Supplementary material for: The metabolic and anatomical complexity of root microhabitats modulate their interaction with the microbiota
Source: Nat Commun. 2026 Aug 13;17:9738. doi: 10.1038/s41467-026-76440-4 (PMC13562559; doi:10.1038/s41467-026-76440-4)
Supplement: Supplementary file 5 [file 41467_2026_76440_MOESM12_ESM.zip › Supplementary Data 10/NEG_msms_mirror_plots/0036_3-dehydroshikimic_acid_negative_M-H171p0299_11p23.pdf]

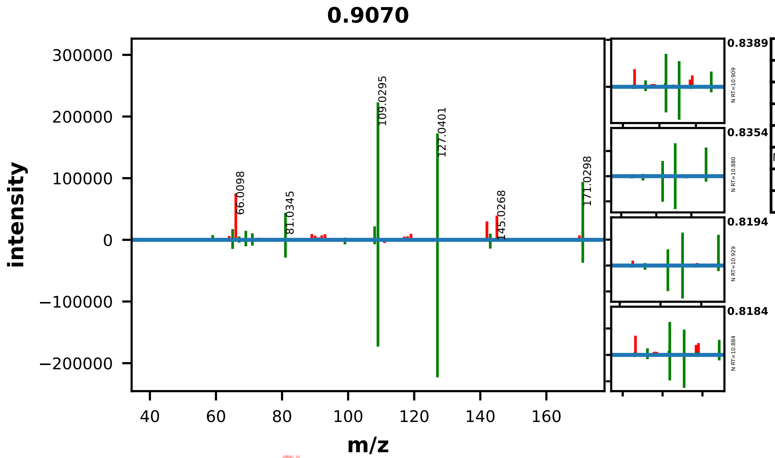

	EMA Compound Info
Name:	3-dehydroshikimic acid
Label:	
Formula:	C7H8O5
Polarity:	negative
Monoisotopic Mass:	172.037173356
Theoretical M/Z:	171.0299
Adduct:	[M-H]-

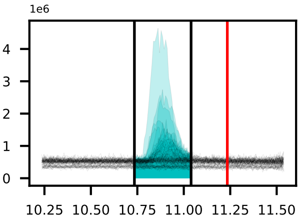

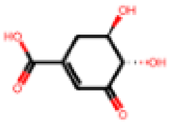

20240411_JGI_GC_509610_DWeedSyn_final_EXP120B_HILICZ_USHXG02558_NEG_MS2_20-UN-23_SP9509-dA289_5_Rg70to1050-CE205060norm-root-S1_Run228.h5

0036_3-dehydroshikimic_acid_negative_M-H171p0299_11p23 [M-H]-
Measured M/Z = 171.0299, 0.1794 ppm difference
Expected Elution of 11.23 minutes, 10.87 min actual

MSMS Scan at 10.910 minutes

Matching M/Zs above 1E-3*max: 59.014, 65.040, 67.019, 69.035, 71.014, 71.050, 81.035, 99.045, 108.022, 109.030, 127.040, 143.024, 171.030

All Matching M/Zs: 59.014, 65.040, 67.019, 69.035, 71.014, 71.050, 81.035, 99.045, 108.022, 109.030, 127.040, 143.024, 171.030
