# Supplementary material for: The metabolic and anatomical complexity of root microhabitats modulate their interaction with the microbiota
Source: Nat Commun. 2026 Aug 13;17:9738. doi: 10.1038/s41467-026-76440-4 (PMC13562559; doi:10.1038/s41467-026-76440-4)
Supplement: Supplementary file 5 [file 41467_2026_76440_MOESM12_ESM.zip › Supplementary Data 10/NEG_msms_mirror_plots/0017_mandelic_acid_negative_M-H151p0401_3p05.pdf]

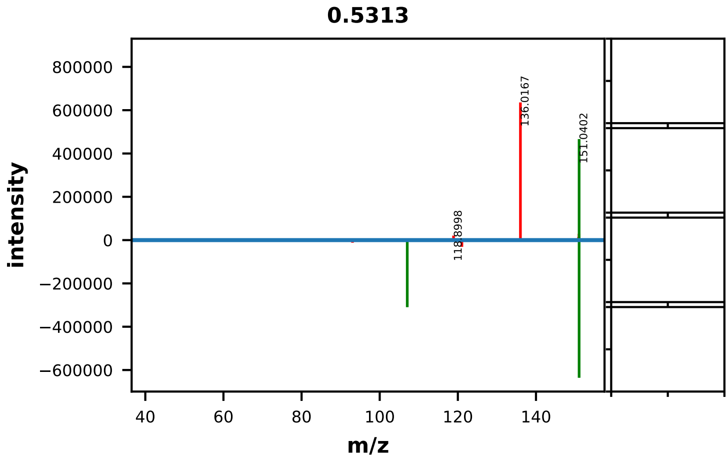

	EMA Compound Info
Name:	mandelic acid
Label:	
Formula:	C8H8O3
Polarity:	negative
Monoisotopic Mass:	152.047344116
Theoretical M/Z:	151.04007
Adduct:	[M-H]-

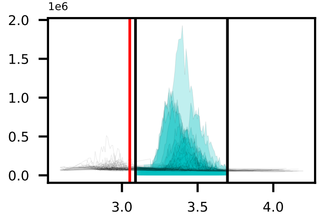

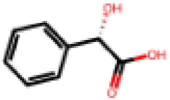

20240411_JGI_GC_509610_DWeedSyn_final_EXP120B_HILICZ_USHXG02558_NEG_MS2_29-UN-34_SP9509-mP65_4_Rg70to1050-CE102040norm-root-S1_Run213.h5

0017_mandelic_acid_negative_M-H151p0401_3p05 [M-H]-
Measured M/Z = 151.0401, 0.0749 ppm difference
Expected Elution of 3.05 minutes, 3.40 min actual

MSMS Scan at 3.373 minutes

Matching M/Zs above 1E-3*max: 107.050, 151.040

All Matching M/Zs: 107.050, 151.040
