# Supplementary material for: The metabolic and anatomical complexity of root microhabitats modulate their interaction with the microbiota
Source: Nat Commun. 2026 Aug 13;17:9738. doi: 10.1038/s41467-026-76440-4 (PMC13562559; doi:10.1038/s41467-026-76440-4)
Supplement: Supplementary file 5 [file 41467_2026_76440_MOESM12_ESM.zip › Supplementary Data 10/NEG_msms_mirror_plots/0001_ferulic_acid_negative_M-H193p0506_1p42.pdf]

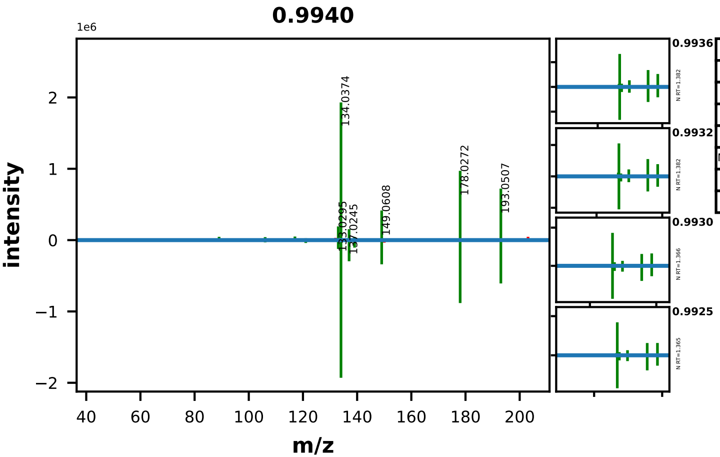

	EMA Compound Info
Name:	ferulic acid
Label:	
Formula:	C10H10O4
Polarity:	negative
Monoisotopic Mass:	194.0579088
Theoretical M/Z:	193.05063
Adduct:	[M-H]-

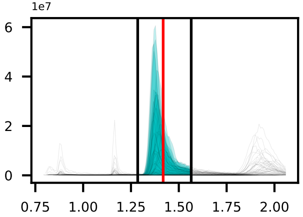

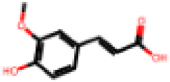

20240411_JGI_GC_509610_DWeedSyn_final_EXP120B_HILICZ_USHXG02558_NEG_MS2_18-UN-21_SP9509-dA289_3_Rg70to1050-CE102040n205060abs-root-S1_Run164.h5

0001_ferulic_acid_negative_M-H193p0506_1p42 [M-H]-
Measured M/Z = 193.0506, 0.0870 ppm difference
Expected Elution of 1.42 minutes, 1.37 min actual

MSMS Scan at 1.364 minutes

Matching M/Zs above 1E-3*max: 89.040, 106.042, 117.035, 121.030, 133.030, 134.037, 137.025, 139.040, 149.061, 178.027, 193.051

All Matching M/Zs: 89.040, 106.042, 117.035, 121.030, 133.030, 134.037, 137.025, 139.040, 149.061, 178.027, 193.051
