# Supplementary material for: The metabolic and anatomical complexity of root microhabitats modulate their interaction with the microbiota
Source: Nat Commun. 2026 Aug 13;17:9738. doi: 10.1038/s41467-026-76440-4 (PMC13562559; doi:10.1038/s41467-026-76440-4)
Supplement: Supplementary file 5 [file 41467_2026_76440_MOESM12_ESM.zip › Supplementary Data 10/NEG_msms_mirror_plots/0005_thymidine_negative_M-H241p0830_1p69.pdf]

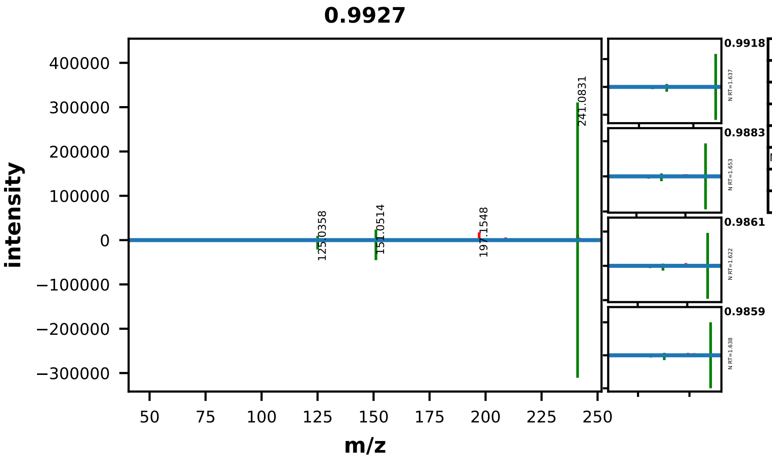

	EMA Compound Info
Name:	thymidine
Label:	
Formula:	C10H14N2O5
Polarity:	negative
Monoisotopic Mass:	242.090271548
Theoretical M/Z:	241.083
Adduct:	[M-H]-

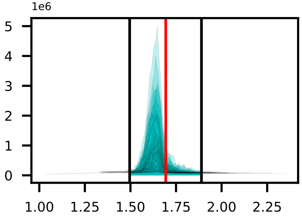

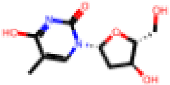

20240411_JGI_GC_509610_DWeedSyn_final_EXP120B_HILICZ_USHXG02558_NEG_MS2_41-UN-48_SP9509-NB_1_Rg70to1050-CE102040norm-root-S1_Run44.h5

0005_thymidine_negative_M-H241p0830_1p69 [M-H]-
Measured M/Z = 241.0831, 0.2329 ppm difference
Expected Elution of 1.69 minutes, 1.65 min actual

MSMS Scan at 1.628 minutes

Matching M/Zs above 1E-3*max: 125.036, 151.051, 240.871, 241.083

All Matching M/Zs: 125.036, 151.051, 240.871, 241.083
