# Supplementary material for: The metabolic and anatomical complexity of root microhabitats modulate their interaction with the microbiota
Source: Nat Commun. 2026 Aug 13;17:9738. doi: 10.1038/s41467-026-76440-4 (PMC13562559; doi:10.1038/s41467-026-76440-4)
Supplement: Supplementary file 5 [file 41467_2026_76440_MOESM12_ESM.zip › Supplementary Data 10/NEG_msms_mirror_plots/0051_9_10-dihydrojasmonic_acid_negative_M-H211p1340_1p18.pdf]

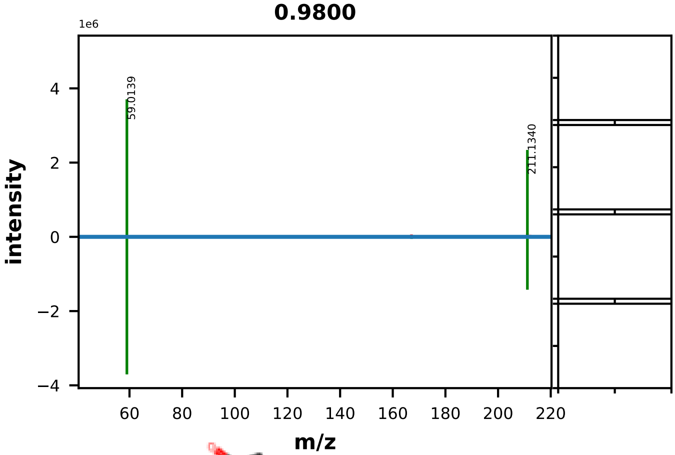

	EMA Compound Info
Name:	9,10-dihydrojasmonic acid
Label:	
Formula:	C12H20O3
Polarity:	negative
Monoisotopic Mass:	212.1412445
Theoretical M/Z:	211.13397
Adduct:	[M-H]-

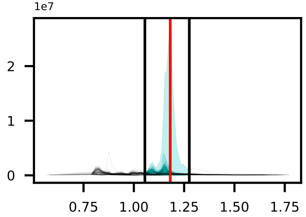

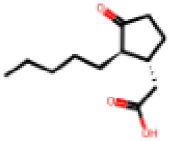

20240411_JGI_GC_509610_DWeedSyn_final_EXP120B_HILICZ_U
SHXG02558_NEG_MS2_11-UN-13_SP9509-dP65_1_Rg70to1050-
CE102040norm-root-S1_Run62.h5

0051_9_10-dihydrojasmonic_acid_negative_M-H211p1340_1p18 [M-H]-
Measured M/Z = 211.1339, 0.1282 ppm difference
Expected Elution of 1.18 minutes, 1.18 min actual

MSMS Scan at 1.153 minutes

Matching M/Zs above 1E-3*max: 52.783, 52.784, 59.003, 59.010, 59.011, 59.014, 59.018, 167.144, 211.134

All Matching M/Zs: 52.783, 52.784, 59.003, 59.010, 59.011, 59.014, 59.018, 167.144, 211.134
