# Supplementary material for: The metabolic and anatomical complexity of root microhabitats modulate their interaction with the microbiota
Source: Nat Commun. 2026 Aug 13;17:9738. doi: 10.1038/s41467-026-76440-4 (PMC13562559; doi:10.1038/s41467-026-76440-4)
Supplement: Supplementary file 5 [file 41467_2026_76440_MOESM12_ESM.zip › Supplementary Data 10/NEG_msms_mirror_plots/0014_4-pyridoxic_acid_negative_M-H182p0459_2p88.pdf]

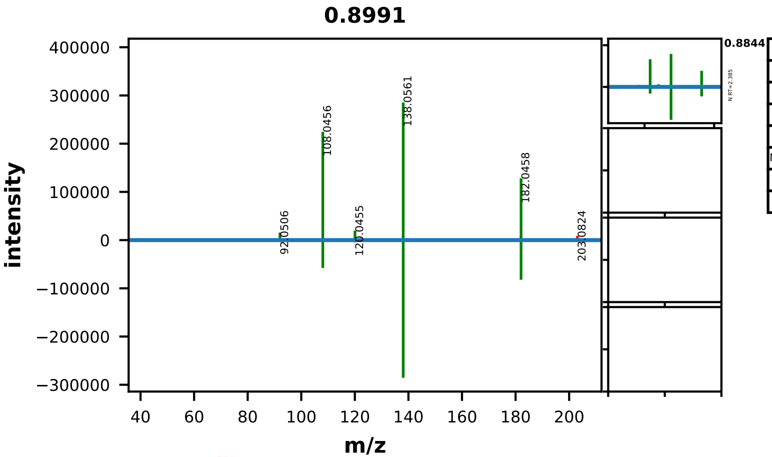

	EMA Compound Info
Name:	4-pyridoxic acid
Label:	
Formula:	C8H9NO4
Polarity:	negative
Monoisotopic Mass:	183.053157768
Theoretical M/Z:	182.04588
Adduct:	[M-H]-

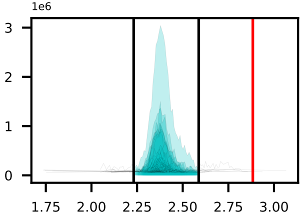

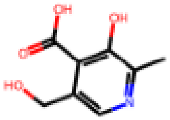

20240411_JGI_GC_509610_DWeedSyn_final_EXP120B_HILICZ_USHXG02558_NEG_MS2_17-UN-20_SP9509-dA289_2_Rg70to1050-CE205060norm-root-S1_Run93.h5

0014_4-pyridoxic_acid_negative_M-H182p0459_2p88 [M-H]-
Measured M/Z = 182.0458, 0.3692 ppm difference
Expected Elution of 2.88 minutes, 2.38 min actual

MSMS Scan at 2.344 minutes

Matching M/Zs above 1E-3*max: 92.051, 94.030, 108.046, 120.045, 136.040, 138.056, 182.046

All Matching M/Zs: 92.051, 94.030, 108.046, 120.045, 136.040, 138.056, 182.046
