# Supplementary material for: The metabolic and anatomical complexity of root microhabitats modulate their interaction with the microbiota
Source: Nat Commun. 2026 Aug 13;17:9738. doi: 10.1038/s41467-026-76440-4 (PMC13562559; doi:10.1038/s41467-026-76440-4)
Supplement: Supplementary file 5 [file 41467_2026_76440_MOESM12_ESM.zip › Supplementary Data 10/NEG_msms_mirror_plots/0052_Luteolin_negative_M-H285p0405_1p38.pdf]

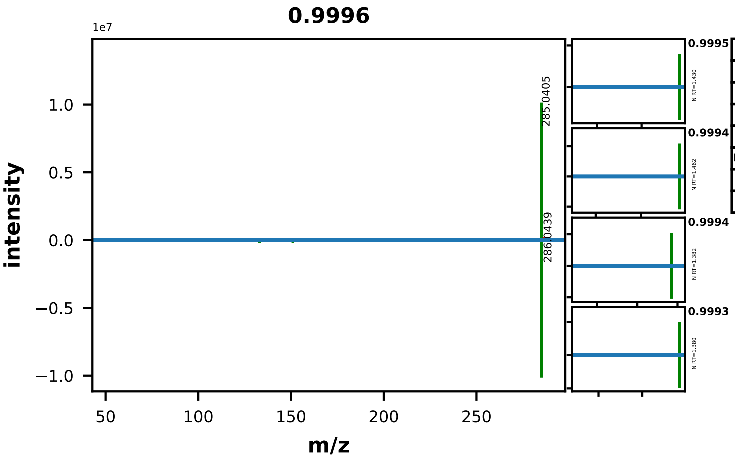

	EMA Compound Info
Name:	Luteolin
Label:	
Formula:	C15H10O6
Polarity:	negative
Monoisotopic Mass:	286.04773804
Theoretical M/Z:	285.04046
Adduct:	[M-H]-

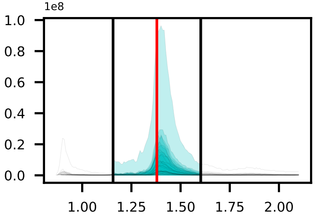

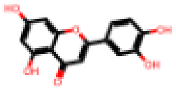

20240411_JGI_GC_509610_DWeedSyn_final_EXP120B_HILICZ_USHXG02558_NEG_MS2_21-UN-25_SP9509-dA245_1_Rg70to1050-CE102040norm-root-S1_Run56.h5

0052_Luteolin_negative_M-H285p0405_1p38 [M-H]-
Measured M/Z = 285.0405, 0.0682 ppm difference
Expected Elution of 1.38 minutes, 1.40 min actual

MSMS Scan at 1.429 minutes

Matching M/Zs above 1E-3*max: 107.014, 133.029, 149.024, 151.004, 175.040, 197.061, 199.040, 201.019, 213.056, 215.035, 217.050, 241.051, 243.029, 285.041

All Matching M/Zs: 107.014, 133.029, 149.024, 151.004, 175.040, 197.061, 199.040, 201.019, 213.056, 215.035, 217.050, 241.051, 243.029, 285.041
