# Supplementary material for: The metabolic and anatomical complexity of root microhabitats modulate their interaction with the microbiota
Source: Nat Commun. 2026 Aug 13;17:9738. doi: 10.1038/s41467-026-76440-4 (PMC13562559; doi:10.1038/s41467-026-76440-4)

	EMA Compound Info
Name:	isoleucine
Label:	
Formula:	C6H13NO2
Polarity:	negative
Monoisotopic Mass:	131.094628656
Theoretical M/Z:	130.08735
Adduct:	[M-H]-

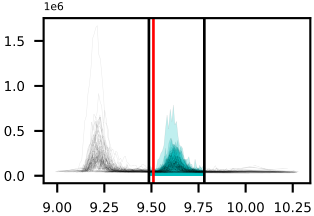

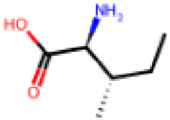

Supplement: Supplementary file 5 [file 41467_2026_76440_MOESM12_ESM.zip › Supplementary Data 10/NEG_msms_mirror_plots/0033_isoleucine_negative_M-H130p0873_9p51.pdf]
