# Supplementary material for: The metabolic and anatomical complexity of root microhabitats modulate their interaction with the microbiota
Source: Nat Commun. 2026 Aug 13;17:9738. doi: 10.1038/s41467-026-76440-4 (PMC13562559; doi:10.1038/s41467-026-76440-4)
Supplement: Supplementary file 5 [file 41467_2026_76440_MOESM12_ESM.zip › Supplementary Data 10/NEG_msms_mirror_plots/0045_melibiose_negative_M-H341p1089_14p62.pdf]

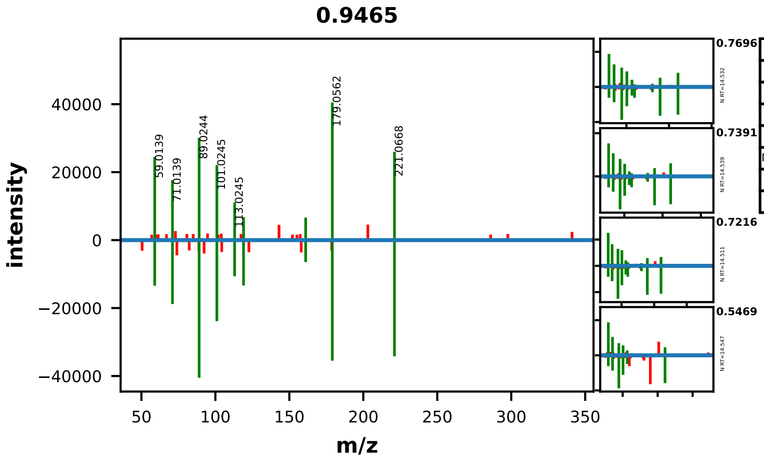

	EMA Compound Info
Name:	melibiose
Label:	
Formula:	C12H22O11
Polarity:	negative
Monoisotopic Mass:	342.116211524
Theoretical M/Z:	341.10894
Adduct:	[M-H]-

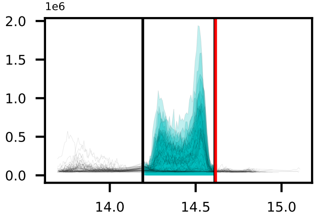

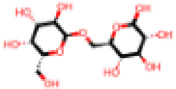

20240411_JGI_GC_509610_DWeedSyn_final_EXP120B_HILICZ_USHXG02558_NEG_MS2_19-UN-22_SP9509-dA289_4_Rg70to1050-CE102040norm-root-S1_Run194.h5

0045_melibiose_negative_M-H341p1089_14p62 [M-H]-
Measured M/Z = 341.1085, 1.2574 ppm difference
Expected Elution of 14.62 minutes, 14.53 min actual

MSMS Scan at 14.534 minutes

Matching M/Zs above 1E-3*max: 59.014, 71.014, 89.024, 101.024, 113.024, 119.035, 161.046, 179.056, 221.067

All Matching M/Zs: 59.014, 71.014, 89.024, 101.024, 113.024, 119.035, 161.046, 179.056, 221.067
