# Supplementary material for: The metabolic and anatomical complexity of root microhabitats modulate their interaction with the microbiota
Source: Nat Commun. 2026 Aug 13;17:9738. doi: 10.1038/s41467-026-76440-4 (PMC13562559; doi:10.1038/s41467-026-76440-4)
Supplement: Supplementary file 5 [file 41467_2026_76440_MOESM12_ESM.zip › Supplementary Data 10/NEG_msms_mirror_plots/0027_cytidine_negative_M-H242p0782_6p79.pdf]

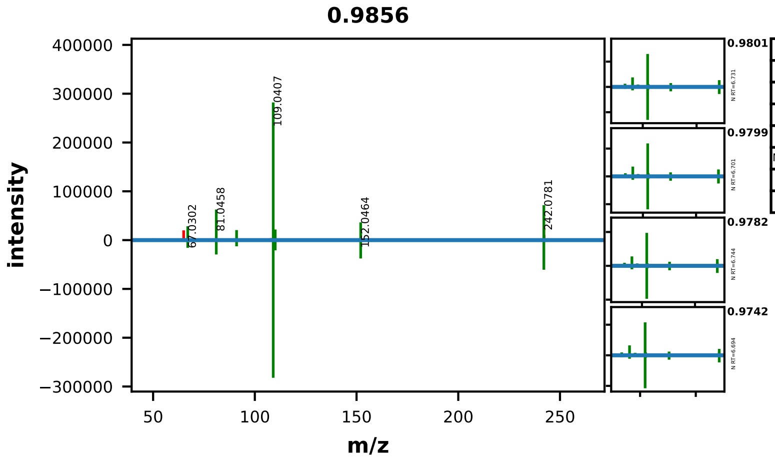

	EMA Compound Info
Name:	cytidine
Label:	
Formula:	C9H13N3O5
Polarity:	negative
Monoisotopic Mass:	243.085520516
Theoretical M/Z:	242.07824
Adduct:	[M-H]-

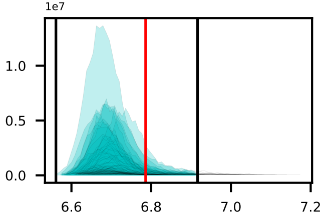

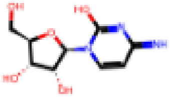

20240411_JGI_GC_509610_DWeedSyn_final_EXP120B_HILICZ_USHXG02558_NEG_MS2_18-UN-21_SP9509-dA289_3_Rg70to1050-CE102040n205060abs-root-S1_Run164.h5

0027_cytidine_negative_M-H242p0782_6p79 [M-H]-
Measured M/Z = 242.0783, 0.2292 ppm difference
Expected Elution of 6.79 minutes, 6.67 min actual

MSMS Scan at 6.689 minutes

Matching M/Zs above 1E-3*max: 67.030, 81.046, 91.030, 95.025, 109.041, 110.036, 152.046, 242.078

All Matching M/Zs: 67.030, 81.046, 91.030, 95.025, 109.041, 110.036, 152.046, 242.078
