# Supplementary material for: The metabolic and anatomical complexity of root microhabitats modulate their interaction with the microbiota
Source: Nat Commun. 2026 Aug 13;17:9738. doi: 10.1038/s41467-026-76440-4 (PMC13562559; doi:10.1038/s41467-026-76440-4)
Supplement: Supplementary file 5 [file 41467_2026_76440_MOESM12_ESM.zip › Supplementary Data 10/NEG_msms_mirror_plots/0048_isocitric_acid_negative_M-H191p0197_16p93.pdf]

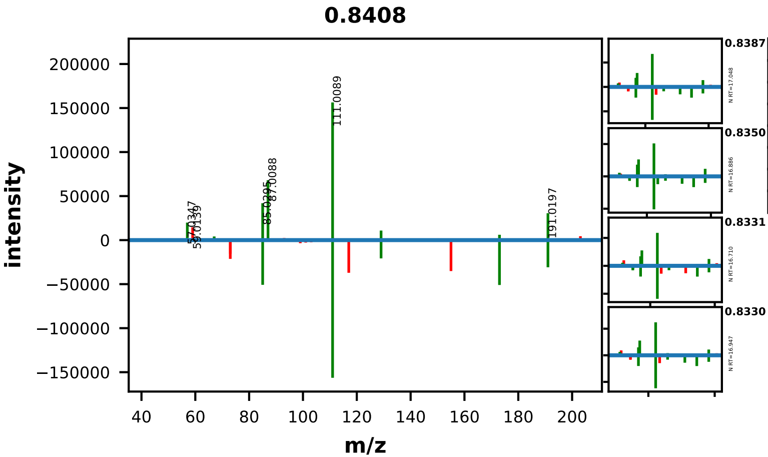

	EMA Compound Info
Name:	isocitric acid
Label:	
Formula:	C6H8O7
Polarity:	negative
Monoisotopic Mass:	192.027002596
Theoretical M/Z:	191.01973
Adduct:	[M-H]-

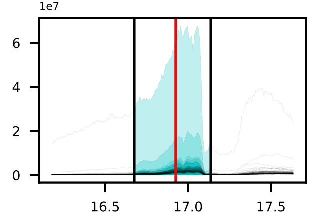

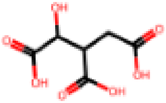

20240411_JGI_GC_509610_DWeedSyn_final_EXP120B_HILICZ_USHXG02558_NEG_MS2_59-UN-71_LJ9250-dA245_3_Rg70to1050-CE102040n205060abs-root-S1_Run158.h5

0048_isocitric_acid_negative_M-H191p0197_16p93 [M-H]-
Measured M/Z = 191.0197, 0.0660 ppm difference
Expected Elution of 16.93 minutes, 17.01 min actual

MSMS Scan at 16.928 minutes

Matching M/Zs above 1E-3*max: 57.035, 67.019, 85.030, 87.009, 111.009, 129.019, 173.009, 191.020

All Matching M/Zs: 57.035, 67.019, 85.030, 87.009, 111.009, 129.019, 173.009, 191.020
