# Supplementary material for: The metabolic and anatomical complexity of root microhabitats modulate their interaction with the microbiota
Source: Nat Commun. 2026 Aug 13;17:9738. doi: 10.1038/s41467-026-76440-4 (PMC13562559; doi:10.1038/s41467-026-76440-4)
Supplement: Supplementary file 5 [file 41467_2026_76440_MOESM12_ESM.zip › Supplementary Data 10/NEG_msms_mirror_plots/0044_gluconic_acid_negative_M-H195p0510_14p23.pdf]

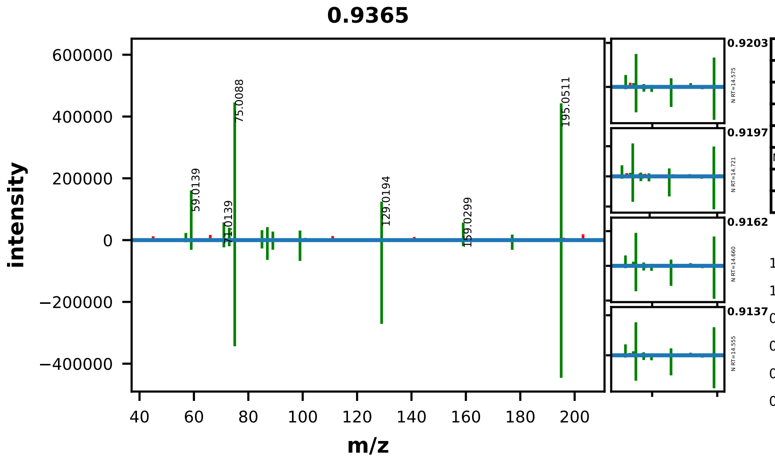

	EMA Compound Info
Name:	gluconic acid
Label:	
Formula:	C6H12O7
Polarity:	negative
Monoisotopic Mass:	196.058302724
Theoretical M/Z:	195.05103
Adduct:	[M-H]-

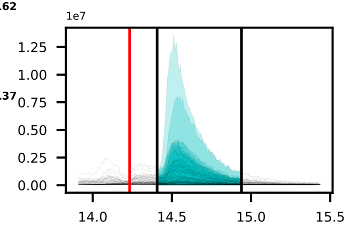

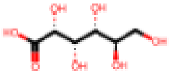

20240411_JGI_GC_509610_DWeedSyn_final_EXP120B_HILICZ_USHXG02558_NEG_MS2_3-UN-3_SP9509-NB_3_Rg70to1050-CE102040n205060abs-root-S1_Run133.h5

0044_gluconic_acid_negative_M-H195p0510_14p23 [M-H]-
Measured M/Z = 195.0510, 0.0997 ppm difference
Expected Elution of 14.23 minutes, 14.51 min actual

MSMS Scan at 14.476 minutes

Matching M/Zs above 1E-3*max: 57.035, 59.014, 71.014, 72.993, 75.009, 83.014, 85.030, 87.009, 89.024, 99.009, 101.024, 129.019, 159.030, 177.041, 195.051

All Matching M/Zs: 57.035, 59.014, 71.014, 72.993, 75.009, 83.014, 85.030, 87.009, 89.024, 99.009, 101.024, 129.019, 159.030, 177.041, 195.051
