# Supplementary material for: The metabolic and anatomical complexity of root microhabitats modulate their interaction with the microbiota
Source: Nat Commun. 2026 Aug 13;17:9738. doi: 10.1038/s41467-026-76440-4 (PMC13562559; doi:10.1038/s41467-026-76440-4)
Supplement: Supplementary file 5 [file 41467_2026_76440_MOESM12_ESM.zip › Supplementary Data 10/NEG_msms_mirror_plots/0047_fumaric_acid_negative_M-H115p0037_16p42.pdf]

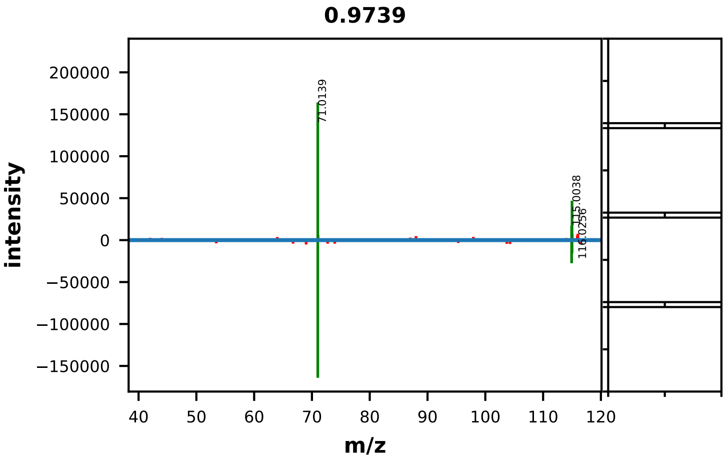

	EMA Compound Info
Name:	fumaric acid
Label:	
Formula:	C4H4O4
Polarity:	negative
Monoisotopic Mass:	116.010958608
Theoretical M/Z:	115.00368
Adduct:	[M-H]-

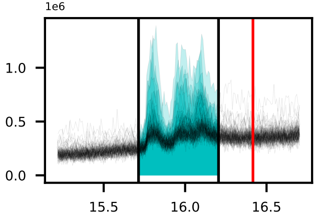

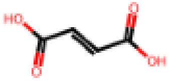

20240411_JGI_GC_509610_DWeedSyn_final_EXP120B_HILICZ_USHXG02558_NEG_MS2_22-UN-26_SP9509-dA245_2_Rg70to1050-CE205060norm-root-S1_Run112.h5

0047_fumaric_acid_negative_M-H115p0037_16p42 [M-H]-
Measured M/Z = 115.0037, 0.2224 ppm difference
Expected Elution of 16.42 minutes, 15.80 min actual

MSMS Scan at 16.072 minutes

Matching M/Zs above 1E-3*max: 71.014, 114.934, 115.004

All Matching M/Zs: 71.014, 114.934, 115.004
