# Supplementary material for: The metabolic and anatomical complexity of root microhabitats modulate their interaction with the microbiota
Source: Nat Commun. 2026 Aug 13;17:9738. doi: 10.1038/s41467-026-76440-4 (PMC13562559; doi:10.1038/s41467-026-76440-4)
Supplement: Supplementary file 5 [file 41467_2026_76440_MOESM12_ESM.zip › Supplementary Data 10/NEG_msms_mirror_plots/0009_2-methylmaleic_acid_negative_M-H129p0193_2p27.pdf]

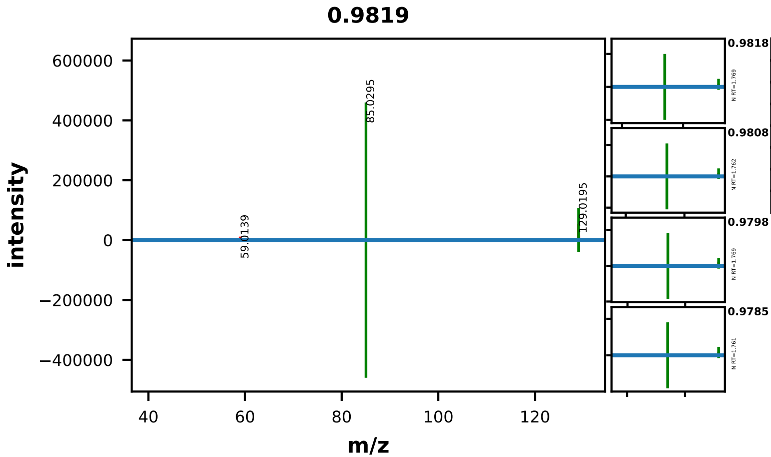

	EMA Compound Info
Name:	2-methylmaleic acid
Label:	
Formula:	C5H6O4
Polarity:	negative
Monoisotopic Mass:	130.026608672
Theoretical M/Z:	129.01933
Adduct:	[M-H]-

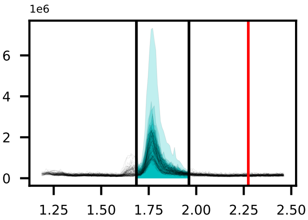

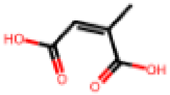

20240411_JGI_GC_509610_DWeedSyn_final_EXP120B_HILICZ_USHXG02558_NEG_MS2_42-UN-49_LJ9250-NB_2_Rg70to1050-CE205060norm-root-S1_Run84.h5

0009_2-methylmaleic_acid_negative_M-H129p0193_2p27 [M-H]-
Measured M/Z = 129.0194, 0.2036 ppm difference
Expected Elution of 2.27 minutes, 1.77 min actual

MSMS Scan at 1.768 minutes

Matching M/Zs above 1E-3*max: 85.029, 129.020

All Matching M/Zs: 85.029, 129.020
