# Supplementary material for: The metabolic and anatomical complexity of root microhabitats modulate their interaction with the microbiota
Source: Nat Commun. 2026 Aug 13;17:9738. doi: 10.1038/s41467-026-76440-4 (PMC13562559; doi:10.1038/s41467-026-76440-4)
Supplement: Supplementary file 5 [file 41467_2026_76440_MOESM12_ESM.zip › Supplementary Data 10/NEG_msms_mirror_plots/0025_guanine_negative_M-H150p0421_6p14.pdf]

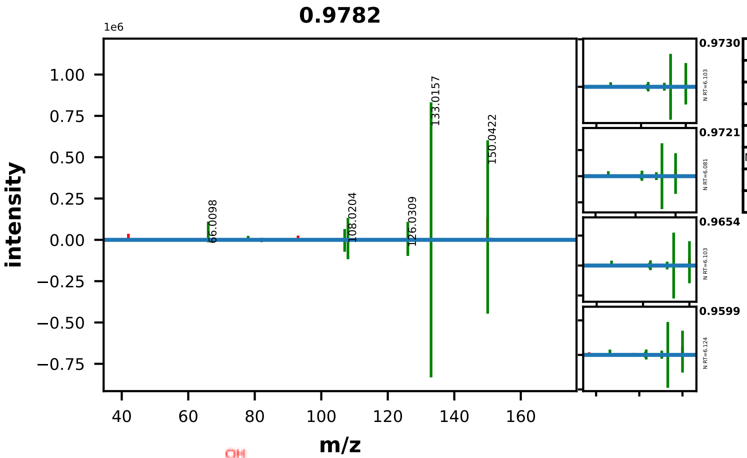

	EMA Compound Info
Name:	guanine
Label:	
Formula:	C5H5N5O
Polarity:	negative
Monoisotopic Mass:	151.04940978
Theoretical M/Z:	150.04213
Adduct:	[M-H]-

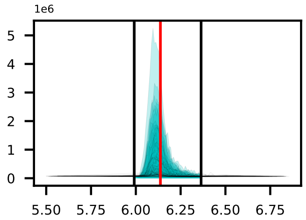

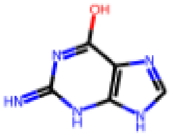

20240411_JGI_GC_509610_DWeedSyn_final_EXP120B_HILICZ_USHXG02558_NEG_MS2_22-UN-26_SP9509-dA245_2_Rg70to1050-CE205060norm-root-S1_Run112.h5

0025_guanine_negative_M-H150p0421_6p14 [M-H]-
Measured M/Z = 150.0421, 0.0273 ppm difference
Expected Elution of 6.14 minutes, 6.10 min actual

MSMS Scan at 6.094 minutes

Matching M/Zs above 1E-3*max: 65.999, 66.010, 78.010, 82.041, 90.010, 105.021, 106.005, 107.036, 108.020, 121.015, 126.031, 132.031, 133.016, 150.042, 151.026

All Matching M/Zs: 65.999, 66.010, 78.010, 82.041, 90.010, 105.021, 106.005, 107.036, 108.020, 121.015, 126.031, 132.031, 133.016, 150.042, 151.026
