# Supplementary material for: The metabolic and anatomical complexity of root microhabitats modulate their interaction with the microbiota
Source: Nat Commun. 2026 Aug 13;17:9738. doi: 10.1038/s41467-026-76440-4 (PMC13562559; doi:10.1038/s41467-026-76440-4)
Supplement: Supplementary file 5 [file 41467_2026_76440_MOESM12_ESM.zip › Supplementary Data 10/NEG_msms_mirror_plots/0039_quinic_acid_negative_M-H191p0561_12p98.pdf]

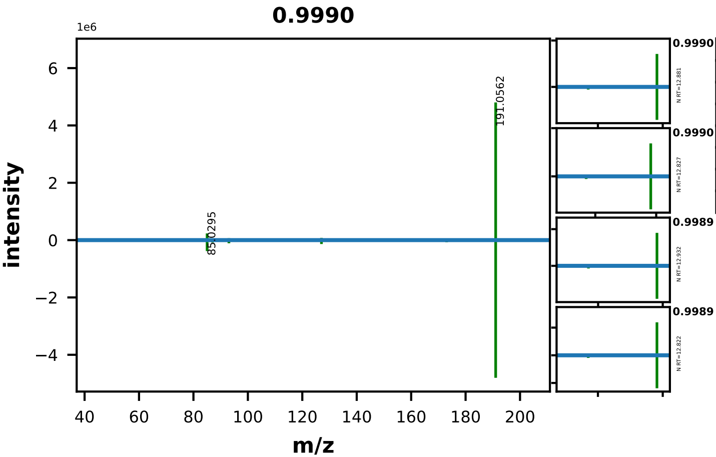


| | EMA Compound Info |
|---|---|
| Name: | quinic acid |
| Label: | |
| Formula: | C7H12O6 |
| Polarity: | negative |
| Monoisotopic Mass: | 192.063388104 |
| Theoretical M/Z: | 191.05611 |
| Adduct: | [M-H]- |

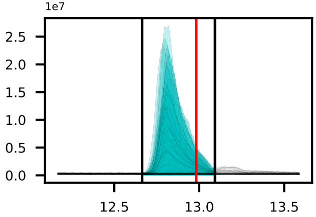


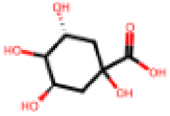


20240411_JGI_GC_509610_DWeedSyn_final_EXP120B_HILICZ_USHXG02558_NEG_MS2_6-UN-7_SP9509-Syncom_1_Rg70to1050-CE102040norm-root-S1_Run66.h5

0039_quinic_acid_negative_M-H191p0561_12p98 [M-H]-
Measured M/Z = 191.0561, 0.0048 ppm difference
Expected Elution of 12.98 minutes, 12.80 min actual

MSMS Scan at 12.806 minutes

Matching M/Zs above 1E-3*max: 59.014, 71.014, 85.029, 87.009, 93.035, 109.029, 111.045, 127.040, 171.029, 173.046, 191.056

All Matching M/Zs: 59.014, 71.014, 85.029, 87.009, 93.035, 109.029, 111.045, 127.040, 171.029, 173.046, 191.056
