# Supplementary material for: The metabolic and anatomical complexity of root microhabitats modulate their interaction with the microbiota
Source: Nat Commun. 2026 Aug 13;17:9738. doi: 10.1038/s41467-026-76440-4 (PMC13562559; doi:10.1038/s41467-026-76440-4)
Supplement: Supplementary file 5 [file 41467_2026_76440_MOESM12_ESM.zip › Supplementary Data 10/NEG_msms_mirror_plots/0034_tryptophan_negative_M-H203p0826_9p96.pdf]

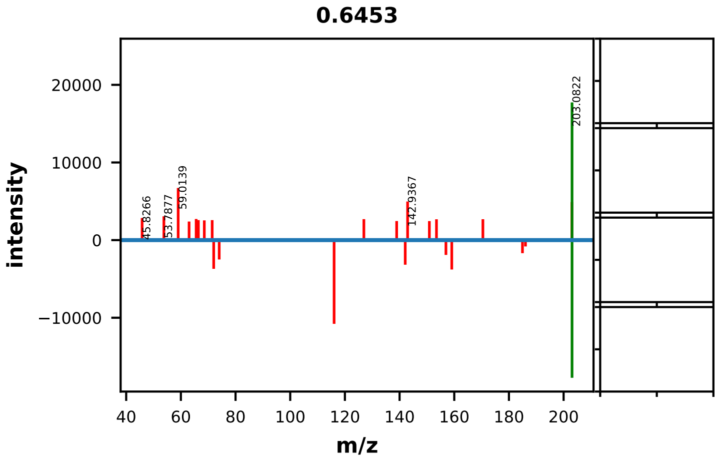

	EMA Compound Info
Name:	tryptophan
Label:	
Formula:	C11H12N2O2
Polarity:	negative
Monoisotopic Mass:	204.089877624
Theoretical M/Z:	203.0826
Adduct:	[M-H]-

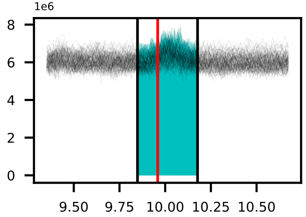

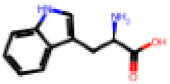

20240411_JGI_GC_509610_DWeedSyn_final_EXP120B_HILICZ_USHXG02558_NEG_MS2_41-UN-48_SP9509-NB_1_Rg70to1050-CE102040norm-root-S1_Run44.h5

0034_tryptophan_negative_M-H203p0826_9p96 [M-H]-
Measured M/Z = 203.0821, 2.3389 ppm difference
Expected Elution of 9.96 minutes, 10.05 min actual

MSMS Scan at 9.938 minutes

Matching M/Zs above 1E-3*max: 203.082

All Matching M/Zs: 203.082
