# Supplementary material for: The metabolic and anatomical complexity of root microhabitats modulate their interaction with the microbiota
Source: Nat Commun. 2026 Aug 13;17:9738. doi: 10.1038/s41467-026-76440-4 (PMC13562559; doi:10.1038/s41467-026-76440-4)
Supplement: Supplementary file 5 [file 41467_2026_76440_MOESM12_ESM.zip › Supplementary Data 10/NEG_msms_mirror_plots/0040_myo-inositol_negative_M-H179p0561_13p18.pdf]

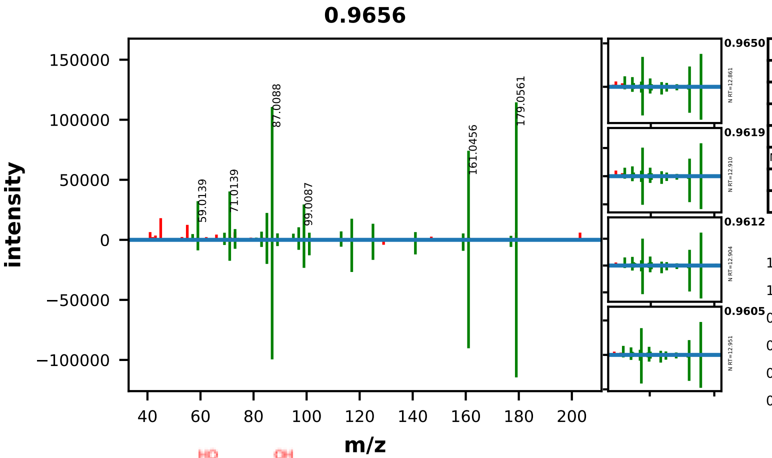

	EMA Compound Info
Name:	myo-inositol
Label:	
Formula:	C6H12O6
Polarity:	negative
Monoisotopic Mass:	180.063388104
Theoretical M/Z:	179.05611
Adduct:	[M-H]-

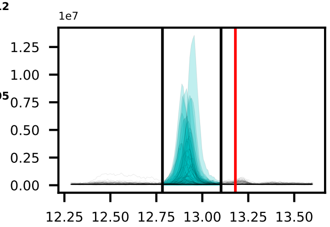

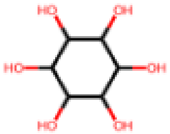

20240411_JGI_GC_509610_DWeedSyn_final_EXP120B_HILICZ_USHXG02558_NEG_MS2_13-UN-15_SP9509-dP65_3_Rg70to1050-CE102040n205060abs-root-S1_Run130.h5

0040_myo-inositol_negative_M-H179p0561_13p18 [M-H]-
Measured M/Z = 179.0561, 0.1299 ppm difference
Expected Elution of 13.18 minutes, 12.90 min actual

MSMS Scan at 12.876 minutes

Matching M/Zs above 1E-3*max: 57.035, 59.014, 69.035, 71.014, 73.029, 81.034, 83.014, 85.029, 87.009, 89.025, 95.014, 97.030, 99.009, 101.024, 113.024, 117.020, 125.025, 141.019, 159.030, 161.046, 177.041, 179.056

All Matching M/Zs: 57.035, 59.014, 69.035, 71.014, 73.029, 81.034, 83.014, 85.029, 87.009, 89.025, 95.014, 97.030, 99.009, 101.024, 113.024, 117.020, 125.025, 141.019, 159.030, 161.046, 177.041, 179.056
