# Supplementary material for: The metabolic and anatomical complexity of root microhabitats modulate their interaction with the microbiota
Source: Nat Commun. 2026 Aug 13;17:9738. doi: 10.1038/s41467-026-76440-4 (PMC13562559; doi:10.1038/s41467-026-76440-4)
Supplement: Supplementary file 5 [file 41467_2026_76440_MOESM12_ESM.zip › Supplementary Data 10/NEG_msms_mirror_plots/0029_pantothenic_acid_negative_M-H218p1034_6p81.pdf]

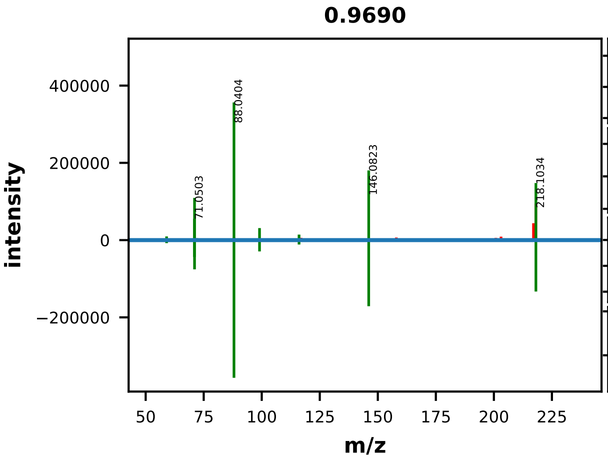

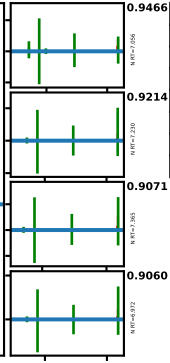

	EMA Compound Info
Name:	pantothenic acid
Label:	
Formula:	C9H17NO5
Polarity:	negative
Monoisotopic Mass:	219.110672644
Theoretical M/Z:	218.1034
Adduct:	[M-H]-

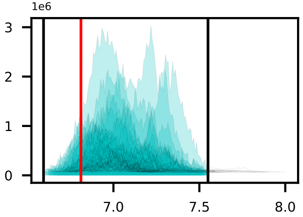

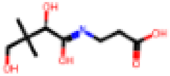

20240411_JGI_GC_509610_DWeedSyn_final_EXP120B_HILICZ_USHXG02558_NEG_MS2_17-UN-20_SP9509-dA289_2_Rg70to1050-CE205060norm-root-S1_Run93.h5

0029_pantothenic_acid_negative_M-H218p1034_6p81 [M-H]-
Measured M/Z = 218.1033, 0.2440 ppm difference
Expected Elution of 6.81 minutes, 6.93 min actual

MSMS Scan at 6.962 minutes

Matching M/Zs above 1E-3*max: 59.014, 71.014, 71.050, 72.009, 88.040, 99.045, 116.035, 116.072, 129.056, 146.082, 218.103

All Matching M/Zs: 59.014, 71.014, 71.050, 72.009, 88.040, 99.045, 116.035, 116.072, 129.056, 146.082, 218.103
