# Supplementary material for: The metabolic and anatomical complexity of root microhabitats modulate their interaction with the microbiota
Source: Nat Commun. 2026 Aug 13;17:9738. doi: 10.1038/s41467-026-76440-4 (PMC13562559; doi:10.1038/s41467-026-76440-4)
Supplement: Supplementary file 5 [file 41467_2026_76440_MOESM12_ESM.zip › Supplementary Data 10/NEG_msms_mirror_plots/0021_malonic_acid_negative_M-H103p0037_4p82.pdf]

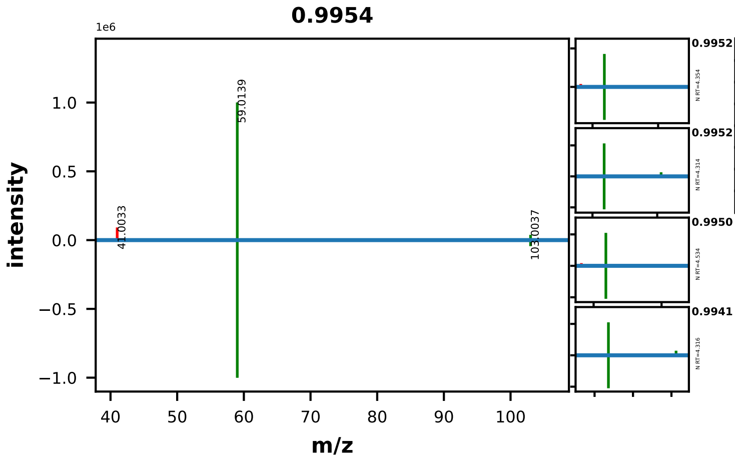

	EMA Compound Info
Name:	malonic acid
Label:	
Formula:	C3H4O4
Polarity:	negative
Monoisotopic Mass:	104.010958608
Theoretical M/Z:	103.00368
Adduct:	[M-H]-

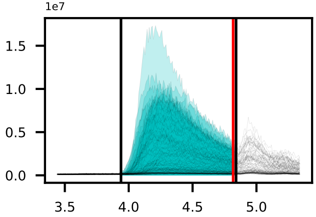

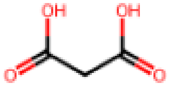

20240411_JGI_GC_509610_DWeedSyn_final_EXP120B_HILICZ_USHXG02558_NEG_MS2_3-UN-3_SP9509-NB_3_Rg70to1050-CE102040n205060abs-root-S1_Run133.h5

0021_malonic_acid_negative_M-H103p0037_4p82 [M-H]-
Measured M/Z = 103.0037, 0.2353 ppm difference
Expected Elution of 4.82 minutes, 4.21 min actual

MSMS Scan at 4.402 minutes

Matching M/Zs above 1E-3*max: 59.014, 103.004

All Matching M/Zs: 59.014, 103.004
