# Supplementary material for: The metabolic and anatomical complexity of root microhabitats modulate their interaction with the microbiota
Source: Nat Commun. 2026 Aug 13;17:9738. doi: 10.1038/s41467-026-76440-4 (PMC13562559; doi:10.1038/s41467-026-76440-4)
Supplement: Supplementary file 5 [file 41467_2026_76440_MOESM12_ESM.zip › Supplementary Data 10/NEG_msms_mirror_plots/0038_5-oxo-proline_negative_M-H128p0353_11p46.pdf]

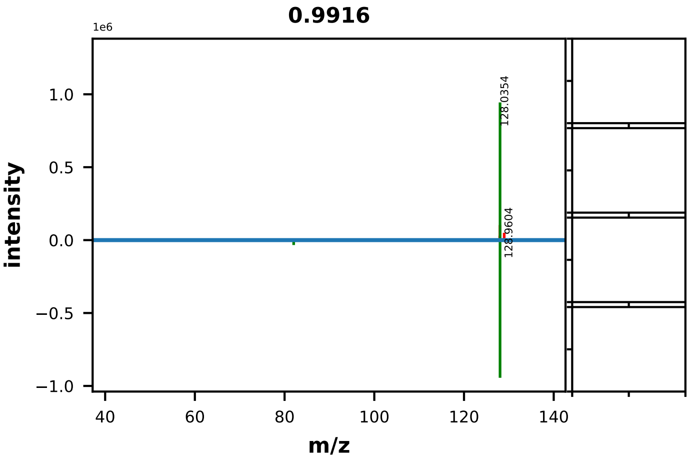

	EMA Compound Info
Name:	5-oxo-proline
Label:	
Formula:	C5H7NO3
Polarity:	negative
Monoisotopic Mass:	129.042593084
Theoretical M/Z:	128.03532
Adduct:	[M-H]-

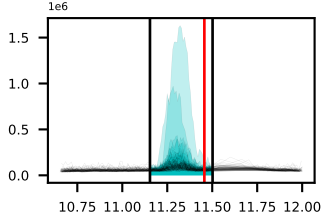

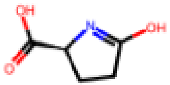

20240411_JGI_GC_509610_DWeedSyn_final_EXP120B_HILICZ_USHXG02558_NEG_MS2_1-UN-1_SP9509-NB_1_Rg70to1050-CE102040norm-root-S1_Run69.h5

0038_5-oxo-proline_negative_M-H128p0353_11p46 [M-H]-
Measured M/Z = 128.0353, 0.0456 ppm difference
Expected Elution of 11.46 minutes, 11.32 min actual

MSMS Scan at 11.295 minutes

Matching M/Zs above 1E-3*max: 82.030, 128.035

All Matching M/Zs: 82.030, 128.035
