# Supplementary material for: The metabolic and anatomical complexity of root microhabitats modulate their interaction with the microbiota
Source: Nat Commun. 2026 Aug 13;17:9738. doi: 10.1038/s41467-026-76440-4 (PMC13562559; doi:10.1038/s41467-026-76440-4)
Supplement: Supplementary file 5 [file 41467_2026_76440_MOESM12_ESM.zip › Supplementary Data 10/NEG_msms_mirror_plots/0023_glyceraldehyde_negative_M-H89p0244_4p96.pdf]

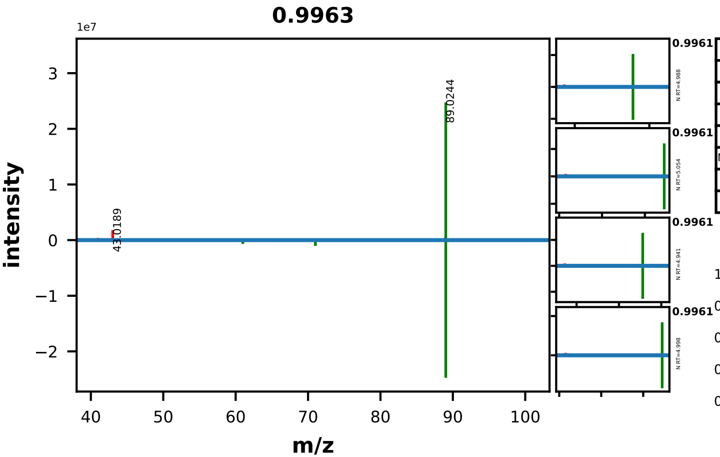

	EMA Compound Info
Name:	glyceraldehyde
Label:	
Formula:	C3H6O3
Polarity:	negative
Monoisotopic Mass:	90.031694052
Theoretical M/Z:	89.02442
Adduct:	[M-H]-

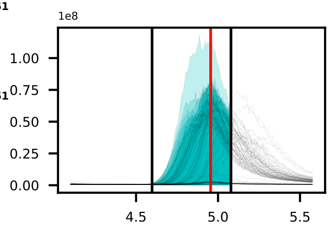

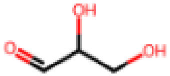

20240411_JGI_GC_509610_DWeedSyn_final_EXP120B_HILICZ_USHXG02558_NEG_MS2_19-UN-22_SP9509-dA289_4_Rg70to1050-CE102040norm-root-S1_Run194.h5

0023_glyceraldehyde_negative_M-H89p0244_4p96 [M-H]-
Measured M/Z = 89.0244, 0.0624 ppm difference
Expected Elution of 4.96 minutes, 4.95 min actual

MSMS Scan at 4.933 minutes

Matching M/Zs above 1E-3*max: 60.993, 71.014, 87.009, 88.988, 89.024

All Matching M/Zs: 60.993, 71.014, 87.009, 88.988, 89.024
