# Supplementary material for: The metabolic and anatomical complexity of root microhabitats modulate their interaction with the microbiota
Source: Nat Commun. 2026 Aug 13;17:9738. doi: 10.1038/s41467-026-76440-4 (PMC13562559; doi:10.1038/s41467-026-76440-4)
Supplement: Supplementary file 5 [file 41467_2026_76440_MOESM12_ESM.zip › Supplementary Data 10/POS_msms_mirror_plots/0031_carnitine_positive_M162p1125_13p10.pdf]

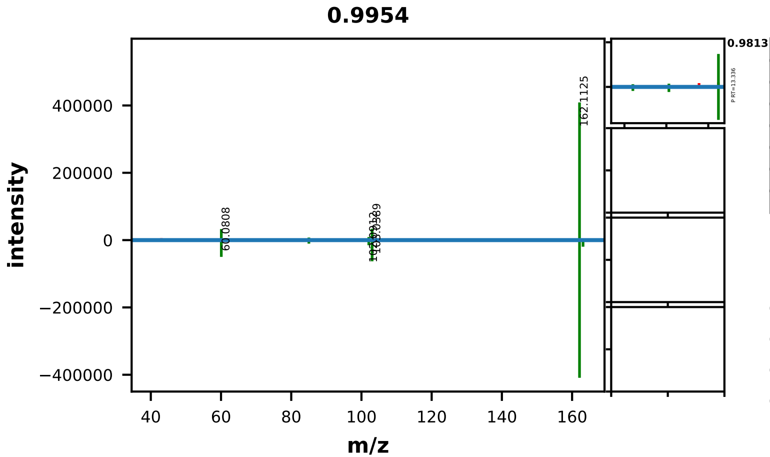

	EMA Compound Info
Name:	carnitine
Label:	
Formula:	C7H16NO3+
Polarity:	positive
Monoisotopic Mass:	162.112469792
Theoretical M/Z:	162.11247
Adduct:	[M]+

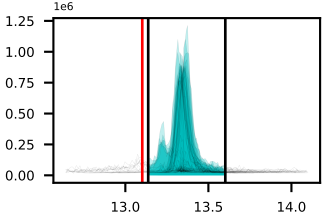

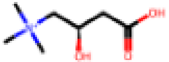

20240411_JGI_GC_509610_DWeedSyn_final_EXP120B_HILICZ_USHXG02558_POS_MS2_0_ISTD_Post_Rg70to1050-CE102040norm--ISTD_Run258.h5

0031_carnitine_positive_M162p1125_13p10 [M]+
Measured M/Z = 162.1125, 0.0956 ppm difference
Expected Elution of 13.10 minutes, 13.37 min actual

MSMS Scan at 13.365 minutes

Matching M/Zs above 1E-3*max: 53.014, 60.081, 85.028, 102.091, 103.039, 162.112, 163.116

All Matching M/Zs: 53.014, 60.081, 85.028, 102.091, 103.039, 162.112, 163.116
