# Supplementary material for: The metabolic and anatomical complexity of root microhabitats modulate their interaction with the microbiota
Source: Nat Commun. 2026 Aug 13;17:9738. doi: 10.1038/s41467-026-76440-4 (PMC13562559; doi:10.1038/s41467-026-76440-4)
Supplement: Supplementary file 5 [file 41467_2026_76440_MOESM12_ESM.zip › Supplementary Data 10/POS_msms_mirror_plots/0023_bis_3-aminopropyl_amine_positive_M-H+2Na176p1134_8p80.pdf]

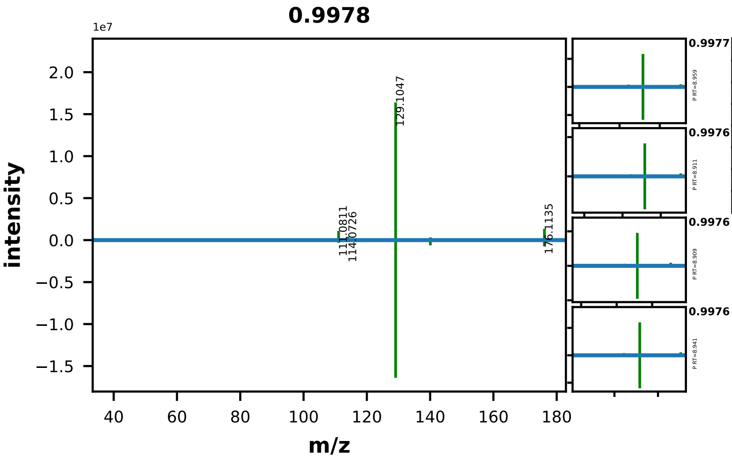

	EMA Compound Info
Name:	bis(3-aminopropyl)amine
Label:	
Formula:	C6H17N3
Polarity:	positive
Monoisotopic Mass:	131.142247544
Theoretical M/Z:	176.11341
Adduct:	[M-H+2Na]+

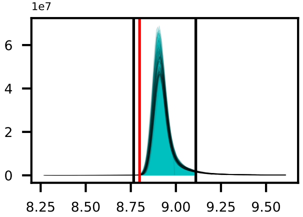

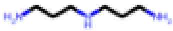

20240411_JGI_GC_509610_DWeedSyn_final_EXP120B_HILICZ_USHXG02558_POS_MS2_46-UN-54_LJ9250-Syncom_2_Rg70to1050-CE205060norm-root-S1_Run114.h5

0023_bis_3-aminopropyl_amine_positive_M-H+2Na176p1134_8p80 [M-H+2Na
Measured M/Z = 176.1135, 0.4158 ppm difference
Expected Elution of 8.80 minutes, 8.91 min actual

MSMS Scan at 8.899 minutes

Matching M/Zs above 1E-3*max: 100.093, 111.081, 114.073, 129.105, 140.079, 158.090, 176.114

All Matching M/Zs: 100.093, 111.081, 114.073, 129.105, 140.079, 158.090, 176.114
