# Supplementary material for: The metabolic and anatomical complexity of root microhabitats modulate their interaction with the microbiota
Source: Nat Commun. 2026 Aug 13;17:9738. doi: 10.1038/s41467-026-76440-4 (PMC13562559; doi:10.1038/s41467-026-76440-4)
Supplement: Supplementary file 5 [file 41467_2026_76440_MOESM12_ESM.zip › Supplementary Data 10/POS_msms_mirror_plots/0020_guanosine_positive_M+H284p0989_8p42.pdf]

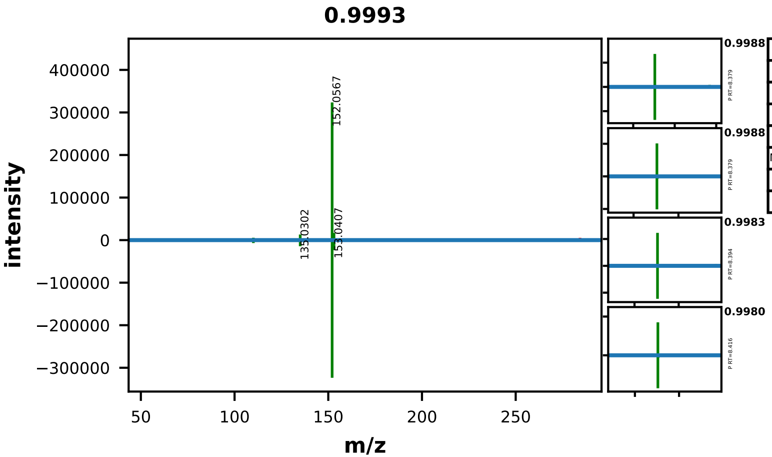

	EMA Compound Info
Name:	guanosine
Label:	
Formula:	C10H13N5O5
Polarity:	positive
Monoisotopic Mass:	283.091668516
Theoretical M/Z:	284.09894
Adduct:	[M+H]+

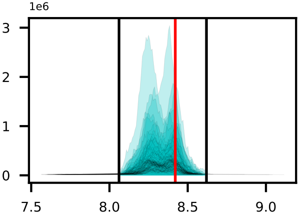

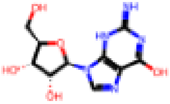

20240411_JGI_GC_509610_DWeedSyn_final_EXP120B_HILICZ_USHXG02558_POS_MS2_27-UN-32_SP9509-mP65_2_Rg70to1050-CE205060norm-root-S1_Run117.h5

0020_guanosine_positive_M+H284p0989_8p42 [M+H]+
Measured M/Z = 284.0989, 0.1401 ppm difference
Expected Elution of 8.42 minutes, 8.41 min actual

MSMS Scan at 8.426 minutes

Matching M/Zs above 1E-3*max: 109.051, 110.035, 128.046, 135.030, 152.057, 153.041

All Matching M/Zs: 109.051, 110.035, 128.046, 135.030, 152.057, 153.041
