# Supplementary material for: The metabolic and anatomical complexity of root microhabitats modulate their interaction with the microbiota
Source: Nat Commun. 2026 Aug 13;17:9738. doi: 10.1038/s41467-026-76440-4 (PMC13562559; doi:10.1038/s41467-026-76440-4)
Supplement: Supplementary file 5 [file 41467_2026_76440_MOESM12_ESM.zip › Supplementary Data 10/POS_msms_mirror_plots/0035_4-aminobutanoic_acid_positive_M+H104p0706_14p21.pdf]

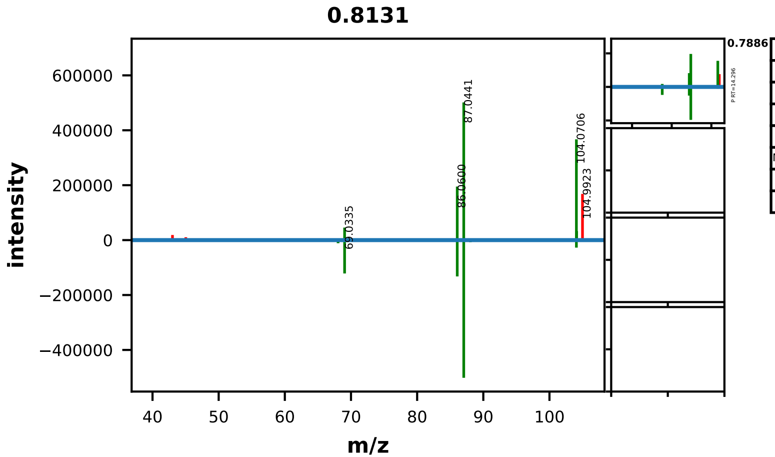

	EMA Compound Info
Name:	4-aminobutyric acid
Label:	
Formula:	C4H9NO2
Polarity:	positive
Monoisotopic Mass:	103.063328528
Theoretical M/Z:	104.0706
Adduct:	[M+H]+

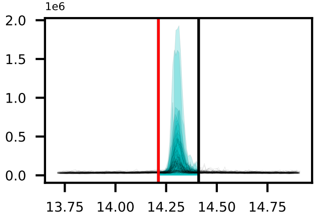

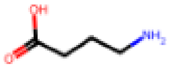

20240411_JGI_GC_509610_DWeedSyn_final_EXP120B_HILICZ_USHXG02558_POS_MS2_14-UN-16_SP9509-dP65_4_Rg70to1050-CE102040norm-root-S1_Run187.h5

0035_4-aminobutanoic_acid_positive_M+H104p0706_14p21 [M+H]+
Measured M/Z = 104.0705, 0.6458 ppm difference
Expected Elution of 14.21 minutes, 14.29 min actual

MSMS Scan at 14.295 minutes

Matching M/Zs above 1E-3*max: 68.050, 69.034, 86.060, 87.044, 88.047, 104.071, 104.107

All Matching M/Zs: 68.050, 69.034, 86.060, 87.044, 88.047, 104.071, 104.107
