# Supplementary material for: The metabolic and anatomical complexity of root microhabitats modulate their interaction with the microbiota
Source: Nat Commun. 2026 Aug 13;17:9738. doi: 10.1038/s41467-026-76440-4 (PMC13562559; doi:10.1038/s41467-026-76440-4)
Supplement: Supplementary file 5 [file 41467_2026_76440_MOESM12_ESM.zip › Supplementary Data 10/POS_msms_mirror_plots/0029_proline_positive_M+H116p0706_10p72.pdf]

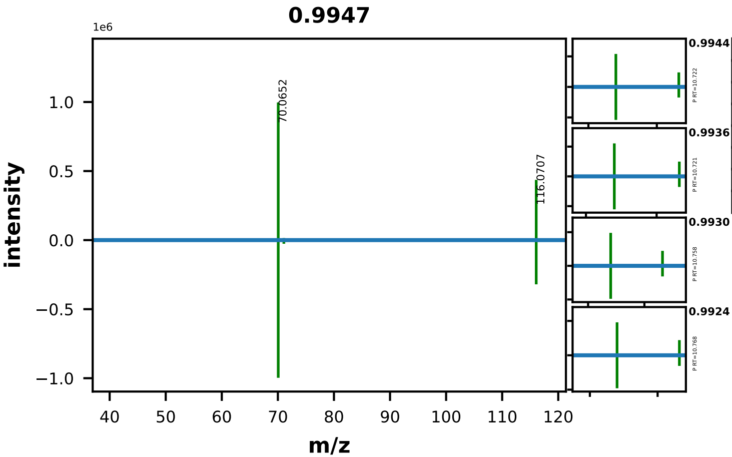

	EMA Compound Info
Name:	proline
Label:	
Formula:	C5H9NO2
Polarity:	positive
Monoisotopic Mass:	115.063328528
Theoretical M/Z:	116.0706
Adduct:	[M+H]+

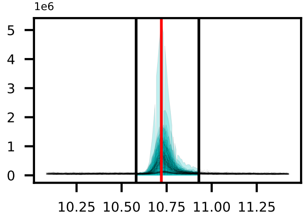

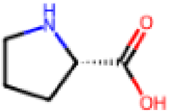

20240411_JGI_GC_509610_DWeedSyn_final_EXP120B_HILICZ_USHXG02558_POS_MS2_27-UN-32_SP9509-mP65_2_Rg70to1050-CE205060norm-root-S1_Run117.h5

0029_proline_positive_M+H116p0706_10p72 [M+H]+
Measured M/Z = 116.0706, 0.4011 ppm difference
Expected Elution of 10.72 minutes, 10.75 min actual

MSMS Scan at 10.744 minutes

Matching M/Zs above 1E-3*max: 70.065, 71.062, 71.068, 116.071, 117.074

All Matching M/Zs: 70.065, 71.062, 71.068, 116.071, 117.074
