# Supplementary material for: The metabolic and anatomical complexity of root microhabitats modulate their interaction with the microbiota
Source: Nat Commun. 2026 Aug 13;17:9738. doi: 10.1038/s41467-026-76440-4 (PMC13562559; doi:10.1038/s41467-026-76440-4)
Supplement: Supplementary file 5 [file 41467_2026_76440_MOESM12_ESM.zip › Supplementary Data 10/POS_msms_mirror_plots/0011_riboflavin_positive_M+H377p1456_4p49.pdf]

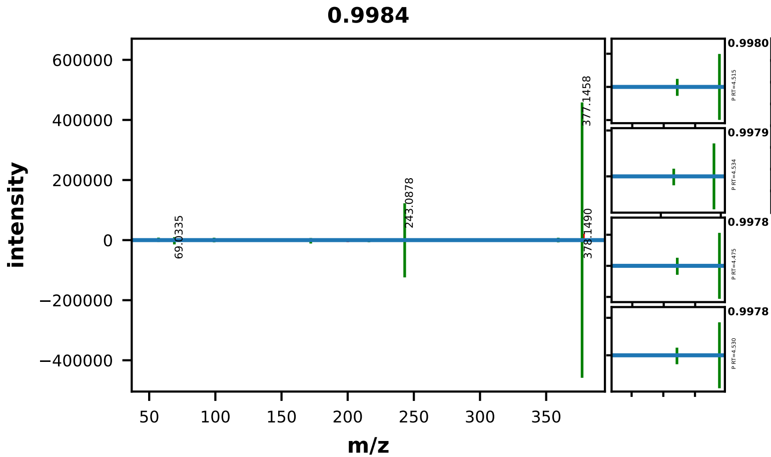

	EMA Compound Info
Name:	riboflavin
Label:	
Formula:	C17H20N4O6
Polarity:	positive
Monoisotopic Mass:	376.13828436
Theoretical M/Z:	377.14556
Adduct:	[M+H]+

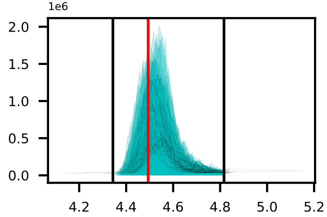

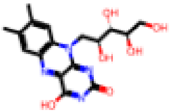

20240411_JGI_GC_509610_DWeedSyn_final_EXP120B_HILICZ_USHXG02558_POS_MS2_21-UN-25_SP9509-dA245_1_Rg70to1050-CE102040norm-root-S1_Run55.h5

0011_riboflavin_positive_M+H377p1456_4p49 [M+H]+
Measured M/Z = 377.1455, 0.0896 ppm difference
Expected Elution of 4.49 minutes, 4.55 min actual

MSMS Scan at 4.518 minutes

Matching M/Zs above 1E-3*max: 57.034, 61.028, 69.034, 71.013, 71.049, 75.044, 81.034, 99.044, 117.055, 172.087, 216.077, 243.088, 244.092, 359.135, 377.146

All Matching M/Zs: 57.034, 61.028, 69.034, 71.013, 71.049, 75.044, 81.034, 99.044, 117.055, 172.087, 216.077, 243.088, 244.092, 359.135, 377.146
