# Supplementary material for: The metabolic and anatomical complexity of root microhabitats modulate their interaction with the microbiota
Source: Nat Commun. 2026 Aug 13;17:9738. doi: 10.1038/s41467-026-76440-4 (PMC13562559; doi:10.1038/s41467-026-76440-4)
Supplement: Supplementary file 5 [file 41467_2026_76440_MOESM12_ESM.zip › Supplementary Data 10/POS_msms_mirror_plots/0037_melibiose_positive_M+NH4360p1500_14p61.pdf]

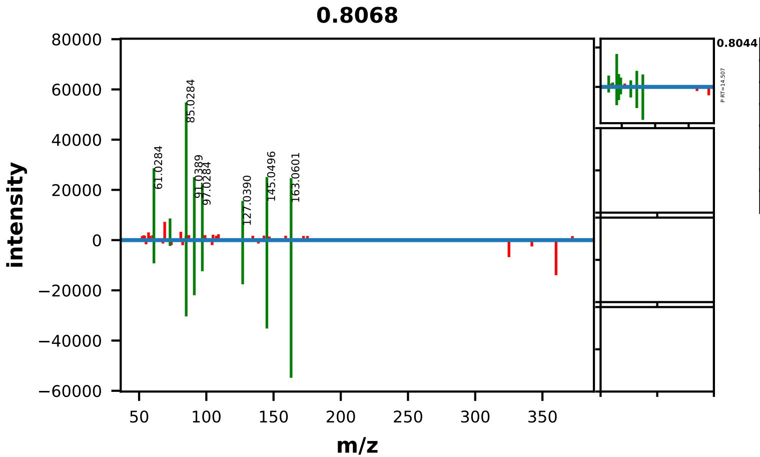

EMA Compound Info	
Name:	melibiose
Label:	
Formula:	C12H22O11
Polarity:	positive
Monoisotopic Mass:	342.116211524
Theoretical M/Z:	360.15004
Adduct:	[M+NH4]+

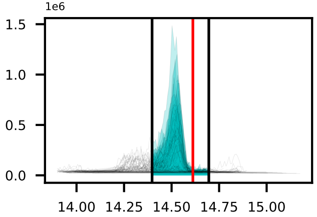

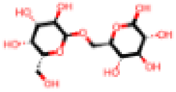

20240411_JGI_GC_509610_DWeedSyn_final_EXP120B_HILICZ_USHXG02558_POS_MS2_15-UN-17_SP9509-dP65_5_Rg70to1050-CE205060norm-root-S1_Run236.h5

0037_melibiose_positive_M+NH4360p1500_14p61 [M+NH4]+
Measured M/Z = 360.1500, 0.0573 ppm difference
Expected Elution of 14.61 minutes, 14.53 min actual

MSMS Scan at 14.532 minutes

Matching M/Zs above 1E-3*max: 61.028, 73.028, 85.028, 91.039, 97.028, 127.039, 145.050, 163.060

All Matching M/Zs: 61.028, 73.028, 85.028, 91.039, 97.028, 127.039, 145.050, 163.060
