# Supplementary material for: The metabolic and anatomical complexity of root microhabitats modulate their interaction with the microbiota
Source: Nat Commun. 2026 Aug 13;17:9738. doi: 10.1038/s41467-026-76440-4 (PMC13562559; doi:10.1038/s41467-026-76440-4)
Supplement: Supplementary file 5 [file 41467_2026_76440_MOESM12_ESM.zip › Supplementary Data 10/POS_msms_mirror_plots/0017_cytidine_positive_M+H244p0928_6p80.pdf]

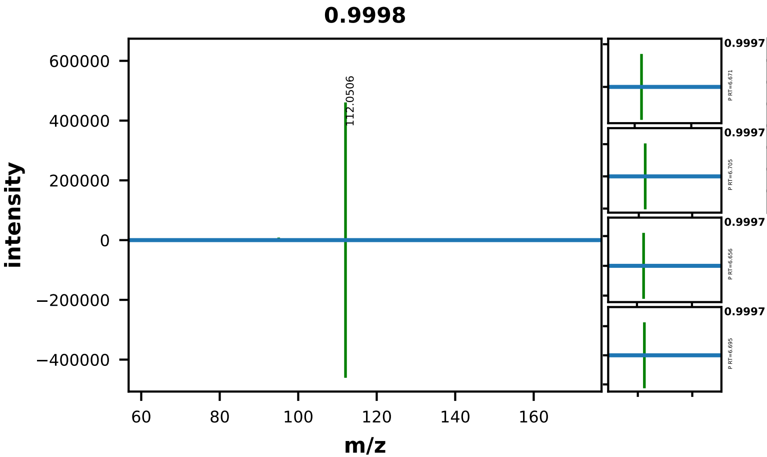

	EMA Compound Info
Name:	cytidine
Label:	
Formula:	C9H13N3O5
Polarity:	positive
Monoisotopic Mass:	243.085520516
Theoretical M/Z:	244.0928
Adduct:	[M+H]+

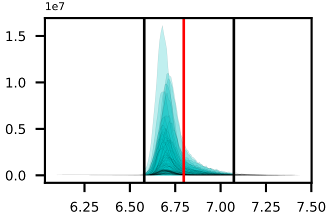

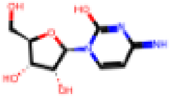

20240411_JGI_GC_509610_DWeedSyn_final_EXP120B_HILICZ_USHXG02558_POS_MS2_54-UN-64_LJ9250-dA289_2_Rg70to1050-CE205060norm-root-S1_Run107.h5

0017_cytidine_positive_M+H244p0928_6p80 [M+H]+
Measured M/Z = 244.0927, 0.3162 ppm difference
Expected Elution of 6.80 minutes, 6.70 min actual

MSMS Scan at 6.669 minutes

Matching M/Zs above 1E-3*max: 95.024, 112.051

All Matching M/Zs: 95.024, 112.051
