# Supplementary material for: The metabolic and anatomical complexity of root microhabitats modulate their interaction with the microbiota
Source: Nat Commun. 2026 Aug 13;17:9738. doi: 10.1038/s41467-026-76440-4 (PMC13562559; doi:10.1038/s41467-026-76440-4)
Supplement: Supplementary file 5 [file 41467_2026_76440_MOESM12_ESM.zip › Supplementary Data 10/POS_msms_mirror_plots/0015_2deoxyguanosine_positive_M+H268p1040_6p74.pdf]

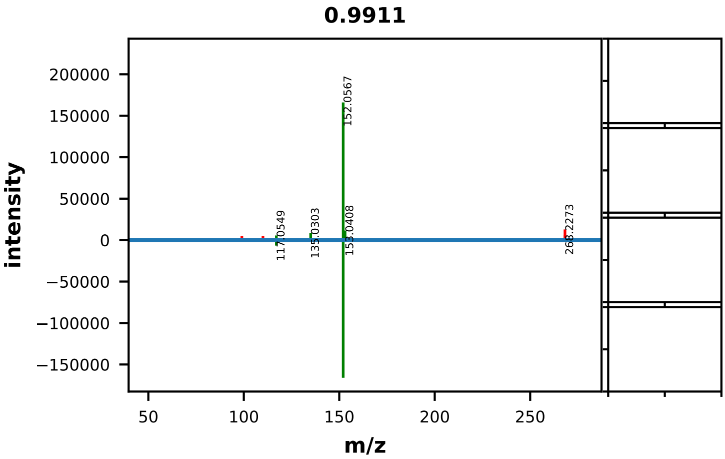

	EMA Compound Info
Name:	2'-deoxyguanosine
Label:	
Formula:	C10H13N5O4
Polarity:	positive
Monoisotopic Mass:	267.096753896
Theoretical M/Z:	268.10403
Adduct:	[M+H]+

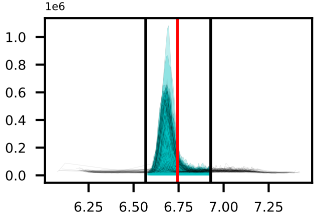

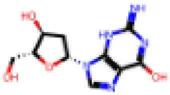

20240411_JGI_GC_509610_DWeedSyn_final_EXP120B_HILICZ_USHXG02558_POS_MS2_54-UN-64_LJ9250-dA289_2_Rg70to1050-CE205060norm-root-S1_Run107.h5

0015_2deoxyguanosine_positive_M+H268p1040_6p74 [M+H]+
Measured M/Z = 268.1039, 0.3897 ppm difference
Expected Elution of 6.74 minutes, 6.70 min actual

MSMS Scan at 6.700 minutes

Matching M/Zs above 1E-3*max: 73.028, 117.055, 135.030, 152.057, 153.041

All Matching M/Zs: 73.028, 117.055, 135.030, 152.057, 153.041
