# Supplementary material for: The metabolic and anatomical complexity of root microhabitats modulate their interaction with the microbiota
Source: Nat Commun. 2026 Aug 13;17:9738. doi: 10.1038/s41467-026-76440-4 (PMC13562559; doi:10.1038/s41467-026-76440-4)
Supplement: Supplementary file 5 [file 41467_2026_76440_MOESM12_ESM.zip › Supplementary Data 10/POS_msms_mirror_plots/0007_hypoxanthine_positive_M+H137p0458_3p09.pdf]

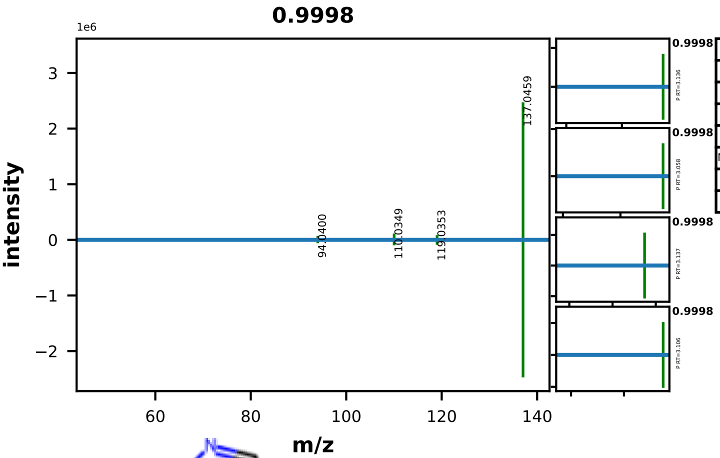

	EMA Compound Info
Name:	hypoxanthine
Label:	
Formula:	C5H4N4O
Polarity:	positive
Monoisotopic Mass:	136.038510748
Theoretical M/Z:	137.04579
Adduct:	[M+H]+

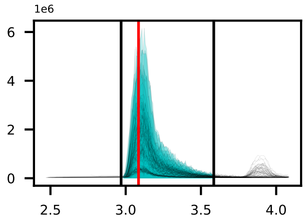

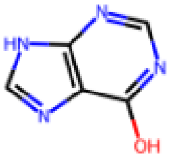

20240411_JGI_GC_509610_DWeedSyn_final_EXP120B_HILICZ_USHXG02558_POS_MS2_40-UN-47_SP9509-mA245_5_Rg70to1050-CE205060norm-root-S1_Run245.h5

0007_hypoxanthine_positive_M+H137p0458_3p09 [M+H]+
Measured M/Z = 137.0458, 0.0793 ppm difference
Expected Elution of 3.09 minutes, 3.09 min actual

MSMS Scan at 3.119 minutes

Matching M/Zs above 1E-3*max: 55.029, 82.040, 94.040, 110.035, 112.051, 119.035, 137.046

All Matching M/Zs: 55.029, 67.029, 82.040, 94.040, 110.035, 112.051, 119.035, 137.046
