# Supplementary material for: The metabolic and anatomical complexity of root microhabitats modulate their interaction with the microbiota
Source: Nat Commun. 2026 Aug 13;17:9738. doi: 10.1038/s41467-026-76440-4 (PMC13562559; doi:10.1038/s41467-026-76440-4)
Supplement: Supplementary file 5 [file 41467_2026_76440_MOESM12_ESM.zip › Supplementary Data 10/POS_msms_mirror_plots/0021_trigonelline_positive_M138p0549_8p59.pdf]

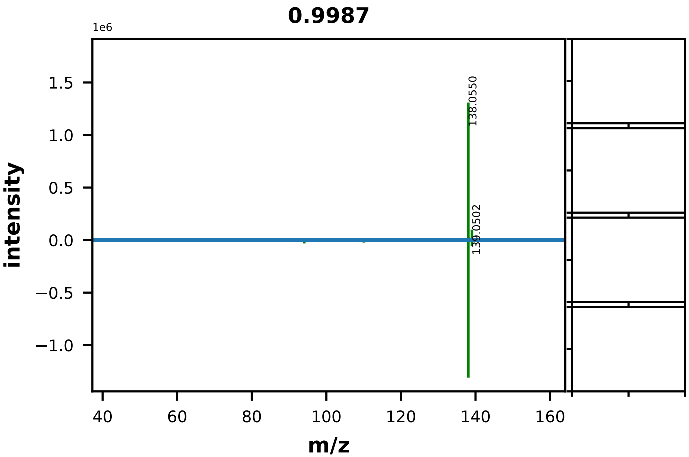

	EMA Compound Info
Name:	trigonelline
Label:	
Formula:	C7H8NO2+
Polarity:	positive
Monoisotopic Mass:	138.054954916
Theoretical M/Z:	138.05495
Adduct:	[M]+

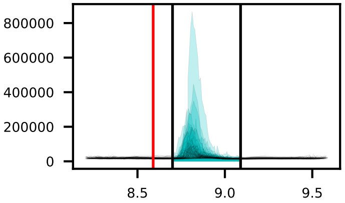

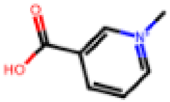

20240411_JGI_GC_509610_DWeedSyn_final_EXP120B_HILICZ_USHXG02558_POS_MS2_34-UN-40_SP9509-mA289_4_Rg70to1050-CE102040norm-root-S1_Run221.h5

0021_trigonelline_positive_M138p0549_8p59 [M]+
Measured M/Z = 138.0549, 0.1466 ppm difference
Expected Elution of 8.59 minutes, 8.81 min actual

MSMS Scan at 8.817 minutes

Matching M/Zs above 1E-3*max: 92.049, 93.057, 94.065, 110.060, 138.055, 139.050

All Matching M/Zs: 92.049, 93.057, 94.065, 110.060, 138.055, 139.050
