# Supplementary material for: The metabolic and anatomical complexity of root microhabitats modulate their interaction with the microbiota
Source: Nat Commun. 2026 Aug 13;17:9738. doi: 10.1038/s41467-026-76440-4 (PMC13562559; doi:10.1038/s41467-026-76440-4)
Supplement: Supplementary file 5 [file 41467_2026_76440_MOESM12_ESM.zip › Supplementary Data 10/POS_msms_mirror_plots/0002_pyridoxine_positive_M+H170p0812_2p21.pdf]

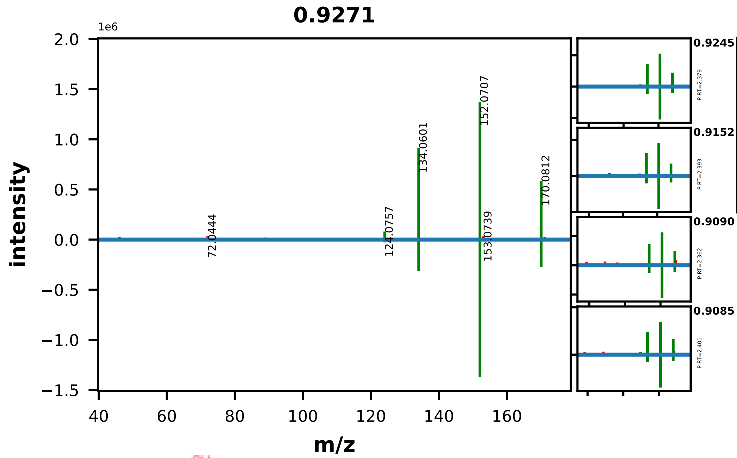


| | EMA Compound Info |
|---|---|
| Name: | pyridoxine |
| Label: | |
| Formula: | C8H11NO3 |
| Polarity: | positive |
| Monoisotopic Mass: | 169.073893212 |
| Theoretical M/Z: | 170.08117 |
| Adduct: | [M+H]+ |

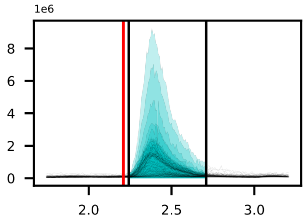


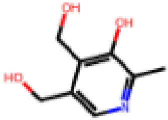


20240411_JGI_GC_509610_DWeedSyn_final_EXP120B_HILICZ_USHXG02558_POS_MS2_17-UN-20_SP9509-dA289_2_Rg70to1050-CE205060norm-root-S1_Run92.h5

0002_pyridoxine_positive_M+H170p0812_2p21 [M+H]+
Measured M/Z = 170.0812, 0.1096 ppm difference
Expected Elution of 2.21 minutes, 2.38 min actual

MSMS Scan at 2.371 minutes

Matching M/Zs above 1E-3*max: 96.081, 124.076, 134.060, 152.071, 170.081

All Matching M/Zs: 96.081, 124.076, 134.060, 152.071, 170.081
