# Supplementary material for: The metabolic and anatomical complexity of root microhabitats modulate their interaction with the microbiota
Source: Nat Commun. 2026 Aug 13;17:9738. doi: 10.1038/s41467-026-76440-4 (PMC13562559; doi:10.1038/s41467-026-76440-4)
Supplement: Supplementary file 5 [file 41467_2026_76440_MOESM12_ESM.zip › Supplementary Data 10/POS_msms_mirror_plots/0038_sn-glycero-3-phosphocholine_positive_M258p1101_14p77.pdf]

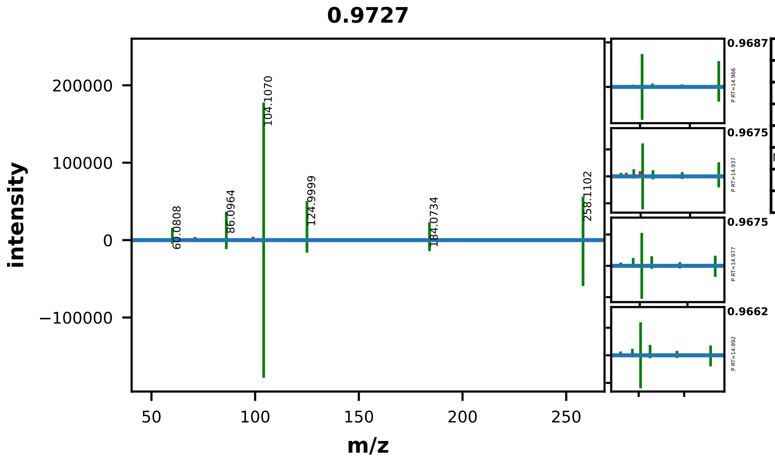

	EMA Compound Info
Name:	sn-glycero-3-phosphocholine
Label:	
Formula:	C8H21NO6P+
Polarity:	positive
Monoisotopic Mass:	258.110100442
Theoretical M/Z:	258.1101
Adduct:	[M]+

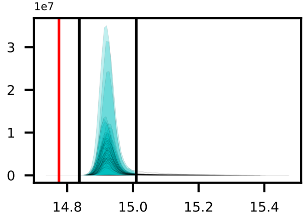

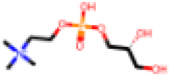

20240411_JGI_GC_509610_DWeedSyn_final_EXP120B_HILICZ_U
SHXG02558_POS_MS2_22-UN-26_SP9509-dA245_2_Rg70to1050-
CE205060norm-root-S1_Run111.h5

0038_sn-glycero-3-phosphocholine_positive_M258p1101_14p77 [M]+
Measured M/Z = 258.1101, 0.1207 ppm difference
Expected Elution of 14.77 minutes, 14.91 min actual

MSMS Scan at 14.968 minutes

Matching M/Zs above 1E-3*max: 60.081, 86.096, 104.107, 125.000, 184.073, 258.110

All Matching M/Zs: 60.081, 86.096, 104.107, 125.000, 184.073, 258.110
