# Supplementary material for: The metabolic and anatomical complexity of root microhabitats modulate their interaction with the microbiota
Source: Nat Commun. 2026 Aug 13;17:9738. doi: 10.1038/s41467-026-76440-4 (PMC13562559; doi:10.1038/s41467-026-76440-4)
Supplement: Supplementary file 5 [file 41467_2026_76440_MOESM12_ESM.zip › Supplementary Data 10/POS_msms_mirror_plots/0006_adenosine_positive_M+H268p1040_3p07.pdf]

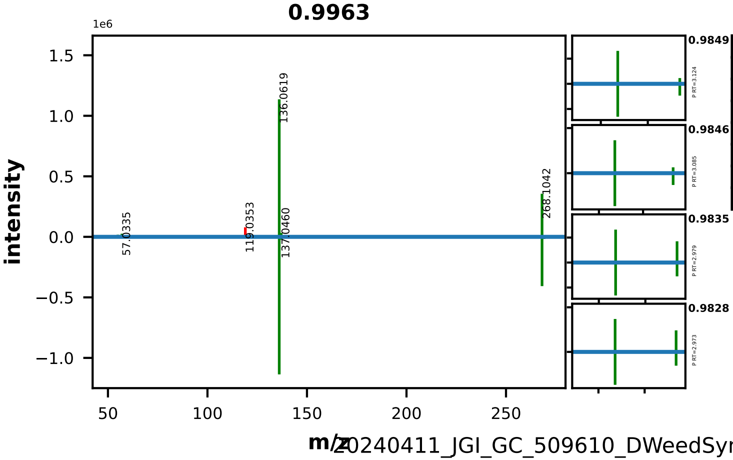

	EMA Compound Info
Name:	adenosine
Label:	
Formula:	C10H13N5O4
Polarity:	positive
Monoisotopic Mass:	267.096753896
Theoretical M/Z:	268.10403
Adduct:	[M+H]+

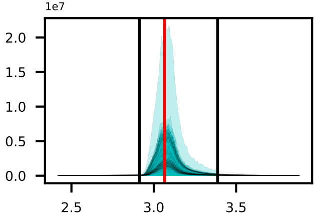

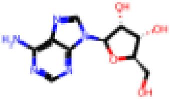

20240411_JGI_GC_509610_DWeedSyn_final_EXP120B_HILICZ_USHXG02558_POS_MS2_0_ISTD-V7-102040n205060abs_1_Rg70to1050-CE102040norm-root-ISTD_Run15.h5

0006_adenosine_positive_M+H268p1040_3p07 [M+H]+
Measured M/Z = 268.1040, 0.1774 ppm difference
Expected Elution of 3.07 minutes, 3.10 min actual

MSMS Scan at 3.091 minutes

Matching M/Zs above 1E-3*max: 55.018, 57.033, 73.029, 85.028, 115.039, 133.050, 136.062, 137.046, 268.104, 269.107

All Matching M/Zs: 55.018, 57.033, 73.029, 85.028, 115.039, 133.050, 136.062, 137.046, 268.104, 269.107
