# Supplementary material for: The metabolic and anatomical complexity of root microhabitats modulate their interaction with the microbiota
Source: Nat Commun. 2026 Aug 13;17:9738. doi: 10.1038/s41467-026-76440-4 (PMC13562559; doi:10.1038/s41467-026-76440-4)
Supplement: Supplementary file 5 [file 41467_2026_76440_MOESM12_ESM.zip › Supplementary Data 10/POS_msms_mirror_plots/0019_1-methyl-6_7-dihydroxy-1_2_3_4-tetrahydroisoquinoline_positive_M+H180p1019_8p02.pdf]

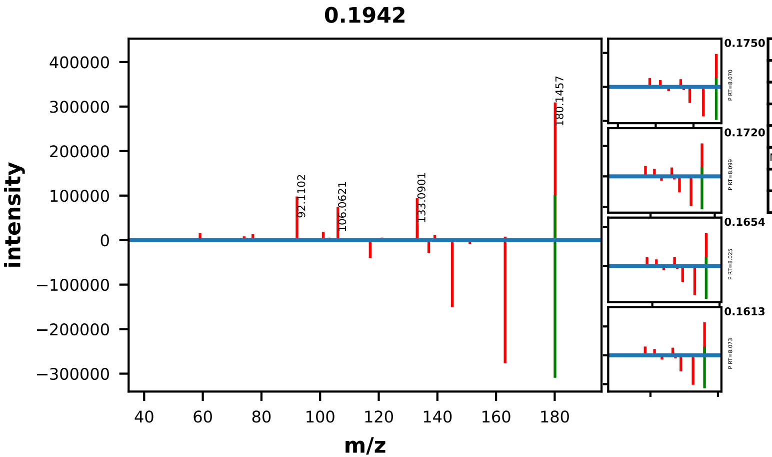

	EMA Compound Info
Name:	1-methyl-6,7-dihydroxy-1,2,3,4-[illegible]
Label:	
Formula:	C10H13NO2
Polarity:	positive
Monoisotopic Mass:	179.094628656
Theoretical M/Z:	180.10191
Adduct:	[M+H]+

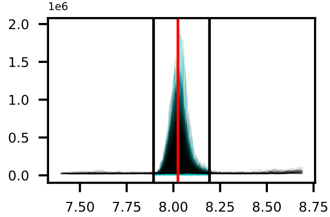

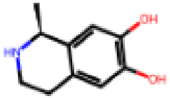

20240411_JGI_GC_509610_DWeedSyn_final_EXP120B_HILICZ_USHXG02558_POS_MS2_49-UN-58_LJ9250-dP65_1_Rg70to1050-CE102040norm-root-S1_Run37.h5

0019_1-methyl-6_7-dihydroxy-1_2_3_4-tetrahydroisoquinoline_positive_M+H18
Measured M/Z = 180.0991, 15.7008 ppm difference
Expected Elution of 8.02 minutes, 8.02 min actual

MSMS Scan at 8.060 minutes

Matching M/Zs above 1E-3*max: 180.099

All Matching M/Zs: 180.099
