# Supplementary material for: The metabolic and anatomical complexity of root microhabitats modulate their interaction with the microbiota
Source: Nat Commun. 2026 Aug 13;17:9738. doi: 10.1038/s41467-026-76440-4 (PMC13562559; doi:10.1038/s41467-026-76440-4)
Supplement: Supplementary file 5 [file 41467_2026_76440_MOESM12_ESM.zip › Supplementary Data 10/POS_msms_mirror_plots/0012_cytosine_positive_M+H112p0505_4p76.pdf]

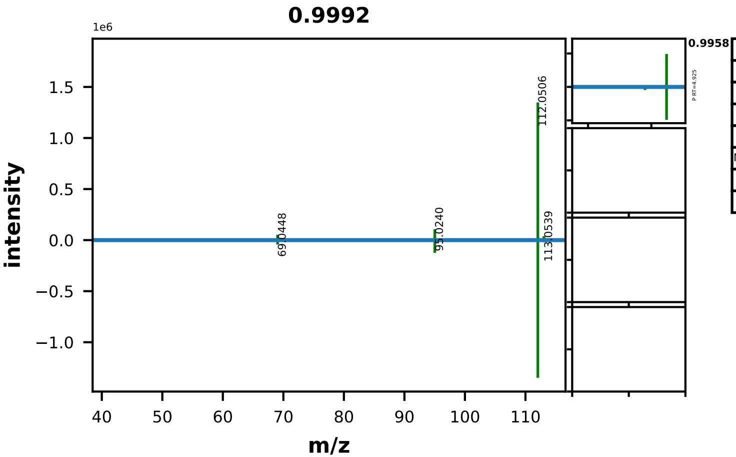

	EMA Compound Info
Name:	cytosine
Label:	
Formula:	C4H5N3O
Polarity:	positive
Monoisotopic Mass:	111.04326178
Theoretical M/Z:	112.05054
Adduct:	[M+H]+

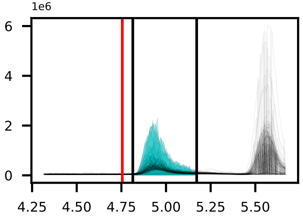

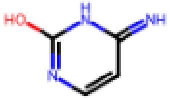

20240411_JGI_GC_509610_DWeedSyn_final_EXP120B_HILICZ_USHXG02558_POS_MS2_54-UN-64_LJ9250-dA289_2_Rg70to1050-CE205060norm-root-S1_Run107.h5

0012_cytosine_positive_M+H112p0505_4p76 [M+H]+
Measured M/Z = 112.0506, 0.4396 ppm difference
Expected Elution of 4.76 minutes, 4.92 min actual

MSMS Scan at 4.929 minutes

Matching M/Zs above 1E-3*max: 68.013, 69.045, 94.040, 95.024, 96.021, 112.051, 113.048, 113.054

All Matching M/Zs: 68.013, 69.045, 94.040, 95.024, 96.021, 112.051, 113.048, 113.054
