# Supplementary material for: The metabolic and anatomical complexity of root microhabitats modulate their interaction with the microbiota
Source: Nat Commun. 2026 Aug 13;17:9738. doi: 10.1038/s41467-026-76440-4 (PMC13562559; doi:10.1038/s41467-026-76440-4)
Supplement: Supplementary file 5 [file 41467_2026_76440_MOESM12_ESM.zip › Supplementary Data 10/POS_msms_mirror_plots/0034_lactose_positive_M+Na365p1054_14p13.pdf]

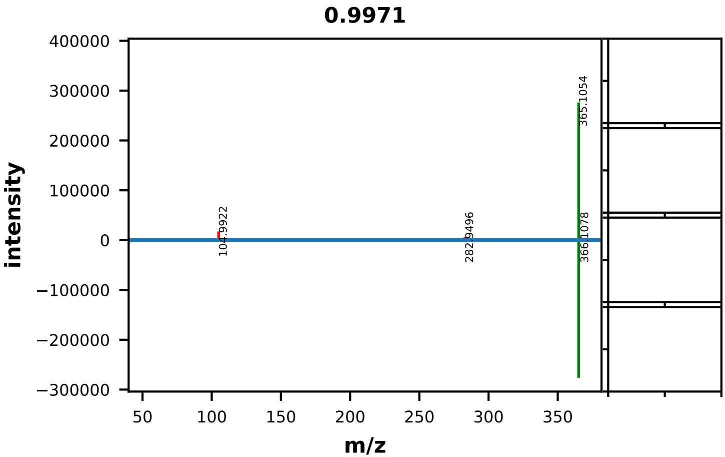

	EMA Compound Info
Name:	lactose
Label:	
Formula:	C12H22O11
Polarity:	positive
Monoisotopic Mass:	342.116211524
Theoretical M/Z:	365.10543
Adduct:	[M+Na]+

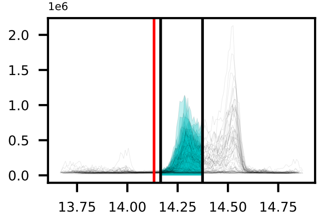

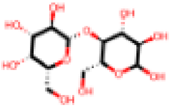

20240411_JGI_GC_509610_DWeedSyn_final_EXP120B_HILICZ_USHXG02558_POS_MS2_30-UN-35_SP9509-mP65_5_Rg70to1050-CE205060norm-root-S1_Run233.h5

0034_lactose_positive_M+Na365p1054_14p13 [M+Na]+
Measured M/Z = 365.1054, 0.1350 ppm difference
Expected Elution of 14.13 minutes, 14.28 min actual

MSMS Scan at 14.272 minutes

Matching M/Zs above 1E-3*max: 365.105

All Matching M/Zs: 365.105
