# Supplementary material for: The metabolic and anatomical complexity of root microhabitats modulate their interaction with the microbiota
Source: Nat Commun. 2026 Aug 13;17:9738. doi: 10.1038/s41467-026-76440-4 (PMC13562559; doi:10.1038/s41467-026-76440-4)
Supplement: Supplementary file 5 [file 41467_2026_76440_MOESM12_ESM.zip › Supplementary Data 10/POS_msms_mirror_plots/0036_5-aminopentanoic_acid_positive_M+H118p0863_14p23.pdf]

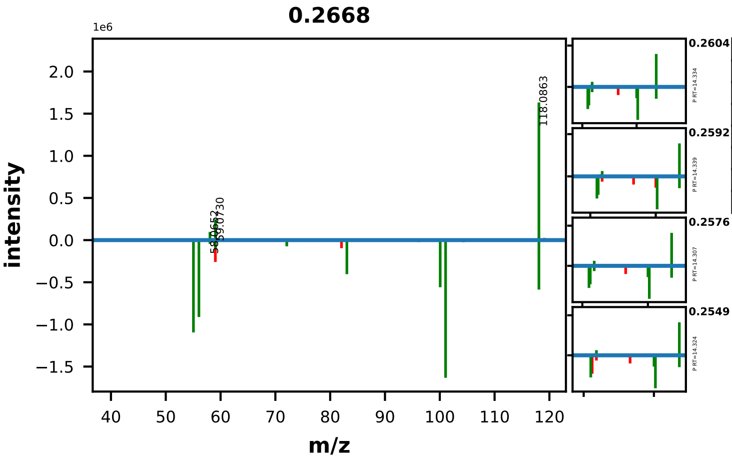

	EMA Compound Info
Name:	5-aminopentanoic acid
Label:	
Formula:	C5H11NO2
Polarity:	positive
Monoisotopic Mass:	117.078978592
Theoretical M/Z:	118.08626
Adduct:	[M+H]+

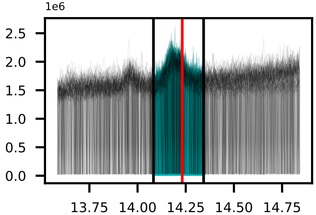

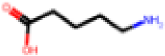

20240411_JGI_GC_509610_DWeedSyn_final_EXP120B_HILICZ_USHXG02558_POS_MS2_58-UN-69_LJ9250-dA245_2_Rg70to1050-CE205060norm-root-S1_Run89.h5

0036_5-aminopentanoic_acid_positive_M+H118p0863_14p23 [M+H]+
Measured M/Z = 118.0862, 0.6193 ppm difference
Expected Elution of 14.23 minutes, 14.21 min actual

MSMS Scan at 14.337 minutes

Matching M/Zs above 1E-3*max: 55.054, 56.049, 58.065, 59.073, 72.081, 83.049, 100.076, 101.060, 118.086

All Matching M/Zs: 55.054, 56.049, 58.065, 59.073, 72.081, 83.049, 100.076, 101.060, 118.086
