# Supplementary material for: The metabolic and anatomical complexity of root microhabitats modulate their interaction with the microbiota
Source: Nat Commun. 2026 Aug 13;17:9738. doi: 10.1038/s41467-026-76440-4 (PMC13562559; doi:10.1038/s41467-026-76440-4)
Supplement: Supplementary file 5 [file 41467_2026_76440_MOESM12_ESM.zip › Supplementary Data 10/POS_msms_mirror_plots/0014_guanine_positive_M+H152p0567_6p13.pdf]

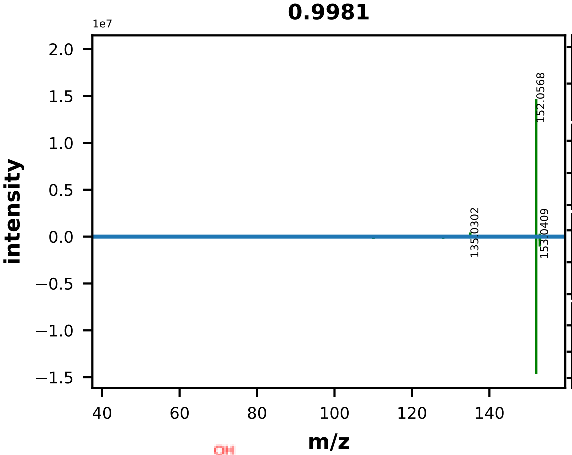

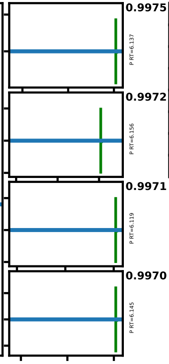

	EMA Compound Info
Name:	guanine
Label:	
Formula:	C5H5N5O
Polarity:	positive
Monoisotopic Mass:	151.04940978
Theoretical M/Z:	152.05669
Adduct:	[M+H]+

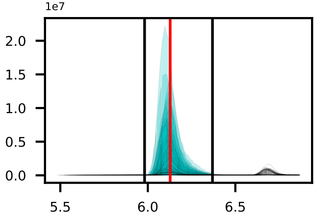

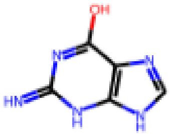

20240411_JGI_GC_509610_DWeedSyn_final_EXP120B_HILICZ_USHXG02558_POS_MS2_14-UN-16_SP9509-dP65_4_Rg70to1050-CE102040norm-root-S1_Run187.h5

0014_guanine_positive_M+H152p0567_6p13 [M+H]+
Measured M/Z = 152.0567, 0.0162 ppm difference
Expected Elution of 6.13 minutes, 6.10 min actual

MSMS Scan at 6.108 minutes

Matching M/Zs above 1E-3*max: 59.073, 109.051, 110.035, 128.046, 134.046, 135.030, 152.057, 153.041, 153.060

All Matching M/Zs: 59.073, 109.051, 110.035, 128.046, 134.046, 135.030, 152.057, 153.041, 153.060
