# Supplementary material for: The metabolic and anatomical complexity of root microhabitats modulate their interaction with the microbiota
Source: Nat Commun. 2026 Aug 13;17:9738. doi: 10.1038/s41467-026-76440-4 (PMC13562559; doi:10.1038/s41467-026-76440-4)
Supplement: Supplementary file 5 [file 41467_2026_76440_MOESM12_ESM.zip › Supplementary Data 10/POS_msms_mirror_plots/0030_valine_positive_M+H118p0863_10p87.pdf]

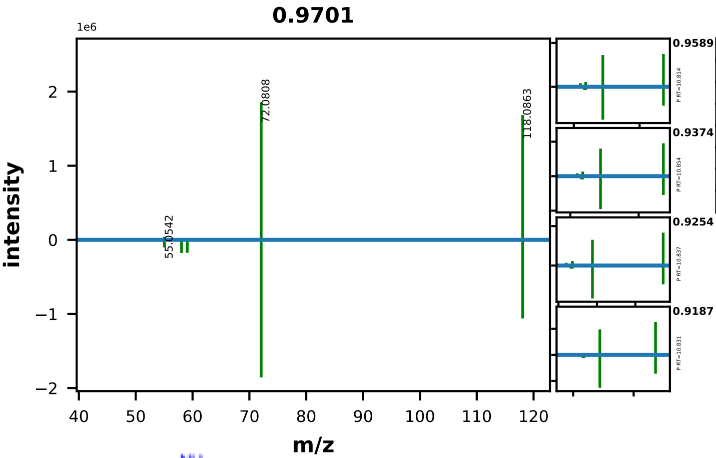

	EMA Compound Info
Name:	valine
Label:	
Formula:	C5H11NO2
Polarity:	positive
Monoisotopic Mass:	117.078978592
Theoretical M/Z:	118.08626
Adduct:	[M+H]+

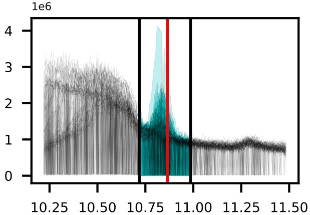

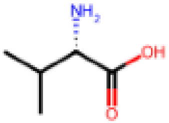

20240411_JGI_GC_509610_DWeedSyn_final_EXP120B_HILICZ_USHXG02558_POS_MS2_14-UN-16_SP9509-dP65_4_Rg70to1050-CE102040norm-root-S1_Run187.h5

0030_valine_positive_M+H118p0863_10p87 [M+H]+
Measured M/Z = 118.0862, 0.5325 ppm difference
Expected Elution of 10.87 minutes, 10.81 min actual

MSMS Scan at 10.844 minutes

Matching M/Zs above 1E-3*max: 55.054, 57.057, 58.065, 59.049, 59.073, 72.081, 118.086, 118.122

All Matching M/Zs: 55.054, 57.057, 58.065, 59.049, 59.073, 72.081, 118.086, 118.122
