# Supplementary material for: The metabolic and anatomical complexity of root microhabitats modulate their interaction with the microbiota
Source: Nat Commun. 2026 Aug 13;17:9738. doi: 10.1038/s41467-026-76440-4 (PMC13562559; doi:10.1038/s41467-026-76440-4)
Supplement: Supplementary file 5 [file 41467_2026_76440_MOESM12_ESM.zip › Supplementary Data 10/POS_msms_mirror_plots/0022_phenylalanine_positive_M+H166p0863_8p77.pdf]

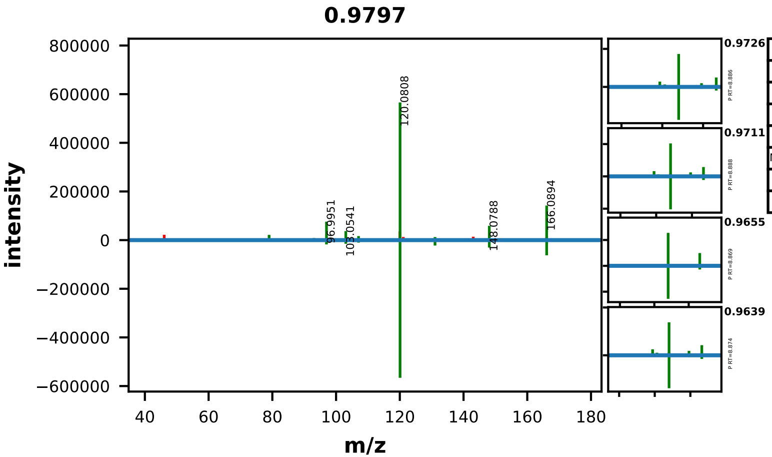

	EMA Compound Info
Name:	phenylalanine
Label:	
Formula:	C9H11NO2
Polarity:	positive
Monoisotopic Mass:	165.078978592
Theoretical M/Z:	166.08626
Adduct:	[M+H]+

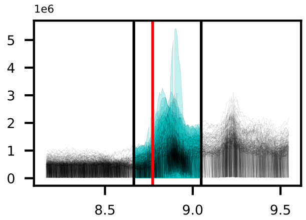

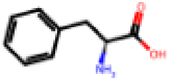

20240411_JGI_GC_509610_DWeedSyn_final_EXP120B_HILICZ_USHXG02558_POS_MS2_15-UN-17_SP9509-dP65_5_Rg70to1050-CE205060norm-root-S1_Run236.h5

0022_phenylalanine_positive_M+H166p0863_8p77 [M+H]+
Measured M/Z = 166.0883, 12.4775 ppm difference
Expected Elution of 8.77 minutes, 8.90 min actual

MSMS Scan at 8.886 minutes

Matching M/Zs above 1E-3*max: 78.985, 79.054, 93.070, 96.995, 103.054, 107.049, 120.081, 131.049, 148.079, 149.060, 166.089

All Matching M/Zs: 78.985, 79.054, 93.070, 96.995, 103.054, 107.049, 120.081, 131.049, 148.079, 149.060, 166.089
