# Supplementary material for: The metabolic and anatomical complexity of root microhabitats modulate their interaction with the microbiota
Source: Nat Commun. 2026 Aug 13;17:9738. doi: 10.1038/s41467-026-76440-4 (PMC13562559; doi:10.1038/s41467-026-76440-4)
Supplement: Supplementary file 5 [file 41467_2026_76440_MOESM12_ESM.zip › Supplementary Data 10/POS_msms_mirror_plots/0043_N-methylanthranilic_acid_positive_M+H-H2O134p0600_1p20.pdf]

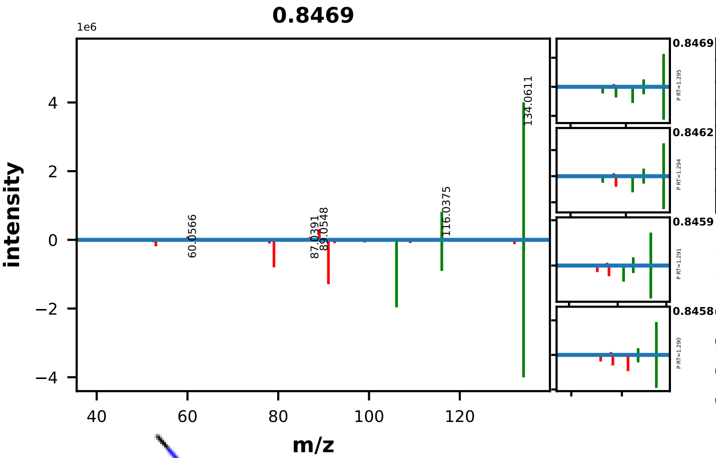

	EMA Compound Info
Name:	N-methylanthranilic acid
Label:	
Formula:	C8H9NO2
Polarity:	positive
Monoisotopic Mass:	151.063328528
Theoretical M/Z:	134.06004
Adduct:	[M+H-H2O]+

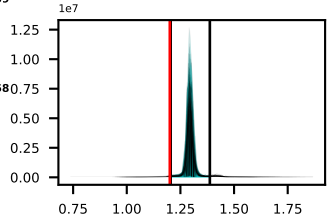

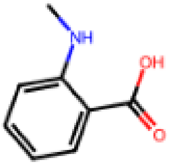

20240411_JGI_GC_509610_DWeedSyn_final_EXP120B_HILICZ_USHXG02558_POS_MS2_15-UN-17_SP9509-dP65_5_Rg70to1050-CE205060norm-root-S1_Run236.h5

0043_N-methylanthranilic_acid_positive_M+H-H2O134p0600_1p20 [M+H-H2O
Measured M/Z = 134.0610, 7.2343 ppm difference
Expected Elution of 1.20 minutes, 1.29 min actual

MSMS Scan at 1.296 minutes

Matching M/Zs above 1E-3*max: 116.037, 134.050, 134.061, 134.075

All Matching M/Zs: 106.065, 116.037, 134.050, 134.061, 134.075
