# Supplementary material for: The metabolic and anatomical complexity of root microhabitats modulate their interaction with the microbiota
Source: Nat Commun. 2026 Aug 13;17:9738. doi: 10.1038/s41467-026-76440-4 (PMC13562559; doi:10.1038/s41467-026-76440-4)
Supplement: Supplementary file 5 [file 41467_2026_76440_MOESM12_ESM.zip › Supplementary Data 10/POS_msms_mirror_plots/0016_pantothenic_acid_positive_M+H220p1180_6p78.pdf]

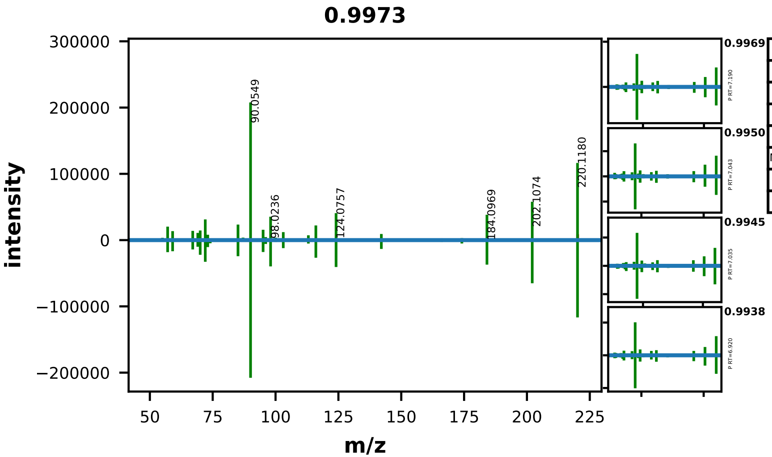

	EMA Compound Info
Name:	pantothenic acid
Label:	
Formula:	C9H17NO5
Polarity:	positive
Monoisotopic Mass:	219.110672644
Theoretical M/Z:	220.11795
Adduct:	[M+H]+

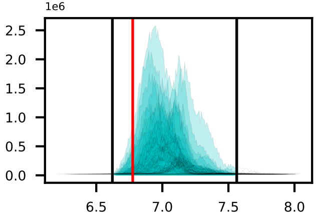

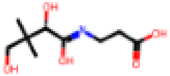

20240411_JGI_GC_509610_DWeedSyn_final_EXP120B_HILICZ_USHXG02558_POS_MS2_22-UN-26_SP9509-dA245_2_Rg70to1050-CE205060norm-root-S1_Run111.h5

0016_pantothenic_acid_positive_M+H220p1180_6p78 [M+H]+
Measured M/Z = 220.1179, 0.2656 ppm difference
Expected Elution of 6.78 minutes, 6.95 min actual

MSMS Scan at 6.902 minutes

Matching M/Zs above 1E-3*max: 55.018, 55.054, 57.070, 59.049, 67.054, 69.070, 70.029, 72.044, 73.028, 74.024, 83.049, 85.065, 86.096, 87.081, 90.055, 95.049, 96.081, 98.024, 100.039, 103.075, 113.060, 116.034, 124.076, 142.086, 160.097, 166.086, 174.113, 184.097, 202.107, 220.118

All Matching M/Zs: 55.018, 55.054, 57.070, 59.049, 67.054, 69.070, 70.029, 72.044, 73.028, 74.024, 83.049, 85.065, 86.096, 87.081, 90.055, 95.049, 96.081, 98.024, 100.039, 103.075, 113.060, 116.034, 124.076, 142.086, 160.097, 166.086, 174.113, 184.097, 202.107, 220.118
