# Supplementary material for: The metabolic and anatomical complexity of root microhabitats modulate their interaction with the microbiota
Source: Nat Commun. 2026 Aug 13;17:9738. doi: 10.1038/s41467-026-76440-4 (PMC13562559; doi:10.1038/s41467-026-76440-4)
Supplement: Supplementary file 5 [file 41467_2026_76440_MOESM12_ESM.zip › Supplementary Data 10/POS_msms_mirror_plots/0010_5-methylcytosine_positive_M+H126p0662_4p34.pdf]

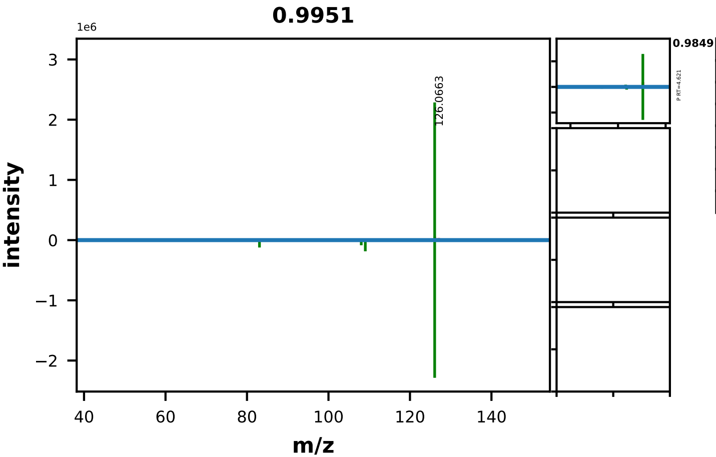

	EMA Compound Info
Name:	5-methylcytosine
Label:	
Formula:	C5H7N3O
Polarity:	positive
Monoisotopic Mass:	125.058911844
Theoretical M/Z:	126.06619
Adduct:	[M+H]+

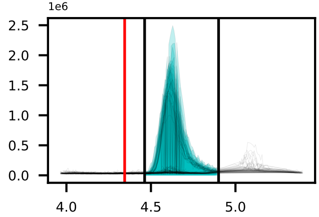

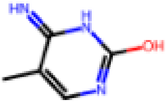

20240411_JGI_GC_509610_DWeedSyn_final_EXP120B_HILICZ_USHXG02558_POS_MS2_0_ISTD_Post_Rg70to1050-CE102040norm--ISTD_Run258.h5

0010_5-methylcytosine_positive_M+H126p0662_4p34 [M+H]+
Measured M/Z = 126.0661, 0.3496 ppm difference
Expected Elution of 4.34 minutes, 4.63 min actual

MSMS Scan at 4.662 minutes

Matching M/Zs above 1E-3*max: 83.060, 108.044, 109.040, 126.066

All Matching M/Zs: 56.049, 83.060, 108.044, 109.040, 126.066
