# Supplementary material for: The metabolic and anatomical complexity of root microhabitats modulate their interaction with the microbiota
Source: Nat Commun. 2026 Aug 13;17:9738. doi: 10.1038/s41467-026-76440-4 (PMC13562559; doi:10.1038/s41467-026-76440-4)
Supplement: Supplementary file 5 [file 41467_2026_76440_MOESM12_ESM.zip › Supplementary Data 10/POS_msms_mirror_plots/0026_isoleucine_positive_M+H132p1019_9p52.pdf]

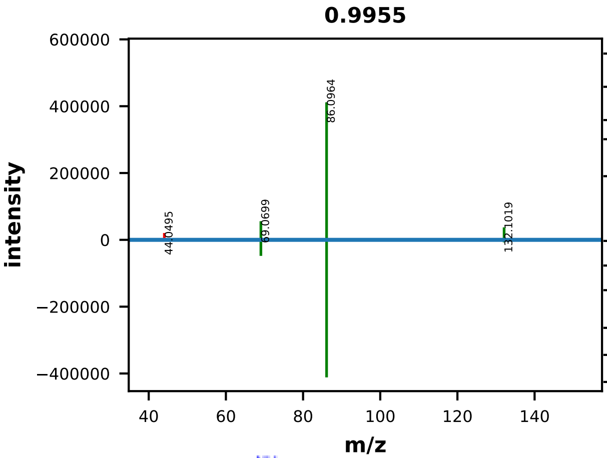

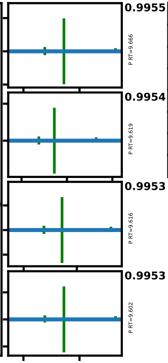

	EMA Compound Info
Name:	isoleucine
Label:	
Formula:	C6H13NO2
Polarity:	positive
Monoisotopic Mass:	131.094628656
Theoretical M/Z:	132.10191
Adduct:	[M+H]+

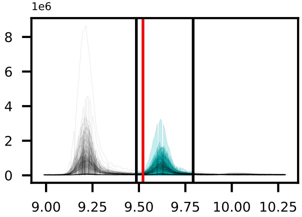

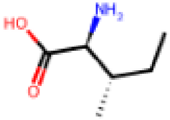

20240411_JGI_GC_509610_DWeedSyn_final_EXP120B_HILICZ_U
SHXG02558_POS_MS2_17-UN-20_SP9509-dA289_2_Rg70to1050-
CE205060norm-root-S1_Run92.h5

0026_isoleucine_positive_M+H132p1019_9p52 [M+H]+
Measured M/Z = 132.1019, 0.2543 ppm difference
Expected Elution of 9.52 minutes, 9.62 min actual

MSMS Scan at 9.629 minutes

Matching M/Zs above 1E-3*max: 58.065, 69.070, 86.096, 132.102

All Matching M/Zs: 58.065, 69.070, 86.096, 132.102
