# Supplementary material for: The metabolic and anatomical complexity of root microhabitats modulate their interaction with the microbiota
Source: Nat Commun. 2026 Aug 13;17:9738. doi: 10.1038/s41467-026-76440-4 (PMC13562559; doi:10.1038/s41467-026-76440-4)
Supplement: Supplementary file 5 [file 41467_2026_76440_MOESM12_ESM.zip › Supplementary Data 10/POS_msms_mirror_plots/0005_4-pyridoxic_acid_positive_M+H184p0604_2p89.pdf]

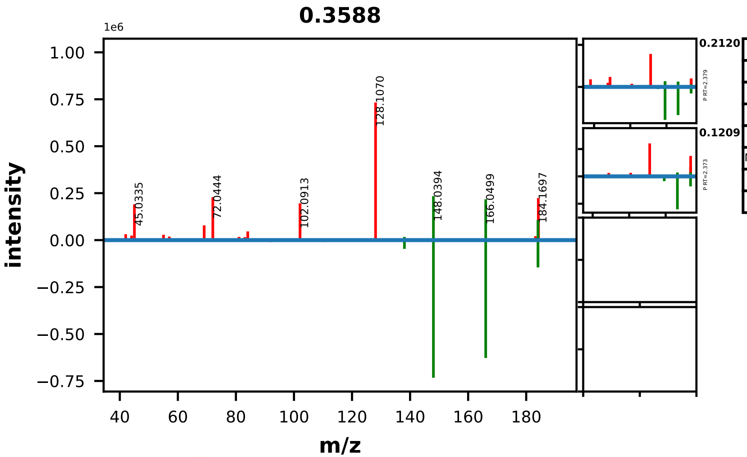

	EMA Compound Info
Name:	4-pyridoxic acid
Label:	
Formula:	C8H9NO4
Polarity:	positive
Monoisotopic Mass:	183.053157768
Theoretical M/Z:	184.06043
Adduct:	[M+H]+

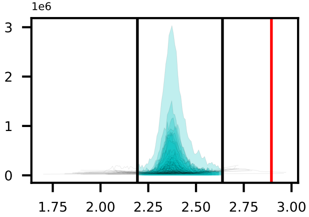

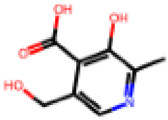

20240411_JGI_GC_509610_DWeedSyn_final_EXP120B_HILICZ_USHXG02558_POS_MS2_17-UN-20_SP9509-dA289_2_Rg70to1050-CE205060norm-root-S1_Run92.h5

0005_4-pyridoxic_acid_positive_M+H184p0604_2p89 [M+H]+
Measured M/Z = 184.0604, 0.0515 ppm difference
Expected Elution of 2.89 minutes, 2.37 min actual

MSMS Scan at 2.331 minutes

Matching M/Zs above 1E-3*max: 138.055, 148.039, 166.050, 184.061

All Matching M/Zs: 138.055, 148.039, 166.050, 184.061
