# Supplementary material for: The metabolic and anatomical complexity of root microhabitats modulate their interaction with the microbiota
Source: Nat Commun. 2026 Aug 13;17:9738. doi: 10.1038/s41467-026-76440-4 (PMC13562559; doi:10.1038/s41467-026-76440-4)
Supplement: Supplementary file 5 [file 41467_2026_76440_MOESM12_ESM.zip › Supplementary Data 10/POS_msms_mirror_plots/0013_deoxycytidine_positive_M+H228p0979_5p50.pdf]

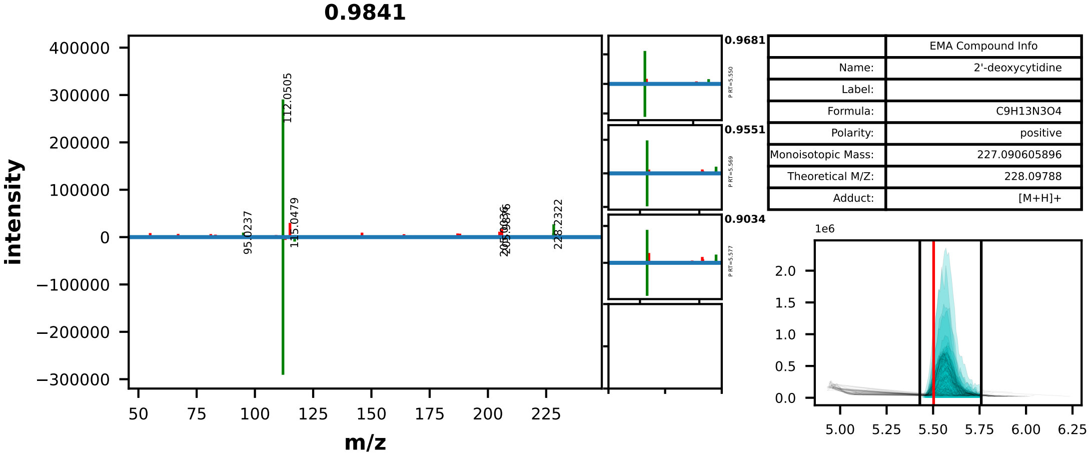

	EMA Compound Info
Name:	2'-deoxycytidine
Label:	
Formula:	C9H13N3O4
Polarity:	positive
Monoisotopic Mass:	227.090605896
Theoretical M/Z:	228.09788
Adduct:	[M+H]+

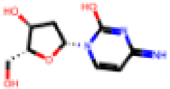

20240411_JGI_GC_509610_DWeedSyn_final_EXP120B_HILICZ_USHXG02558_POS_MS2_54-UN-64_LJ9250-dA289_2_Rg70to1050-CE205060norm-root-S1_Run107.h5

0013_deoxycytidine_positive_M+H228p0979_5p50 [M+H]+
Measured M/Z = 228.0978, 0.4712 ppm difference
Expected Elution of 5.50 minutes, 5.57 min actual

MSMS Scan at 5.536 minutes

Matching M/Zs above 1E-3*max: 69.045, 71.049, 73.028, 95.024, 99.044, 112.051, 117.054, 228.122, 228.232

All Matching M/Zs: 69.045, 71.049, 73.028, 95.024, 99.044, 112.051, 117.054, 228.122, 228.232
