# Supplementary material for: The metabolic and anatomical complexity of root microhabitats modulate their interaction with the microbiota
Source: Nat Commun. 2026 Aug 13;17:9738. doi: 10.1038/s41467-026-76440-4 (PMC13562559; doi:10.1038/s41467-026-76440-4)
Supplement: Supplementary file 5 [file 41467_2026_76440_MOESM12_ESM.zip › Supplementary Data 10/POS_msms_mirror_plots/0040_glutamic_acid_positive_M+H148p0604_15p83.pdf]

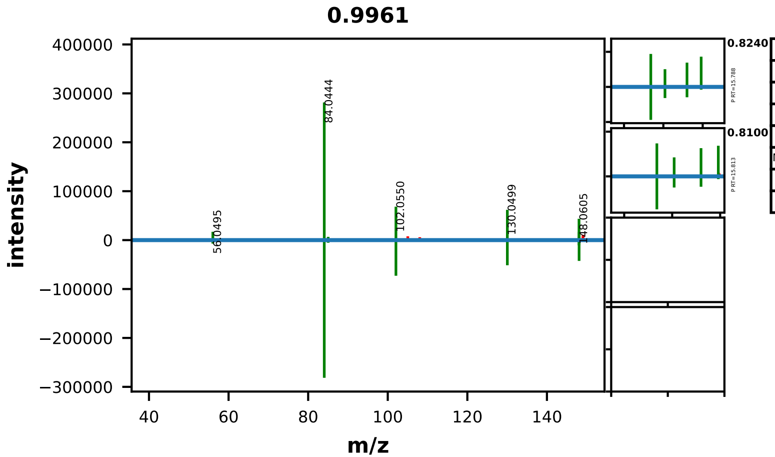

	EMA Compound Info
Name:	glutamic acid
Label:	
Formula:	C5H9NO4
Polarity:	positive
Monoisotopic Mass:	147.053157768
Theoretical M/Z:	148.06043
Adduct:	[M+H]+

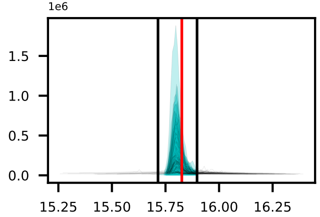

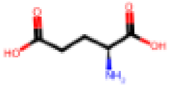

20240411_JGI_GC_509610_DWeedSyn_final_EXP120B_HILICZ_USHXG02558_POS_MS2_17-UN-20_SP9509-dA289_2_Rg70to1050-CE205060norm-root-S1_Run92.h5

0040_glutamic_acid_positive_M+H148p0604_15p83 [M+H]+
Measured M/Z = 148.0604, 0.0659 ppm difference
Expected Elution of 15.83 minutes, 15.81 min actual

MSMS Scan at 15.820 minutes

Matching M/Zs above 1E-3*max: 56.049, 84.044, 85.028, 102.055, 130.050, 148.060, 148.079

All Matching M/Zs: 56.049, 84.044, 85.028, 102.055, 130.050, 148.060, 148.079
