# Supplementary material for: The metabolic and anatomical complexity of root microhabitats modulate their interaction with the microbiota
Source: Nat Commun. 2026 Aug 13;17:9738. doi: 10.1038/s41467-026-76440-4 (PMC13562559; doi:10.1038/s41467-026-76440-4)
Supplement: Supplementary file 5 [file 41467_2026_76440_MOESM12_ESM.zip › Supplementary Data 10/POS_msms_mirror_plots/0004_adenine_positive_M+H136p0618_2p59.pdf]

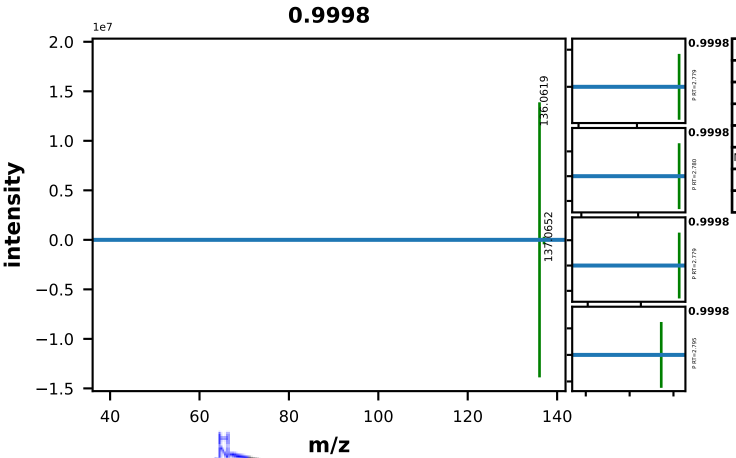

	EMA Compound Info
Name:	adenine
Label:	
Formula:	C5H5N5
Polarity:	positive
Monoisotopic Mass:	135.05449516
Theoretical M/Z:	136.06177
Adduct:	[M+H]+

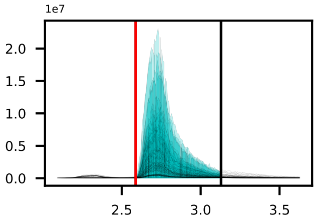

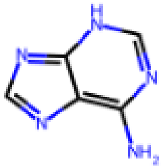

20240411_JGI_GC_509610_DWeedSyn_final_EXP120B_HILICZ_USHXG02558_POS_MS2_34-UN-40_SP9509-mA289_4_Rg70to1050-CE102040norm-root-S1_Run221.h5

0004_adenine_positive_M+H136p0618_2p59 [M+H]+
Measured M/Z = 136.0618, 0.1910 ppm difference
Expected Elution of 2.59 minutes, 2.72 min actual

MSMS Scan at 2.715 minutes

Matching M/Zs above 1E-3*max: 119.035, 136.062, 137.059, 137.065

All Matching M/Zs: 94.040, 119.035, 136.062, 137.059, 137.065
