# Supplementary material for: The metabolic and anatomical complexity of root microhabitats modulate their interaction with the microbiota
Source: Nat Commun. 2026 Aug 13;17:9738. doi: 10.1038/s41467-026-76440-4 (PMC13562559; doi:10.1038/s41467-026-76440-4)
Supplement: Supplementary file 5 [file 41467_2026_76440_MOESM12_ESM.zip › Supplementary Data 10/POS_msms_mirror_plots/0001_nicotinamide_positive_M+H123p0553_1p34.pdf]

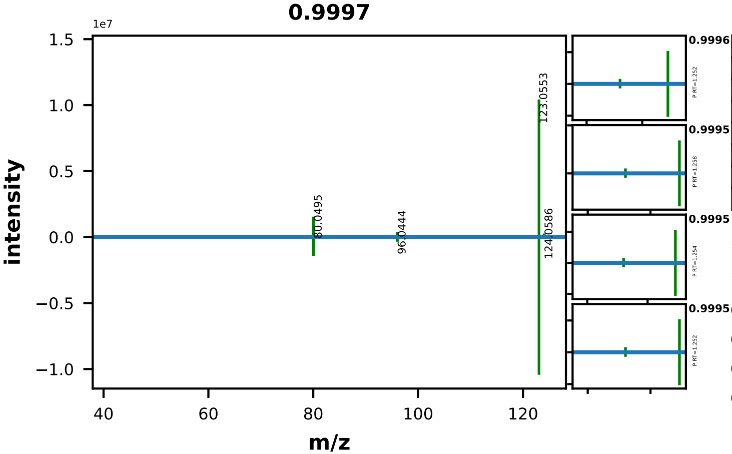

	EMA Compound Info
Name:	nicotinamide
Label:	
Formula:	C6H6N2O
Polarity:	positive
Monoisotopic Mass:	122.048012812
Theoretical M/Z:	123.05529
Adduct:	[M+H]+

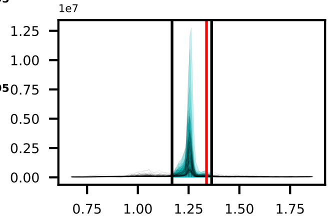

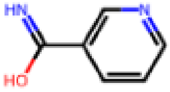

20240411_JGI_GC_509610_DWeedSyn_final_EXP120B_HILICZ_USHXG02558_POS_MS2_27-UN-32_SP9509-mP65_2_Rg70to1050-CE205060norm-root-S1_Run117.h5

0001_nicotinamide_positive_M+H123p0553_1p34 [M+H]+
Measured M/Z = 123.0552, 0.3894 ppm difference
Expected Elution of 1.34 minutes, 1.26 min actual

MSMS Scan at 1.251 minutes

Matching M/Zs above 1E-3*max: 78.034, 79.042, 80.049, 96.044, 106.029, 123.055, 124.059

All Matching M/Zs: 78.034, 79.042, 80.049, 96.044, 106.029, 123.055, 124.059
