# Supplementary material for: The metabolic and anatomical complexity of root microhabitats modulate their interaction with the microbiota
Source: Nat Commun. 2026 Aug 13;17:9738. doi: 10.1038/s41467-026-76440-4 (PMC13562559; doi:10.1038/s41467-026-76440-4)
Supplement: Supplementary file 5 [file 41467_2026_76440_MOESM12_ESM.zip › Supplementary Data 10/POS_msms_mirror_plots/0039_raffinose_positive_M+Na527p1583_15p41.pdf]

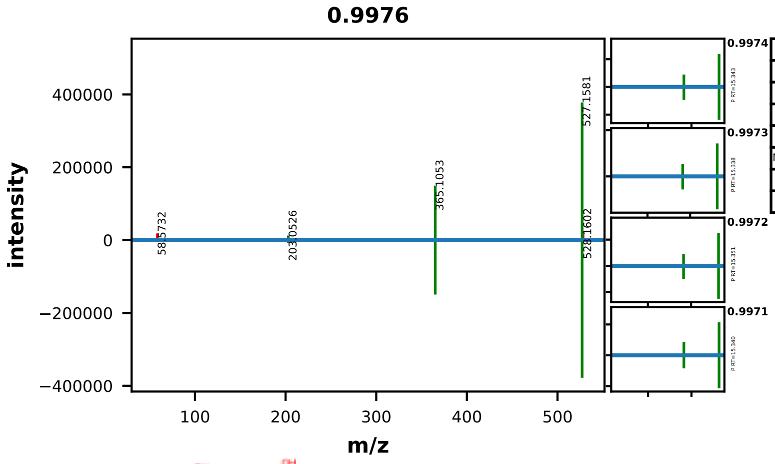

	EMA Compound Info
Name:	raffinose
Label:	
Formula:	C18H32O16
Polarity:	positive
Monoisotopic Mass:	504.169034944
Theoretical M/Z:	527.15826
Adduct:	[M+Na]+

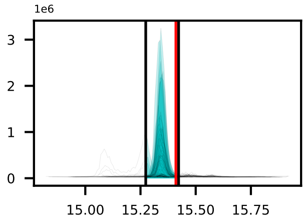

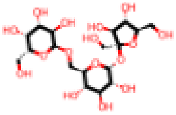

20240411_JGI_GC_509610_DWeedSyn_final_EXP120B_HILICZ_USHXG02558_POS_MS2_22-UN-26_SP9509-dA245_2_Rg70to1050-CE205060norm-root-S1_Run111.h5

0039_raffinose_positive_M+Na527p1583_15p41 [M+Na]+
Measured M/Z = 527.1583, 0.0688 ppm difference
Expected Elution of 15.41 minutes, 15.34 min actual

MSMS Scan at 15.339 minutes

Matching M/Zs above 1E-3*max: 185.042, 203.053, 305.085, 347.095, 365.105, 527.158

All Matching M/Zs: 185.042, 203.053, 305.085, 347.095, 365.105, 527.158
