# Supplementary material for: The metabolic and anatomical complexity of root microhabitats modulate their interaction with the microbiota
Source: Nat Commun. 2026 Aug 13;17:9738. doi: 10.1038/s41467-026-76440-4 (PMC13562559; doi:10.1038/s41467-026-76440-4)

	EMA Compound Info
Name:	epinephrine
Label:	
Formula:	C9H13NO3
Polarity:	positive
Monoisotopic Mass:	183.089543276
Theoretical M/Z:	166.08626
Adduct:	[M+H-H2O]+

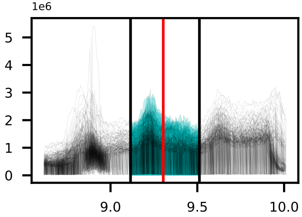

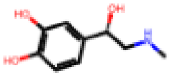

Supplement: Supplementary file 5 [file 41467_2026_76440_MOESM12_ESM.zip › Supplementary Data 10/POS_msms_mirror_plots/0025_epinephrine_positive_M+H-H2O166p0863_9p30.pdf]
