# Supplementary material for: The metabolic and anatomical complexity of root microhabitats modulate their interaction with the microbiota
Source: Nat Commun. 2026 Aug 13;17:9738. doi: 10.1038/s41467-026-76440-4 (PMC13562559; doi:10.1038/s41467-026-76440-4)
Supplement: Supplementary file 5 [file 41467_2026_76440_MOESM12_ESM.zip › Supplementary Data 10/POS_msms_mirror_plots/0042_Indole-3-carboxaldehyde_positive_M+H146p0600_1p06.pdf]

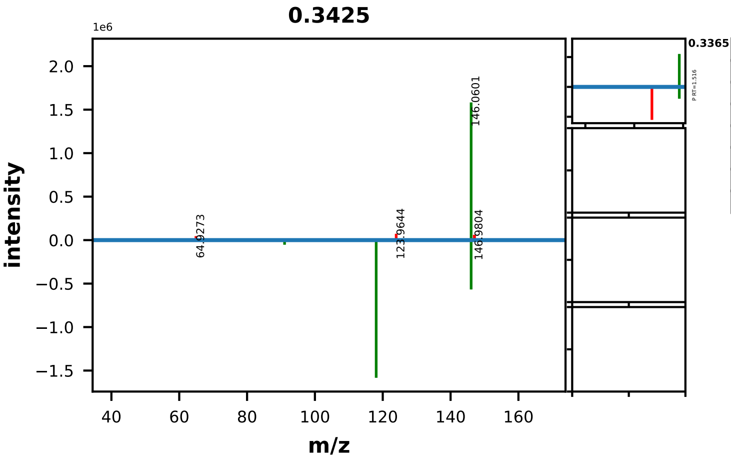

	EMA Compound Info
Name:	Indole-3-carboxaldehyde
Label:	
Formula:	C9H7NO
Polarity:	positive
Monoisotopic Mass:	145.052763844
Theoretical M/Z:	146.06004
Adduct:	[M+H]+

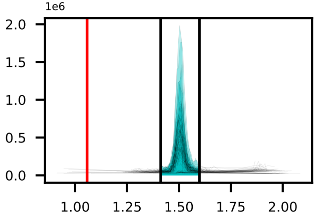

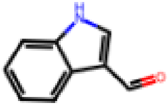

20240411_JGI_GC_509610_DWeedSyn_final_EXP120B_HILICZ_USHXG02558_POS_MS2_27-UN-32_SP9509-mP65_2_Rg70to1050-CE205060norm-root-S1_Run117.h5

0042_Indole-3-carboxaldehyde_positive_M+H146p0600_1p06 [M+H]+
Measured M/Z = 146.0600, 0.0081 ppm difference
Expected Elution of 1.06 minutes, 1.51 min actual

MSMS Scan at 1.518 minutes

Matching M/Zs above 1E-3*max: 91.054, 118.065, 146.060

All Matching M/Zs: 91.054, 118.065, 146.060
