# Supplementary material for: The metabolic and anatomical complexity of root microhabitats modulate their interaction with the microbiota
Source: Nat Commun. 2026 Aug 13;17:9738. doi: 10.1038/s41467-026-76440-4 (PMC13562559; doi:10.1038/s41467-026-76440-4)
Supplement: Supplementary file 5 [file 41467_2026_76440_MOESM12_ESM.zip › Supplementary Data 10/POS_msms_mirror_plots/0003_2deoxyadenosine_positive_M+H252p1091_2p28.pdf]

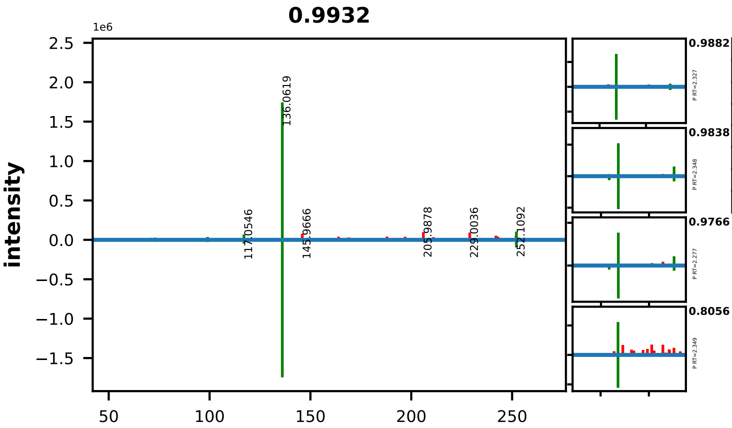

	EMA Compound Info
Name:	2'-deoxyadenosine
Label:	
Formula:	C10H13N5O3
Polarity:	positive
Monoisotopic Mass:	251.101839276
Theoretical M/Z:	252.10912
Adduct:	[M+H]+

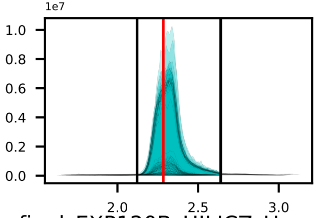

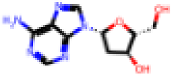

20240411_JGI_GC_509610_DWeedSyn_final_EXP120B_HILICZ_USHXG02558_POS_MS2_0_ISTD-V7-102040n205060norm_1_Rg70to1050-CE102040norm-root-ISTD_Run14.h5

0003_2deoxyadenosine_positive_M+H252p1091_2p28 [M+H]+
Measured M/Z = 252.1090, 0.3245 ppm difference
Expected Elution of 2.28 minutes, 2.32 min actual

MSMS Scan at 2.315 minutes

Matching M/Zs above 1E-3*max: 57.034, 69.033, 71.049, 73.028, 81.033, 99.044, 117.055, 119.035, 136.062, 137.066, 252.109

All Matching M/Zs: 57.034, 69.033, 71.049, 73.028, 81.033, 99.044, 117.055, 119.035, 136.062, 137.066, 252.109
