# Supplementary material for: The metabolic and anatomical complexity of root microhabitats modulate their interaction with the microbiota
Source: Nat Commun. 2026 Aug 13;17:9738. doi: 10.1038/s41467-026-76440-4 (PMC13562559; doi:10.1038/s41467-026-76440-4)
Supplement: Supplementary file 5 [file 41467_2026_76440_MOESM12_ESM.zip › Supplementary Data 10/POS_msms_mirror_plots/0018_betaine_positive_M118p0863_7p75.pdf]

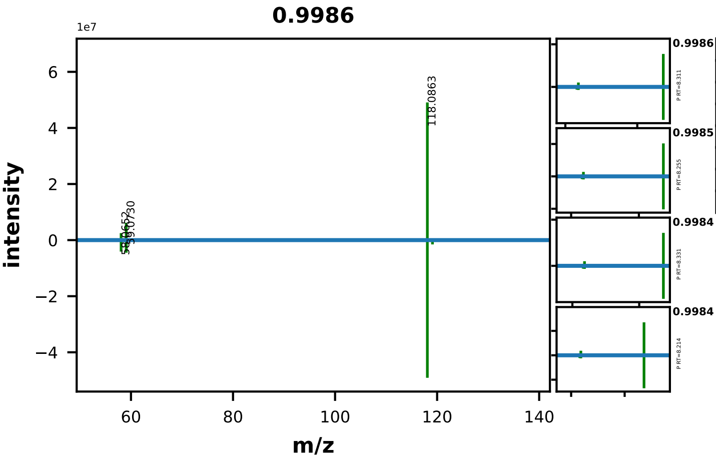

	EMA Compound Info
Name:	betaine
Label:	
Formula:	C5H12NO2+
Polarity:	positive
Monoisotopic Mass:	118.086255044
Theoretical M/Z:	118.08626
Adduct:	[M]+

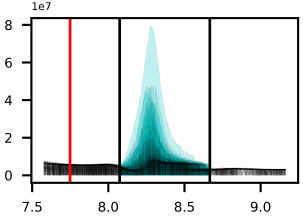

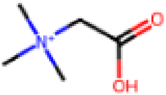

20240411_JGI_GC_509610_DWeedSyn_final_EXP120B_HILICZ_USHXG02558_POS_MS2_25-UN-29_SP9509-dA245_5_Rg70to1050-CE205060norm-root-S1_Run239.h5

0018_betaine_positive_M118p0863_7p75 [M]+
Measured M/Z = 118.0862, 0.4166 ppm difference
Expected Elution of 7.75 minutes, 8.28 min actual

MSMS Scan at 8.308 minutes

Matching M/Zs above 1E-3*max: 58.065, 59.073, 60.076, 118.086, 119.084, 119.090

All Matching M/Zs: 58.065, 59.073, 60.076, 118.086, 119.084, 119.090
