# Supplementary material for: The metabolic and anatomical complexity of root microhabitats modulate their interaction with the microbiota
Source: Nat Commun. 2026 Aug 13;17:9738. doi: 10.1038/s41467-026-76440-4 (PMC13562559; doi:10.1038/s41467-026-76440-4)
Supplement: Supplementary file 5 [file 41467_2026_76440_MOESM12_ESM.zip › Supplementary Data 10/POS_msms_mirror_plots/0041_stachyose_positive_M+Na689p2111_16p61.pdf]

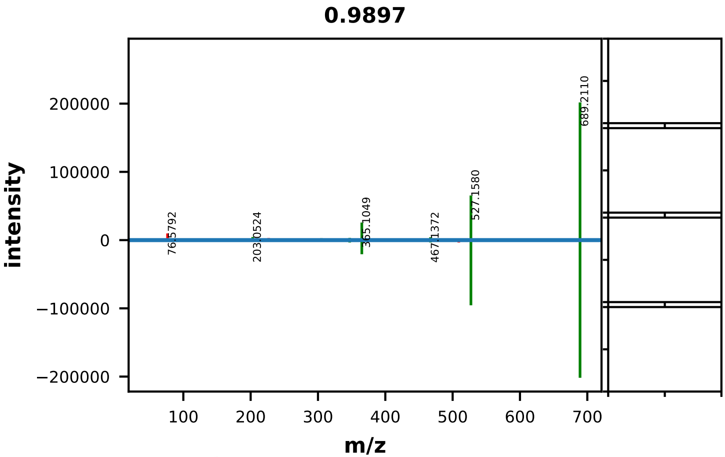

	EMA Compound Info
Name:	stachyose
Label:	
Formula:	C24H42O21
Polarity:	positive
Monoisotopic Mass:	666.221858364
Theoretical M/Z:	689.21108
Adduct:	[M+Na]+

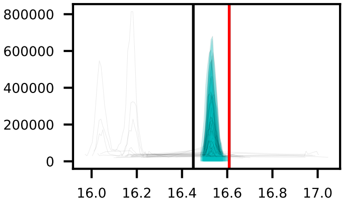

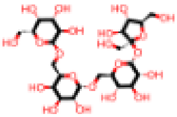

20240411_JGI_GC_509610_DWeedSyn_final_EXP120B_HILICZ_USHXG02558_POS_MS2_30-UN-35_SP9509-mP65_5_Rg70to1050-CE205060norm-root-S1_Run233.h5

0041_stachyose_positive_M+Na689p2111_16p61 [M+Na]+
Measured M/Z = 689.2109, 0.2047 ppm difference
Expected Elution of 16.61 minutes, 16.53 min actual

MSMS Scan at 16.534 minutes

Matching M/Zs above 1E-3*max: 203.052, 305.085, 347.094, 365.105, 467.137, 527.158, 689.211

All Matching M/Zs: 203.052, 305.085, 347.094, 365.105, 467.137, 527.158, 689.211
