# Supplementary material for: The metabolic and anatomical complexity of root microhabitats modulate their interaction with the microbiota
Source: Nat Commun. 2026 Aug 13;17:9738. doi: 10.1038/s41467-026-76440-4 (PMC13562559; doi:10.1038/s41467-026-76440-4)
Supplement: Supplementary file 5 [file 41467_2026_76440_MOESM12_ESM.zip › Supplementary Data 10/POS_msms_mirror_plots/0008_sphinganine_positive_M+H302p3054_4p11.pdf]

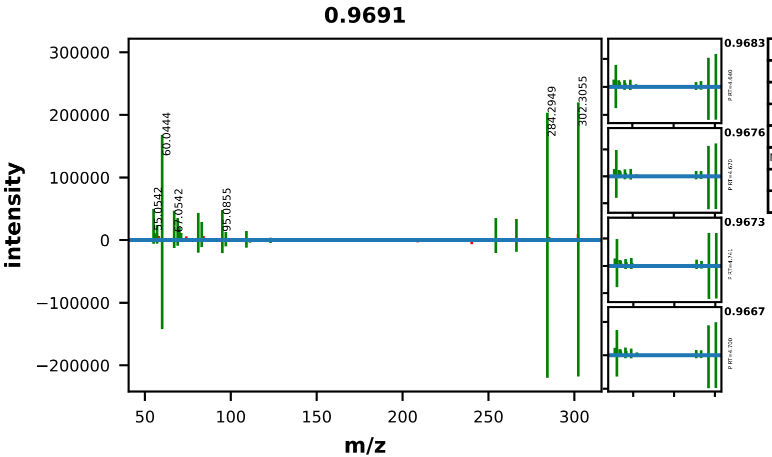

	EMA Compound Info
Name:	sphinganine
Label:	
Formula:	C18H39NO2
Polarity:	positive
Monoisotopic Mass:	301.298079488
Theoretical M/Z:	302.30536
Adduct:	[M+H]+

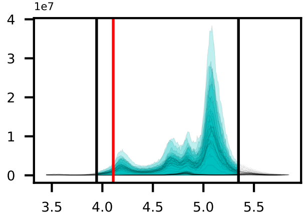

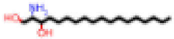

20240411_JGI_GC_509610_DWeedSyn_final_EXP120B_HILICZ_USHXG02558_POS_MS2_42-UN-49_LJ9250-NB_2_Rg70to1050-CE205060norm-root-S1_Run83.h5

0008_sphinganine_positive_M+H302p3054_4p11 [M+H]+
Measured M/Z = 302.3054, 0.0100 ppm difference
Expected Elution of 4.11 minutes, 5.07 min actual

MSMS Scan at 4.665 minutes

Matching M/Zs above 1E-3*max: 55.054, 56.049, 57.070, 60.044, 67.054, 69.070, 70.065, 71.049, 71.086, 81.070, 83.086, 95.086, 97.101, 109.101, 111.117, 123.117, 137.133, 254.284, 266.284, 267.269, 284.295, 302.305

All Matching M/Zs: 55.054, 56.049, 57.070, 60.044, 67.054, 69.070, 70.065, 71.049, 71.086, 81.070, 83.086, 95.086, 97.101, 109.101, 111.117, 123.117, 137.133, 254.284, 266.284, 267.269, 284.295, 302.305
