# Supplementary material for: The metabolic and anatomical complexity of root microhabitats modulate their interaction with the microbiota
Source: Nat Commun. 2026 Aug 13;17:9738. doi: 10.1038/s41467-026-76440-4 (PMC13562559; doi:10.1038/s41467-026-76440-4)
Supplement: Supplementary file 5 [file 41467_2026_76440_MOESM12_ESM.zip › Supplementary Data 10/POS_msms_mirror_plots/0027_2_3cyclic_AMP_positive_M+H330p0598_10p46.pdf]

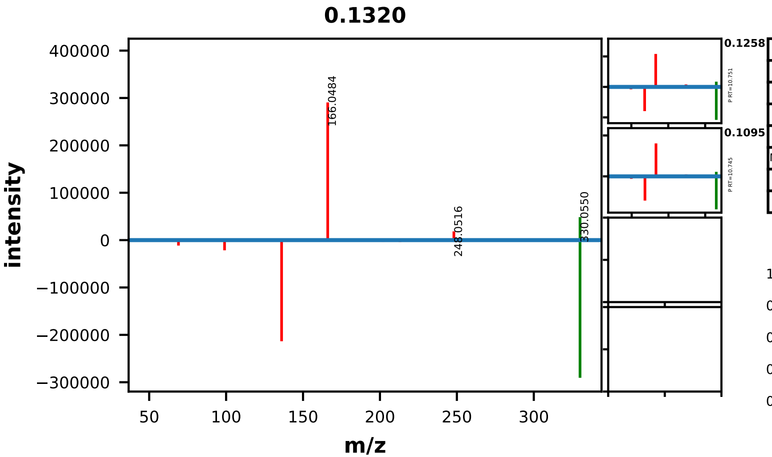

	EMA Compound Info
Name:	2',3'-cyclic AMP
Label:	
Formula:	C10H12N5O6P
Polarity:	positive
Monoisotopic Mass:	329.052519734
Theoretical M/Z:	330.0598
Adduct:	[M+H]+

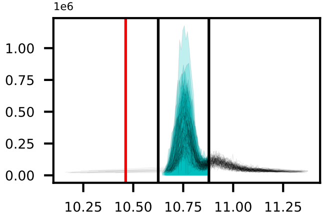

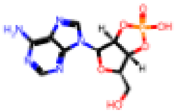

20240411_JGI_GC_509610_DWeedSyn_final_EXP120B_HILICZ_USHXG02558_POS_MS2_73-UN-88_ExCtrl_1_Rg70to1050-CE102040norm-root-S1_Run19.h5

0027_2_3cyclic_AMP_positive_M+H330p0598_10p46 [M+H]+
Measured M/Z = 330.0545, 16.0143 ppm difference
Expected Elution of 10.46 minutes, 10.76 min actual

MSMS Scan at 10.740 minutes

Matching M/Zs above 1E-3*max: 330.055

All Matching M/Zs: 330.055
