# Supplementary material for: The metabolic and anatomical complexity of root microhabitats modulate their interaction with the microbiota
Source: Nat Commun. 2026 Aug 13;17:9738. doi: 10.1038/s41467-026-76440-4 (PMC13562559; doi:10.1038/s41467-026-76440-4)
Supplement: Supplementary file 5 [file 41467_2026_76440_MOESM12_ESM.zip › Supplementary Data 10/POS_msms_mirror_plots/0000_indole-3-acetaldehyde_positive_M+H160p0757_1p24.pdf]

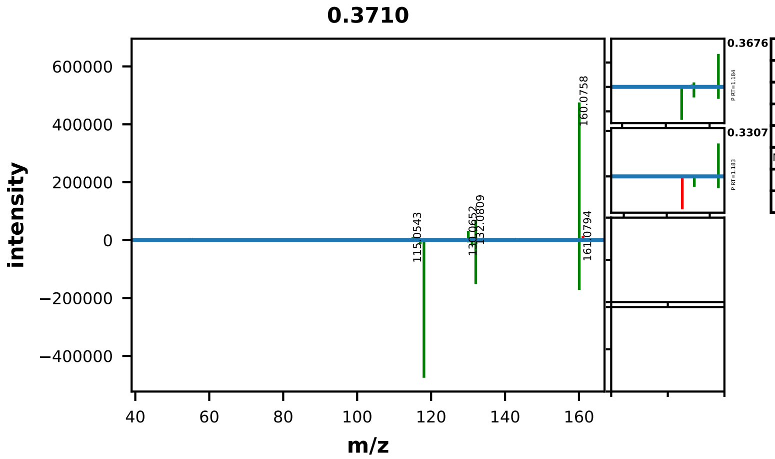

	EMA Compound Info
Name:	indole-3-acetaldehyde
Label:	
Formula:	C10H9NO
Polarity:	positive
Monoisotopic Mass:	159.068413908
Theoretical M/Z:	160.07569
Adduct:	[M+H]+

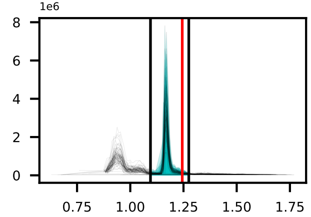

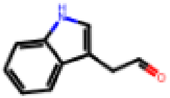

20240411_JGI_GC_509610_DWeedSyn_final_EXP120B_HILICZ_USHXG02558_POS_MS2_54-UN-64_LJ9250-dA289_2_Rg70to1050-CE205060norm-root-S1_Run107.h5

0000_indole-3-acetaldehyde_positive_M+H160p0757_1p24 [M+H]+
Measured M/Z = 160.0757, 0.2385 ppm difference
Expected Elution of 1.24 minutes, 1.16 min actual

MSMS Scan at 1.183 minutes

Matching M/Zs above 1E-3*max: 105.070, 115.054, 117.057, 118.065, 130.065, 131.073, 132.081, 142.065, 160.076

All Matching M/Zs: 105.070, 115.054, 117.057, 118.065, 130.065, 131.073, 132.081, 142.065, 160.076
