# Supplementary material for: The metabolic and anatomical complexity of root microhabitats modulate their interaction with the microbiota
Source: Nat Commun. 2026 Aug 13;17:9738. doi: 10.1038/s41467-026-76440-4 (PMC13562559; doi:10.1038/s41467-026-76440-4)
Supplement: Supplementary file 5 [file 41467_2026_76440_MOESM12_ESM.zip › Supplementary Data 10/POS_msms_mirror_plots/0009_choline_o-sulfuric_acid_positive_M184p0638_4p21.pdf]

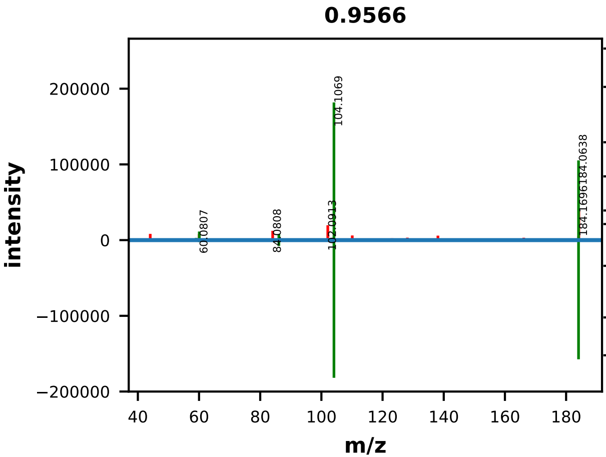

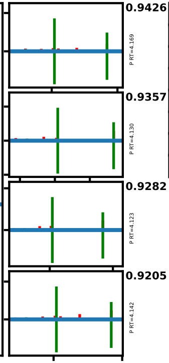

	EMA Compound Info
Name:	choline o-sulfuric acid
Label:	
Formula:	C5H14NO4S+
Polarity:	positive
Monoisotopic Mass:	184.06380534809
Theoretical M/Z:	184.06381
Adduct:	[M]+

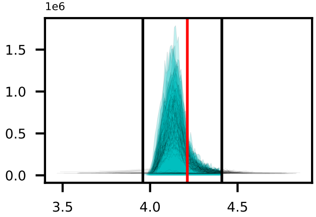

20240411_JGI_GC_509610_DWeedSyn_final_EXP120B_HILICZ_USHXG02558_POS_MS2_50-UN-59_LJ9250-dP65_2_Rg70to1050-CE205060norm-root-S1_Run71.h5

0009_choline_o-sulfuric_acid_positive_M184p0638_4p21 [M]+
Measured M/Z = 184.0638, 0.2414 ppm difference
Expected Elution of 4.21 minutes, 4.14 min actual

MSMS Scan at 4.107 minutes

Matching M/Zs above 1E-3*max: 58.065, 59.060, 60.081, 86.096, 104.107, 184.064

All Matching M/Zs: 58.065, 59.060, 60.081, 86.096, 104.107, 184.064
